# Accumulation of Deleterious Passenger Mutations Is Associated with the Progression of Hepatocellular Carcinoma

**DOI:** 10.1371/journal.pone.0162586

**Published:** 2016-09-15

**Authors:** Magdalena A. Budzinska, Thomas Tu, William M. H. d’Avigdor, Geoffrey W. McCaughan, Fabio Luciani, Nicholas A. Shackel

**Affiliations:** 1 Centenary Institute, University of Sydney, Sydney, NSW, Australia; 2 Sydney Medical School, University of Sydney, Sydney, NSW, Australia; 3 School of Medical Sciences, The University of New South Wales, Sydney, NSW, Australia; 4 A.W. Morrow Gastroenterology and Liver Centre, Royal Prince Alfred Hospital, Sydney, NSW, Australia; Universitatsmedizin Greifswald, GERMANY

## Abstract

In hepatocellular carcinoma (HCC), somatic genome-wide DNA mutations are numerous, universal and heterogeneous. Some of these somatic mutations are drivers of the malignant process but the vast majority are passenger mutations. These passenger mutations can be deleterious to individual protein function but are tolerated by the cell or are offset by a survival advantage conferred by driver mutations. It is unknown if these somatic deleterious passenger mutations (DPMs) develop in the precancerous state of cirrhosis or if it is confined to HCC. Therefore, we studied four whole-exome sequencing datasets, including patients with non-cirrhotic liver (n = 12), cirrhosis without HCC (n = 6) and paired HCC with surrounding non-HCC liver (n = 74 paired samples), to identify DPMs. After filtering out putative germline mutations, we identified 187±22 DPMs per non-diseased tissue. DPMs number was associated with liver disease progressing to HCC, independent of the number of exonic mutations. Tumours contained significantly more DPMs compared to paired non-tumour tissue (258–293 per HCC exome). Cirrhosis- and HCC-associated DPMs do not occur predominantly in specific genes, chromosomes or biological pathways and the effect on tumour biology is presently unknown. Importantly, for the first time we have shown a significant increase in DPMs with HCC.

## Introduction

Hepatocellular carcinoma (HCC) is a common cancer with 500,000–1,000,000 new cases annually, leading to ~600,000 deaths each year [1–3]. While surgical treatments are effective with early detection (70% 5-year survival), HCC diagnosis typically occurs in the late stages when no curative therapies exist [4–6], leading to a poor (<20%) 5-year survival rate [7, 8]. HCC typically occurs after decades of progressive chronic liver injury, caused by 3 main risk factors: (1) chronic hepatitis B and C virus (HBV and HCV) infection; (2) chronic alcohol consumption; and (3) exposure to the food-borne mycotoxin aflatoxin B1 (AFB1) [9, 10].

As in other cancers, HCC is associated with the accumulation of genetic alterations in cancer driver genes. However, whole exome sequencing (WES) and whole genome sequencing (WGS) studies searching for genes responsible for tumour initiation have shown that HCC is a heterogeneous disease, and no driver mutation is necessary or sufficient for carcinogenesis [11–17]. For example, while mutations are commonly found in *hTERT*, *β-catenin*, and *p53*-dependent pathways [18–20], these mutations are also found in surrounding non-tumour tissue [21–24].

Much focus has been committed to identifying genetic variants common in different tumours or in HCC subtypes [25]. This approach ignores the majority of somatic variants unique to each patient, known as passenger mutations [26]. These stochastic mutations are more likely to be either neutral or deleterious than advantageous [27]. Passenger mutations observed in cancer biology are generally assumed to be neutral and to not play a role in cancer evolution. Deleterious passenger mutations (DPMs, defined as non-driver mutations that cause a deleterious effect on protein function) that confer a profound survival disadvantage would see the clone eliminated and thus are not easily detected. However, DPMs with only moderate effect may lead to changes in protein function that are tolerated due to a previously acquired survival advantage (provided, for example, by a driver mutation).

DPM accumulation has been observed in cancer mutations curated by Catalogue of Somatic Mutations in Cancer (COSMIC) and The Cancer Genome Atlas (TCGA), revealing that DPMs with moderate effect can evade deletion through selection and accumulate during the neoplastic progression [28]. While these studies have focused on patients in whom cancer has already occurred, we and others have shown that significant clonal expansion of histologically normal cells occurs prior to carcinogenesis in patients with procarcinogenic diseases, including chronic HBV infection, a major risk factor for HCC [29, 30]. Therefore, DPMs could also accumulate in precancerous liver tissues.

We hypothesise that DPMs progressively accumulate in the liver during injury progression to HCC. Further, the presence and frequency of DPMs may be a potential marker that can help estimate risk of HCC or help understand the pathobiology of the premalignant state. Here, we have analysed WES datasets of tumour and matched non-tumour adjacent liver tissue controls of HCC patients with differing aetiologies [11, 12, 17]. Further, we have generated a WES dataset of liver tissue from patients without overt liver injury and cirrhotic patients without HCC. Our results are consistent with the hypothesis that DPMs frequency increases with progression towards HCC and therefore may help identify individuals at risk of HCC.

## Materials and Methods

### Ethics Statement

Human tissue samples were obtained from Royal Prince Alfred Hospital, Sydney, Australia with approval of Human Research Ethics Committee of the Royal Prince Alfred Hospital (Protocol number X10-0072). Informed written consent was obtained from all participants.

### Whole exome sequencing (WES) datasets

The WES 1 dataset included liver tissue from 12 patients with limited levels of liver injury and 6 HCV-positive patients with liver cirrhosis (Patient characteristics are shown in Table 1). Briefly, snap-frozen liver wedge biopsies of donor (HCV-negative) and recipient (HCV-positive) liver tissue were taken during liver transplants at the Royal Prince Alfred Hospital (RPAH), Sydney. Total DNA was extracted as previously described [31]. DNA was prepared for WES using the Agilent SureSelect Human All Exon 51M enrichment kit by BGI Hong Kong. Sequence data has been deposited at the European Genome-phenome Archive (EGA), accession number PRJEB9907. Data for WES 2–5 were taken from previously published studies [11, 12, 17, 32]. Further information is available in S1 Supplementary Methods.

**Table 1:** Clinical characteristics of patients analysed in WES 1: non-HCC liver injury samples.

	Sample	Sex	Age	HCV infection	HCV Genotype	METAVIR score
**Non-cirrhotic patients (n = 12)**	NC1	M	28	No	NA	0–1
	NC2	M	13	No	NA	0–1
	NC3	M	27	No	NA	0–1
	NC4	M	17	No	NA	0–1
	NC5	M	18	No	NA	0–1
	NC6	M	18	No	NA	0–1
	NC7	M	60	No	NA	0–1
	NC8	F	53	No	NA	0–1
	NC9	F	22	No	NA	0–1
	NC10	F	55	No	NA	0–1
	NC11	M	18	No	NA	0–1
	NC12	M	68	No	NA	0–1
**Cirrhotic HCV-positive patients without HCC (n = 6)**	C1	M	54	Yes	3a	4
	C2	M	57	Yes	3a	4
	C3	F	61	Yes	3a	4
	C4	M	45	Yes	3b	4
	C5	M	53	Yes	1b	4
	C6	M	59	Yes	1a	4

### Bioinformatics analysis pipeline

**Fig 1:**
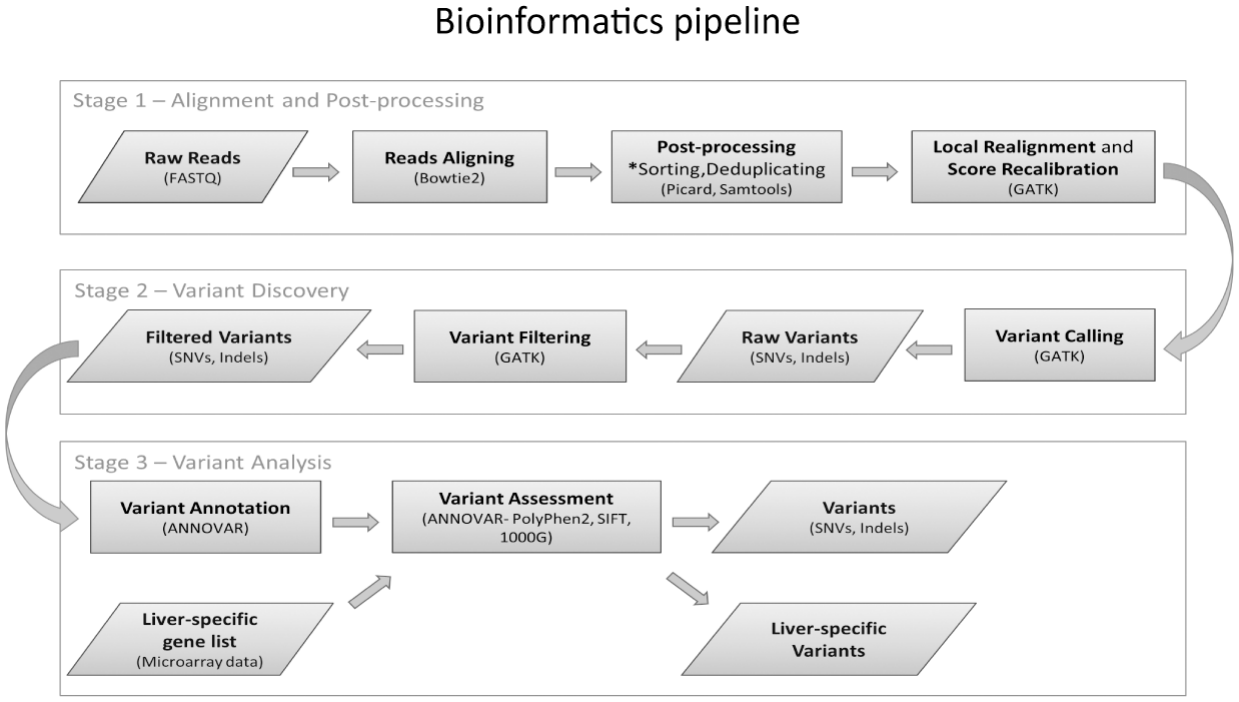
Bioinformatics analysis pipeline. Each resultant data file is indicated by a sloped rectangle and each process represented by a square rectangle. Our pipeline contains 3 stages: alignment and calibration; variant calling and filtering; and variants annotation and filtration of putative germline mutations.

Details on alignment and variant filtering are shown in Fig 1 and described in greater depth in S1 Supplementary Methods. All variants were annotated using ANNOVAR [33] with UCSC Known Gene annotation to determine the amino acid changes. Probable germline mutations were excluded by filtering out variants present in 1000 Genomes Project database [34] (v1000g2014oct). The allelic frequency of each SNV was estimated by dividing the number of reads carrying the specific SNV by the number of total reads at that position.

Variants were classified as DPMs if they met one of the following criteria:

1. missense SNVs judged as “probably damaging” or “possibly damaging” by the PolyPhen-2 algorithm [35] (PolyPhen-2 score ≥0.453) or by the SIFT algorithm [36] (SIFT score ≤0.05);
2. stop-gain or stop-loss mutations
3. frameshift indels.

Missense variants that lay outside of these criteria were classed as benign. Due to their unknown effect, non-frameshift indels were classed neither as DPMs nor benign mutations and excluded from further analysis.

### Analysis of liver-expressed genes

A list of genes expressed in the liver was generated from analysis of microarray gene expression data generated in our laboratory from total RNA extracts of non-diseased liver tissue of 6 donors (S2 Table). Specific details on analysis are given in S1 Supplementary Methods.

### Statistical analysis

Statistical analyses were carried out using PRISM 6 software (GraphPad, La Jolla, USA). The Wilcoxon matched-pairs signed-rank test was used to assess the differences between each set of paired samples (tumour vs. non-tumour) and the Mann-Whitney U test was used for unpaired samples and comparison of datasets. The association of DPMs relative to the occurrence of putative driver mutations was analysed by Spearman rank correlation coefficient test.

### Pathway and functional enrichment analysis

The Ingenuity Pathway Analysis (IPA, Ingenuity Systems, Mountain View, CA; http://www.ingenuity.com) was used to identify the pathways and biological functions of genes affected by DPMs. The significance was set at a p-value of 0.01 by the right-tailed Fisher Exact Test.

## Results

### Normalisation and identification of somatic DPMs

**Fig 2:**
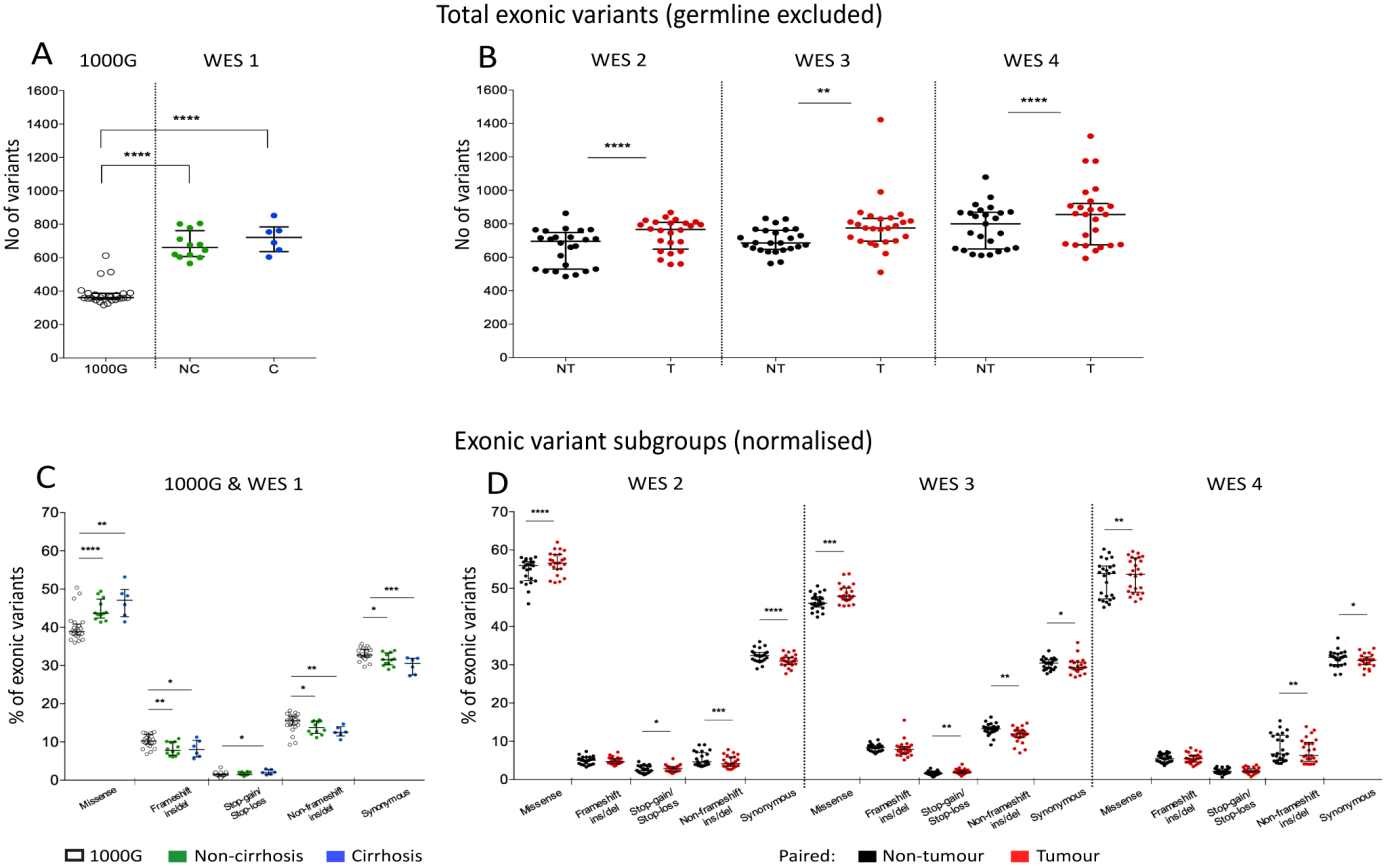
Absolute number of exonic variants and mutation subtypes in 1000G, liver injury, cirrhosis and HCC. The exonic variants in each of the 5 datasets were enumerated (A and B) and then subdivided into 5 groups (missense, frameshift ins/del, stop-gain/-loss and non-frameshift ins/del) (C and D, expressed as a percentage of all somatic exonic mutations). 1000G and WES 1 (A and C) contain unpaired samples, while WES 2–4 (B and D) are composed of paired tumour and non-tumour samples taken from the same individual. Data are expressed as median (interquartile range). * p<0.05, ** p<0.01, *** p<0.001 and **** p<0.0001, Mann-Whitney U test (1000G and WES 1) or Wilcoxon matched-pairs signed-rank test (WES 2–4). NC-non-cirrhosis; C-cirrhosis; NT-non-tumour; T-tumour.

The bioinformatics pipeline, outlined in Fig 1, was used to analyse the number of exonic variants in datasets derived from liver tissue DNA (WES 1–4) and serum DNA (1000G and WES 5). Overviews of the datasets are provided in Table 1 (WES 1), Table 2 (WES 2–4) and S1 Table (1000G). Expectedly, the number of detectable variants differed with each dataset (Fig 2A and 2B), reflecting factors such as different enrichment kits, sequencing platforms and sequencing depth. Therefore, we normalised values to the total exonic mutations for each tissue sample to reduce inter-dataset and inter-patient variation in subsequent analyses.

**Table 2:** Summary data of publicly-available WES datasets used in this study.

WES dataset	Ref.	Aetiology	n	% Male	Mean depth	Mean read length	Mean read count per sample (millions)	Platform	Enrichment kit	Location
WES 2	[11]	Alcohol (50%) HBV (4%) HCV (17%) NASH (8%) Other (29%)	24	83	x 73	Paired-end 75 bp	132.7	Illumina HiSeq2000	SureSelect Human All Exon Kit v2 (44Mb)	France
WES 3*	[12]	HBV (43%) HCV (21%)	72* 10 5	77	x 59 x 4.8 x 5.8	Paired-end 100 bp 76 bp 76 bp	62.6*	Illumina HiSeq2000 Illumina GAIIx Illumina GAIIx	SureSelect Human All Exon v4(51Mb) All exon v1 (38 Mb) NimbleGen Human Exome v1 (2.1Mb)	USA/Canada
WES 4*	[17]	NR	30*	NR	NR	Paired-end 75 bp	184.0*	Illumina HiSeq	NR	USA

*25 paired samples were used in the analysis from each of these studies to allow dataset comparisons.

NR = Not reported

We excluded potential germline mutations using the 1000 Genomes Project data. While peripheral blood mononuclear cells (PBMC) of the same patient are often used as a control for germline mutations, a number of important confounders are evident with this approach. Firstly, we had found that the mutational profile of PBMCs differs with liver injury, likely due to clonal expansion of circulating immune cells during inflammation associated with liver disease (S2 Fig). Further, somatic mutations in PBMCs acquired with age are not accounted for and may be incorrectly assumed to be germline. Finally, previously described tissue differences in somatic mutation rates and profiles may be missed [37, 38]. Thus, germline mutations were imputed using the 1000 Genomes Project data and all samples were filtered identically. The number of excluded variants was not significantly different between tumour and non-tumour samples (p>0.05, Wilcoxon signed-rank test). After filtration, only few of the variants (2.4%, 4.7%, 5.1%, 5.1%, 2.1%, 3.7% for WES 1–5, and 1000G respectively) occurred at an allelic frequency of 1.0 (S3 Fig), suggesting that the majority of homozygous germline variants have been excluded and the remainders were likely to be somatic variants.

While the numbers of somatic exonic variants (either single nucleotide variants (SNVs) or small indels) between non-cirrhotic and cirrhotic patients were not significantly different (Fig 2A, p>0.05, Mann-Whitney U test), greater numbers of variants were detected in tumour compared to non-tumour tissue (Fig 2B, p<0.0001, p<0.01, and p<0.0001 for WES 2–4 respectively, Wilcoxon signed-rank test). However, the absolute number of mutations did not consistently separate tumour and non-tumour tissue.

### Description of variants and deleterious passenger mutations (DPMs) in liver injury and HCC

Exonic variants were then classified based on their effect on open reading frames (i.e. missense mutations, stop-gain/-loss mutations, and indels with or without a frame-shift). An increase in missense and synonymous mutations was observed with liver disease progression (S4 Fig). After normalisation of each sample to the number of exonic variants (Fig 2C and 2D), we found a consistent increase of missense mutations (a mean relative increase of 3.5%, 4.3%, and 2.2% from non-tumour to tumour in WES 2–4).

**Fig 3:**
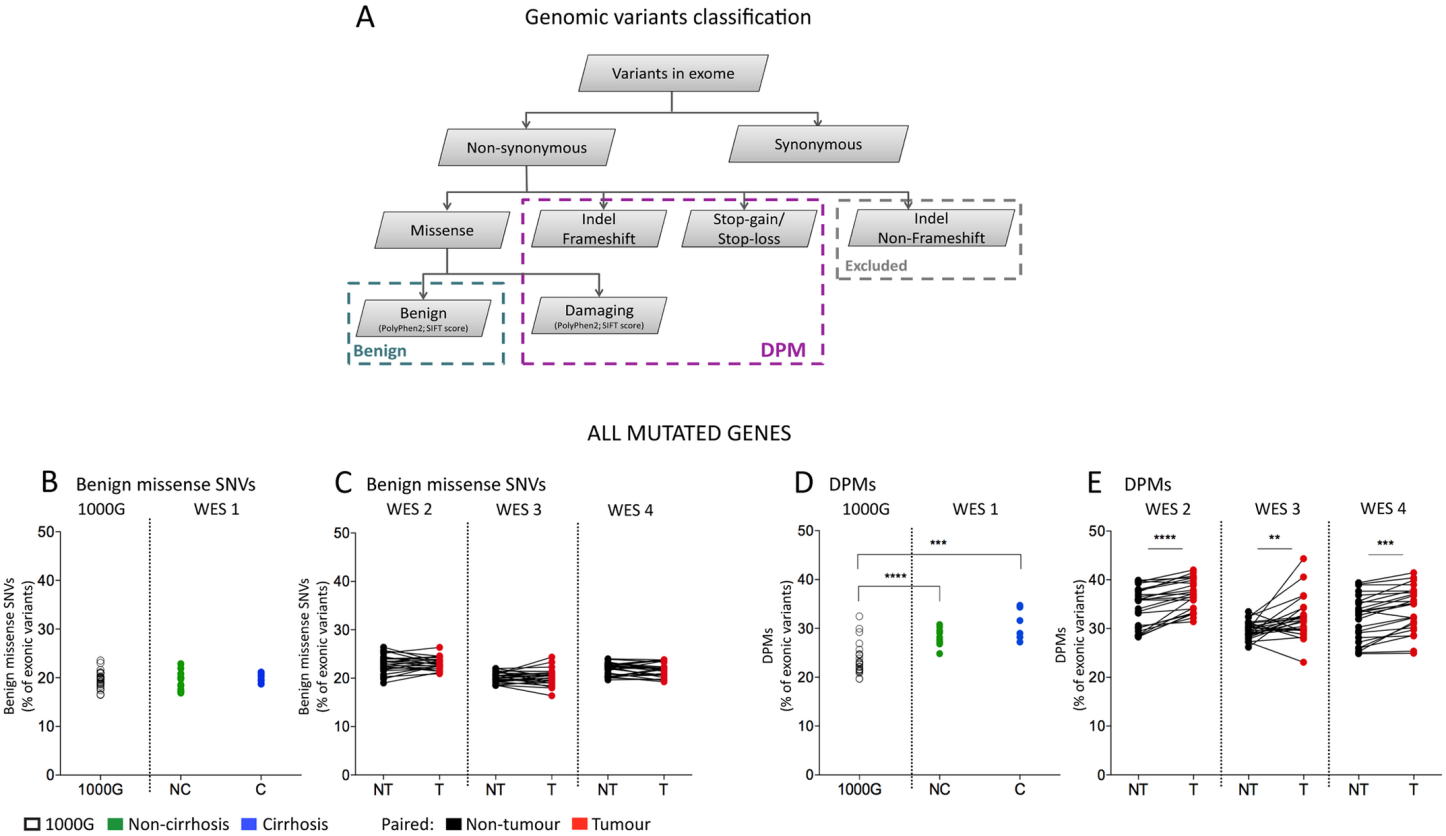
DPMs in HCC and surrounding non-tumour tissue. Variants were classified based on the predicted effect on the amino acid sequence (A). Total benign missense variants (B and D) and DPMs (C and E) in the datasets 1000G and WES 1–4 are shown as a percentage of all somatic exonic mutations. Significantly more DPMs (but not benign missense SNVs) were detected in tumour compared to paired non-tumour tissue (* p<0.05, ** p<0.01, *** p<0.001 and **** p<0.0001, Wilcoxon matched-pairs signed-rank test). Lines link matched non-tumour and tumour tissues samples. NC-non-cirrhosis; C-cirrhosis; NT-non-tumour; T-tumour.

To test the hypothesis that DPMs accumulate in the development of HCC, we examined the percentage of benign SNVs and DPMs in 1000G, liver injury, cirrhosis and paired non-tumour and tumour samples (Fig 3). Our classification of benign SNVs and DPMs is shown in Fig 3A. Briefly, exonic mutations predicted to affect protein function (including stop-gain/-loss, frame-shift mutations and those judged to be damaging by PolyPhen2 or SIFT algorithms) were classified as DPMs. Benign missense SNVs were also classified by PolyPhen2 or SIFT algorithms. In the majority of patients (91.7%, 72% and 88% for WES 2, WES 3 and WES 4 respectively), more DPMs were observed in tumours compared to surrounding non-tumour tissue (Fig 3B–3E, p<0.01, Wilcoxon signed-rank test). The mean relative increase in DPMs was 7.1%, 7.8% and 4.4% from non-tumour to tumour samples for WES 2–4, respectively. However, no significant differences were observed in benign missense SNVs, suggesting that the observed accumulation occurred specifically in DPMs. Similar results were seen when the SIFT algorithm was used (S6 Fig).

We estimated the allelic frequency of benign missense SNVs and DPMs in each patient by the ratio of wild type to mutated reads. The allelic frequency distributions of variants were similar between benign missense SNVs and DPMs for any given patient or disease stage including HCC (S3 Fig). Further, using available clinical data, we showed that DPM accumulation did not significantly correlate with patient age, cause of liver disease or tumor size (WES 2: R^2^ = 0.09–0.12 and p = 0.24–0.3). Therefore, DPMs appear to accumulate from the non-tumour to tumour progression of HCC irrespective of a range of clinical features, and so may represent a general phenomenon in hepatocarcinogenesis.

DPM accumulation was observed even when the analysis was restricted to genes expressed in the liver (S5 Fig and S2 Table). As genes containing some DPMs may not be expressed (and so do not alter cell phenotype), we excluded mutations within genes not expressed in liver tissue (S2 Table). After filtration, significantly more DPMs (but not benign missense SNVs) were still observed in tumours compared to surrounding non-tumour tissue (S5D Fig, p<0.01, Wilcoxon signed-rank test). Further, DPM accumulation in patients without HCC (WES 1) was significantly lower compared to both tumour (for WES 2 and 4) and non-tumour samples (for WES 2) in HCC patients (Table 3). In summary, the accumulated DPMs potentially generate a novel phenotype within the liver cells containing them due to alterations in encoded protein function.

**Table 3:** Summary statistics for normalised DPMs between datasets.

			**Total**		
			**1000G**	**WES 1**	
				**NC**	**C**
		**Mean (**±**SD)**	23.60 (±3.18)	28.17 (±1.71)	30.85 (±3.22)
**1000G**		23.60 (±3.18)	NA	p<0.0001	p<0.001
**WES 2**	**NT**	34.34 (±4.07)	p<0.0001	p<0.0001	p<0.05
	**T**	36.78 (±3.19)	p<0.0001	p<0.0001	p<0.01
**WES 3**	**NT**	29.76 (±1.8)	p<0.0001	p<0.05	p = 0.57
	**T**	32.07 (±4.27)	p<0.0001	p<0.001	p = 0.49
**WES 4**	**NT**	32.11 (±4.7)	p<0.0001	p<0.05	p = 0.55
	**T**	33.54 (±5.23)	p<0.0001	p<0.01	p = 0.12
			**Liver-specific**		
			**1000G**	**WES 1**	
				**NC**	**C**
		**Mean (**±**SD)**	8.41 (±1.87)	14.56 (±1.48)	16.11 (±2.18)
**1000G**		8.41 (±1.87)	NA	p<0.0001	p<0.0001
**WES 2**	**NT**	19.35 (±2.54)	p<0.0001	p<0.0001	p<0.01
	**T**	20.80 (±2.34)	p<0.0001	p<0.0001	p<0.001
**WES 3**	**NT**	14.92 (±1.5)	p<0.0001	p = 0.44	p = 0.25
	**T**	16.44 (±2.66)	p<0.0001	p<0.05	p = 0.98
**WES 4**	**NT**	17.60 (±3.08)	p<0.0001	p<0.01	p = 0.19
	**T**	18.54 (±2.98)	p<0.0001	p<0.001	p<0.05

### The accumulation of DPMs in non-tumour tissue and its relationship with putative driver mutations

**Fig 4:**
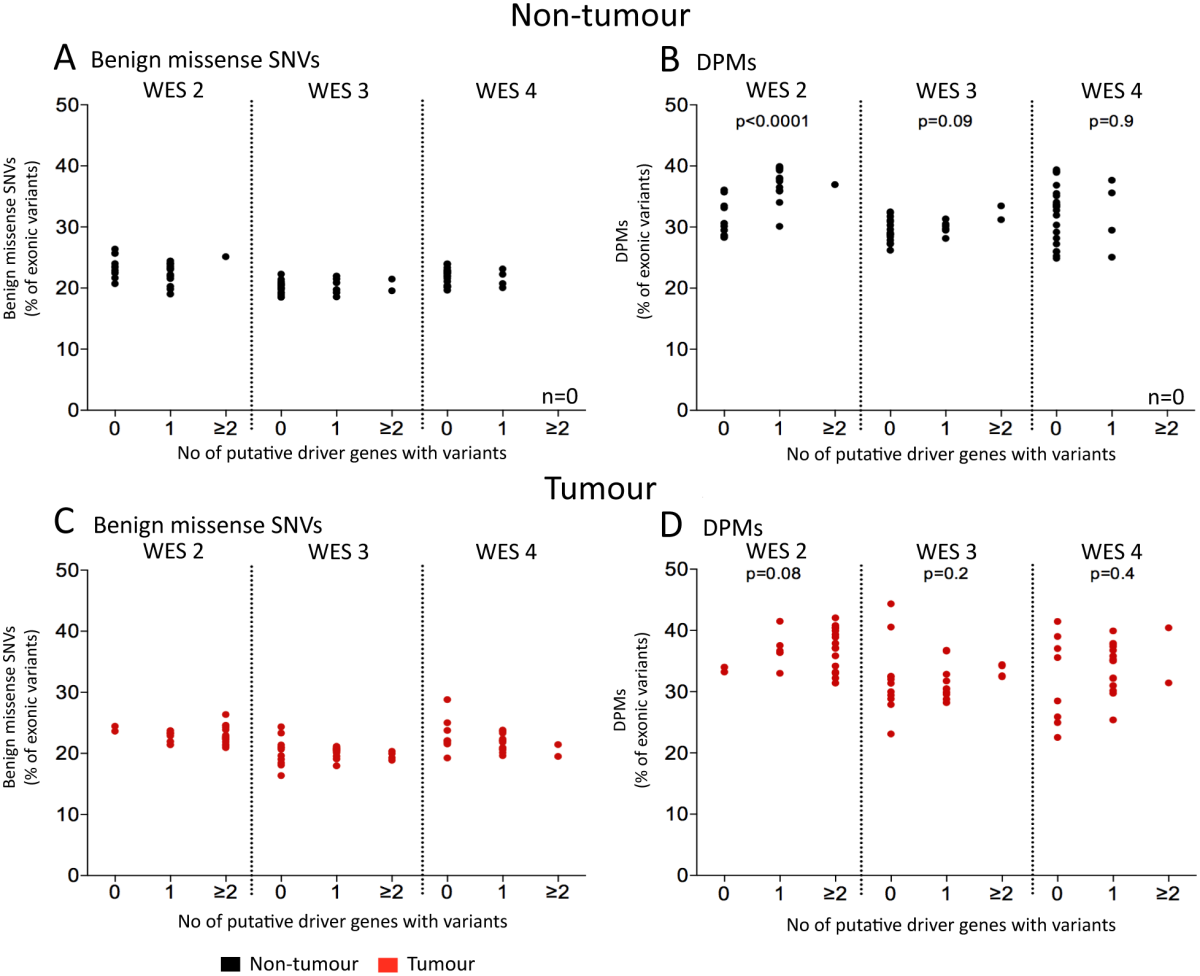
Driver mutations in non-tumour tissue. Patient samples were separated based on the number of mutations in putative driver genes (x-axis, defined as the 20 top recurrently mutated genes in HCC according to COSMIC database, listed in S3 Table) and analysed the number of benign missense SNVs (A and C) and DPMs (B and D). Significant correlation between DPMs and putative driver mutations (p<0.0001, Spearman rank correlation test) was observed in non-tumour tissue of WES 2. No significant correlation was seen in HCC tissues (p>0.05, Spearman rank correlation test).

We tested the possibility that increasing DPMs were associated with accumulation of putative driver mutations and the development of HCC. We did not see a consistent association after analysis of putative HCC driver mutations in HCC patient datasets (Fig 4). Putative HCC driver genes were defined in this case as the 20 most frequently mutated genes in HCC tissues as retrieved from the COSMIC database (listed in S3 Table). The least frequent driver mutation in this list occurs at ~2%, and thus would not be expected to occur in our dataset more than once. These putative driver mutations occurred between 0 to 2 times per tissue, consistent with previous studies showing that drivers are relatively rare and that passenger mutations outnumber them by up to 2 orders of magnitude [39, 40].

Putative driver mutations were seen in both tumour and non-tumour tissue (S3 Table). Although, we observed both damaging and benign mutations in putative HCC driver genes repeated in HCC tissue, mutations in the majority of these genes (except for *CTNNB1* in dataset WES 2 and *TP53* in datasets WES 2, 3 and 4) were also observed at similar frequencies in the surrounding non-tumour tissue (S3 Table). Further, the average allelic frequency (as estimated by the ratio of wild type to mutated reads) of the mutations in the putative driver genes did not appear to differ between tumour and non-tumour samples (data not shown).

In the non-tumour tissue of WES 2 (but not WES 3 or 4), we observed a significantly greater proportion of DPMs with an increasing number of damaging mutations in driver genes (Fig 4B, p<0.0001, p = 0.095, and p>0.1 respectively, Spearman rank correlation coefficient test). We repeated this analysis on the tumour tissue and observed no significant association between detected driver mutations and either benign missense SNVs or DPMs (Fig 4C and 4D, p>0.1, Spearman rank correlation coefficient test). This was expected, as all tumours presumably have gained sufficient driver mutations (though not observable using the NGS data) to have proceeded to HCC. As a control, we performed the same analysis (n = 10), but using 20 randomly selected genes containing DPMs instead of known driver genes and observed no significant DPM increase in any datasets (data not shown). Together, these findings are consistent with the hypothesis that the surrounding non-tumour tissue is not necessarily normal and can contain precancerous changes.

### The majority of DPMs are likely to be true passenger mutations

**Fig 5:**
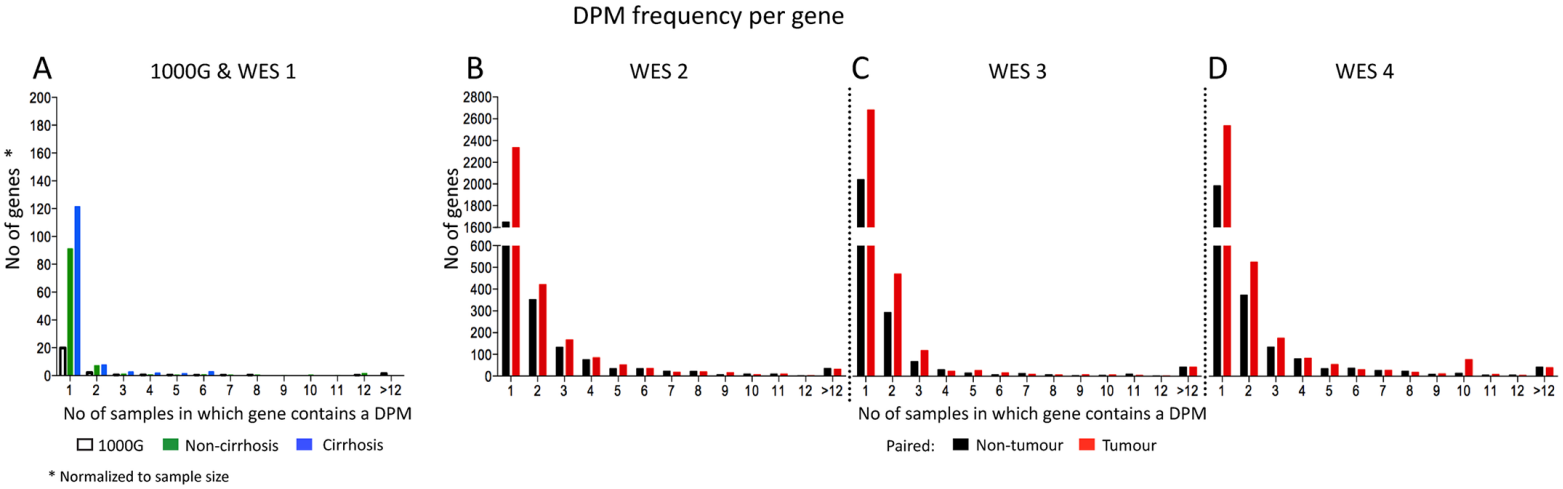
Frequency distribution of DPMs. A frequency distribution of the genes containing DPMs in 1000G and WES 1 (A), WES 2 (B), WES 3 (C), and WES 4 (D) shows that most are unique to a given patient. Each gene containing a DPM was grouped based on the number of patients in which that gene contained a DPM (x-axis).

The majority of DPMs seen were not shared between patients (Fig 5). Pooled DPMs from all samples for each dataset were analysed to determine if the accumulated DPMs represented potential novel driver genes. We found that the majority (>70% for each dataset) of genes with DPMs were not recurrent and instead DPMs occurred in unique locations for each patient (Fig 5), consistent with random accumulation. Further, the chromosomal distribution of DPMs (S7 Fig) showed broad occurrence throughout the genome, without any obvious hotspots.

Pathway enrichment analysis showed that there was significant enrichment (p<0.01, right-tailed Fisher Exact Test) of DPMs in some functional biological pathways in both tumour and non-tumour samples (S4 Table). However, only a minority of DPMs contributed to these pathways: 0%, 0.61%, 1.7%, 0.36%, 0.44% in non-tumour tissues in WES 1, 2, 3, and 4, respectively; and 2.8%, 2.4%, 0.49%, in tumour tissues of WES 2, 3, and 4, respectively. Even if these DPMs in these functional pathways all represented novel driver mutations, this is still insufficient to explain the increase in DPMs associated with liver disease progression, which had a mean relative increase in DPMs of 7.1%, 7.8% and 4.4% from non-tumour to tumour tissues in WES 2, 3, and 4, respectively. In summary, these results suggest that the increased frequency of DPMs in tumour compared to non-tumour is due to stochastic accumulation of passenger mutations. Further, the difference in DPM load between tumour and non-tumour samples does not likely represent a gain in novel driver mutations.

## Discussion

This is the first NGS study to our knowledge to DNA sequence normal liver tissue and recognise that there are exome-wide DNA alterations in liver tissues prior to carcinogenesis. Our focus was on DPMs (defined as randomly-acquired somatic mutations that altered protein function), which composed of approximately a third of all somatic variants. Our key finding shows that an increase in DPMs is associated with progressively worse liver disease leading up to HCC. This was also observed even when genes not expressed in liver tissue (measured by microarray analysis) were excluded.

DPMs could be promoting tumour development in these tissues, but we could not find evidence of this occurring. The majority of DPMs were not found to occur predominantly in any specific genes, chromosomes or biological pathways. While this may be explained in part by the poor recognition and understanding of such pathways, given the rarity of tumour suppressors and the observed overall progressive accumulation of DPMs, our data would suggest that DPMs are randomly acquired and true passenger mutations rather than uncharacterised drivers of HCC.

The pattern of observed DPMs in HCC is consistent over multiple algorithms for scoring deleterious effect, in multiple aetiologies of HCC, and in multiple datasets with different ethnic compositions. Further, the observed DPMs are not at a low frequency (S3 Fig), which would be seen in sporadic occurrences, as they have the same overall allelic frequency as benign missense mutations. This suggests that DPM accumulation is a general mechanism accompanying tumour evolution and agrees with the theory that DPMs accumulate during the evolution of preneoplastic HCC subclones [28]. Our comparison with DNA extracted from normal tissue suggests that genetic changes have occurred in non-tumour hepatocytes in patients with HCC. These results are consistent with mathematical models suggesting that >50% of exonic mutations occur prior to carcinogenesis [41] and observations of *TERT* promoter mutations in preneoplastic nodules [42]. Here, we extend these studies showing that many driver mutations are observed in histologically-normal non-tumour tissue. Mutations that occur prior to tumourigenesis should not be ignored as they may contribute to the carcinogenic process.

**Fig 6:**
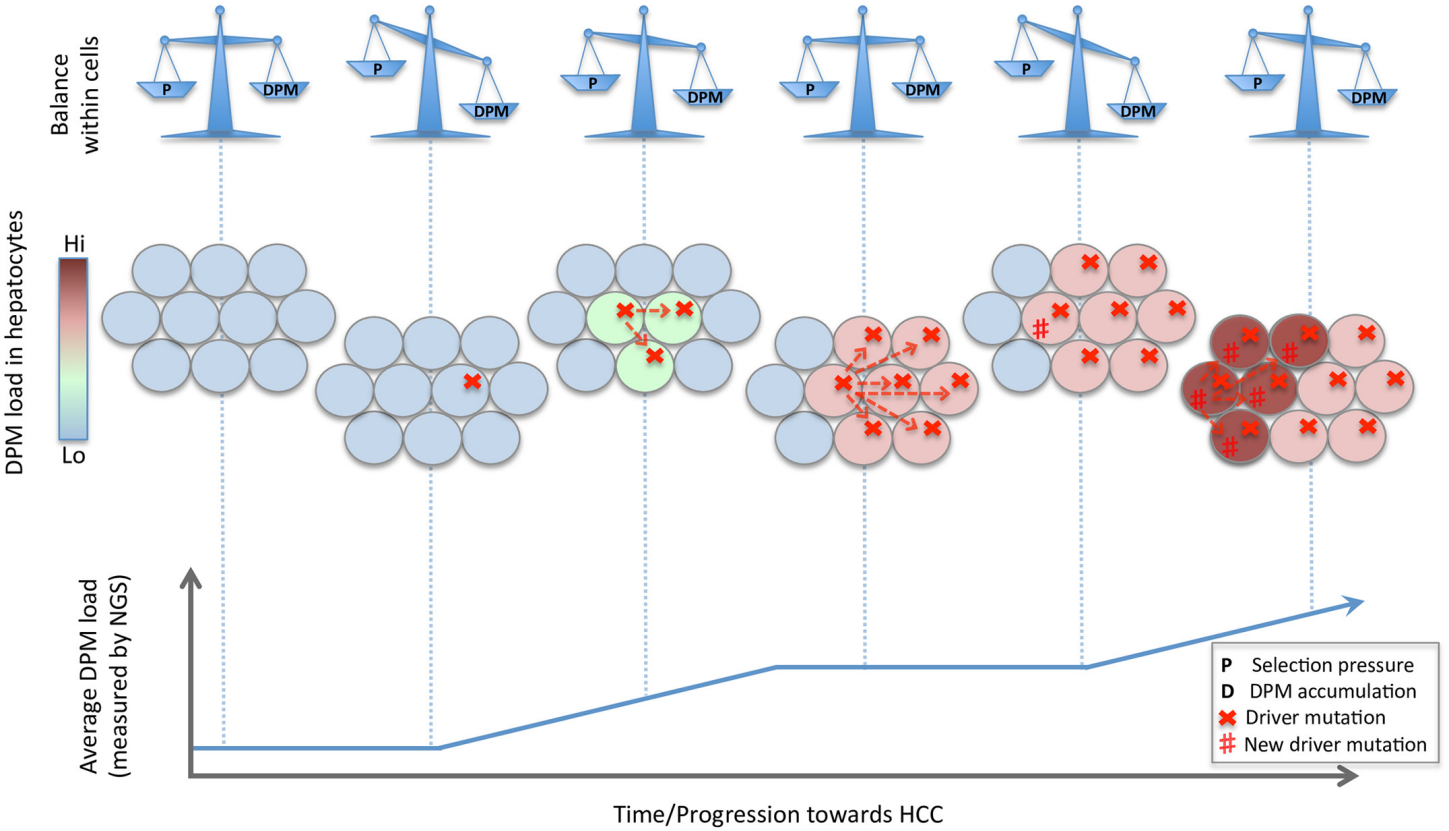
Hypothetical model of HCC progression. HCC progression is presented here as multiple waves of driver sweeps within hepatocyte subclones. The equilibrium between DPM accumulation and negative selection on the hepatocyte subclones are shown in the top row. A schematic model of the liver (with each circle representing a hepatocyte and the colour gradient representing the DPM load within each hepatocyte) is shown in the centre row. The average DPM load for the tissue is depicted in the bottom row.

Based on our findings we propose the following model of HCC development (Fig 6):

1. Hepatocyte subclones acquire driver mutations through random mutation, giving them a survival advantage.
2. This disrupts the selection equilibrium in favour of DPM acquisition.
3. Equilibrium is restored when selection against the accumulated DPMs evens out the survival advantage.
4. The hepatocyte subclone population plateaus until the next driver mutation.
5. Steps 1–4 are repeated, eventually culminating into HCC through acquiring sufficient driver mutations.

This model suggests that DPMs could form the basis of a genetic biomarker, though our results suggest that interpatient variability is considerable and it may be of limited use as a measure of HCC risk. However, the data raises the intriguing possibility that cirrhosis progression with increasing DPM accumulation may be a risk factor or signature for HCC development. Further, it is unclear if certain subsets of DPMs may predict aspects of tumour biology and/or behaviour. These possibilities are difficult to investigate, as they require serial sampling in humans over months to many years. Animal models with their lack of cirrhosis associated with HCC as well the use of agents that globally damage DNA such as diethyl-nitrosamine (DEN) are poor surrogates to answer these questions. In future studies (especially as more sequencing data becomes publically available), larger patient cohorts, serial samples and a better understanding of deleterious effects of DNA mutations on liver cell phenotype will allow better tests for this hypothetical model.

In summary, we have shown that progressive liver injury and HCC are accompanied by accumulation of DPMs. We also have provided evidence that surrounding non-tumour tissue is not genetically “normal”. While the true effect of accumulated DPMs on tumour biology is still unknown, given their frequency and functional implications, they cannot be ignored

## Supporting Information

S1 Fig Summary statistics of WES 1 reads. (A) Mean depth of reads for each sample, (B) Fraction of target covered in caption region (4-fold, 10-fold and 20-fold coverage) per exome.
(TIFF)

S2 Fig Comparison of benign missense variants and DPMs in 1000G and WES 5 datasets. To determine if circulating leukocytes could be used as a control to account for germline mutations in individuals, we compared DNA from peripheral blood mononuclear cells (PBMCs) in 1000G and patients with hepatitis B virus (HBV) infection (WES 5) [32]. We found significantly more benign mutations (A) and DPMs (B) in HBV-exposed patients compared to healthy people from the 1000G dataset (*p<0.05, ****p<0.0001, Mann-Whitney test), suggesting the DNA genome of PBMCs are altered as a result of HBV infection. This may be due many factors dependent on HBV-associated inflammation, including: DNA mutations introduced during high levels of PBMC mitosis; or DPMs being accumulating as a result of clonal expansion of PBMCs. Greater immune activation associated with HBV infection would be expected to increase clonal expansion, and therefore DPMs according to our model. Crucially, this result suggests that liver disease causes changes in the DNA within the blood (not just the liver) and so using PBMC-derived DNA sequences to exclude germline variants would introduce bias. This therefore justifies our approach of using only the 1000 Genomes Project database to exclude probable germline mutations.
(TIFF)

S3 Fig Estimated allelic frequency distribution of benign missense variants and DPMs. The allelic frequency of each benign missense variant (left) and DPMs (right) was estimated by the number of reads containing the variant divided by the number of the total reads at that particular base (x-axis). This was expressed as a cumulative plot with each patient as different colours for all benign missense variants and DPMs for 1000G and WES 1–4 (top, middle and bottom respectively). For WES 2–4, paired tumour (solid line) and non-tumour (dashed line) for the same patient are coloured the same colour.
(TIFF)

S4 Fig Absolute number of variants. Non-synonymous mutations for all datasets were subdivided into 4 groups: missense, non-frameshift ins/del, frameshift ins/del and stop-gain/-loss. Samples in 1000G and WES 1 are unpaired, while samples in WES 2–4 paired. After excluding probable germline mutations, absolute numbers of variants (A and B) are shown for each sample. * p<0.05, ** p<0.01, *** p<0.001 and **** p<0.0001, Mann-Whitney U test (1000G and WES 1) or Wilcoxon matched-pairs signed-rank test (WES 2–4).
(TIFF)

S5 Fig DPMs in HCC and surrounding non-tumour tissue in genes expressed in non-diseased liver. We analysed benign missense variants (A and B) and DPMs (C and D) in genes expressed in non-diseased liver tissue (measured by microarray analysis). The significant increase in DPMs in tumour tissue compared to paired non-tumour tissue was maintained (** p<0.01, *** p<0.001 and **** p<0.0001, Wilcoxon matched-pairs signed-rank test). No significant differences in benign missense variants or DPMs were detected between non-cirrhotic and cirrhotic patients (p>0.05, Mann-Whitney U test).
(TIFF)

S6 Fig Comparison of benign missense variants and DPMs using SIFT algorithm. We compared total benign missense variants (A and B) and DPMs (C and D) in the datasets 1000G and WES 1–4. 1000G and WES 1 are unpaired and WES 2–4 paired. Significantly more DPMs (but not benign missense SNVs) were detected in tumour compared to paired non-tumour tissue (* p<0.05, ** p<0.01, *** p<0.001 and **** p<0.0001, Wilcoxon matched-pairs signed-rank test). We also analysed benign missense variants (E and F) and DPMs (G and H) in genes expressed in non-diseased liver tissue. The significant increase in tumour tissue was maintained. No significant differences in benign missense variants or DPMs were detected between non-cirrhotic and cirrhotic patients (p>0.05, Mann-Whitney U test). All variants were normalised to the total exonic variants after exclusion of probable germline mutations. Lines show linkages between matched paired non-tumour and tumour tissues samples.
(TIFF)

S7 Fig DPMs distribution throughout the exome. The genomic distribution of benign missense mutations (radial lines) are shown in Circos plot for (A) 1000G (grey) and WES 1 for non-cirrhotic (green) and cirrhotic (blue) patients, and for WES 2 (B), WES 3 (C), and WES 4 (D) for non-tumour (black) and tumour (red) tissues. The distribution of DPMs is also shown for 1000G and WES 1 (E), WES 2 (F), WES 3 (G) and WES 4 (H). The outer grey circle represents the exons location (USCS). Even distribution throughout the genome was observed given the exon distribution and the coverage of the reference sequence hg19.
(TIFF)

S1 Supplementary Methods (PDF)

S1 Table 1000 Genome samples summary. (DOCX)

S2 Table List of genes expressed above background levels detected in liver tissue of donor patients. (DOCX)

S3 Table Frequency of driver mutations in analysed samples. (DOCX)

S4 Table Significantly enriched (p≤10^−3^) canonical pathways in DPM-affected genes. (DOCX)
