# Supplementary figures and images for: Accumulation of Deleterious Passenger Mutations Is Associated with the Progression of Hepatocellular Carcinoma
Source: PLoS One. 2016 Sep 15;11(9):e0162586. doi: 10.1371/journal.pone.0162586 (PMC5025244; doi:10.1371/journal.pone.0162586)

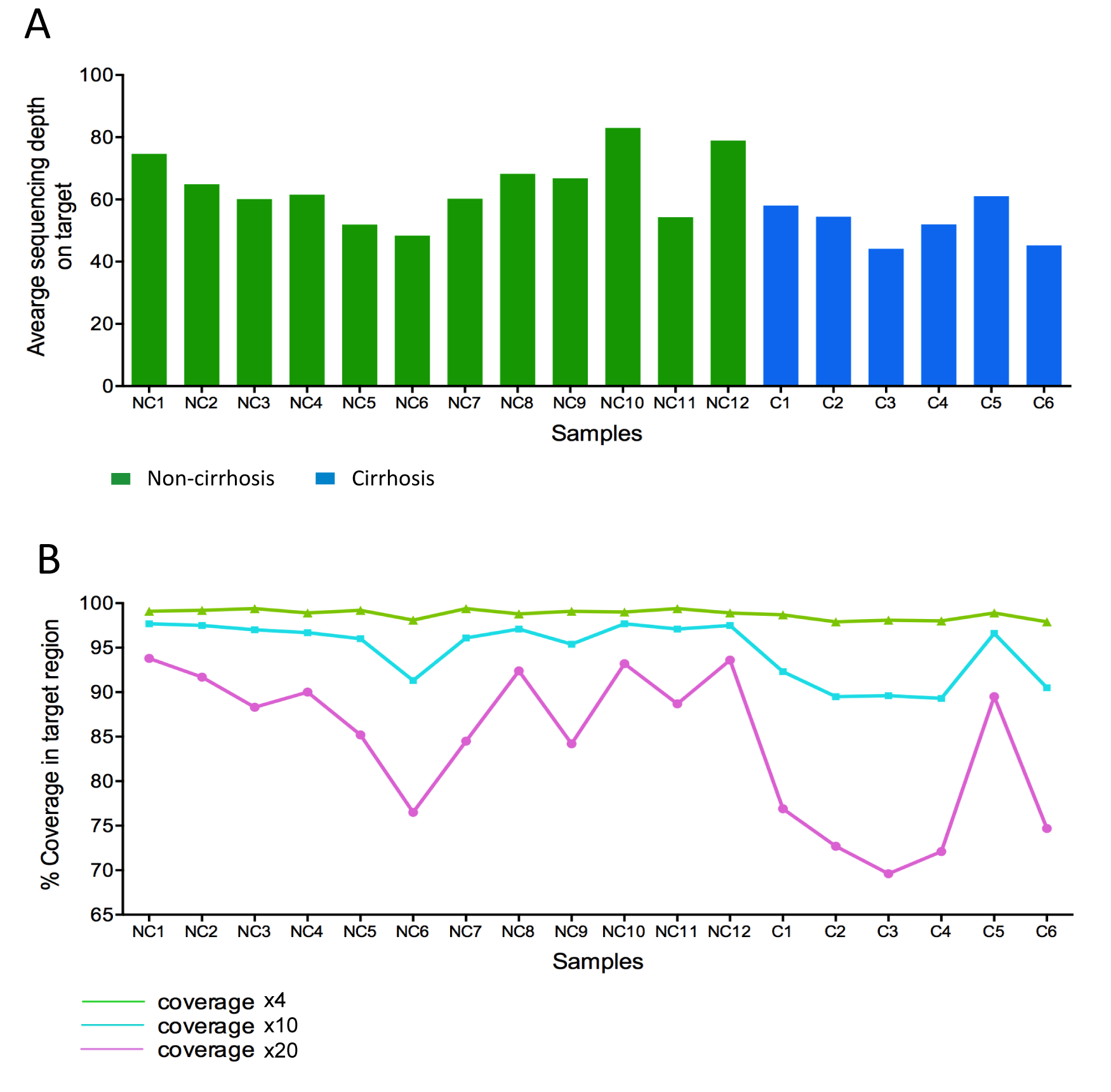

Supplement: S1 Fig — (A) Mean depth of reads for each sample, (B) Fraction of target covered in caption region (4-fold, 10-fold and 20-fold coverage) per exome. (TIFF) [file pone.0162586.s001.tiff]

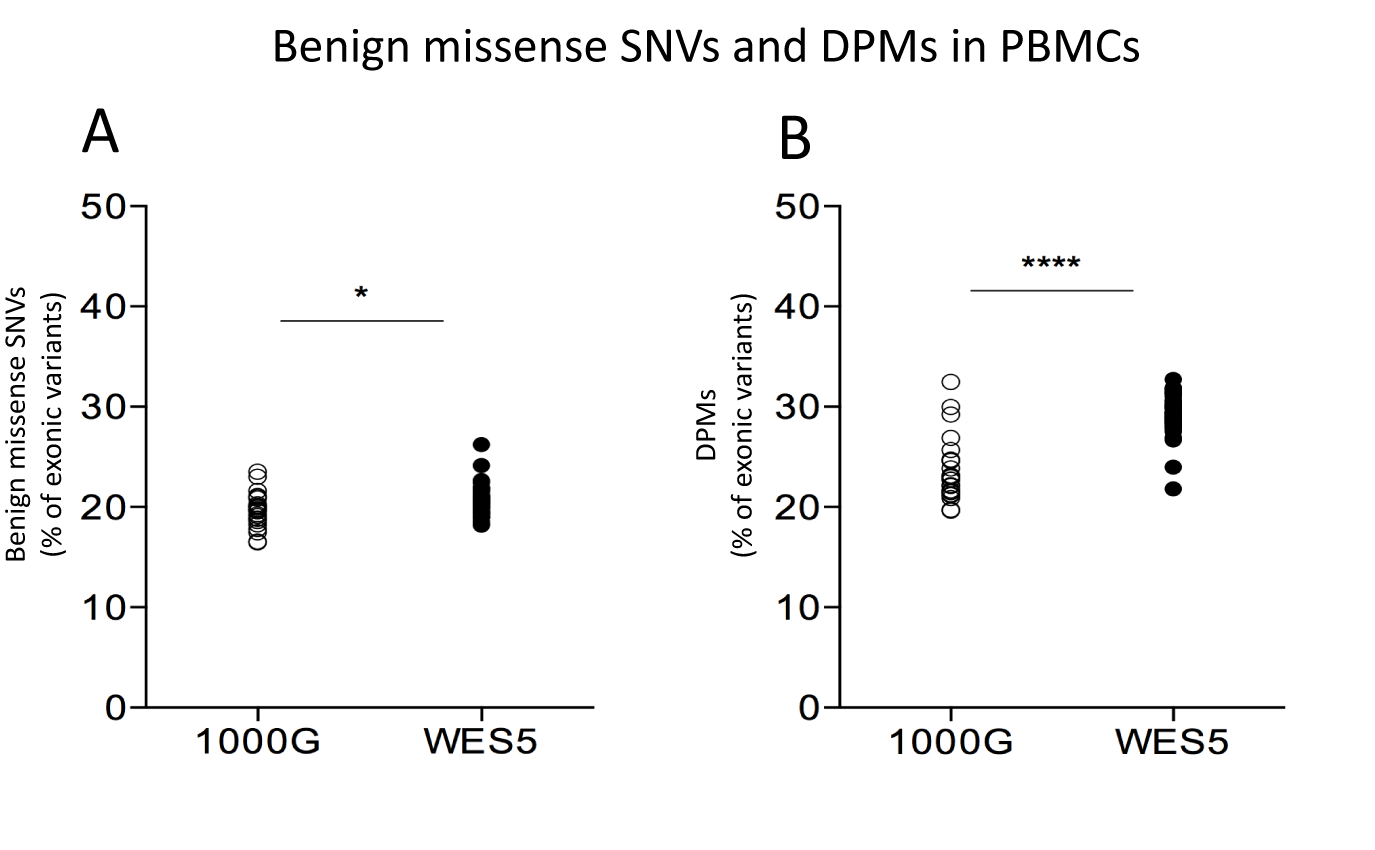

Supplement: S2 Fig — To determine if circulating leukocytes could be used as a control to account for germline mutations in individuals, we compared DNA from peripheral blood mononuclear cells (PBMCs) in 1000G and patients with hepatitis B virus (HBV) infection (WES 5) [32]. We found significantly more benign mutations (A) and DPMs (B) in HBV-exposed patients compared to healthy people from the 1000G dataset (*p<0.05, ****p<0.0001, Mann-Whitney test), suggesting the DNA genome of PBMCs are altered as a result of HBV infection. This may be due many factors dependent on HBV-associated inflammation, including: DNA mutations introduced during high levels of PBMC mitosis; or DPMs being accumulating as a result of clonal expansion of PBMCs. Greater immune activation associated with HBV infection would be expected to increase clonal expansion, and therefore DPMs according to our model. Crucially, this result suggests that liver disease causes changes in the DNA within the blood (not just the liver) and so using PBMC-derived DNA sequences to exclude germline variants would introduce bias. This therefore justifies our approach of using only the 1000 Genomes Project database to exclude probable germline mutations. (TIFF) [file pone.0162586.s002.tiff]

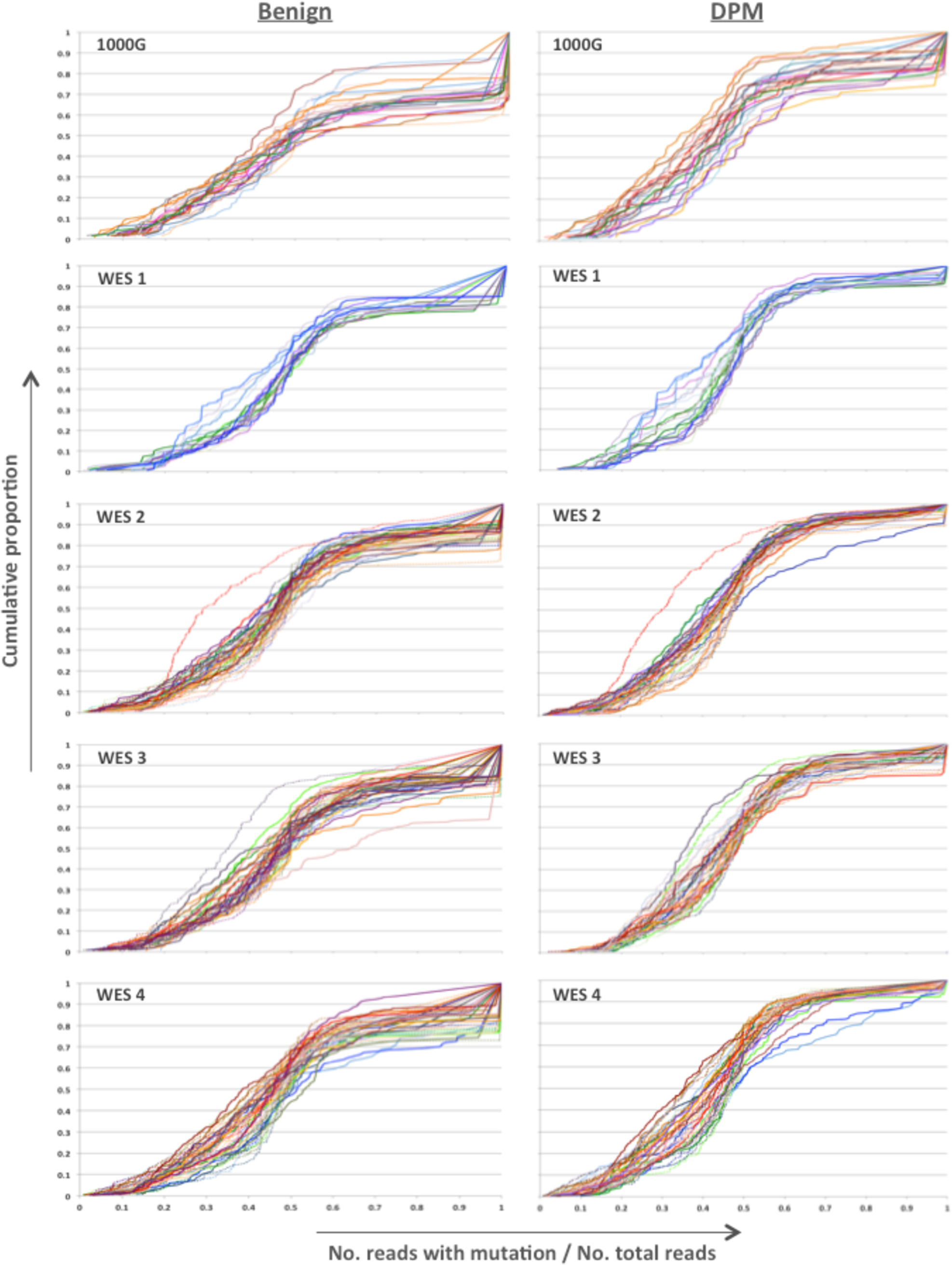

Supplement: S3 Fig — The allelic frequency of each benign missense variant (left) and DPMs (right) was estimated by the number of reads containing the variant divided by the number of the total reads at that particular base (x-axis). This was expressed as a cumulative plot with each patient as different colours for all benign missense variants and DPMs for 1000G and WES 1–4 (top, middle and bottom respectively). For WES 2–4, paired tumour (solid line) and non-tumour (dashed line) for the same patient are coloured the same colour. (TIFF) [file pone.0162586.s003.tiff]

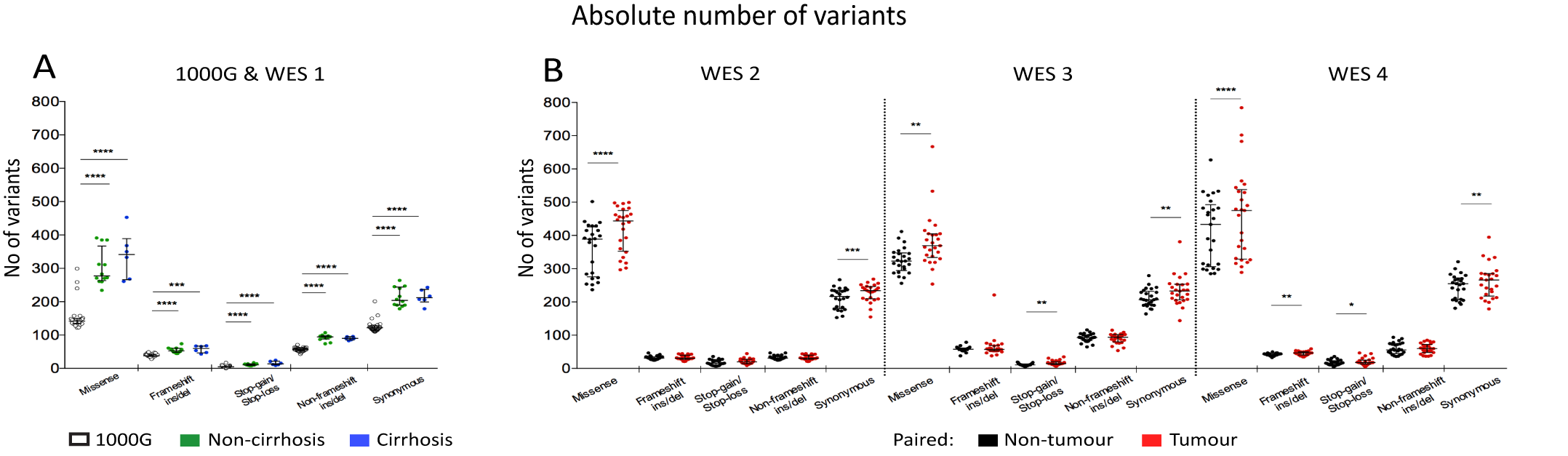

Supplement: S4 Fig — Non-synonymous mutations for all datasets were subdivided into 4 groups: missense, non-frameshift ins/del, frameshift ins/del and stop-gain/-loss. Samples in 1000G and WES 1 are unpaired, while samples in WES 2–4 paired. After excluding probable germline mutations, absolute numbers of variants (A and B) are shown for each sample. * p<0.05, ** p<0.01, *** p<0.001 and **** p<0.0001, Mann-Whitney U test (1000G and WES 1) or Wilcoxon matched-pairs signed-rank test (WES 2–4). (TIFF) [file pone.0162586.s004.tiff]

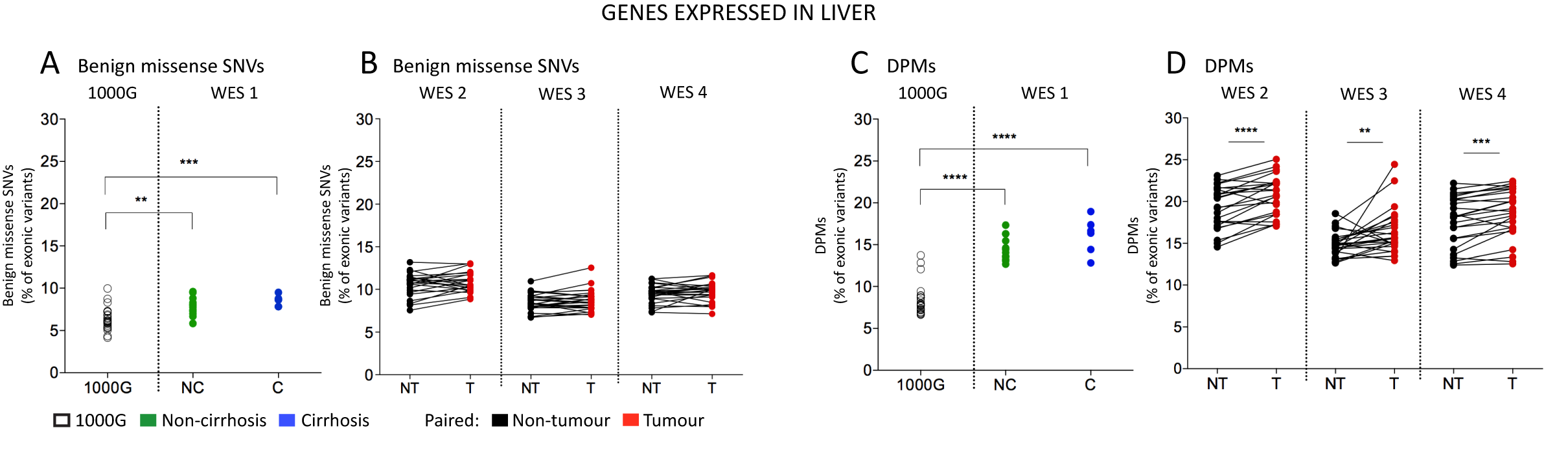

Supplement: S5 Fig — We analysed benign missense variants (A and B) and DPMs (C and D) in genes expressed in non-diseased liver tissue (measured by microarray analysis). The significant increase in DPMs in tumour tissue compared to paired non-tumour tissue was maintained (** p<0.01, *** p<0.001 and **** p<0.0001, Wilcoxon matched-pairs signed-rank test). No significant differences in benign missense variants or DPMs were detected between non-cirrhotic and cirrhotic patients (p>0.05, Mann-Whitney U test). (TIFF) [file pone.0162586.s005.tiff]

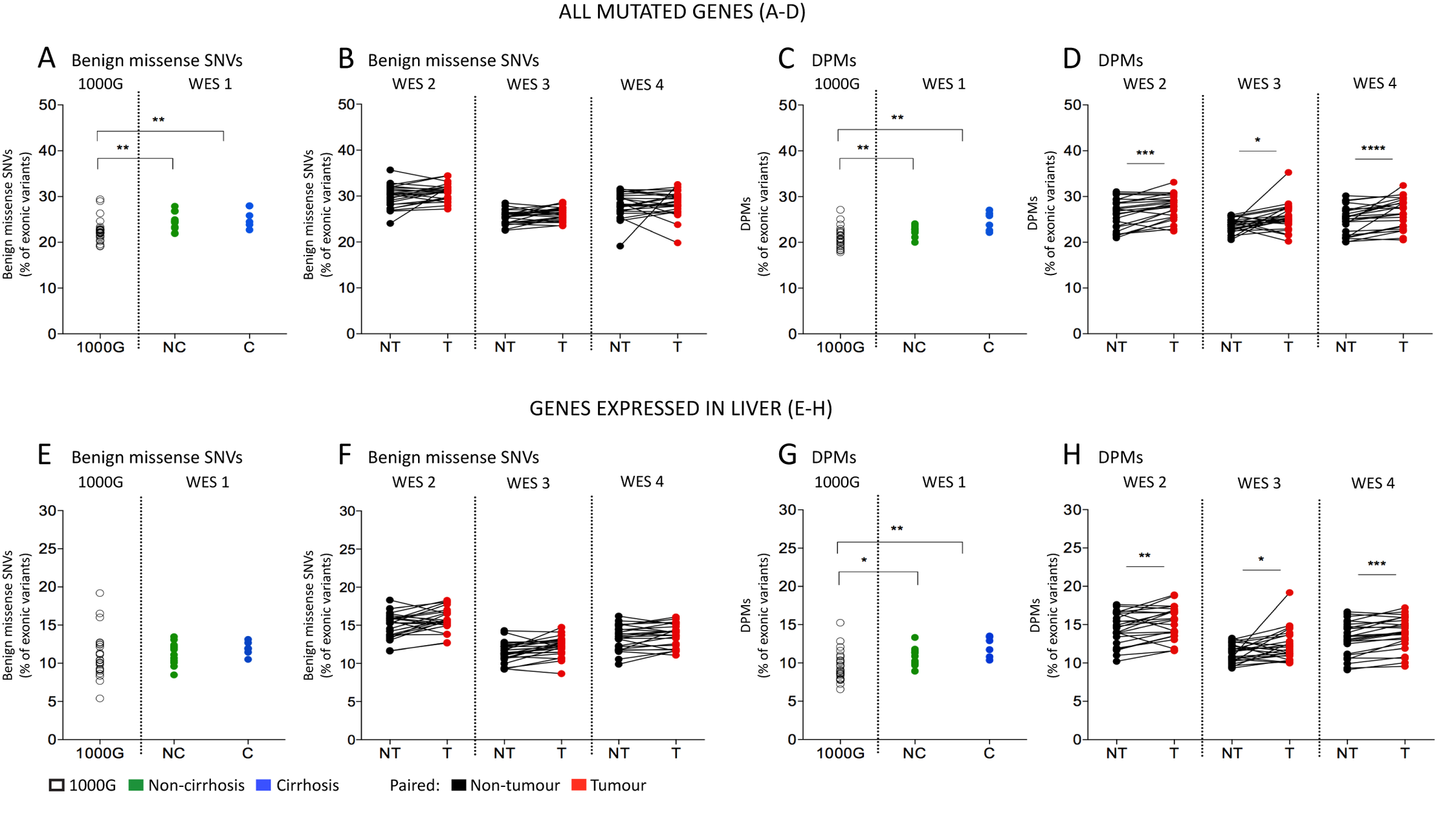

Supplement: S6 Fig — We compared total benign missense variants (A and B) and DPMs (C and D) in the datasets 1000G and WES 1–4. 1000G and WES 1 are unpaired and WES 2–4 paired. Significantly more DPMs (but not benign missense SNVs) were detected in tumour compared to paired non-tumour tissue (* p<0.05, ** p<0.01, *** p<0.001 and **** p<0.0001, Wilcoxon matched-pairs signed-rank test). We also analysed benign missense variants (E and F) and DPMs (G and H) in genes expressed in non-diseased liver tissue. The significant increase in tumour tissue was maintained. No significant differences in benign missense variants or DPMs were detected between non-cirrhotic and cirrhotic patients (p>0.05, Mann-Whitney U test). All variants were normalised to the total exonic variants after exclusion of probable germline mutations. Lines show linkages between matched paired non-tumour and tumour tissues samples. (TIFF) [file pone.0162586.s006.tiff]

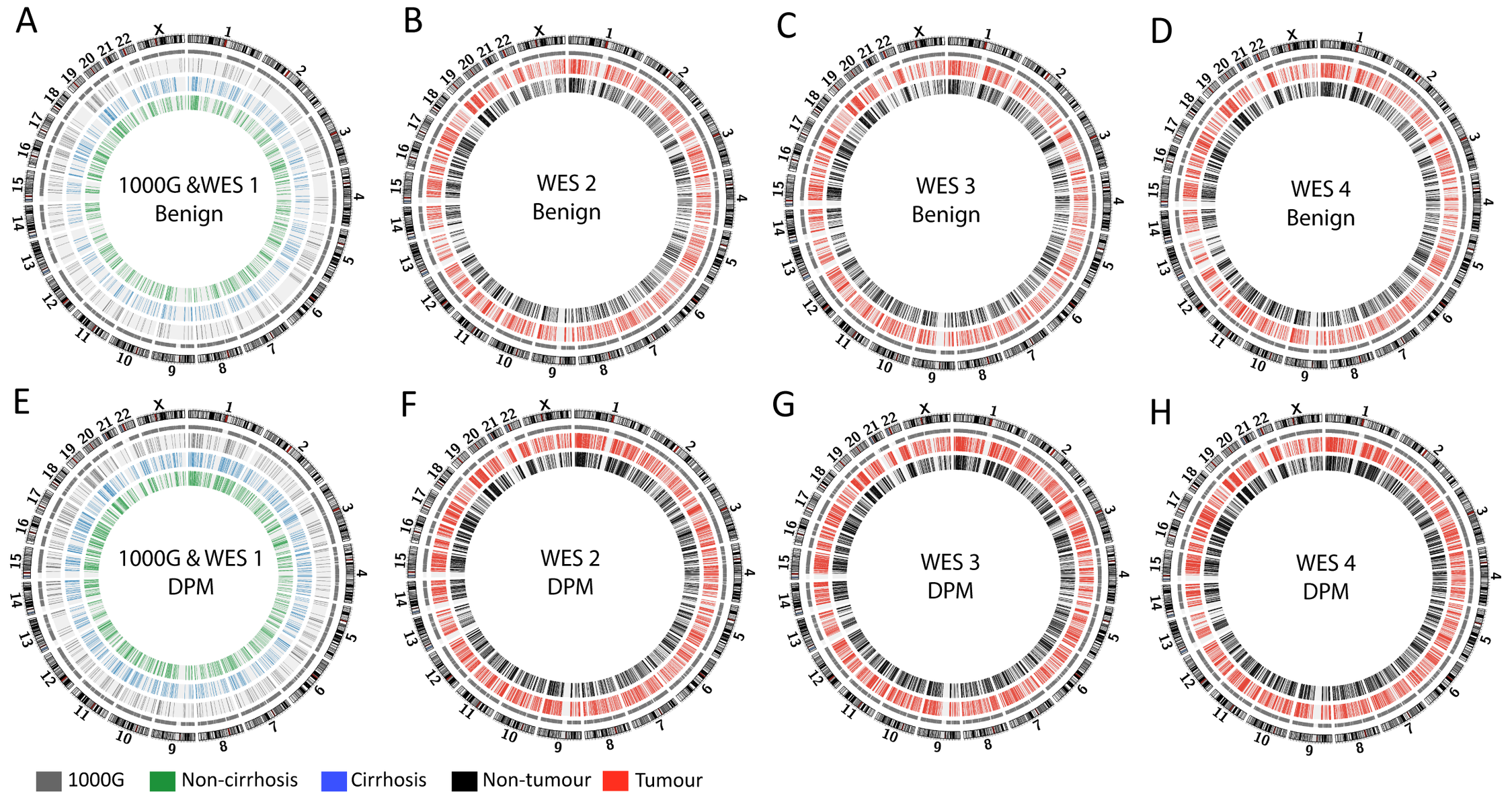

Supplement: S7 Fig — The genomic distribution of benign missense mutations (radial lines) are shown in Circos plot for (A) 1000G (grey) and WES 1 for non-cirrhotic (green) and cirrhotic (blue) patients, and for WES 2 (B), WES 3 (C), and WES 4 (D) for non-tumour (black) and tumour (red) tissues. The distribution of DPMs is also shown for 1000G and WES 1 (E), WES 2 (F), WES 3 (G) and WES 4 (H). The outer grey circle represents the exons location (USCS). Even distribution throughout the genome was observed given the exon distribution and the coverage of the reference sequence hg19. (TIFF) [file pone.0162586.s007.tiff]
