# Supplementary material for: Accumulation of Deleterious Passenger Mutations Is Associated with the Progression of Hepatocellular Carcinoma
Source: PLoS One. 2016 Sep 15;11(9):e0162586. doi: 10.1371/journal.pone.0162586 (PMC5025244; doi:10.1371/journal.pone.0162586)
Supplement: S1 Supplementary Methods — (PDF) [file pone.0162586.s008.pdf]

Supplementary Methods

WES datasets

WES 1. 90/100bp paired-end reads were sequenced on HiSeq2000 platform to an average depth of 50x, with ~86.75% target region covered by ≥20x, ~96.5% covered by ≥10x and ~98.90 covered by ≥4x (S1 Fig).

WES 2-4. HCC datasets were obtained from three sources: (1) WES 2 from *Institut National de la Santé et de la Recherche Médicale* (INSERM), (2) WES 3 from the database of Genotypes and Phenotypes (dbGAP) found at http://www.ncbi.nlm.nih.gov/gap through dbGAP accession number phs000627.v1.p1, and (3) WES 4 deposited at the EGA under accession number EGAS00001000824.

1000G. As controls for germ-line mutations, we analysed WES raw data from Phase 3 1000 Genomes Project [1] generated by different sequencing centres, including various ethnic groups and 15/10 (male/female) gender ratio (summarised in S1 Table).

WES 5. We analysed WES serum data (WES 5) from 25 individuals with chronic hepatitis B (CHB) and 25 healthy individuals from the same geographical location (Han Chinese cohort from Guangdong Province, China) [2].

Bioinformatics analysis pipeline

All FASTQ files were aligned to GRCh37/hg19 human reference genome (UCSC Genome Browser) with Bowtie2 (v.2.2.3) [3]. The PCR duplicates detected in BAM files were marked and removed using Picard tools (http://broadinstitute.github.io/picard/). The Genome Analysis Toolkit (GATK) [4, 5] was used for base quality recalibration and local realignment around indels (insertion/deletion) to correct mapping-related artefacts. The HaplotypeCaller in the GATK was used for calling single nucleotide variants (SNVs) and small indels. The minimum Phred-scaled confidence threshold at which variants were called was set at 30. Variant filtering was done according to recommended GATK best practices workflows for exome variant analysis (www.broadinstitute.org/) [6]. Large genomic rearrangements or copy number variations were not called, as WES datasets are not sensitive

to these changes due to the non-uniform read depth in captured regions and restricted coverage of the targets specific for the exome-enrichment kits used.

To compare different datasets with various regions of coverage, we analysed mutations that occurred in only protein-coding genes extracted from the UCSC genome annotation database (genome.ucsc.edu) for the GRCh37/hg19 assembly of the human genome.

Generation of a list of liver-expressed genes

Snap frozen liver biopsies of donor liver extraneous to patient need were taken from patients undergoing liver transplant from the RPAH. RNA was isolated using an RNAqueous® micro kit (Life Technologies, Carlsbad, CA, USA), loaded onto a Whole-Genome Expression Sentrix BeadChip array (v2.0, Illumina Inc., San Diego, CA, USA), and then read on a BeadArray reader (Illumina), according to the manufacturers' protocols. The raw data was analysed and transformed using GenomeStudio (version 1.1.1) and the LIMMA package (v.3.4.5) in R (v.3.0.1). Genes that were lower or equal to background fluorescence in all samples (detection p-value threshold of p=0.05) were excluded.

References

1. Abecasis GR, Auton A, Brooks LD, DePristo MA, Durbin RM, Handsaker RE, Kang HM, Marth GT, McVean GA: An integrated map of genetic variation from 1,092 human genomes. *Nature* 2012, 491:56-65.
2. Peng L, Zhao Q, Li Q, Li M, Li C, Xu T, Jing X, Zhu X, Wang Y, Li F, et al: The p.Ser267Phe variant in SLC10A1 is associated with resistance to chronic hepatitis B. *Hepatology* 2015, 61:1251-1260.
3. Langmead B, Salzberg SL: Fast gapped-read alignment with Bowtie 2. *Nat Methods* 2012, 9:357-359.
4. McKenna A, Hanna M, Banks E, Sivachenko A, Cibulskis K, Kernytsky A, Garimella K, Altshuler D, Gabriel S, Daly M, DePristo MA: The Genome Analysis Toolkit: a MapReduce framework for analyzing next-generation DNA sequencing data. *Genome Res* 2010, 20:1297-1303.
5. DePristo MA, Banks E, Poplin R, Garimella KV, Maguire JR, Hartl C, Philippakis AA, del Angel G, Rivas MA, Hanna M, et al: A framework for variation discovery and genotyping using next-generation DNA sequencing data. *Nat Genet* 2011, 43:491-498.
6. Van der Auwera GA, Carneiro MO, Hartl C, Poplin R, Del Angel G, Levy-Moonshine A, Jordan T, Shakir K, Roazen D, Thibault J, et al: From FastQ data to high confidence variant calls: the Genome Analysis Toolkit best practices pipeline. *Curr Protoc Bioinformatics* 2013, 11:11 10 11-11 10 33.
