# Supplementary material for: Accumulation of Deleterious Passenger Mutations Is Associated with the Progression of Hepatocellular Carcinoma
Source: PLoS One. 2016 Sep 15;11(9):e0162586. doi: 10.1371/journal.pone.0162586 (PMC5025244; doi:10.1371/journal.pone.0162586)
Supplement: S1 Table — (DOCX) [file pone.0162586.s009.docx]

| **Table S1. 1000 Genome samples summary.** | | | |  |  |  |  |  |
| --- | --- | --- | --- | --- | --- | --- | --- | --- |
|  |  |  |  |  |  |  |  |  |
| **Sample** | **Sample ID** | **Population** | **Population description** | **Gender** | **Platform** | **Center** | **Total**  **sequence** | **Average**  **depth on**  **exome*** |
| NA18527 | SRR718069 | CHB | Han Chinese in Bejing, China | female | Illumina HiSeq2000 | BCM | 57470692 | 9.61 |
| NA18618 | SRR715905 | CHB | Han Chinese in Bejing, China | female | Illumina HiSeq2000 | BCM | 68194578 | 10.85 |
| NA18645 | SRR716436 | CHB | Han Chinese in Bejing, China | male | Illumina HiSeq2000 | BCM | 48599766 | 7.97 |
| NA18748 | SRR702077 | CHB | Han Chinese in Bejing, China | male | Illumina HiSeq2000 | BCM | 70473818 | 9.71 |
| NA07056 | SRR764718 | CEU | Utah residents with Northern and Western European ancestry | female | Illumina HiSeq2000 | BCM | 44347290 | 6.91 |
| NA12750 | SRR794547 | CEU | Utah residents with Northern and Western European ancestry | male | Illumina HiSeq2000 | WUGSC | 44025078 | 5.62 |
| HG00097 | SRR765989 | GBR | British in England and Scotland | female | Illumina HiSeq2000 | BCM | 57715188 | 9.14 |
| HG00105 | SRR711354 | GBR | British in England and Scotland | male | Illumina HiSeq2000 | BCM | 57862372 | 8.62 |
| HG00112 | SRR702072 | GBR | British in England and Scotland | male | Illumina HiSeq2000 | BCM | 62381256 | 9.54 |
| HG00132 | SRR768526 | GBR | British in England and Scotland | female | Illumina HiSeq2000 | BCM | 65449116 | 11.59 |
| HG00173 | SRR764756 | FIN | Finnish in Finland | female | Illumina HiSeq2000 | BCM | 70332632 | 11.74 |
| HG00179 | SRR764731 | FIN | Finnish in Finland | female | Illumina HiSeq2000 | WUGSC | 83853818 | 11.74 |
| HG01500 | SRR764709 | IBS | Iberian populations in Spain | male | Illumina HiSeq2000 | BCM | 48276918 | 7.36 |
| HG01503 | SRR709962 | IBS | Iberian populations in Spain | male | Illumina HiSeq2000 | BCM | 53448700 | 7.71 |
| NA20538 | SRR766017 | TSI | Toscani in Italy | male | Illumina HiSeq2000 | BCM | 94926268 | 15.07 |
| NA20525 | SRR748570 | TSI | Toscani in Italy | male | Illumina HiSeq2000 | BCM | 53487232 | 8.37 |
| NA18977 | SRR718080 | JPT | Japanese in Tokyo, Japan | male | Illumina HiSeq2000 | BCM | 58771484 | 9.44 |
| NA18992 | SRR716428 | JPT | Japanese in Tokyo, Japan | female | Illumina HiSeq2000 | BCM | 56059440 | 9.15 |
| NA18501 | SRR100022 | YRI | Yoruba in Ibadan, Nigeria | male | Illumina HiSeq2000 | BI | 156192234 | 17.22 |
| NA18516 | SRR100026 | YRI | Yoruba in Ibadan, Nigeria | male | Illumina HiSeq2000 | BI | 149726904 | 16.45 |
| NA19466 | SRR748563 | LWK | Luhya in Webuye, Kenya | male | Illumina HiSeq2000 | BCM | 62799128 | 9.78 |
| HG01344 | SRR701482 | CLM | Colombian in Medellin, Colombia | male | Illumina HiSeq2000 | BCM | 70228076 | 10.88 |
| HG01357 | SRR710109 | CLM | Colombian in Medellin, Colombia | female | Illumina HiSeq2000 | BCM | 57799900 | 9.00 |
| HG02017 | SRR711366 | KHV | Kinh in Ho Chi Minh City, Vietnam | male | Illumina HiSeq2000 | BCM | 95003508 | 14.57 |
| HG02075 | SRR715105 | KHV | Kinh in Ho Chi Minh City, Vietnam | female | Illumina HiSeq2000 | BCM | 81483476 | 12.59 |
|  |  |  |  |  |  |  |  |  |
| *UCSC annotation | |  | WUGSC-Washington University, Genome Sequencing Center |  |  |  |  |  |
| BI-Broad Intitute | |  | BCM-Human Genome Sequencing Center of the Baylor College of Medicine | |  |  |  |  |
