# Supplementary material for: Accumulation of Deleterious Passenger Mutations Is Associated with the Progression of Hepatocellular Carcinoma
Source: PLoS One. 2016 Sep 15;11(9):e0162586. doi: 10.1371/journal.pone.0162586 (PMC5025244; doi:10.1371/journal.pone.0162586)
Supplement: S2 Table — (DOCX) [file pone.0162586.s010.docx]

| **Table S2. List of genes expressed above background levels detected in liver tissue of donor patients.** | |
| --- | --- |
|  |  |
| **Gene name** | **Average normalised expression** |
| A1CF | 10.363 |
| A2M | 14.16999138 |
| A2ML1 | 5.614888637 |
| A3GALT2 | 5.80611463 |
| A4GALT | 6.494241456 |
| A4GNT | 6.056602235 |
| AAA1 | 5.856794127 |
| AAAS | 6.096746033 |
| AACS | 7.377215349 |
| AACSL | 6.305048593 |
| AADAC | 12.43757161 |
| AADACL1 | 7.973417203 |
| AADACL4 | 5.969385241 |
| AADAT | 7.378 |
| AAMP | 9.283103235 |
| AARS | 12.80312511 |
| AARSD1 | 9.583693273 |
| AARSL | 7.382849158 |
| AASDH | 7.856434083 |
| AASDHPPT | 8.006217611 |
| AASS | 7.670428965 |
| AATF | 8.801260526 |
| AATK | 5.621456364 |
| ABAT | 8.778 |
| ABC1 | 6.584048908 |
| ABCA1 | 13.09664916 |
| ABCA12 | 7.128 |
| ABCA2 | 5.591 |
| ABCA3 | 6.669696659 |
| ABCA5 | 8.062 |
| ABCA6 | 9.256 |
| ABCA7 | 5.571426304 |
| ABCA8 | 9.520681509 |
| ABCA9 | 6.384 |
| ABCB1 | 10.86085986 |
| ABCB10 | 9.71615058 |
| ABCB11 | 7.770576152 |
| ABCB4 | 10.49811533 |
| ABCB6 | 9.161684739 |
| ABCB7 | 10.21076259 |
| ABCB8 | 6.04777306 |
| ABCC10 | 6.163404918 |
| ABCC11 | 6.452 |
| ABCC13 | 5.759060727 |
| ABCC2 | 9.473260848 |
| ABCC3 | 9.114 |
| ABCC4 | 6.748628682 |
| ABCC5 | 6.288 |
| ABCC6 | 7.357 |
| ABCC9 | 7.068 |
| ABCD1 | 5.555313907 |
| ABCD3 | 9.789046989 |
| ABCD4 | 5.817 |
| ABCE1 | 10.01957865 |
| ABCF1 | 11.01914056 |
| ABCF2 | 6.646 |
| ABCF3 | 6.029217543 |
| ABCG1 | 7.737 |
| ABCG2 | 5.613131123 |
| ABCG5 | 6.029924693 |
| ABCG8 | 9.750340752 |
| ABHD1 | 5.728188346 |
| ABHD10 | 9.097493329 |
| ABHD11 | 6.267712696 |
| ABHD13 | 5.686169969 |
| ABHD14A | 9.44277489 |
| ABHD14B | 9.255337586 |
| ABHD2 | 8.632621194 |
| ABHD3 | 9.400776868 |
| ABHD4 | 5.864338754 |
| ABHD5 | 8.449269061 |
| ABHD6 | 10.21974006 |
| ABHD7 | 5.921439205 |
| ABHD8 | 9.944059104 |
| ABI1 | 7.291 |
| ABI2 | 6.587 |
| ABI3 | 8.003295471 |
| ABI3BP | 6.266697917 |
| ABL1 | 6.715 |
| ABLIM1 | 9.908041412 |
| ABLIM2 | 5.7170939 |
| ABLIM3 | 9.316270656 |
| ABP1 | 6.204783992 |
| ABR | 6.891 |
| ABT1 | 5.415962308 |
| ABTB1 | 7.663628102 |
| ABTB2 | 5.711664908 |
| ACAA1 | 12.17715584 |
| ACAA2 | 13.10482501 |
| ACACA | 7.723 |
| ACACB | 11.59598789 |
| ACAD10 | 7.773640646 |
| ACAD11 | 11.616365 |
| ACAD8 | 7.207177758 |
| ACAD9 | 10.56677736 |
| ACADL | 7.643248233 |
| ACADM | 11.42546886 |
| ACADS | 9.625082469 |
| ACADSB | 8.728004218 |
| ACADVL | 12.56324866 |
| ACAT1 | 14.64354534 |
| ACAT2 | 12.71688886 |
| ACBD3 | 10.84537896 |
| ACBD4 | 6.799803135 |
| ACBD5 | 6.074888735 |
| ACBD6 | 7.739869373 |
| ACCN3 | 8.837956303 |
| ACCN4 | 8.793778334 |
| ACD | 6.499895132 |
| ACE2 | 6.377369649 |
| ACIN1 | 7.745050346 |
| ACLY | 10.13110206 |
| ACMSD | 12.25302699 |
| ACN9 | 8.954851516 |
| ACO1 | 12.98296595 |
| ACO2 | 8.409363057 |
| ACOT1 | 10.46040803 |
| ACOT12 | 9.855433576 |
| ACOT2 | 9.774272911 |
| ACOT4 | 10.85828668 |
| ACOT7 | 7.06 |
| ACOT8 | 7.626280509 |
| ACOT9 | 9.451284448 |
| ACOX1 | 8.121 |
| ACOX2 | 12.79332596 |
| ACOX3 | 9.205814177 |
| ACP1 | 10.80671652 |
| ACP2 | 10.17721454 |
| ACP5 | 8.270142328 |
| ACP6 | 9.099024953 |
| ACPL2 | 5.973422644 |
| ACSF3 | 5.996725268 |
| ACSL1 | 12.87637578 |
| ACSL3 | 7.837 |
| ACSL4 | 9.379179223 |
| ACSL5 | 8.25 |
| ACSM2 | 11.454 |
| ACSM3 | 9.022 |
| ACSM5 | 10.85533698 |
| ACSS1 | 7.476329066 |
| ACSS2 | 8.379 |
| ACSS3 | 6.559607561 |
| ACTA2 | 12.00667982 |
| ACTB | 13.749 |
| ACTG1 | 11.64384208 |
| ACTG2 | 8.398122514 |
| ACTL6A | 10.79107719 |
| ACTN1 | 12.62455145 |
| ACTN4 | 9.437846442 |
| ACTR10 | 10.07402969 |
| ACTR1A | 10.0729801 |
| ACTR1B | 9.932323589 |
| ACTR2 | 11.80877061 |
| ACTR3 | 10.48637851 |
| ACTR3B | 5.424925969 |
| ACTR5 | 7.475804296 |
| ACTR6 | 10.14206971 |
| ACTR8 | 5.861660903 |
| ACTRT1 | 6.898658618 |
| ACVR1 | 10.44604722 |
| ACVR1B | 7.05 |
| ACVR1C | 5.795366979 |
| ACVR2A | 5.656291016 |
| ACVR2B | 6.7041987 |
| ACVRL1 | 7.224035983 |
| ACY1 | 12.46164404 |
| ACY3 | 6.974401789 |
| ACYP1 | 6.572 |
| ACYP2 | 6.53774687 |
| ADA | 7.890006245 |
| ADAL | 6.308848293 |
| ADAM10 | 7.322808273 |
| ADAM15 | 8.649 |
| ADAM17 | 5.850586578 |
| ADAM18 | 8.739503846 |
| ADAM19 | 9.06492607 |
| ADAM2 | 5.794096368 |
| ADAM23 | 7.678749056 |
| ADAM28 | 5.671822545 |
| ADAM3A | 5.917201081 |
| ADAM7 | 5.995341137 |
| ADAM9 | 6.306274843 |
| ADAMTS1 | 10.47018214 |
| ADAMTS13 | 5.750974273 |
| ADAMTS15 | 5.843079718 |
| ADAMTS19 | 5.698807604 |
| ADAMTS2 | 5.593954335 |
| ADAMTS4 | 9.754272309 |
| ADAMTS5 | 5.569178807 |
| ADAMTS8 | 5.940291577 |
| ADAMTS9 | 6.942421139 |
| ADAMTSL2 | 9.127961429 |
| ADAMTSL3 | 7.998147731 |
| ADAR | 11.42796313 |
| ADARB1 | 6.99 |
| ADAT1 | 7.903056985 |
| ADAT3 | 5.795021604 |
| ADCK1 | 6.079981181 |
| ADCK2 | 9.387831957 |
| ADCK4 | 7.544232397 |
| ADCK5 | 5.682421543 |
| ADCY1 | 6.206940309 |
| ADCY3 | 9.161714442 |
| ADCY4 | 7.521203336 |
| ADCY6 | 7.169711509 |
| ADCY7 | 7.726700904 |
| ADCY9 | 8.305452619 |
| ADD1 | 11.82145613 |
| ADD3 | 8.746 |
| ADFP | 12.87905366 |
| ADH1A | 14.93408991 |
| ADH1B | 11.8065078 |
| ADH1C | 13.19503081 |
| ADH4 | 13.25858028 |
| ADH5 | 7.774191749 |
| ADH6 | 10.52563292 |
| ADHFE1 | 8.741777657 |
| ADI1 | 13.93305566 |
| ADIPOR1 | 10.19595632 |
| ADIPOR2 | 12.40038001 |
| ADK | 9.091 |
| ADM | 12.01006974 |
| ADM2 | 8.745550458 |
| ADNP | 8.666522694 |
| ADNP2 | 9.442852967 |
| ADO | 9.047477246 |
| ADORA1 | 5.843281469 |
| ADORA2A | 6.886316776 |
| ADORA2B | 5.833646562 |
| ADORA3 | 7.597 |
| ADPGK | 8.76657124 |
| ADPRH | 6.602980369 |
| ADPRHL1 | 5.912166192 |
| ADPRHL2 | 9.361975375 |
| ADRA1A | 7.571 |
| ADRA1B | 9.228734819 |
| ADRA1D | 6.259136956 |
| ADRA2A | 5.845496048 |
| ADRA2B | 6.68636417 |
| ADRB2 | 9.695433213 |
| ADRM1 | 11.47464634 |
| ADSL | 11.94391084 |
| ADSS | 7.383839819 |
| ADSSL1 | 7.143 |
| AEBP1 | 7.569291467 |
| AEBP2 | 7.83869645 |
| AES | 7.784 |
| AFAP | 5.852233852 |
| AFAP1L1 | 7.003736723 |
| AFAP1L2 | 6.104240279 |
| AFAR3 | 6.400536242 |
| AFF1 | 5.7728865 |
| AFF3 | 7.122334081 |
| AFF4 | 7.860574484 |
| AFG3L1 | 6.762112163 |
| AFG3L2 | 9.076079255 |
| AFM | 11.2052514 |
| AFMID | 10.40007594 |
| AFP | 8.576887197 |
| AFTPH | 8.581235028 |
| AGA | 8.759107234 |
| AGBL4 | 5.937347511 |
| AGER | 5.885020256 |
| AGGF1 | 6.154618419 |
| AGK | 8.60362362 |
| AGL | 7.283 |
| AGMAT | 11.10368313 |
| AGPAT1 | 6.185 |
| AGPAT2 | 9.804 |
| AGPAT3 | 9.085429998 |
| AGPAT5 | 11.01944134 |
| AGPAT6 | 5.900884674 |
| AGPAT9 | 9.038968627 |
| AGPS | 8.784317137 |
| AGRN | 7.926500455 |
| AGT | 13.63022737 |
| AGTPBP1 | 8.256701694 |
| AGTR1 | 11.59630369 |
| AGTR2 | 5.803509722 |
| AGTRAP | 6.928747877 |
| AGTRL1 | 9.286583031 |
| AGXT | 13.75982766 |
| AGXT2 | 10.98716425 |
| AGXT2L1 | 11.23748225 |
| AGXT2L2 | 7.11993364 |
| AHCTF1 | 8.25 |
| AHCY | 12.12444863 |
| AHCYL1 | 11.80032071 |
| AHCYL2 | 6.629440437 |
| AHDC1 | 5.49214452 |
| AHI1 | 6.218286489 |
| AHNAK | 9.968 |
| AHR | 12.72602814 |
| AHSA1 | 10.70608107 |
| AHSA2 | 6.272408554 |
| AHSG | 14.48427543 |
| AIF1 | 10.907 |
| AIFM1 | 8.006 |
| AIFM2 | 5.924889028 |
| AIG1 | 9.761859669 |
| AIM1 | 6.583359765 |
| AIM2 | 6.939319594 |
| AIP | 9.54935817 |
| AIPL1 | 5.969441562 |
| AK1 | 9.693959973 |
| AK2 | 10.876 |
| AK3 | 12.63732918 |
| AK3L1 | 8.263 |
| AK3L2 | 9.383635907 |
| AK7 | 6.172842411 |
| AKAP1 | 7.136651803 |
| AKAP10 | 5.774401584 |
| AKAP11 | 6.873 |
| AKAP12 | 6.636 |
| AKAP13 | 6.706 |
| AKAP3 | 5.844028518 |
| AKAP4 | 5.732208761 |
| AKAP7 | 9.35 |
| AKAP8 | 7.943145138 |
| AKAP8L | 5.962643921 |
| AKAP9 | 5.761059278 |
| AKIRIN1 | 9.867138117 |
| AKIRIN2 | 10.24577021 |
| AKNA | 6.899231337 |
| AKR1A1 | 10.097 |
| AKR1B1 | 9.020727621 |
| AKR1B10 | 9.057435823 |
| AKR1C2 | 5.490877682 |
| AKR1C3 | 14.40387797 |
| AKR1C4 | 13.33973699 |
| AKR1CL1 | 5.769649743 |
| AKR1CL2 | 6.055178698 |
| AKR1D1 | 9.903769671 |
| AKR7A2 | 12.22074624 |
| AKR7A3 | 10.66812836 |
| AKT1 | 7.052 |
| AKT1S1 | 8.721826607 |
| AKT2 | 5.502701863 |
| AKT3 | 5.757 |
| AKTIP | 9.128339006 |
| ALAD | 10.27958212 |
| ALAS1 | 7.242356298 |
| ALB | 15.22783822 |
| ALCAM | 10.96023434 |
| ALDH16A1 | 9.8626113 |
| ALDH1A1 | 13.52329932 |
| ALDH1A2 | 6.696415449 |
| ALDH1A3 | 6.561674566 |
| ALDH1B1 | 7.374450737 |
| ALDH1L1 | 11.44942068 |
| ALDH2 | 12.98615295 |
| ALDH3A1 | 6.835557845 |
| ALDH3A2 | 10.267 |
| ALDH3B1 | 6.242201052 |
| ALDH3B2 | 5.926597831 |
| ALDH4A1 | 11.439 |
| ALDH5A1 | 10.04058412 |
| ALDH6A1 | 12.69185658 |
| ALDH7A1 | 11.482 |
| ALDH8A1 | 9.324 |
| ALDH9A1 | 13.73691277 |
| ALDOA | 9.837 |
| ALDOB | 14.76552784 |
| ALDOC | 11.63537299 |
| ALG1 | 7.700681268 |
| ALG10 | 5.522666361 |
| ALG11 | 8.037533934 |
| ALG12 | 6.459634081 |
| ALG13 | 11.09309051 |
| ALG14 | 9.616726561 |
| ALG2 | 6.817 |
| ALG3 | 10.5787413 |
| ALG5 | 12.12018644 |
| ALG6 | 8.157752526 |
| ALG8 | 10.93935768 |
| ALG9 | 10.08015124 |
| ALKBH | 7.528335376 |
| ALKBH2 | 8.696558363 |
| ALKBH3 | 11.18454415 |
| ALKBH4 | 7.057947945 |
| ALKBH5 | 12.45203682 |
| ALKBH6 | 8.658484711 |
| ALKBH7 | 12.38663756 |
| ALKBH8 | 6.971553525 |
| ALLC | 5.538220137 |
| ALMS1 | 6.80055597 |
| ALOX15 | 5.878 |
| ALOX5 | 9.595755979 |
| ALOX5AP | 10.29739779 |
| ALPL | 12.25205057 |
| ALPP | 11.57028842 |
| ALS2 | 9.65298058 |
| ALS2CL | 5.866276597 |
| ALS2CR12 | 5.678470987 |
| ALS2CR13 | 8.514310927 |
| ALS2CR14 | 6.267404073 |
| ALS2CR19 | 5.706218352 |
| ALS2CR2 | 10.96634161 |
| ALS2CR4 | 8.20889311 |
| ALX3 | 6.275451608 |
| AMAC1 | 5.524781453 |
| AMACR | 7.832 |
| AMBP | 15.51454693 |
| AMBRA1 | 6.053864039 |
| AMD1 | 9.867 |
| AMDHD1 | 12.44857782 |
| AMDHD2 | 6.149800448 |
| AMFR | 8.709 |
| AMH | 5.676907052 |
| AMHR2 | 7.518389018 |
| AMICA1 | 8.648475824 |
| AMIGO1 | 5.924229144 |
| AMIGO2 | 5.985944515 |
| AMMECR1 | 8.4493042 |
| AMMECR1L | 7.820704763 |
| AMN | 5.689448543 |
| AMN1 | 5.691152271 |
| AMOT | 6.55521881 |
| AMT | 10.05575328 |
| AMY1A | 5.739166402 |
| AMY1B | 7.560680833 |
| AMY1C | 13.0169943 |
| AMZ2 | 10.38767843 |
| ANAPC1 | 8.926521371 |
| ANAPC10 | 9.427497017 |
| ANAPC11 | 12.24498611 |
| ANAPC13 | 12.09916321 |
| ANAPC2 | 7.042 |
| ANAPC4 | 9.29585849 |
| ANAPC5 | 11.19181501 |
| ANAPC7 | 6.349086863 |
| ANG | 15.31353454 |
| ANGEL1 | 7.28674012 |
| ANGEL2 | 8.331981995 |
| ANGPT1 | 5.737016521 |
| ANGPT2 | 6.230402664 |
| ANGPTL2 | 8.601308975 |
| ANGPTL3 | 14.30663815 |
| ANGPTL4 | 9.085 |
| ANGPTL6 | 8.54832648 |
| ANK2 | 6.241335099 |
| ANK3 | 7.36 |
| ANKDD1A | 6.617361448 |
| ANKFY1 | 9.564359845 |
| ANKH | 5.6958025 |
| ANKHD1 | 8.338388138 |
| ANKHD1-EIF4EBP3 | 5.654706503 |
| ANKMY1 | 6.314 |
| ANKMY2 | 8.371127059 |
| ANKRA2 | 8.507597414 |
| ANKRD10 | 7.708662277 |
| ANKRD12 | 8.434286538 |
| ANKRD13A | 9.665527942 |
| ANKRD13C | 6.00413123 |
| ANKRD16 | 6.883 |
| ANKRD17 | 8.586845832 |
| ANKRD20A3 | 5.374294943 |
| ANKRD22 | 6.648978439 |
| ANKRD24 | 6.492737298 |
| ANKRD27 | 5.885842885 |
| ANKRD28 | 5.716918596 |
| ANKRD30B | 11.48666496 |
| ANKRD32 | 5.970194532 |
| ANKRD33 | 11.18665178 |
| ANKRD35 | 8.220622702 |
| ANKRD37 | 8.6675442 |
| ANKRD38 | 7.062513006 |
| ANKRD39 | 8.807215646 |
| ANKRD40 | 8.647801048 |
| ANKRD43 | 6.189169918 |
| ANKRD46 | 10.23606215 |
| ANKRD47 | 7.003857527 |
| ANKRD49 | 8.829198117 |
| ANKRD5 | 8.542120329 |
| ANKRD50 | 7.411278057 |
| ANKRD54 | 7.919122137 |
| ANKRD7 | 5.637897466 |
| ANKS1A | 8.717005225 |
| ANKS1B | 5.674217096 |
| ANKS4B | 7.01363015 |
| ANKZF1 | 6.568705667 |
| ANLN | 6.053005593 |
| ANO6 | 10.4089965 |
| ANO8 | 5.348983941 |
| ANP32A | 7.892009829 |
| ANP32B | 13.05428319 |
| ANP32C | 6.917485592 |
| ANP32E | 5.711230741 |
| ANPEP | 10.65199229 |
| ANTXR1 | 7.02 |
| ANTXR2 | 10.04459345 |
| ANUBL1 | 5.678579659 |
| ANXA1 | 10.02444035 |
| ANXA10 | 12.18838108 |
| ANXA11 | 8.342086654 |
| ANXA13 | 6.081 |
| ANXA2 | 8.382 |
| ANXA2P1 | 8.048239597 |
| ANXA2P3 | 6.693112515 |
| ANXA3 | 6.651283273 |
| ANXA4 | 8.993789338 |
| ANXA5 | 11.97742668 |
| ANXA7 | 12.5050549 |
| ANXA9 | 7.413721448 |
| AOAH | 6.847775344 |
| AOC3 | 6.478876943 |
| AOF2 | 9.492317049 |
| AOX1 | 13.96678334 |
| AP1B1 | 9.908353616 |
| AP1G1 | 8.73036146 |
| AP1G2 | 6.273 |
| AP1GBP1 | 5.473649654 |
| AP1M1 | 11.0583967 |
| AP1S1 | 9.219 |
| AP1S2 | 9.941741328 |
| AP2A1 | 6.592 |
| AP2A2 | 6.268678065 |
| AP2B1 | 6.382633833 |
| AP2M1 | 9.198365571 |
| AP2S1 | 12.748 |
| AP3B1 | 10.5579656 |
| AP3D1 | 9.609043502 |
| AP3M1 | 9.037245044 |
| AP3M2 | 6.749740501 |
| AP3S1 | 10.48817937 |
| AP3S2 | 8.243695294 |
| AP4B1 | 8.884861161 |
| AP4E1 | 7.676441409 |
| APAF1 | 8.911376424 |
| APBA2 | 5.62859414 |
| APBA2BP | 6.1694029 |
| APBA3 | 9.049628161 |
| APBB1IP | 9.229343651 |
| APBB3 | 7.878622198 |
| APCS | 14.9605291 |
| APEH | 11.2392096 |
| APEX1 | 11.45723737 |
| APEX2 | 8.827472721 |
| APH1A | 10.85669805 |
| APH1B | 7.680747938 |
| API5 | 10.45495607 |
| APIP | 10.33351692 |
| APITD1 | 7.786 |
| APLN | 5.559919599 |
| APLP2 | 8.982577829 |
| APOA1 | 15.43046166 |
| APOA1BP | 10.27053058 |
| APOA2 | 15.54095851 |
| APOA4 | 10.41865098 |
| APOA5 | 10.35826452 |
| APOB | 14.46295654 |
| APOBEC3A | 5.735682535 |
| APOBEC3B | 5.491134266 |
| APOBEC3C | 5.515419532 |
| APOBEC3F | 6.755 |
| APOBEC3G | 6.166936767 |
| APOC1 | 14.79774049 |
| APOC2 | 13.8806242 |
| APOC3 | 15.17474103 |
| APOC4 | 13.0011684 |
| APOD | 6.135501639 |
| APOE | 14.25938531 |
| APOF | 13.30887686 |
| APOH | 14.69774273 |
| APOL1 | 6.87 |
| APOL2 | 5.752 |
| APOL3 | 9.302681566 |
| APOL6 | 5.921372272 |
| APOLD1 | 7.721646848 |
| APOM | 12.19324478 |
| APOO | 9.79344423 |
| APP | 8.522 |
| APPBP2 | 5.690912571 |
| APPL | 9.395361311 |
| APRIN | 7.890665548 |
| APRT | 10.64 |
| APTX | 8.645310033 |
| AQP1 | 7.43 |
| AQP10 | 5.966494939 |
| AQP11 | 10.13724778 |
| AQP12A | 12.67217425 |
| AQP3 | 9.881704047 |
| AQP7 | 6.511122681 |
| AQP7P1 | 6.809243478 |
| AQP7P2 | 6.486 |
| AQP9 | 13.14539494 |
| AQR | 7.962382925 |
| AR | 9.109816967 |
| ARAF | 8.049761316 |
| ARAP2 | 6.340826398 |
| ARCN1 | 11.50737784 |
| ARD1A | 9.783169709 |
| ARF1 | 12.70990236 |
| ARF3 | 9.214593155 |
| ARF4 | 11.882 |
| ARF5 | 10.60720083 |
| ARFGAP1 | 7.810893908 |
| ARFGAP2 | 9.120149052 |
| ARFGAP3 | 10.93933275 |
| ARFGEF1 | 8.973373229 |
| ARFGEF2 | 6.733565381 |
| ARFIP1 | 7.817 |
| ARFIP2 | 5.749607662 |
| ARG1 | 13.62401959 |
| ARG2 | 5.855892663 |
| ARG99 | 6.467798976 |
| ARGFX | 5.462273222 |
| ARHGAP1 | 10.83592555 |
| ARHGAP10 | 8.140575206 |
| ARHGAP12 | 8.314375322 |
| ARHGAP15 | 8.6242856 |
| ARHGAP17 | 9.850816701 |
| ARHGAP18 | 6.384964471 |
| ARHGAP19 | 7.535136337 |
| ARHGAP20 | 5.78176606 |
| ARHGAP21 | 10.61675469 |
| ARHGAP22 | 5.533162772 |
| ARHGAP23 | 8.037264409 |
| ARHGAP24 | 6.221 |
| ARHGAP25 | 6.692 |
| ARHGAP27 | 8.331267539 |
| ARHGAP29 | 5.704230356 |
| ARHGAP30 | 7.574810174 |
| ARHGAP4 | 7.255874541 |
| ARHGAP5 | 6.155788649 |
| ARHGAP9 | 7.876936379 |
| ARHGDIA | 9.994212724 |
| ARHGDIB | 13.42218163 |
| ARHGDIG | 6.076580447 |
| ARHGEF1 | 5.917270193 |
| ARHGEF10L | 6.244 |
| ARHGEF11 | 6.061704923 |
| ARHGEF12 | 8.096154878 |
| ARHGEF15 | 6.05251315 |
| ARHGEF16 | 7.483660559 |
| ARHGEF18 | 10.04085039 |
| ARHGEF19 | 6.51810142 |
| ARHGEF2 | 9.836667552 |
| ARHGEF3 | 9.235621833 |
| ARHGEF5 | 7.702546896 |
| ARHGEF6 | 8.661070324 |
| ARHGEF7 | 9.757101676 |
| ARHGEF9 | 6.873871849 |
| ARID1A | 9.667417288 |
| ARID3A | 7.795778497 |
| ARID3B | 5.944593145 |
| ARID4A | 6.476 |
| ARID4B | 5.565712969 |
| ARID5A | 5.734792871 |
| ARID5B | 9.329155045 |
| ARIH1 | 8.389009241 |
| ARIH2 | 10.33540278 |
| ARL1 | 10.82915161 |
| ARL13B | 6.437 |
| ARL14 | 6.502558311 |
| ARL15 | 5.76762132 |
| ARL16 | 8.515933675 |
| ARL2 | 10.25339405 |
| ARL2BP | 10.2446474 |
| ARL4 | 7.563 |
| ARL4C | 6.011198832 |
| ARL4D | 6.899083966 |
| ARL5 | 10.001 |
| ARL5B | 7.75977998 |
| ARL6 | 5.72757528 |
| ARL6IP1 | 12.36504049 |
| ARL6IP4 | 7.733 |
| ARL6IP5 | 11.48926784 |
| ARL6IP6 | 8.445414362 |
| ARL8B | 10.75376372 |
| ARMC1 | 9.903124666 |
| ARMC10 | 7.905684971 |
| ARMC4 | 5.481341031 |
| ARMC5 | 6.592125537 |
| ARMC6 | 8.975092988 |
| ARMC7 | 7.633069776 |
| ARMC8 | 6.688 |
| ARMC9 | 5.729494803 |
| ARMCX1 | 7.195971505 |
| ARMCX2 | 8.072081848 |
| ARMCX3 | 9.105673634 |
| ARMCX5 | 8.230150873 |
| ARMCX6 | 7.335 |
| ARMET | 14.31812801 |
| ARMETL1 | 7.473680606 |
| ARNT | 7.723510304 |
| ARNTL | 7.790105083 |
| ARNTL2 | 6.534434525 |
| ARPC1A | 11.70330235 |
| ARPC2 | 10.168 |
| ARPC3 | 11.94017359 |
| ARPC4 | 9.873610598 |
| ARPC5 | 12.55154292 |
| ARPC5L | 12.09503708 |
| ARPM1 | 7.498862332 |
| ARPP-19 | 7.880088381 |
| ARRB1 | 7.601216872 |
| ARRB2 | 5.795115274 |
| ARRDC1 | 6.537209802 |
| ARRDC2 | 6.221 |
| ARRDC3 | 7.097649024 |
| ARRDC4 | 7.313816182 |
| ARS2 | 8.255441155 |
| ARSA | 6.747038287 |
| ARSB | 6.147 |
| ARSD | 8.241 |
| ARSE | 8.96201719 |
| ARSF | 6.610178936 |
| ARSG | 6.51474094 |
| ARSK | 5.443116253 |
| ART4 | 8.473234443 |
| ART5 | 5.66756008 |
| ARTS-1 | 5.500524216 |
| ARV1 | 10.52469228 |
| ARVCF | 5.851404934 |
| ARX | 5.558177115 |
| AS3MT | 9.032490215 |
| ASAH1 | 7.788157221 |
| ASAH3 | 5.584980811 |
| ASAHL | 9.496545678 |
| ASAP1 | 8.994206207 |
| ASAP3 | 9.022672515 |
| ASB1 | 8.22458947 |
| ASB13 | 8.935845084 |
| ASB15 | 5.771451709 |
| ASB16 | 5.602327801 |
| ASB17 | 5.695469626 |
| ASB18 | 5.726617244 |
| ASB2 | 7.094833638 |
| ASB3 | 7.415 |
| ASB4 | 6.115161717 |
| ASB6 | 5.757864164 |
| ASB7 | 6.593 |
| ASB8 | 9.510365132 |
| ASB9 | 8.522407435 |
| ASCC1 | 10.21030221 |
| ASCC2 | 11.68239926 |
| ASCC3 | 8.803 |
| ASCC3L1 | 6.485774782 |
| ASCL2 | 6.639303148 |
| ASF1A | 8.779690788 |
| ASGR1 | 14.80448735 |
| ASGR2 | 11.053 |
| ASH1L | 6.174097342 |
| ASH2L | 10.13615316 |
| ASL | 7.916030225 |
| ASMTL | 7.833 |
| ASNA1 | 7.204826046 |
| ASNS | 8.645331704 |
| ASNSD1 | 10.9356432 |
| ASPA | 6.782556512 |
| ASPH | 5.828 |
| ASPHD1 | 5.693 |
| ASPM | 6.103143856 |
| ASPN | 9.571208727 |
| ASPRV1 | 5.591658858 |
| ASPSCR1 | 7.679 |
| ASRGL1 | 6.956184917 |
| ASS | 9.397 |
| ASTE1 | 7.616878667 |
| ASTL | 5.81543928 |
| ASTN2 | 5.527869015 |
| ASXL1 | 7.817103089 |
| ASXL2 | 9.198330399 |
| ATAD1 | 9.43157319 |
| ATAD2 | 7.603181787 |
| ATAD3A | 8.41045524 |
| ATAD4 | 7.069864485 |
| ATBF1 | 7.896380213 |
| ATE1 | 6.193005648 |
| ATF1 | 7.513577659 |
| ATF2 | 7.017434159 |
| ATF3 | 10.76177357 |
| ATF4 | 9.972526916 |
| ATF5 | 14.64607784 |
| ATF6 | 11.08417383 |
| ATF7 | 5.349923704 |
| ATF7IP | 10.16534577 |
| ATF7IP2 | 7.560795336 |
| ATG10 | 9.047606146 |
| ATG12 | 7.660322229 |
| ATG16L1 | 7.948817941 |
| ATG2A | 8.301536772 |
| ATG3 | 11.1139805 |
| ATG4A | 9.997929566 |
| ATG4C | 9.09735007 |
| ATG5 | 8.284607033 |
| ATG7 | 9.398115214 |
| ATG9A | 8.029732869 |
| ATHL1 | 6.25543704 |
| ATIC | 11.70201454 |
| ATL2 | 8.386764899 |
| ATL3 | 10.9657711 |
| ATM | 8.634557828 |
| ATMIN | 7.995133637 |
| ATN1 | 5.89699664 |
| ATOH8 | 7.762672953 |
| ATOX1 | 12.62435686 |
| ATP10A | 6.624289112 |
| ATP11B | 6.549733119 |
| ATP11C | 8.250245525 |
| ATP13A1 | 8.376526859 |
| ATP13A3 | 6.572730335 |
| ATP1A1 | 10.35250923 |
| ATP1A3 | 6.330395726 |
| ATP1B1 | 11.57219474 |
| ATP1B2 | 5.787378597 |
| ATP1B3 | 9.747669217 |
| ATP2A2 | 10.18622357 |
| ATP2B1 | 5.643419843 |
| ATP2B2 | 8.008785424 |
| ATP2B4 | 7.907507545 |
| ATP2C1 | 7.974877027 |
| ATP5A1 | 14.17093565 |
| ATP5B | 14.2342411 |
| ATP5C1 | 13.98785813 |
| ATP5D | 10.14504309 |
| ATP5E | 14.63190988 |
| ATP5F1 | 12.52833218 |
| ATP5G1 | 10.73712347 |
| ATP5G2 | 10.34876919 |
| ATP5G3 | 8.236341565 |
| ATP5H | 14.00612527 |
| ATP5I | 12.31212955 |
| ATP5J | 13.53989941 |
| ATP5J2 | 12.82697542 |
| ATP5L | 13.91187365 |
| ATP5O | 14.3064414 |
| ATP5S | 9.378047955 |
| ATP5SL | 9.691286867 |
| ATP6AP1 | 11.44758503 |
| ATP6AP2 | 12.52216843 |
| ATP6V0A1 | 11.14723315 |
| ATP6V0A2 | 9.075104454 |
| ATP6V0A4 | 5.642925565 |
| ATP6V0B | 12.28026611 |
| ATP6V0C | 10.57845556 |
| ATP6V0D1 | 9.888885 |
| ATP6V0E1 | 11.59139155 |
| ATP6V0E2L | 11.62243606 |
| ATP6V1A | 12.02331049 |
| ATP6V1B2 | 11.47793483 |
| ATP6V1C1 | 8.625461042 |
| ATP6V1D | 10.96371494 |
| ATP6V1E1 | 11.75936008 |
| ATP6V1E2 | 7.114665565 |
| ATP6V1F | 9.343000296 |
| ATP6V1G1 | 12.38854412 |
| ATP6V1G2 | 6.115198408 |
| ATP6V1H | 8.155531492 |
| ATP7A | 5.712547697 |
| ATP7B | 7.492422287 |
| ATP8B1 | 5.849779682 |
| ATP8B2 | 7.013151265 |
| ATP8B4 | 8.242604005 |
| ATP9A | 5.714302654 |
| ATP9B | 6.31105176 |
| ATPAF1 | 11.12276759 |
| ATPAF2 | 6.899748635 |
| ATPBD1B | 9.192302916 |
| ATPBD3 | 7.312366219 |
| ATPBD4 | 6.27538613 |
| ATPIF1 | 9.230727003 |
| ATR | 5.810548229 |
| ATRIP | 6.574938482 |
| ATRN | 8.618895843 |
| ATRX | 5.526572321 |
| ATXN1 | 7.584685752 |
| ATXN10 | 6.821163786 |
| ATXN2 | 10.65567374 |
| ATXN3 | 5.542139554 |
| ATXN7 | 5.818897537 |
| ATXN7L1 | 5.50650553 |
| AUH | 9.964742777 |
| AUP1 | 10.27661991 |
| AURKA | 7.319502715 |
| AURKAIP1 | 9.289455549 |
| AURKB | 6.637842042 |
| AUTS2 | 10.0273923 |
| AVEN | 8.890715873 |
| AVPI1 | 10.84410554 |
| AVPR1A | 5.506412106 |
| AVPR2 | 7.163281864 |
| AXIN1 | 7.537808351 |
| AXIN2 | 6.713536937 |
| AXL | 9.55493473 |
| AXUD1 | 12.33507509 |
| AYP1p1 | 9.720101943 |
| AZGP1 | 12.93461384 |
| AZI1 | 6.356802191 |
| AZI2 | 8.894067861 |
| AZIN1 | 9.113279795 |
| AZU1 | 5.609664819 |
| B2M | 14.9647691 |
| B3GALNT2 | 6.295193481 |
| B3GALT3 | 5.924370553 |
| B3GALT4 | 5.815011785 |
| B3GALT6 | 7.991154313 |
| B3GAT3 | 6.903832317 |
| B3GNT1 | 6.013415027 |
| B3GNT3 | 5.523913241 |
| B3GNT5 | 5.402094684 |
| B3GNT6 | 10.49241037 |
| B3GNT8 | 6.708659899 |
| B3GTL | 7.398196074 |
| B4GALNT4 | 5.980611445 |
| B4GALT1 | 10.14772718 |
| B4GALT2 | 7.632568735 |
| B4GALT3 | 9.896281076 |
| B4GALT4 | 9.164569254 |
| B4GALT5 | 10.09977325 |
| B4GALT7 | 10.21656688 |
| B9D2 | 8.194739273 |
| bA16L21.2.1 | 10.33185551 |
| BAALC | 6.673818086 |
| BAAT | 11.50539337 |
| BACE1 | 6.905970935 |
| BACE2 | 8.03165143 |
| BACH1 | 5.710020302 |
| BACH2 | 6.302897778 |
| BAD | 6.690106807 |
| BAG1 | 5.671718477 |
| BAG2 | 6.19547593 |
| BAG3 | 9.108688542 |
| BAG4 | 8.474071978 |
| BAG5 | 6.671081575 |
| BAHD1 | 6.662133368 |
| BAIAP2 | 6.82343778 |
| BAIAP2L1 | 9.473714649 |
| BAIAP2L2 | 6.64758045 |
| BAK1 | 7.199140839 |
| BAMBI | 11.61700059 |
| BANF1 | 6.883818375 |
| BANK1 | 6.030696141 |
| BANP | 9.694815851 |
| BAP1 | 7.150306709 |
| BARD1 | 7.649542524 |
| BARHL2 | 6.203120446 |
| BARX1 | 5.84380965 |
| BASP1 | 10.62858161 |
| BAT1 | 10.87695817 |
| BAT2 | 6.912338048 |
| BAT2D1 | 8.94507607 |
| BAT3 | 8.123026933 |
| BAT4 | 6.165788351 |
| BAT5 | 9.447416462 |
| BATF | 6.221083737 |
| BATF2 | 5.942699524 |
| BATF3 | 7.646802364 |
| BAX | 5.644579652 |
| BAZ1A | 8.594298582 |
| BAZ1B | 6.656737776 |
| BAZ2A | 5.839298573 |
| BAZ2B | 8.711937288 |
| BBC3 | 8.816813553 |
| BBOX1 | 11.28638154 |
| BBS1 | 7.293222567 |
| BBS2 | 8.177876899 |
| BBS4 | 8.55784957 |
| BBS5 | 5.65924264 |
| BBS9 | 6.580982224 |
| BBX | 9.539739006 |
| BCAM | 6.552232741 |
| BCAN | 5.754759579 |
| BCAP29 | 8.702202503 |
| BCAP31 | 11.78125439 |
| BCAR1 | 6.971550506 |
| BCAR3 | 9.353884266 |
| BCAS1 | 5.815822849 |
| BCAS2 | 6.592472181 |
| BCAS3 | 5.759382428 |
| BCAS4 | 6.091139258 |
| BCAT1 | 6.765857325 |
| BCAT2 | 7.521054642 |
| BCCIP | 10.71300211 |
| BCDIN3D | 9.722712456 |
| BCDO2 | 9.379348633 |
| BCHE | 9.70766731 |
| BCKDHA | 10.7737783 |
| BCKDHB | 9.402924562 |
| BCKDK | 12.08360231 |
| BCL10 | 6.324216073 |
| BCL11A | 5.93590207 |
| BCL11B | 5.548625126 |
| BCL2 | 6.368242741 |
| BCL2A1 | 6.063169426 |
| BCL2L1 | 8.972704025 |
| BCL2L10 | 7.072379951 |
| BCL2L11 | 5.548331926 |
| BCL2L12 | 7.507067238 |
| BCL2L13 | 11.20854885 |
| BCL2L14 | 6.000844988 |
| BCL2L2 | 10.09306781 |
| BCL3 | 7.994905742 |
| BCL6 | 10.96991177 |
| BCL6B | 6.994896424 |
| BCL7A | 6.654362252 |
| BCL7B | 7.581501266 |
| BCL7C | 7.58845186 |
| BCL9 | 7.14505047 |
| BCL9L | 5.857873271 |
| BCLAF1 | 10.67746456 |
| BCMO1 | 6.283037746 |
| BCOR | 7.448007715 |
| BCORL1 | 6.444875108 |
| BCR | 5.858429087 |
| BCS1L | 8.363827216 |
| BCYRN1 | 10.10183093 |
| BDH | 9.559529077 |
| BDH2 | 5.898397339 |
| BDKRB2 | 6.086510176 |
| BDNF | 6.178371387 |
| BECN1 | 9.966225691 |
| BET1 | 10.03374218 |
| BET1L | 11.61196451 |
| BEX1 | 7.449943468 |
| BEX5 | 7.347445355 |
| BEXL1 | 7.693056609 |
| BFAR | 9.743746572 |
| BGLAP | 6.061318427 |
| BGN | 9.719545451 |
| BHLHB2 | 12.2494287 |
| BHLHB5 | 5.900622543 |
| BHLHB9 | 6.527769706 |
| BHMT | 10.76910135 |
| BHMT2 | 11.57297127 |
| BICD1 | 5.774100245 |
| BICD2 | 10.66517347 |
| BID | 10.001498 |
| BIN1 | 9.118067789 |
| BIN2 | 6.31834648 |
| BIRC1 | 5.757894909 |
| BIRC2 | 7.51683685 |
| BIRC3 | 8.330591356 |
| BIRC6 | 7.765314465 |
| BIRC8 | 5.870570889 |
| BIVM | 8.267843025 |
| BLCAP | 9.58586185 |
| BLM | 6.075255615 |
| BLMH | 9.873657835 |
| BLNK | 9.889978724 |
| BLOC1S1 | 11.59382299 |
| BLOC1S2 | 5.71686674 |
| BLVRA | 9.810292463 |
| BLVRB | 9.552750402 |
| BLZF1 | 8.651973631 |
| BMF | 5.545136923 |
| BMI1 | 10.84278093 |
| BMP1 | 6.391889964 |
| BMP2 | 7.480400691 |
| BMP2K | 6.428773965 |
| BMP2KL | 6.032268901 |
| BMP4 | 6.147123803 |
| BMP5 | 5.574438646 |
| BMP6 | 6.308524737 |
| BMP8B | 5.723898777 |
| BMPER | 5.816314577 |
| BMPR1A | 6.767617591 |
| BMPR2 | 9.361559558 |
| BMS1 | 10.4999851 |
| BNIP1 | 8.097951547 |
| BNIP2 | 10.09504754 |
| BNIP3 | 12.70744819 |
| BNIP3L | 9.271174536 |
| BNIPL | 5.720400891 |
| BOC | 5.873535351 |
| BOK | 8.039499081 |
| BOLA1 | 7.632941815 |
| BOLA2 | 10.87312026 |
| BOLA3 | 12.38837209 |
| BOLL | 8.11085921 |
| BOP1 | 10.63242058 |
| BPESC1 | 6.226183647 |
| BPGM | 7.62937282 |
| BPHL | 10.89663786 |
| BPI | 5.461886357 |
| BPIL1 | 5.683345178 |
| BPNT1 | 8.424302364 |
| BRAF | 5.875863018 |
| BRCA1 | 6.861303618 |
| BRCC3 | 6.762040409 |
| BRD1 | 5.675650006 |
| BRD2 | 11.04291416 |
| BRD3 | 8.509611481 |
| BRD4 | 7.438628858 |
| BRD7 | 8.490082005 |
| BRD8 | 7.16365076 |
| BRD9 | 10.09861052 |
| BRE | 7.084285625 |
| BRF1 | 5.581540406 |
| BRF2 | 7.659924459 |
| BRI3 | 11.89373235 |
| BRI3BP | 7.630444736 |
| BRMS1 | 7.728952969 |
| BRMS1L | 8.490418357 |
| BRP44 | 13.27531986 |
| BRP44L | 11.74743748 |
| BRPF1 | 6.032458007 |
| BRPF3 | 9.563488919 |
| BRRN1 | 6.119438518 |
| BRSK1 | 7.553176889 |
| BRUNOL4 | 6.321679428 |
| BRUNOL6 | 5.961397454 |
| BRWD1 | 7.509959598 |
| BRWD2 | 7.918803161 |
| BRWD3 | 6.330684063 |
| BSCL2 | 11.24365695 |
| BSDC1 | 10.80330664 |
| BSG | 9.233530663 |
| BSN | 5.615417659 |
| BSPRY | 8.295216947 |
| BST1 | 6.112927662 |
| BST2 | 12.10034704 |
| BTAF1 | 8.629063329 |
| BTBD1 | 8.103581064 |
| BTBD10 | 9.108129895 |
| BTBD11 | 5.416452181 |
| BTBD12 | 6.609439347 |
| BTBD14A | 8.461576943 |
| BTBD14B | 5.802554175 |
| BTBD15 | 6.831024708 |
| BTBD16 | 8.407380927 |
| BTBD2 | 9.984106745 |
| BTBD3 | 8.26270829 |
| BTBD5 | 8.822149954 |
| BTBD6 | 8.093497514 |
| BTBD7 | 7.190918933 |
| BTBD9 | 5.760164378 |
| BTD | 9.507325884 |
| BTF3 | 10.19167198 |
| BTF3L4 | 9.684674522 |
| BTG1 | 13.58587259 |
| BTG2 | 6.699893683 |
| BTG3 | 9.283699245 |
| BTK | 8.29576722 |
| BTLA | 6.723254657 |
| BTN2A1 | 7.694001397 |
| BTN2A2 | 7.34030672 |
| BTN3A1 | 7.416891758 |
| BTN3A2 | 7.820623967 |
| BTN3A3 | 7.047928005 |
| BTNL3 | 5.534392097 |
| BTNL9 | 5.512632564 |
| BTRC | 6.363780469 |
| BUB1 | 6.323020287 |
| BUB1B | 5.725292916 |
| BUB3 | 10.01174256 |
| BUD13 | 7.719272039 |
| BUD31 | 11.70224769 |
| BXDC1 | 11.90910162 |
| BXDC2 | 11.0775945 |
| BXDC5 | 9.551495546 |
| BYSL | 8.621233338 |
| BZW1 | 7.429933154 |
| BZW2 | 10.46426129 |
| C10orf10 | 11.6292009 |
| C10orf104 | 10.12253084 |
| C10orf108 | 6.567457477 |
| C10orf11 | 9.489820686 |
| C10orf116 | 11.28513207 |
| C10orf118 | 5.823251481 |
| C10orf119 | 7.549789997 |
| C10orf125 | 10.69282551 |
| C10orf128 | 6.287348386 |
| C10orf137 | 6.746384426 |
| C10orf2 | 7.379298814 |
| C10orf26 | 8.435262323 |
| C10orf28 | 7.875029981 |
| C10orf30 | 5.735700477 |
| C10orf32 | 11.09007511 |
| C10orf33 | 6.351090042 |
| C10orf35 | 7.529664007 |
| C10orf4 | 6.035501126 |
| C10orf46 | 5.673076771 |
| C10orf47 | 8.16937066 |
| C10orf49 | 5.747831825 |
| C10orf55 | 6.072442374 |
| C10orf56 | 9.653307984 |
| C10orf57 | 10.18944298 |
| C10orf58 | 10.90163216 |
| C10orf59 | 8.058513119 |
| C10orf6 | 7.27019079 |
| C10orf61 | 7.887825847 |
| C10orf65 | 9.508953124 |
| C10orf72 | 5.987409544 |
| C10orf73 | 5.78566688 |
| C10orf76 | 8.159353062 |
| C10orf78 | 6.888179328 |
| C10orf82 | 5.627511515 |
| C10orf83 | 5.840377677 |
| C10orf85 | 5.620867689 |
| C10orf88 | 5.980912452 |
| C10orf92 | 6.914068968 |
| C10orf93 | 6.148934946 |
| C10orf96 | 5.957783758 |
| C10orf97 | 9.901975559 |
| C11orf10 | 14.23841794 |
| C11orf16 | 5.720584134 |
| C11orf17 | 9.582446231 |
| C11orf2 | 10.93600485 |
| C11orf24 | 8.972956281 |
| C11orf30 | 6.991465086 |
| C11orf31 | 6.063365937 |
| C11orf42 | 5.622694373 |
| C11orf46 | 8.980478271 |
| C11orf48 | 10.73966225 |
| C11orf51 | 7.581611198 |
| C11orf52 | 7.991177723 |
| C11orf54 | 10.07624794 |
| C11orf56 | 8.255522732 |
| C11orf57 | 8.528154491 |
| C11orf58 | 7.495902561 |
| C11orf59 | 11.20743462 |
| C11orf60 | 7.103291872 |
| C11orf61 | 5.686783408 |
| C11orf63 | 5.540461089 |
| C11orf67 | 10.74527688 |
| C11orf68 | 6.557683535 |
| C11orf71 | 9.718838193 |
| C11orf73 | 9.382397591 |
| C11orf74 | 10.06384962 |
| C11orf75 | 12.14377105 |
| C11orf77 | 5.679481935 |
| C11orf79 | 11.76517775 |
| C11orf80 | 6.077948224 |
| C11orf82 | 6.247667414 |
| C11orf84 | 6.608012538 |
| C11orf9 | 6.205635567 |
| C12orf10 | 11.26529844 |
| C12orf11 | 8.302741631 |
| C12orf23 | 9.204156289 |
| C12orf24 | 9.560566522 |
| C12orf26 | 8.439775636 |
| C12orf29 | 9.77390858 |
| C12orf30 | 6.643663873 |
| C12orf31 | 9.655027021 |
| C12orf32 | 7.491359808 |
| C12orf34 | 6.309446411 |
| C12orf35 | 7.883666861 |
| C12orf39 | 5.513267563 |
| C12orf4 | 8.32309133 |
| C12orf40 | 5.780896917 |
| C12orf41 | 10.18079872 |
| C12orf43 | 8.260064114 |
| C12orf44 | 10.12351923 |
| C12orf45 | 8.532088302 |
| C12orf47 | 9.637423982 |
| C12orf48 | 6.560174369 |
| C12orf49 | 8.00051118 |
| C12orf5 | 8.214646019 |
| C12orf52 | 9.632063614 |
| C12orf57 | 12.75731035 |
| C12orf58 | 7.051425876 |
| C12orf60 | 6.377911445 |
| C12orf61 | 6.580519982 |
| C12orf62 | 11.12364176 |
| C12orf65 | 8.488838621 |
| C13orf16 | 5.658343783 |
| C13orf23 | 7.180405175 |
| C13orf27 | 10.50705563 |
| C13orf3 | 6.172234032 |
| C13orf31 | 5.881820856 |
| C13orf33 | 5.674659222 |
| C13orf34 | 7.067693228 |
| C13orf7 | 6.142624502 |
| C14orf1 | 7.288626858 |
| C14orf100 | 9.850603881 |
| C14orf101 | 5.723456681 |
| C14orf102 | 9.99578635 |
| C14orf103 | 7.069203179 |
| C14orf104 | 8.68202589 |
| C14orf105 | 7.557835161 |
| C14orf106 | 8.421671623 |
| C14orf108 | 9.3224646 |
| C14orf11 | 11.00496176 |
| C14orf112 | 12.31312934 |
| C14orf118 | 5.792892165 |
| C14orf120 | 9.455547912 |
| C14orf124 | 6.052160188 |
| C14orf125 | 5.840368919 |
| C14orf126 | 6.969014109 |
| C14orf129 | 11.42799268 |
| C14orf130 | 8.680655505 |
| C14orf131 | 5.566478164 |
| C14orf132 | 7.225184632 |
| C14orf133 | 6.677766429 |
| C14orf135 | 8.542058731 |
| C14orf138 | 7.194077273 |
| C14orf139 | 5.705324929 |
| C14orf140 | 7.444941596 |
| C14orf142 | 10.08405917 |
| C14orf147 | 9.962794458 |
| C14orf149 | 8.711678968 |
| C14orf156 | 13.9896631 |
| C14orf159 | 8.754882858 |
| C14orf162 | 5.708067618 |
| C14orf165 | 5.741020515 |
| C14orf166 | 11.83248427 |
| C14orf169 | 8.801101973 |
| C14orf172 | 6.884343691 |
| C14orf173 | 7.242073218 |
| C14orf174 | 7.851601017 |
| C14orf179 | 10.71293744 |
| C14orf2 | 10.08086825 |
| C14orf21 | 6.226529524 |
| C14orf24 | 8.279744919 |
| C14orf28 | 7.328244164 |
| C14orf32 | 9.107442007 |
| C14orf37 | 6.392784129 |
| C14orf4 | 7.087633995 |
| C14orf43 | 8.718955371 |
| C14orf45 | 6.320256845 |
| C14orf48 | 7.265140383 |
| C14orf49 | 6.152357491 |
| C14orf50 | 5.700321787 |
| C14orf68 | 10.00676497 |
| C14orf72 | 5.686406453 |
| C14orf73 | 8.318605295 |
| C14orf78 | 8.489153038 |
| C14orf79 | 5.803067216 |
| C14orf80 | 7.89819921 |
| C14orf82 | 5.732022891 |
| C14orf92 | 8.012375552 |
| C14orf93 | 7.87759068 |
| C14orf94 | 8.845263367 |
| C15orf15 | 12.06391177 |
| C15orf17 | 8.197371971 |
| C15orf23 | 6.709803507 |
| C15orf24 | 11.48864652 |
| C15orf28 | 6.606021036 |
| C15orf29 | 7.37650667 |
| C15orf38 | 6.514273778 |
| C15orf39 | 6.331801396 |
| C15orf40 | 6.465809197 |
| C15orf41 | 6.569240636 |
| C15orf44 | 6.431159588 |
| C15orf48 | 7.405569462 |
| C15orf52 | 6.618399171 |
| C15orf54 | 5.854234823 |
| C16orf14 | 8.88536016 |
| C16orf28 | 6.379254055 |
| C16orf30 | 7.897126286 |
| C16orf33 | 10.99285406 |
| C16orf35 | 6.205498944 |
| C16orf42 | 7.878262568 |
| C16orf45 | 7.433992492 |
| C16orf48 | 8.98008686 |
| C16orf5 | 6.190632481 |
| C16orf50 | 5.798316003 |
| C16orf52 | 6.326959612 |
| C16orf53 | 8.435628155 |
| C16orf54 | 6.476081589 |
| C16orf56 | 7.636283647 |
| C16orf57 | 8.352387955 |
| C16orf58 | 11.52237667 |
| C16orf61 | 12.34875022 |
| C16orf63 | 10.49939484 |
| C16orf69 | 9.623499343 |
| C16orf7 | 6.908250295 |
| C16orf70 | 6.418966101 |
| C16orf72 | 9.166020949 |
| C16orf75 | 7.244804224 |
| C16orf79 | 5.666539583 |
| C16orf80 | 9.815708376 |
| C16orf82 | 5.770427693 |
| C16orf84 | 5.640425824 |
| C17orf28 | 6.169645029 |
| C17orf37 | 7.326573931 |
| C17orf39 | 5.953891299 |
| C17orf44 | 6.372217522 |
| C17orf45 | 13.19548264 |
| C17orf48 | 8.658042886 |
| C17orf49 | 10.16508581 |
| C17orf53 | 6.30923688 |
| C17orf56 | 7.584909163 |
| C17orf58 | 10.01668584 |
| C17orf59 | 9.697269071 |
| C17orf60 | 5.993547028 |
| C17orf61 | 13.22067385 |
| C17orf62 | 8.922363369 |
| C17orf63 | 7.365689397 |
| C17orf65 | 5.494915938 |
| C17orf67 | 5.852221861 |
| C17orf68 | 6.96533661 |
| C17orf70 | 10.3131938 |
| C17orf71 | 5.779428736 |
| C17orf74 | 6.031070448 |
| C17orf75 | 6.715195396 |
| C17orf76 | 6.389864536 |
| C17orf77 | 6.063399606 |
| C17orf79 | 11.47526967 |
| C17orf80 | 6.117220015 |
| C17orf81 | 6.653069975 |
| C17orf82 | 5.627973944 |
| C17orf85 | 7.210101507 |
| C17orf87 | 7.35813871 |
| C17orf91 | 10.43405501 |
| C18orf1 | 6.934230696 |
| C18orf10 | 7.714515489 |
| C18orf17 | 12.61206272 |
| C18orf19 | 8.806610716 |
| C18orf20 | 5.390426981 |
| C18orf21 | 9.186330061 |
| C18orf22 | 8.755815871 |
| C18orf24 | 6.040222714 |
| C18orf25 | 9.561180026 |
| C18orf26 | 5.715002761 |
| C18orf37 | 9.824658687 |
| C18orf45 | 7.524961092 |
| C18orf51 | 5.699383561 |
| C18orf55 | 10.09635227 |
| C18orf56 | 6.096948617 |
| C18orf8 | 9.819441618 |
| C19orf10 | 13.33048818 |
| C19orf12 | 9.032226838 |
| C19orf2 | 11.86160253 |
| C19orf22 | 9.645474513 |
| C19orf23 | 7.136537615 |
| C19orf24 | 8.398465776 |
| C19orf25 | 7.020975714 |
| C19orf26 | 6.189437953 |
| C19orf28 | 7.625353745 |
| C19orf33 | 5.494993044 |
| C19orf36 | 5.678459428 |
| C19orf39 | 5.750621511 |
| C19orf4 | 5.624229593 |
| C19orf40 | 5.827215574 |
| C19orf43 | 11.12582477 |
| C19orf47 | 5.599052559 |
| C19orf48 | 9.80167784 |
| C19orf49 | 6.605787861 |
| C19orf50 | 9.670867559 |
| C19orf52 | 8.075332982 |
| C19orf56 | 12.39475775 |
| C19orf6 | 6.582522386 |
| C19orf62 | 10.74949027 |
| C19orf70 | 12.03681293 |
| C1D | 5.56914675 |
| C1GALT1 | 7.239823505 |
| C1GALT1C1 | 11.2229589 |
| C1orf101 | 6.379449511 |
| C1orf102 | 6.286729197 |
| C1orf105 | 5.969853674 |
| C1orf107 | 5.688632029 |
| C1orf108 | 8.255443603 |
| C1orf110 | 5.634517221 |
| C1orf112 | 8.5062714 |
| C1orf115 | 11.06762022 |
| C1orf120 | 5.69129684 |
| C1orf121 | 9.49749203 |
| C1orf122 | 9.133190471 |
| C1orf123 | 10.1799507 |
| C1orf124 | 6.835395247 |
| C1orf128 | 10.78895362 |
| C1orf130 | 8.317436154 |
| C1orf131 | 8.63437826 |
| C1orf135 | 5.638950483 |
| C1orf141 | 6.403345944 |
| C1orf142 | 7.384309816 |
| C1orf144 | 11.35166996 |
| C1orf149 | 6.31497159 |
| C1orf152 | 6.05440885 |
| C1orf156 | 6.72755507 |
| C1orf159 | 5.427770372 |
| C1orf160 | 8.111543776 |
| C1orf161 | 5.961224176 |
| C1orf162 | 10.72392853 |
| C1orf163 | 7.873898641 |
| C1orf166 | 8.36851445 |
| C1orf168 | 8.176651705 |
| C1orf170 | 5.792844916 |
| C1orf172 | 5.614601227 |
| C1orf174 | 10.90068345 |
| C1orf176 | 7.726684693 |
| C1orf177 | 6.019261532 |
| C1orf180 | 5.896628132 |
| C1orf181 | 8.675092385 |
| C1orf183 | 5.960094159 |
| C1orf188 | 5.840144866 |
| C1orf19 | 9.536634514 |
| C1orf192 | 6.144677162 |
| C1orf198 | 7.807777227 |
| C1orf2 | 6.554532126 |
| C1orf201 | 6.140390066 |
| C1orf21 | 6.045876382 |
| C1orf212 | 9.474004376 |
| C1orf216 | 7.134422153 |
| C1orf218 | 8.655758836 |
| C1orf24 | 8.279108005 |
| C1orf25 | 7.478570732 |
| C1orf26 | 7.214230928 |
| C1orf27 | 5.923174429 |
| C1orf31 | 7.476957295 |
| C1orf33 | 8.821076899 |
| C1orf35 | 7.02601593 |
| C1orf37 | 11.85473128 |
| C1orf41 | 9.557912262 |
| C1orf43 | 11.30239168 |
| C1orf48 | 11.28031042 |
| C1orf50 | 10.82597885 |
| C1orf51 | 6.012443335 |
| C1orf52 | 9.44317106 |
| C1orf53 | 11.44479559 |
| C1orf54 | 8.172673501 |
| C1orf55 | 9.881800169 |
| C1orf56 | 5.729777663 |
| C1orf57 | 10.45538751 |
| C1orf58 | 5.491097104 |
| C1orf59 | 7.401089519 |
| C1orf63 | 8.613480826 |
| C1orf66 | 8.83566552 |
| C1orf67 | 6.031925723 |
| C1orf69 | 6.002404074 |
| C1orf71 | 10.79961457 |
| C1orf74 | 7.845610947 |
| C1orf77 | 10.18406726 |
| C1orf80 | 6.572118524 |
| C1orf83 | 5.80731151 |
| C1orf84 | 5.822689215 |
| C1orf85 | 11.04437851 |
| C1orf86 | 7.094557652 |
| C1orf89 | 5.804361245 |
| C1orf9 | 6.016474644 |
| C1orf90 | 6.249711407 |
| C1orf91 | 5.368021662 |
| C1orf92 | 6.604321575 |
| C1orf93 | 7.27847588 |
| C1orf94 | 5.999480048 |
| C1orf95 | 6.092479795 |
| C1orf97 | 9.400784417 |
| C1QA | 12.15362332 |
| C1QB | 12.2865709 |
| C1QBP | 12.39681327 |
| C1QC | 11.5542287 |
| C1QL2 | 5.936821995 |
| C1QL3 | 5.629669179 |
| C1QR1 | 10.60995182 |
| C1QTNF1 | 6.017349913 |
| C1QTNF5 | 8.507769838 |
| C1QTNF6 | 6.290353808 |
| C1QTNF8 | 6.201915272 |
| C1QTNF9 | 6.254995739 |
| C1R | 9.669907509 |
| C1RL | 11.16795489 |
| C1S | 11.72378364 |
| C2 | 10.93435161 |
| C20orf107 | 5.625117494 |
| C20orf108 | 10.5814362 |
| C20orf11 | 9.741740344 |
| C20orf111 | 6.341300924 |
| C20orf112 | 5.709405593 |
| C20orf116 | 11.37473743 |
| C20orf12 | 5.588333278 |
| C20orf121 | 9.501517536 |
| C20orf127 | 12.84839287 |
| C20orf129 | 6.004660324 |
| C20orf133 | 5.4280581 |
| C20orf14 | 7.102774706 |
| C20orf149 | 6.264712587 |
| C20orf160 | 6.731271351 |
| C20orf177 | 7.752472771 |
| C20orf18 | 8.616441854 |
| C20orf19 | 6.304353641 |
| C20orf194 | 6.009407817 |
| C20orf196 | 6.317952115 |
| C20orf197 | 5.745347667 |
| C20orf20 | 9.431645489 |
| C20orf22 | 8.265907593 |
| C20orf24 | 13.95125745 |
| C20orf26 | 5.952486707 |
| C20orf27 | 9.006502638 |
| C20orf28 | 5.859928191 |
| C20orf29 | 8.331888523 |
| C20orf3 | 12.4331087 |
| C20orf30 | 13.58969138 |
| C20orf35 | 10.30967705 |
| C20orf39 | 5.867919552 |
| C20orf4 | 9.167470007 |
| C20orf43 | 11.84322271 |
| C20orf44 | 10.23871456 |
| C20orf45 | 6.400406941 |
| C20orf46 | 7.378170378 |
| C20orf52 | 13.11201168 |
| C20orf55 | 7.276828884 |
| C20orf59 | 6.393778887 |
| C20orf7 | 9.912609514 |
| C20orf72 | 9.180356994 |
| C20orf74 | 6.211106965 |
| C20orf82 | 7.884943228 |
| C20orf86 | 5.623873474 |
| C20orf91 | 5.735362173 |
| C20orf94 | 5.94012275 |
| C20orf95 | 6.159552255 |
| C21orf119 | 7.17213424 |
| C21orf121 | 7.097388418 |
| C21orf124 | 5.745665942 |
| C21orf127 | 8.019304793 |
| C21orf128 | 5.757138492 |
| C21orf129 | 7.767615099 |
| C21orf2 | 8.776675119 |
| C21orf25 | 9.65758218 |
| C21orf33 | 11.59348533 |
| C21orf34 | 7.446912457 |
| C21orf41 | 6.736432898 |
| C21orf45 | 7.690791547 |
| C21orf51 | 6.58589297 |
| C21orf55 | 13.48697658 |
| C21orf57 | 8.740050618 |
| C21orf59 | 8.549861447 |
| C21orf62 | 5.90438273 |
| C21orf63 | 7.31951176 |
| C21orf66 | 5.762676976 |
| C21orf67 | 7.048305374 |
| C21orf69 | 5.80929939 |
| C21orf7 | 8.051272036 |
| C21orf70 | 7.591626234 |
| C21orf81 | 5.796270147 |
| C21orf84 | 6.488602034 |
| C21orf91 | 5.952592723 |
| C22orf13 | 12.10135999 |
| C22orf15 | 5.884338886 |
| C22orf25 | 7.270419772 |
| C22orf28 | 11.12087125 |
| C22orf32 | 7.501372777 |
| C22orf33 | 6.06276128 |
| C22orf39 | 5.646021543 |
| C22orf9 | 6.538095294 |
| C2orf12 | 5.664027795 |
| C2orf13 | 5.612181256 |
| C2orf15 | 5.584272241 |
| C2orf17 | 7.848468333 |
| C2orf18 | 8.717672314 |
| C2orf24 | 7.953461557 |
| C2orf25 | 13.00160571 |
| C2orf26 | 12.57234305 |
| C2orf28 | 10.64839201 |
| C2orf29 | 11.07387476 |
| C2orf30 | 11.40130708 |
| C2orf32 | 8.860284645 |
| C2orf33 | 8.631431106 |
| C2orf34 | 6.808265632 |
| C2orf40 | 5.625937898 |
| C2orf42 | 9.326487443 |
| C2orf43 | 7.905385291 |
| C2orf44 | 8.423186442 |
| C2orf47 | 11.86754929 |
| C2orf49 | 8.882398109 |
| C2orf50 | 5.742760998 |
| C2orf58 | 5.858078489 |
| C2orf62 | 6.178754092 |
| C2orf63 | 5.800718765 |
| C2orf64 | 9.576437276 |
| C2orf7 | 11.07543798 |
| C2orf71 | 5.906445463 |
| C2orf79 | 9.120417065 |
| C2orf83 | 5.66506697 |
| C3 | 14.54060584 |
| C3AR1 | 7.580770822 |
| C3orf1 | 11.73331733 |
| C3orf10 | 9.862487825 |
| C3orf15 | 5.633771745 |
| C3orf17 | 6.437759401 |
| C3orf19 | 7.134517038 |
| C3orf21 | 8.699491274 |
| C3orf22 | 5.692814156 |
| C3orf23 | 7.620534996 |
| C3orf26 | 8.937388799 |
| C3orf29 | 10.69953828 |
| C3orf31 | 8.659585183 |
| C3orf33 | 6.873622936 |
| C3orf34 | 5.539422617 |
| C3orf37 | 9.218044606 |
| C3orf38 | 6.953576997 |
| C3orf39 | 7.589796411 |
| C3orf41 | 6.725704013 |
| C3orf54 | 7.128291363 |
| C3orf58 | 8.828982827 |
| C3orf59 | 6.936366438 |
| C3orf60 | 6.269570628 |
| C3orf62 | 5.543964109 |
| C3orf63 | 5.911490671 |
| C3orf64 | 6.108100573 |
| C4B | 5.93463911 |
| C4BPA | 14.83620051 |
| C4BPB | 13.72651512 |
| C4orf12 | 5.859762884 |
| C4orf13 | 6.32011553 |
| C4orf14 | 9.279624406 |
| C4orf16 | 8.539886154 |
| C4orf18 | 7.928771615 |
| C4orf19 | 10.63141823 |
| C4orf27 | 9.602020344 |
| C4orf32 | 8.224277394 |
| C4orf33 | 8.675607065 |
| C4orf34 | 13.57236961 |
| C4orf38 | 5.427532003 |
| C4orf41 | 8.196108615 |
| C4orf42 | 6.034060295 |
| C4orf6 | 5.733942806 |
| C4orf8 | 6.649917005 |
| C5 | 14.19022552 |
| C5AR1 | 7.089067031 |
| C5orf13 | 8.154789764 |
| C5orf14 | 8.922453603 |
| C5orf15 | 12.00198778 |
| C5orf21 | 8.898399307 |
| C5orf22 | 7.147221603 |
| C5orf24 | 7.446132087 |
| C5orf28 | 6.503476133 |
| C5orf29 | 5.972734446 |
| C5orf3 | 7.049733362 |
| C5orf30 | 5.628624363 |
| C5orf33 | 9.912092319 |
| C5orf35 | 9.433030297 |
| C5orf37 | 6.62932845 |
| C5orf39 | 6.256414281 |
| C5orf4 | 7.189205551 |
| C5orf41 | 8.338945317 |
| C5orf5 | 8.738614674 |
| C5orf51 | 9.338629141 |
| C6 | 13.99960246 |
| C6orf1 | 10.34701554 |
| C6orf105 | 6.470355348 |
| C6orf106 | 7.826082169 |
| C6orf108 | 11.77497969 |
| C6orf111 | 7.533975264 |
| C6orf114 | 6.363314937 |
| C6orf115 | 11.1069855 |
| C6orf120 | 7.984489793 |
| C6orf122 | 5.708934892 |
| C6orf125 | 11.37575335 |
| C6orf129 | 10.86528993 |
| C6orf130 | 9.82943784 |
| C6orf134 | 6.599495528 |
| C6orf136 | 9.170804169 |
| C6orf142 | 6.97575977 |
| C6orf145 | 8.58753919 |
| C6orf150 | 5.730656915 |
| C6orf151 | 6.714088347 |
| C6orf153 | 11.29126336 |
| C6orf160 | 12.74828406 |
| C6orf162 | 6.182810735 |
| C6orf163 | 5.728583554 |
| C6orf165 | 5.763782421 |
| C6orf167 | 5.748640742 |
| C6orf173 | 7.137601453 |
| C6orf188 | 5.839253637 |
| C6orf189 | 6.209775581 |
| C6orf190 | 5.832038177 |
| C6orf192 | 9.124157171 |
| C6orf195 | 5.832424562 |
| C6orf199 | 5.902408269 |
| C6orf203 | 7.992787077 |
| C6orf204 | 6.066879979 |
| C6orf206 | 8.568586468 |
| C6orf208 | 5.775366006 |
| C6orf211 | 8.293915769 |
| C6orf25 | 5.942892333 |
| C6orf27 | 6.056894542 |
| C6orf35 | 5.778796758 |
| C6orf47 | 5.946408082 |
| C6orf48 | 7.40631205 |
| C6orf49 | 12.51022511 |
| C6orf52 | 6.72742136 |
| C6orf57 | 8.931338278 |
| C6orf60 | 5.626819554 |
| C6orf61 | 6.909805478 |
| C6orf62 | 11.19891688 |
| C6orf64 | 8.715362647 |
| C6orf66 | 8.946880715 |
| C6orf70 | 8.380221648 |
| C6orf72 | 11.88837495 |
| C6orf75 | 8.188889831 |
| C6orf79 | 11.17919807 |
| C6orf85 | 8.810864082 |
| C6orf89 | 7.295830003 |
| C7 | 10.4125427 |
| C7orf10 | 9.392979744 |
| C7orf11 | 8.659229661 |
| C7orf20 | 7.429043486 |
| C7orf23 | 8.818793889 |
| C7orf25 | 8.57367058 |
| C7orf26 | 8.694701282 |
| C7orf27 | 9.806840169 |
| C7orf28A | 7.865508863 |
| C7orf28B | 8.525817929 |
| C7orf29 | 6.744697466 |
| C7orf30 | 12.71406048 |
| C7orf31 | 5.639204456 |
| C7orf36 | 8.947956452 |
| C7orf38 | 12.15964032 |
| C7orf46 | 6.056415191 |
| C7orf47 | 10.08269706 |
| C7orf49 | 7.950346139 |
| C7orf51 | 6.334055336 |
| C7orf54 | 7.303288645 |
| C8A | 14.07694041 |
| C8B | 13.67367462 |
| C8G | 13.59267326 |
| C8orf30A | 8.571889221 |
| C8orf32 | 8.332382096 |
| C8orf33 | 8.408844069 |
| C8orf35 | 11.18571592 |
| C8orf38 | 9.772548518 |
| C8orf4 | 10.80023695 |
| C8orf40 | 11.99318962 |
| C8orf41 | 9.23304559 |
| C8orf42 | 7.635542749 |
| C8orf44 | 5.864850979 |
| C8orf46 | 5.938075432 |
| C8orf47 | 5.852667189 |
| C8orf48 | 7.778223194 |
| C8orf49 | 5.731321216 |
| C8orf51 | 5.94963634 |
| C8orf54 | 5.762010385 |
| C8orf55 | 11.90578176 |
| C8orf59 | 12.00673908 |
| C8orf61 | 5.705521382 |
| C8orf72 | 7.077444449 |
| C8orf76 | 11.28629975 |
| C8ORFK29 | 5.833814902 |
| C8ORFK32 | 6.282913522 |
| C8ORFK36 | 5.844368793 |
| C9 | 14.25306204 |
| C9orf103 | 10.73673023 |
| C9orf10OS | 10.36421474 |
| C9orf114 | 9.249236446 |
| C9orf116 | 6.557981504 |
| C9orf119 | 10.20195066 |
| C9orf123 | 8.289478344 |
| C9orf125 | 5.493296546 |
| C9orf127 | 6.336370636 |
| C9orf130 | 8.760479701 |
| C9orf132 | 6.138145345 |
| C9orf142 | 10.10128993 |
| C9orf150 | 8.171479339 |
| C9orf152 | 5.614309206 |
| C9orf156 | 8.442399563 |
| C9orf16 | 5.769156765 |
| C9orf165 | 5.925606552 |
| C9orf21 | 8.425490645 |
| C9orf23 | 8.258136936 |
| C9orf25 | 5.492251584 |
| C9orf3 | 8.496245828 |
| C9orf30 | 9.337997428 |
| C9orf37 | 7.326930214 |
| C9orf38 | 5.680335884 |
| C9orf39 | 5.945951704 |
| C9orf40 | 6.710882235 |
| C9orf41 | 5.938654957 |
| C9orf45 | 5.777269359 |
| C9orf46 | 10.40417489 |
| C9orf5 | 9.893097595 |
| C9orf58 | 9.001169598 |
| C9orf6 | 8.30675461 |
| C9orf64 | 8.616790958 |
| C9orf66 | 6.282121183 |
| C9orf7 | 7.779221185 |
| C9orf72 | 6.98939789 |
| C9orf75 | 6.927406956 |
| C9orf78 | 11.99845439 |
| C9orf79 | 6.503564268 |
| C9orf80 | 8.907874453 |
| C9orf82 | 8.497520223 |
| C9orf85 | 7.010579858 |
| C9orf86 | 6.409730053 |
| C9orf89 | 8.479949728 |
| C9orf9 | 6.479543484 |
| C9orf90 | 6.355393554 |
| C9orf91 | 6.53936197 |
| C9orf95 | 10.30038718 |
| C9orf97 | 6.080159324 |
| CA1 | 6.574247651 |
| CA13 | 7.238968241 |
| CA2 | 13.39625073 |
| CA4 | 5.998297904 |
| CA5A | 11.02943378 |
| CA5B | 7.891703704 |
| CA9 | 5.861347299 |
| CAB39 | 11.39189485 |
| CAB39L | 7.330227393 |
| CABC1 | 10.98968596 |
| CABIN1 | 6.066605363 |
| CABLES1 | 7.720380217 |
| CABLES2 | 6.718523346 |
| CABP1 | 7.785789281 |
| CACHD1 | 5.926928664 |
| CACNA1B | 5.495975027 |
| CACNA1D | 5.826348968 |
| CACNA1H | 6.561902116 |
| CACNA2D4 | 6.044207308 |
| CACNB3 | 5.789127976 |
| CACNG2 | 6.063672686 |
| CACNG6 | 5.766574757 |
| CACYBP | 7.982409794 |
| CAD | 6.570871176 |
| CADM1 | 9.105390536 |
| CADM4 | 6.252104232 |
| CADPS | 6.480702902 |
| CADPS2 | 9.330395164 |
| CAGE1 | 6.132493851 |
| CALCA | 6.423603495 |
| CALCB | 6.662737788 |
| CALCOCO1 | 5.453726671 |
| CALCOCO2 | 8.309285096 |
| CALCRL | 7.600431913 |
| CALD1 | 10.02644036 |
| CALHM2 | 6.001523821 |
| CALM1 | 11.43958787 |
| CALM2 | 14.3383767 |
| CALM3 | 12.84135552 |
| CALML3 | 6.360485112 |
| CALML4 | 8.462159558 |
| CALML6 | 5.958692029 |
| CALN1 | 7.1780878 |
| CALR | 12.4121266 |
| CALU | 10.31698761 |
| CAMK1 | 8.390361432 |
| CAMK1D | 6.836414815 |
| CAMK1G | 5.788197258 |
| CAMK2B | 7.611168218 |
| CAMK2D | 7.150872505 |
| CAMK2G | 8.624406176 |
| CAMK2N1 | 12.04757238 |
| CAMKK2 | 7.603972964 |
| CAMKV | 7.393095684 |
| CAMLG | 12.36069349 |
| CAMP | 6.391938228 |
| CAMSAP1 | 7.824033668 |
| CAMSAP1L1 | 9.108470875 |
| CAND1 | 8.944015302 |
| CANT1 | 8.64527761 |
| CANX | 9.822398984 |
| CAP1 | 12.71750284 |
| CAP2 | 8.116054913 |
| CAPG | 6.387940386 |
| CAPN1 | 11.17154462 |
| CAPN10 | 5.766349041 |
| CAPN12 | 5.519361683 |
| CAPN2 | 6.484577453 |
| CAPN3 | 5.631085331 |
| CAPN5 | 9.986545794 |
| CAPN6 | 9.007506113 |
| CAPN7 | 7.327939361 |
| CAPNS1 | 9.696644457 |
| CAPRIN1 | 7.373171138 |
| CAPRIN2 | 7.61790416 |
| CAPS | 6.475033154 |
| CAPSL | 5.998087289 |
| CAPZA1 | 8.991645419 |
| CAPZA2 | 12.50067454 |
| CAPZB | 10.76537506 |
| CARD10 | 8.668885403 |
| CARD11 | 6.152443006 |
| CARD6 | 6.946617828 |
| CARD8 | 6.662270984 |
| CARD9 | 6.935761096 |
| CARHSP1 | 13.03780317 |
| CARM1 | 8.574716946 |
| CARS | 7.566325187 |
| CARS2 | 7.389032261 |
| CARTPT | 5.883767064 |
| CASC3 | 7.913831405 |
| CASC4 | 8.803477548 |
| CASC5 | 5.574341742 |
| CASD1 | 8.052858135 |
| CASK | 7.654570168 |
| CASKIN2 | 6.147472191 |
| CASP1 | 8.583139361 |
| CASP10 | 5.862574311 |
| CASP2 | 6.605928152 |
| CASP3 | 8.033767881 |
| CASP4 | 10.02324101 |
| CASP5 | 6.026289364 |
| CASP6 | 7.667236517 |
| CASP7 | 8.778424502 |
| CASP8 | 5.487197695 |
| CASP8AP2 | 5.978490545 |
| CASP9 | 7.464730019 |
| CASQ1 | 5.537051385 |
| CAST | 8.615554348 |
| CASZ1 | 7.411821652 |
| CAT | 12.64543348 |
| CATSPER1 | 5.935748898 |
| CATSPER2 | 6.259032109 |
| CATSPER2P1 | 7.541707589 |
| CAV1 | 6.42431198 |
| CAV2 | 9.46060694 |
| CAV3 | 6.382540929 |
| CBARA1 | 8.798586915 |
| CBFA2T2 | 5.950276946 |
| CBFA2T3 | 6.202050178 |
| CBFB | 10.42684005 |
| CBL | 6.69535112 |
| CBLB | 7.650076283 |
| CBLC | 7.189647094 |
| CBLL1 | 6.218461852 |
| CBLN4 | 5.718400945 |
| CBR1 | 9.24781514 |
| CBR3 | 6.114071922 |
| CBR4 | 9.909166565 |
| CBS | 12.82185008 |
| CBX1 | 7.740693333 |
| CBX2 | 6.096307127 |
| CBX3 | 8.905504179 |
| CBX4 | 8.70567533 |
| CBX5 | 8.558330297 |
| CBX6 | 9.473043051 |
| CBX7 | 8.588590261 |
| CBX8 | 6.033114031 |
| CBY1 | 7.616724637 |
| CC2D1B | 5.862217884 |
| CCAR1 | 10.84539464 |
| CCBE1 | 5.772545433 |
| CCBL1 | 7.716165526 |
| CCBL2 | 7.987450773 |
| CCBP2 | 10.13160062 |
| CCDC101 | 6.466330243 |
| CCDC102A | 6.071942103 |
| CCDC104 | 7.940251254 |
| CCDC107 | 7.70735089 |
| CCDC109A | 7.529326957 |
| CCDC109B | 9.356947657 |
| CCDC111 | 5.769075352 |
| CCDC113 | 6.212539576 |
| CCDC115 | 9.100587551 |
| CCDC117 | 9.905036701 |
| CCDC12 | 10.69220515 |
| CCDC120 | 7.12415941 |
| CCDC121 | 5.656705012 |
| CCDC123 | 5.863687657 |
| CCDC124 | 6.496614241 |
| CCDC127 | 9.05905813 |
| CCDC128 | 8.929198328 |
| CCDC129 | 6.076386904 |
| CCDC130 | 10.02305282 |
| CCDC132 | 7.491218043 |
| CCDC134 | 6.188594761 |
| CCDC135 | 6.93943035 |
| CCDC138 | 6.471711749 |
| CCDC14 | 8.042752101 |
| CCDC148 | 6.495172153 |
| CCDC16 | 7.466720207 |
| CCDC17 | 6.730490874 |
| CCDC19 | 8.312130123 |
| CCDC21 | 6.512388885 |
| CCDC22 | 5.829051292 |
| CCDC23 | 11.96490235 |
| CCDC24 | 6.677135784 |
| CCDC25 | 10.23722621 |
| CCDC28A | 10.94682846 |
| CCDC28B | 5.672435395 |
| CCDC3 | 7.777301281 |
| CCDC32 | 10.00999779 |
| CCDC33 | 5.998241191 |
| CCDC34 | 8.043168609 |
| CCDC4 | 5.783458122 |
| CCDC41 | 7.128261927 |
| CCDC43 | 7.785933724 |
| CCDC44 | 11.60429313 |
| CCDC45 | 8.087662548 |
| CCDC47 | 9.536144402 |
| CCDC48 | 5.993330852 |
| CCDC49 | 7.850490786 |
| CCDC5 | 8.666271328 |
| CCDC50 | 6.09055372 |
| CCDC51 | 8.599488776 |
| CCDC53 | 10.92733641 |
| CCDC55 | 7.920823572 |
| CCDC56 | 6.356984969 |
| CCDC58 | 9.976009054 |
| CCDC59 | 11.0536152 |
| CCDC6 | 10.26363171 |
| CCDC64 | 6.016665467 |
| CCDC66 | 6.26517752 |
| CCDC68 | 6.883690103 |
| CCDC69 | 8.321222001 |
| CCDC7 | 5.945764129 |
| CCDC71 | 8.063871824 |
| CCDC72 | 13.09890079 |
| CCDC76 | 7.913524445 |
| CCDC80 | 5.478760351 |
| CCDC84 | 6.894127767 |
| CCDC85B | 8.454347189 |
| CCDC86 | 6.201166021 |
| CCDC88A | 6.523187761 |
| CCDC90B | 8.546583823 |
| CCDC91 | 8.823490067 |
| CCDC92 | 10.64344731 |
| CCDC97 | 7.482520349 |
| CCL13 | 6.81979311 |
| CCL14 | 11.57071259 |
| CCL15 | 6.821482961 |
| CCL16 | 11.82674393 |
| CCL18 | 5.836073425 |
| CCL19 | 9.268401322 |
| CCL2 | 11.08645475 |
| CCL20 | 10.20175707 |
| CCL21 | 8.257102838 |
| CCL22 | 6.131674741 |
| CCL23 | 8.986395836 |
| CCL25 | 7.356377646 |
| CCL26 | 5.972162383 |
| CCL3 | 8.602461098 |
| CCL3L1 | 6.109536367 |
| CCL3L3 | 6.542100813 |
| CCL4L1 | 9.543638976 |
| CCL5 | 7.587618246 |
| CCL7 | 8.282746763 |
| CCL8 | 8.819896211 |
| CCM2 | 9.60025429 |
| CCNA1 | 5.377916135 |
| CCNA2 | 6.436274854 |
| CCNB1 | 5.735819479 |
| CCNB1IP1 | 8.881948549 |
| CCNB2 | 6.334758751 |
| CCNC | 9.946792395 |
| CCND1 | 12.11289713 |
| CCND2 | 6.366609458 |
| CCND3 | 11.31384171 |
| CCNDBP1 | 8.944497065 |
| CCNE2 | 7.070863468 |
| CCNG1 | 11.75167814 |
| CCNG2 | 5.564964458 |
| CCNH | 9.556418601 |
| CCNI | 13.0937915 |
| CCNJ | 5.611349986 |
| CCNK | 9.973965062 |
| CCNL1 | 6.137116661 |
| CCNT1 | 6.231448289 |
| CCNT2 | 7.020475303 |
| CCNY | 9.857345564 |
| CCNYL1 | 7.783357174 |
| CCPG1 | 10.39748632 |
| CCR1 | 7.571588869 |
| CCR10 | 5.557097926 |
| CCR2 | 6.706170503 |
| CCR6 | 6.052250082 |
| CCR7 | 5.773552543 |
| CCR9 | 5.985445173 |
| CCRL1 | 5.703597081 |
| CCRN4L | 9.139196833 |
| CCS | 8.462021862 |
| CCT2 | 12.34317875 |
| CCT3 | 10.36705777 |
| CCT4 | 8.072167832 |
| CCT5 | 8.220373554 |
| CCT6A | 9.445914679 |
| CCT6B | 8.142848125 |
| CCT7 | 10.13509555 |
| CCT8 | 12.01471765 |
| CCT8L2 | 6.991353377 |
| CD14 | 13.18253956 |
| CD151 | 8.396845837 |
| CD160 | 7.038581343 |
| CD163 | 10.56163756 |
| CD164 | 9.340402554 |
| CD1C | 5.731506979 |
| CD1D | 8.732254002 |
| CD1E | 5.71555027 |
| CD2 | 6.869776826 |
| CD200 | 6.353230495 |
| CD200R1 | 5.603790492 |
| CD209 | 8.105625041 |
| CD24 | 6.677800373 |
| CD244 | 5.998816862 |
| CD247 | 8.304334484 |
| CD248 | 5.877475986 |
| CD2AP | 7.560362399 |
| CD2BP2 | 10.02201425 |
| CD300A | 7.835569068 |
| CD300C | 6.585774324 |
| CD300LF | 6.852152563 |
| CD300LG | 6.027386339 |
| CD302 | 11.51137283 |
| CD320 | 5.968337754 |
| CD33 | 8.068603609 |
| CD36 | 10.13726715 |
| CD37 | 6.203759179 |
| CD38 | 9.817712277 |
| CD3EAP | 5.730838055 |
| CD4 | 6.865622108 |
| CD40 | 7.114694387 |
| CD44 | 11.42202887 |
| CD46 | 11.38674848 |
| CD47 | 9.252327222 |
| CD48 | 9.294890096 |
| CD52 | 6.14951648 |
| CD53 | 8.209694411 |
| CD55 | 10.2575818 |
| CD58 | 9.164965926 |
| CD59 | 9.149934789 |
| CD5L | 8.436321993 |
| CD6 | 5.835525267 |
| CD63 | 11.95412442 |
| CD68 | 13.17176559 |
| CD69 | 6.34436306 |
| CD7 | 6.857169567 |
| CD72 | 7.130354136 |
| CD74 | 9.274726532 |
| CD79A | 6.004057394 |
| CD79B | 7.012035192 |
| CD80 | 6.140610891 |
| CD81 | 14.84614901 |
| CD82 | 7.975930287 |
| CD83 | 7.395624764 |
| CD84 | 6.359250452 |
| CD86 | 7.716436051 |
| CD8A | 6.314412587 |
| CD9 | 12.13618023 |
| CD96 | 6.002826198 |
| CD97 | 10.1623934 |
| CD99 | 11.48275159 |
| CD99L2 | 9.889622551 |
| CDA | 8.93084998 |
| CDA08 | 10.39230213 |
| CDAN1 | 6.986229037 |
| CDC123 | 10.76357224 |
| CDC14A | 5.848581194 |
| CDC14B | 8.712001944 |
| CDC14C | 6.427261992 |
| CDC16 | 10.61184624 |
| CDC2 | 6.124262258 |
| CDC20 | 7.647584608 |
| CDC23 | 10.74976922 |
| CDC25A | 6.247166121 |
| CDC25B | 7.385345876 |
| CDC26 | 10.27304734 |
| CDC27 | 7.427531997 |
| CDC2L1 | 5.839407831 |
| CDC2L2 | 7.05270671 |
| CDC2L5 | 8.736538325 |
| CDC2L6 | 8.508045499 |
| CDC34 | 9.15421416 |
| CDC37 | 12.84057685 |
| CDC37L1 | 11.75114644 |
| CDC40 | 9.804272773 |
| CDC42 | 8.391143731 |
| CDC42BPA | 6.671242378 |
| CDC42BPB | 7.308662547 |
| CDC42EP1 | 8.82988744 |
| CDC42EP2 | 6.591784092 |
| CDC42EP3 | 5.50380927 |
| CDC42EP4 | 11.10978462 |
| CDC42EP5 | 7.202900481 |
| CDC42SE1 | 6.135098389 |
| CDC42SE2 | 7.088917286 |
| CDC45L | 5.709182092 |
| CDC5L | 8.237524721 |
| CDC7 | 7.215095526 |
| CDCA4 | 6.574855528 |
| CDCA5 | 6.909729045 |
| CDCA7 | 5.479217561 |
| CDCA7L | 8.420496467 |
| CDCA8 | 5.77285282 |
| CDCP1 | 5.695654516 |
| CDGAP | 5.838518571 |
| CDH1 | 6.208228618 |
| CDH11 | 5.969868156 |
| CDH13 | 5.633217753 |
| CDH15 | 7.144547499 |
| CDH19 | 6.611365884 |
| CDH2 | 11.9157455 |
| CDH23 | 6.894452795 |
| CDH24 | 5.620652312 |
| CDH26 | 5.793384408 |
| CDH5 | 8.46896945 |
| CDH6 | 5.864953239 |
| CDH7 | 5.881133457 |
| CDH9 | 8.126062458 |
| CDIPT | 7.234363197 |
| CDK10 | 7.463429576 |
| CDK2 | 7.392589705 |
| CDK2AP1 | 12.880447 |
| CDK2AP2 | 8.460443883 |
| CDK4 | 11.02479776 |
| CDK5 | 8.542963386 |
| CDK5R1 | 6.407318626 |
| CDK5RAP1 | 7.699673761 |
| CDK5RAP2 | 9.411411061 |
| CDK5RAP3 | 10.09422799 |
| CDK6 | 8.72106874 |
| CDK7 | 9.994439955 |
| CDK8 | 6.089245781 |
| CDK9 | 9.94100208 |
| CDKAL1 | 6.050369568 |
| CDKL3 | 5.978137255 |
| CDKL4 | 5.816849833 |
| CDKN1A | 12.52121627 |
| CDKN1B | 9.593812347 |
| CDKN1C | 5.585561512 |
| CDKN2A | 5.621146356 |
| CDKN2AIP | 8.598970789 |
| CDKN2B | 5.480568744 |
| CDKN2C | 6.380823167 |
| CDKN2D | 7.688000506 |
| CDKN3 | 6.683176732 |
| CDO1 | 12.07760678 |
| CDR2 | 9.309575416 |
| CDR2L | 6.186983769 |
| CDS1 | 7.08374545 |
| CDS2 | 9.299874318 |
| CDT1 | 6.282237437 |
| CDV3 | 9.547591841 |
| CDY2B | 5.663629093 |
| CDYL | 6.246824505 |
| CDYL2 | 5.576144405 |
| CEACAM1 | 9.999551247 |
| CEACAM21 | 6.246533397 |
| CEACAM4 | 5.713062581 |
| CEACAM6 | 6.974835881 |
| CEACAM8 | 5.845175886 |
| CEBPA | 10.81873745 |
| CEBPB | 13.71579036 |
| CEBPD | 13.05558311 |
| CEBPG | 10.85218587 |
| CEBPZ | 11.39354897 |
| CECR1 | 7.189857172 |
| CECR5 | 11.22100354 |
| CECR7 | 7.736044664 |
| CEL | 5.419690978 |
| CENPB | 11.66539811 |
| CENPC1 | 6.32917573 |
| CENPH | 5.891717872 |
| CENPL | 6.059448808 |
| CENPM | 6.654813954 |
| CENPN | 8.830515191 |
| CENPO | 5.810546376 |
| CENPP | 6.106893026 |
| CENPQ | 6.874953041 |
| CENTA2 | 10.26400806 |
| CENTB2 | 7.16133562 |
| CENTD1 | 5.924344253 |
| CENTD2 | 7.840290381 |
| CENTD3 | 8.340659947 |
| CENTG2 | 6.210267062 |
| CENTG3 | 7.8968462 |
| CEP1 | 6.011008623 |
| CEP135 | 7.300907398 |
| CEP164 | 6.144908357 |
| CEP192 | 7.468047009 |
| CEP27 | 9.139178169 |
| CEP290 | 5.741511039 |
| CEP350 | 9.30264539 |
| CEP55 | 5.745338694 |
| CEP57 | 7.56811885 |
| CEP63 | 9.915202251 |
| CEP70 | 8.355370196 |
| CEP72 | 5.652833541 |
| CEP76 | 6.863764568 |
| CEPT1 | 7.868969577 |
| CERK | 9.080556536 |
| CES1 | 8.575323473 |
| CES2 | 12.5664874 |
| CES7 | 6.9780998 |
| CETN1 | 5.458635921 |
| CETN2 | 11.62275116 |
| CETN3 | 5.737855139 |
| CETP | 7.611158206 |
| CFB | 15.07251477 |
| CFD | 10.15146401 |
| CFDP1 | 9.492048861 |
| CFH | 12.24608567 |
| CFHR1 | 13.27266913 |
| CFHR2 | 11.67346372 |
| CFHR3 | 10.87537593 |
| CFHR4 | 10.66068073 |
| CFHR5 | 10.17139617 |
| CFI | 13.66902927 |
| CFL1 | 12.41816628 |
| CFL2 | 6.943059291 |
| CFLAR | 10.21026326 |
| CFP | 7.04224118 |
| CFTR | 6.714929839 |
| CG018 | 8.870100655 |
| CGA | 5.870489053 |
| CGB | 5.563295093 |
| CGB7 | 5.819412152 |
| CGGBP1 | 11.06221308 |
| CGI-115 | 9.92310628 |
| CGN | 10.73072724 |
| CGNL1 | 11.59738667 |
| CGRRF1 | 9.587570931 |
| CH25H | 7.427856347 |
| CHAC1 | 5.726206056 |
| CHAC2 | 7.437886117 |
| CHAD | 6.410298101 |
| CHAF1A | 6.119864526 |
| CHAF1B | 6.575740606 |
| CHCHD1 | 9.090588282 |
| CHCHD10 | 12.81560194 |
| CHCHD2 | 14.84081304 |
| CHCHD3 | 10.15761118 |
| CHCHD4 | 10.52460292 |
| CHCHD5 | 10.51266609 |
| CHCHD6 | 7.065336691 |
| CHCHD7 | 9.39165811 |
| CHCHD8 | 9.721884511 |
| CHD1 | 9.226424247 |
| CHD1L | 8.057541731 |
| CHD4 | 8.472650502 |
| CHD6 | 6.285171078 |
| CHD7 | 5.801511528 |
| CHD8 | 9.576150243 |
| CHD9 | 10.26075982 |
| CHDH | 5.835072017 |
| CHEK1 | 5.830574807 |
| CHEK2 | 6.228226591 |
| CHERP | 6.453162684 |
| CHFR | 8.654879267 |
| CHIC2 | 11.24844805 |
| CHID1 | 5.926110035 |
| CHIT1 | 5.898510973 |
| CHKA | 7.258098746 |
| CHKB | 6.71556742 |
| CHM | 5.389379896 |
| CHMP1B | 10.07367914 |
| CHMP2A | 9.996311337 |
| CHMP2B | 10.62773466 |
| CHMP4A | 9.048712087 |
| CHMP4B | 9.292257104 |
| CHMP4C | 6.977313348 |
| CHMP5 | 11.0194274 |
| CHMP6 | 7.631777487 |
| CHMP7 | 8.239193998 |
| CHN1 | 7.059701822 |
| CHN2 | 8.1131614 |
| CHODL | 5.766159687 |
| CHORDC1 | 8.078547319 |
| CHP | 11.90836132 |
| CHPF | 7.963468053 |
| CHPT1 | 11.63703398 |
| CHRAC1 | 6.350457558 |
| CHRD | 5.880478023 |
| CHRDL2 | 9.377288797 |
| CHRM2 | 5.881264004 |
| CHRM3 | 5.528957615 |
| CHRNA10 | 5.771854413 |
| CHRNA5 | 5.609270616 |
| CHRNA6 | 5.755981925 |
| CHRNB1 | 8.041003725 |
| CHRNB2 | 6.22817809 |
| CHRNB3 | 7.354393141 |
| CHST1 | 5.581055516 |
| CHST10 | 5.789403813 |
| CHST11 | 6.591284536 |
| CHST12 | 6.250484742 |
| CHST13 | 11.63962841 |
| CHST14 | 6.263691097 |
| CHST2 | 5.826850138 |
| CHST3 | 5.782712591 |
| CHST4 | 10.01803119 |
| CHST7 | 9.333500433 |
| CHST9 | 6.437072959 |
| CHSY1 | 9.89770135 |
| CHSY3 | 9.717098006 |
| CHUK | 10.03763561 |
| CHURC1 | 10.94065632 |
| CHX10 | 5.54457998 |
| CIAO1 | 8.845398569 |
| CIAPIN1 | 8.444926314 |
| CIB1 | 11.8855997 |
| CIB2 | 5.648935368 |
| CIC | 7.30027125 |
| CICE | 10.47442038 |
| CIDEA | 5.773336184 |
| CIDEB | 11.53375504 |
| CIDEC | 6.747794818 |
| CILP | 5.682858839 |
| CINP | 7.769035142 |
| CIP29 | 11.43651579 |
| CIR | 7.896391953 |
| CIRBP | 11.92670287 |
| CIRH1A | 10.3118535 |
| CISD1 | 10.14085438 |
| CISD2 | 10.51312041 |
| CISH | 7.52013942 |
| CIT | 6.045366153 |
| CITED2 | 8.686341616 |
| CITED4 | 8.516540715 |
| CKAP2 | 5.704931133 |
| CKAP2L | 5.946023793 |
| CKAP4 | 10.59836376 |
| CKAP5 | 7.036306169 |
| CKB | 6.207554564 |
| CKLF | 11.50287107 |
| CKS1B | 12.15814469 |
| CKS2 | 9.325732793 |
| CLASP1 | 8.440443294 |
| CLASP2 | 6.43099534 |
| CLC | 7.076363452 |
| CLCA2 | 6.073526859 |
| CLCC1 | 9.157853661 |
| CLCF1 | 6.323340133 |
| CLCN3 | 9.19431337 |
| CLCN5 | 6.099217298 |
| CLCN6 | 6.488959672 |
| CLCN7 | 11.2701281 |
| CLDN1 | 13.17012205 |
| CLDN10 | 7.413743355 |
| CLDN12 | 11.2025359 |
| CLDN14 | 8.616142528 |
| CLDN15 | 7.043539674 |
| CLDN2 | 9.304439161 |
| CLDN23 | 10.27182968 |
| CLDN3 | 9.79396831 |
| CLDN5 | 9.716554367 |
| CLDN7 | 9.529374492 |
| CLDN9 | 5.609607158 |
| CLDND1 | 10.33156893 |
| CLDND2 | 5.963206658 |
| CLEC11A | 6.042883763 |
| CLEC12A | 7.161991582 |
| CLEC14A | 6.827422168 |
| CLEC16A | 9.343417468 |
| CLEC1A | 6.608373952 |
| CLEC1B | 12.26172661 |
| CLEC2B | 6.92819816 |
| CLEC2D | 8.619903662 |
| CLEC3B | 9.117840323 |
| CLEC4A | 6.995068825 |
| CLECL1 | 6.624236271 |
| CLGN | 5.864328081 |
| CLIC1 | 8.578893952 |
| CLIC2 | 6.786583834 |
| CLIC4 | 7.01934366 |
| CLIC6 | 6.236855421 |
| CLINT1 | 11.08928818 |
| CLIP1 | 6.121832813 |
| CLIP3 | 6.148722002 |
| CLIP4 | 5.546513937 |
| CLK1 | 8.816318774 |
| CLK2 | 6.38051275 |
| CLK3 | 7.23850901 |
| CLK4 | 6.300415284 |
| CLMN | 7.542836062 |
| CLN3 | 8.12275088 |
| CLN5 | 9.328344043 |
| CLN6 | 6.079353111 |
| CLN8 | 5.755675237 |
| CLNS1A | 12.84504946 |
| CLOCK | 7.407459785 |
| CLP1 | 8.625673497 |
| CLPP | 10.30939823 |
| CLPS | 5.659357789 |
| CLPTM1 | 9.907778296 |
| CLPTM1L | 11.6995063 |
| CLPX | 10.23449255 |
| CLRN1 | 8.387204721 |
| CLRN3 | 9.032818277 |
| CLSTN1 | 5.682703806 |
| CLSTN3 | 5.747193983 |
| CLTA | 10.47887291 |
| CLTB | 11.47154844 |
| CLTC | 8.704077738 |
| CLTCL1 | 6.290803819 |
| CLU | 6.260027163 |
| CLUAP1 | 6.585523934 |
| CLUL1 | 6.272381892 |
| CLYBL | 10.90570005 |
| CMAH | 6.269691948 |
| CMAS | 10.66477404 |
| CMC1 | 10.03161586 |
| CMIP | 10.52244833 |
| CMKLR1 | 5.91143828 |
| CMKOR1 | 9.987211093 |
| CML2 | 10.37653393 |
| CMPK1 | 12.19042705 |
| CMTM1 | 5.726603006 |
| CMTM2 | 6.338317382 |
| CMTM3 | 7.78027069 |
| CMTM4 | 7.807141323 |
| CMTM5 | 6.107489447 |
| CMTM6 | 12.09134293 |
| CMTM7 | 8.269847112 |
| CMTM8 | 11.77172137 |
| CMYA5 | 5.922928465 |
| CNBP | 12.77724832 |
| CNDP1 | 7.569918258 |
| CNDP2 | 13.78332069 |
| CNGA1 | 8.0584904 |
| CNIH | 11.98736267 |
| CNIH2 | 5.878257014 |
| CNIH3 | 5.552711955 |
| CNIH4 | 12.74929032 |
| CNKSR3 | 10.00743972 |
| CNN2 | 7.561576726 |
| CNN3 | 12.5114597 |
| CNNM2 | 5.994722503 |
| CNNM3 | 9.133850606 |
| CNNM4 | 6.19440082 |
| CNO | 10.30440206 |
| CNOT1 | 10.92948512 |
| CNOT10 | 9.243017014 |
| CNOT2 | 10.29934519 |
| CNOT3 | 6.380664689 |
| CNOT4 | 7.281227292 |
| CNOT6 | 6.820897778 |
| CNOT7 | 8.868973258 |
| CNOT8 | 7.885624868 |
| CNPY4 | 6.549331651 |
| CNR2 | 5.725837194 |
| CNTFR | 6.144572485 |
| CNTN3 | 5.670164002 |
| CNTN4 | 5.795810502 |
| CNTNAP1 | 5.65093516 |
| CNTNAP2 | 8.96133557 |
| CNTNAP3 | 5.957891739 |
| CNTNAP3B | 6.185254092 |
| CNTNAP5 | 5.661526585 |
| COASY | 11.89083739 |
| COBL | 9.30048475 |
| COBLL1 | 11.89277636 |
| COBRA1 | 9.157451404 |
| COCH | 5.909176994 |
| COG1 | 5.846643427 |
| COG2 | 8.557590639 |
| COG3 | 9.12603432 |
| COG4 | 7.223726195 |
| COG5 | 5.822904424 |
| COG6 | 9.0252115 |
| COG7 | 6.763388789 |
| COG8 | 6.272896371 |
| COIL | 9.252301731 |
| COL11A1 | 5.82050117 |
| COL11A2 | 5.686545394 |
| COL12A1 | 5.992103739 |
| COL14A1 | 5.937485691 |
| COL16A1 | 6.076359535 |
| COL17A1 | 6.166385261 |
| COL18A1 | 11.16781304 |
| COL1A1 | 7.23774348 |
| COL1A2 | 7.600312962 |
| COL24A1 | 5.60963006 |
| COL25A1 | 5.795548261 |
| COL3A1 | 9.344049562 |
| COL4A1 | 10.56191811 |
| COL4A2 | 6.554786412 |
| COL4A3BP | 8.066522497 |
| COL4A5 | 6.118174564 |
| COL5A1 | 7.879018984 |
| COL5A2 | 7.970998672 |
| COL6A1 | 7.842135603 |
| COL6A2 | 5.927446684 |
| COL6A3 | 7.834454366 |
| COL8A1 | 6.252056 |
| COL8A2 | 7.993795543 |
| COLEC10 | 7.499281484 |
| COLEC11 | 11.79900419 |
| COLEC12 | 5.713978979 |
| COLQ | 5.658876441 |
| COMMD1 | 11.94678257 |
| COMMD10 | 10.34056704 |
| COMMD2 | 10.12496198 |
| COMMD3 | 12.38769278 |
| COMMD6 | 12.98796682 |
| COMMD7 | 11.63305142 |
| COMMD8 | 10.06574217 |
| COMMD9 | 10.0778125 |
| COMT | 10.21991995 |
| COMTD1 | 9.150504104 |
| COP1 | 6.146187901 |
| COPA | 11.32960393 |
| COPB1 | 11.95721479 |
| COPB2 | 11.15515995 |
| COPE | 10.85080029 |
| COPG | 9.872352302 |
| COPG2 | 6.752695867 |
| COPS2 | 7.814536835 |
| COPS3 | 11.88594875 |
| COPS4 | 10.21190077 |
| COPS5 | 12.03421398 |
| COPS6 | 9.817862492 |
| COPS7A | 11.98520352 |
| COPS7B | 8.128462475 |
| COPS8 | 8.236212826 |
| COPZ1 | 10.83334402 |
| COPZ2 | 7.515148429 |
| COQ10A | 9.076827766 |
| COQ10B | 11.93946305 |
| COQ2 | 11.12801537 |
| COQ3 | 10.44831391 |
| COQ4 | 9.371984969 |
| COQ5 | 11.62309795 |
| COQ6 | 8.554409089 |
| COQ7 | 6.364658197 |
| COQ9 | 10.60927504 |
| CORO1A | 8.469954768 |
| CORO1B | 9.513898058 |
| CORO1C | 9.676251127 |
| CORO2A | 7.054146144 |
| CORO7 | 6.412180702 |
| CORT | 5.846576271 |
| COTL1 | 7.894926152 |
| COX10 | 6.147096166 |
| COX11P | 6.084745574 |
| COX15 | 8.316352459 |
| COX17 | 13.13488438 |
| COX4I1 | 14.81357878 |
| COX4NB | 8.194967429 |
| COX5A | 13.8265207 |
| COX5B | 14.11709006 |
| COX6A1 | 13.05903745 |
| COX6A2 | 6.077913008 |
| COX6B1 | 13.75378741 |
| COX6C | 13.77248411 |
| COX7A1 | 9.094242187 |
| COX7A2 | 14.6428157 |
| COX7A2L | 12.43169249 |
| COX7B | 13.09370965 |
| COX7C | 14.79712889 |
| COX8A | 13.94275302 |
| CP | 14.07096698 |
| CP110 | 8.969145409 |
| CPA1 | 5.63931874 |
| CPA2 | 5.576089228 |
| CPA3 | 6.104839453 |
| CPB1 | 6.321324646 |
| CPB2 | 13.75411746 |
| CPD | 11.04859515 |
| CPE | 8.693058821 |
| CPEB2 | 7.987212719 |
| CPEB3 | 10.03987895 |
| CPEB4 | 7.78115153 |
| CPLX1 | 6.800863945 |
| CPLX2 | 5.929658363 |
| CPM | 7.502463062 |
| CPN1 | 12.34056463 |
| CPN2 | 13.28154467 |
| CPNE1 | 7.463259189 |
| CPNE3 | 11.6588836 |
| CPNE8 | 8.895822158 |
| CPNE9 | 5.782666143 |
| CPOX | 10.75383559 |
| CPS1 | 13.40244016 |
| CPSF1 | 9.34414066 |
| CPSF2 | 8.229499892 |
| CPSF3 | 10.42867224 |
| CPSF3L | 7.951210341 |
| CPSF4 | 10.84167323 |
| CPSF6 | 5.611077931 |
| CPT1A | 6.236116034 |
| CPT1C | 5.875545401 |
| CPT2 | 11.14030288 |
| CPVL | 9.673994018 |
| CPXM1 | 5.694637986 |
| CPXM2 | 6.32419663 |
| CR1 | 5.602943917 |
| CRADD | 11.26315944 |
| CRAMP1L | 5.90744844 |
| CRAT | 9.059220056 |
| CRB2 | 7.931906734 |
| CRB3 | 7.722955481 |
| CRBN | 10.0398231 |
| CREB1 | 6.872295474 |
| CREB3 | 8.069403306 |
| CREB3L2 | 9.737214626 |
| CREB3L3 | 12.78135027 |
| CREB3L4 | 6.411886037 |
| CREB5 | 6.421945246 |
| CREBBP | 8.819013137 |
| CREBL1 | 7.545676487 |
| CREBL2 | 7.947955563 |
| CREG1 | 11.90410103 |
| CRELD1 | 7.978285349 |
| CRELD2 | 12.34434853 |
| CREM | 7.481426552 |
| CRHBP | 10.9839983 |
| CRHR1 | 6.336240805 |
| CRIM1 | 6.116629981 |
| CRIP1 | 8.813065857 |
| CRIP2 | 8.650521617 |
| CRIP3 | 6.020111263 |
| CRIPAK | 6.73813207 |
| CRIPT | 9.77345127 |
| CRISPLD2 | 9.426065304 |
| CRK | 8.539399627 |
| CRKL | 11.20778255 |
| CRKRS | 7.52094434 |
| CRLF1 | 6.744716397 |
| CRLF3 | 6.43765301 |
| CRLS1 | 12.98880355 |
| CRMP1 | 5.528478428 |
| CRNKL1 | 6.484492105 |
| CROCC | 5.642762691 |
| CROP | 5.483953934 |
| CROT | 9.075330715 |
| CRP | 15.21001097 |
| CRSP2 | 5.843241837 |
| CRSP8 | 7.144667093 |
| CRSP9 | 8.589545295 |
| CRTAP | 7.625612906 |
| CRTC2 | 6.346271919 |
| CRX | 5.781151553 |
| CRY1 | 8.290825298 |
| CRY2 | 9.389381329 |
| CRYAA | 9.031064786 |
| CRYAB | 6.636948758 |
| CRYBB2 | 5.954323959 |
| CRYBB3 | 5.363239152 |
| CRYGB | 5.570860332 |
| CRYL1 | 11.68761983 |
| CRYM | 6.914189029 |
| CRYZ | 13.6469269 |
| CRYZL1 | 7.501854982 |
| CS | 11.56201184 |
| CSAD | 8.242038262 |
| CSAG1 | 5.496285399 |
| CSAG3A | 9.265713943 |
| CSDA | 11.72632506 |
| CSDE1 | 6.796773429 |
| CSE1L | 9.05148868 |
| CSEN | 5.985650532 |
| CSF1R | 11.54229803 |
| CSF2RA | 7.45233506 |
| CSF3 | 5.98307057 |
| CSGALNACT2 | 5.733285981 |
| CSGLCA-T | 7.216611716 |
| CSH1 | 6.255641384 |
| CSK | 10.18555587 |
| CSMD1 | 6.037587098 |
| CSNK1A1 | 7.518935961 |
| CSNK1A1L | 6.019139348 |
| CSNK1D | 8.592245098 |
| CSNK1E | 7.557893083 |
| CSNK1G1 | 7.5190626 |
| CSNK1G2 | 6.701186695 |
| CSNK1G3 | 8.276325673 |
| CSNK2A1 | 7.634295462 |
| CSNK2A1P | 6.061164673 |
| CSNK2A2 | 8.749900894 |
| CSNK2B | 11.88120119 |
| CSPG4 | 5.728801671 |
| CSPG5 | 5.688697859 |
| CSRP1 | 9.558026494 |
| CSRP2 | 10.87358366 |
| CSRP2BP | 9.15182565 |
| CST3 | 13.8314661 |
| CST6 | 6.290188322 |
| CST7 | 7.680799887 |
| CSTA | 5.882857112 |
| CSTB | 13.21531754 |
| CSTF1 | 6.200793197 |
| CSTF2 | 8.618334793 |
| CSTF2T | 7.585493782 |
| CSTF3 | 9.88149227 |
| CT45-2 | 6.194340015 |
| CTAGE5 | 5.731415937 |
| CTAGE6 | 5.680833497 |
| CTBP1 | 7.926741057 |
| CTBS | 11.55136646 |
| CTCF | 8.976749893 |
| CTDP1 | 6.862418649 |
| CTDSP1 | 7.728538915 |
| CTDSP2 | 10.57762548 |
| CTDSPL | 9.245085983 |
| CTDSPL2 | 7.036060264 |
| CTGF | 11.32914292 |
| CTH | 9.729950653 |
| CTHRC1 | 6.666337587 |
| CTLA4 | 6.64313823 |
| CTNNA1 | 11.64631862 |
| CTNNA3 | 5.571842915 |
| CTNNAL1 | 10.3030955 |
| CTNNB1 | 6.75322919 |
| CTNNBIP1 | 6.872299786 |
| CTNNBL1 | 9.823253658 |
| CTNND1 | 5.665689555 |
| CTNS | 7.647336489 |
| CTPS | 9.993380003 |
| CTPS2 | 8.051423959 |
| CTR9 | 8.275122499 |
| CTRB2 | 5.849139283 |
| CTRC | 5.502838279 |
| CTSB | 11.54601371 |
| CTSC | 9.390527641 |
| CTSD | 13.16274647 |
| CTSE | 5.908837258 |
| CTSF | 8.285852944 |
| CTSG | 8.826443646 |
| CTSH | 8.371044669 |
| CTSK | 6.888944314 |
| CTSL | 11.19760948 |
| CTSL2 | 6.00513077 |
| CTSO | 10.40081921 |
| CTSS | 8.737034484 |
| CTSW | 6.158020826 |
| CTSZ | 10.65043187 |
| CTTN | 6.886845963 |
| CTTNBP2NL | 5.875310913 |
| CTXN1 | 5.738690851 |
| CUEDC1 | 8.82455726 |
| CUEDC2 | 9.696793114 |
| CUGBP1 | 7.042442111 |
| CUGBP2 | 5.733202836 |
| CUL1 | 10.26155615 |
| CUL2 | 10.01474936 |
| CUL4A | 10.12084495 |
| CUL4B | 6.429660862 |
| CUL5 | 9.48587793 |
| CUTA | 12.00792258 |
| CUTC | 9.139610337 |
| CUTL1 | 6.044970307 |
| CUTL2 | 9.156132243 |
| CUX1 | 5.398751134 |
| CWF19L1 | 7.074732285 |
| CWF19L2 | 8.274836319 |
| CX3CL1 | 8.195722144 |
| CX3CR1 | 5.98487504 |
| CXADR | 10.9986804 |
| CXCL1 | 9.183864406 |
| CXCL10 | 9.242744763 |
| CXCL11 | 6.733014558 |
| CXCL12 | 9.51401791 |
| CXCL13 | 6.268320334 |
| CXCL14 | 7.853477761 |
| CXCL16 | 11.72631449 |
| CXCL2 | 10.92654569 |
| CXCL3 | 6.788659583 |
| CXCL6 | 8.312307535 |
| CXCL9 | 6.216475387 |
| CXCR3 | 5.8323789 |
| CXCR4 | 7.707012142 |
| CXCR5 | 5.820394366 |
| CXCR6 | 5.744723818 |
| CXorf12 | 9.606728053 |
| CXorf15 | 5.450892008 |
| CXorf21 | 6.27485759 |
| CXorf23 | 6.397806752 |
| CXorf26 | 11.07217264 |
| CXorf33 | 7.74644018 |
| CXorf34 | 6.035465421 |
| CXorf38 | 8.697502339 |
| CXorf39 | 8.125858177 |
| CXorf40A | 6.092340804 |
| CXorf40B | 5.599860218 |
| CXorf42 | 6.096395261 |
| CXorf43 | 6.181550778 |
| CXorf45 | 5.449673543 |
| CXorf56 | 7.229719771 |
| CXorf57 | 6.747789711 |
| CXorf59 | 5.812409476 |
| CXorf6 | 6.94049106 |
| CXorf61 | 5.78409622 |
| CXX1 | 9.299183203 |
| CXXC1 | 10.09540706 |
| CXXC4 | 6.102097075 |
| CXXC5 | 11.552726 |
| CYB561 | 8.621811497 |
| CYB561D1 | 8.901759647 |
| CYB561D2 | 9.652236648 |
| CYB5A | 15.00469574 |
| CYB5D1 | 8.582750841 |
| CYB5D2 | 9.380868086 |
| CYB5-M | 11.86853209 |
| CYB5R1 | 10.79581906 |
| CYB5R2 | 8.017256935 |
| CYB5R3 | 9.749564987 |
| CYB5R4 | 8.999670512 |
| CYBA | 11.55871503 |
| CYBASC3 | 8.793982893 |
| CYBB | 8.942234773 |
| CYBRD1 | 8.667716677 |
| CYC1 | 12.47663349 |
| CYCS | 8.411243414 |
| CYCSL1 | 11.08699656 |
| CYFIP1 | 7.690342642 |
| CYFIP2 | 9.285725111 |
| CYGB | 7.735935958 |
| CYHR1 | 6.239669192 |
| CYLC1 | 5.90229343 |
| CYLD | 7.702351753 |
| CYLN2 | 8.505979337 |
| CYorf14 | 6.58022368 |
| CYorf15A | 9.375041555 |
| CYP11A1 | 6.886332292 |
| CYP1A1 | 8.444522963 |
| CYP1A2 | 9.427076083 |
| CYP1B1 | 9.145682226 |
| CYP20A1 | 10.13962822 |
| CYP21A2 | 7.813610947 |
| CYP26A1 | 6.993774788 |
| CYP26B1 | 5.853735695 |
| CYP27A1 | 12.94737564 |
| CYP2A13 | 5.671024787 |
| CYP2A6 | 8.926947335 |
| CYP2A7 | 7.665662452 |
| CYP2C18 | 11.98140321 |
| CYP2C19 | 7.282994071 |
| CYP2C8 | 13.77043748 |
| CYP2C9 | 13.139476 |
| CYP2D6 | 8.231559399 |
| CYP2E1 | 15.21665198 |
| CYP2J2 | 12.46794556 |
| CYP2R1 | 9.746211404 |
| CYP2S1 | 5.611420696 |
| CYP2U1 | 7.096345779 |
| CYP39A1 | 9.406361992 |
| CYP3A4 | 11.01532358 |
| CYP3A43 | 6.123104206 |
| CYP3A5 | 10.64941304 |
| CYP3A7 | 7.797261758 |
| CYP4A11 | 13.52434819 |
| CYP4A22 | 6.235724751 |
| CYP4F11 | 11.64665104 |
| CYP4F12 | 9.076268662 |
| CYP4F2 | 7.686405861 |
| CYP4F3 | 11.42086931 |
| CYP4V2 | 10.64123715 |
| CYP4X1 | 6.404852447 |
| CYP51A1 | 10.42233217 |
| CYP7A1 | 5.580450754 |
| CYP7B1 | 6.239659637 |
| CYP8B1 | 12.92843599 |
| CYR61 | 9.380760311 |
| CYSLTR1 | 6.008058269 |
| CYYR1 | 8.41938546 |
| DAAM1 | 9.337684235 |
| DAAM2 | 5.728511646 |
| DAB2 | 9.981531164 |
| DAB2IP | 5.864231253 |
| DACH1 | 7.599227863 |
| DACT1 | 6.585151061 |
| DACT2 | 5.745743153 |
| DACT3 | 6.397505524 |
| DAD1 | 13.79776639 |
| DAG1 | 9.696956545 |
| DAGLB | 9.049416103 |
| DAK | 8.336367807 |
| DALRD3 | 5.543611976 |
| DAO | 8.126866926 |
| DAP | 13.3739976 |
| DAP3 | 8.94376439 |
| DAPK1 | 8.039970095 |
| DAPK2 | 6.147349783 |
| DAPK3 | 6.513279264 |
| DARC | 7.734496909 |
| DARS | 13.17205227 |
| DARS2 | 8.448551703 |
| DAXX | 6.470063755 |
| DAZAP1 | 8.61228653 |
| DAZAP2 | 13.21179711 |
| DBF4B | 5.761039425 |
| DBH | 9.654209156 |
| DBI | 14.55790599 |
| DBN1 | 7.377523216 |
| DBNL | 11.56309809 |
| DBP | 5.667776423 |
| DBR1 | 6.149117623 |
| DBT | 11.36275639 |
| DC2 | 13.47002127 |
| DCAKD | 10.22472277 |
| DCAMKL3 | 5.881593482 |
| DCBLD1 | 7.925074448 |
| DCBLD2 | 8.044754361 |
| DCDC2 | 5.720384001 |
| DCHS1 | 6.094421552 |
| DCI | 12.51364042 |
| DCK | 8.302413016 |
| DCLRE1A | 8.181488678 |
| DCLRE1B | 5.810764433 |
| DCLRE1C | 7.447704988 |
| DCN | 10.57773942 |
| DCP1A | 7.817128509 |
| DCP1B | 5.861721792 |
| DCP2 | 8.785365955 |
| DCPS | 8.847449218 |
| DCTD | 10.80328088 |
| DCTN1 | 5.763116741 |
| DCTN2 | 11.83119214 |
| DCTN3 | 11.23914272 |
| DCTN4 | 9.484885944 |
| DCTN5 | 9.875506292 |
| DCTN6 | 10.89361337 |
| DC-UbP | 7.087463905 |
| DCUN1D1 | 8.540614208 |
| DCUN1D3 | 10.16476104 |
| DCUN1D4 | 7.643369178 |
| DCUN1D5 | 10.42563705 |
| DCXR | 12.75008022 |
| DDA1 | 10.99445316 |
| DDAH1 | 10.91380962 |
| DDAH2 | 8.670574253 |
| DDB1 | 7.750102712 |
| DDB2 | 7.046915436 |
| DDC | 11.42314191 |
| DDEF2 | 7.162608796 |
| DDHD2 | 8.237149055 |
| DDI2 | 5.878551808 |
| DDIT3 | 9.512922978 |
| DDIT4 | 11.83227092 |
| DDIT4L | 5.594129433 |
| DDO | 6.453720242 |
| DDOST | 13.01309155 |
| DDR1 | 6.522679435 |
| DDR2 | 6.815863538 |
| DDT | 14.29435251 |
| DDX1 | 12.18112623 |
| DDX10 | 8.848796867 |
| DDX12 | 6.15450562 |
| DDX17 | 9.016987197 |
| DDX18 | 10.93603722 |
| DDX19A | 7.831574121 |
| DDX19B | 6.71056808 |
| DDX19-DDX19L | 5.918455427 |
| DDX20 | 5.806711702 |
| DDX21 | 11.63437495 |
| DDX23 | 8.850453334 |
| DDX24 | 9.771532146 |
| DDX26 | 10.4720746 |
| DDX27 | 9.234771732 |
| DDX28 | 8.892899719 |
| DDX31 | 7.086173348 |
| DDX39 | 10.99922519 |
| DDX3X | 11.1539575 |
| DDX3Y | 6.050891536 |
| DDX41 | 8.085423754 |
| DDX42 | 9.670680496 |
| DDX43 | 6.393536351 |
| DDX46 | 8.024471806 |
| DDX47 | 10.39786166 |
| DDX49 | 6.864068449 |
| DDX5 | 12.39817594 |
| DDX50 | 9.357555499 |
| DDX51 | 6.761809658 |
| DDX52 | 5.577561764 |
| DDX54 | 6.924191448 |
| DDX55 | 9.313969996 |
| DDX56 | 9.803105687 |
| DDX58 | 6.900166667 |
| DDX59 | 9.153690906 |
| DEADC1 | 5.755727719 |
| DEAF1 | 6.151629047 |
| DECR1 | 13.72608205 |
| DECR2 | 12.2763694 |
| DEDD | 6.612064741 |
| DEDD2 | 10.05194284 |
| DEF6 | 5.938614922 |
| DEF8 | 8.684544837 |
| DEFA1 | 8.806326445 |
| DEFA3 | 9.300011363 |
| DEFA4 | 7.421756949 |
| DEFB1 | 11.78614245 |
| DEFB108B | 6.43309297 |
| DEFB125 | 5.978549708 |
| DEFB32 | 5.858935239 |
| DEGS1 | 11.00040675 |
| DEK | 11.68801596 |
| DENND1A | 6.346649245 |
| DENND1C | 5.946319933 |
| DENND2A | 7.41121054 |
| DENND2C | 8.668396213 |
| DENND2D | 8.644416105 |
| DENND4A | 5.85075776 |
| DENND4C | 9.335073321 |
| DENR | 11.1780106 |
| DEPDC5 | 6.092041498 |
| DEPDC6 | 7.965011123 |
| DERA | 13.05402112 |
| DERL1 | 10.87823757 |
| DERL2 | 10.94410194 |
| DERL3 | 6.121031431 |
| DERPC | 6.550953055 |
| DET1 | 7.269456922 |
| DEXI | 11.53826165 |
| DFFA | 7.140844398 |
| DFFB | 6.2342233 |
| DFNA5 | 8.832052058 |
| DGAT1 | 7.355172169 |
| DGAT2 | 8.317965655 |
| DGCR13 | 5.920920741 |
| DGCR14 | 6.784301463 |
| DGCR2 | 7.441851771 |
| DGCR6 | 11.91240303 |
| DGCR6L | 12.03522929 |
| DGCR8 | 6.961244176 |
| DGKA | 5.507912226 |
| DGKB | 8.313243944 |
| DGKD | 6.930645782 |
| DGKE | 5.808322716 |
| DGKQ | 7.655788951 |
| DGKZ | 5.694550132 |
| DGUOK | 8.914137168 |
| DHCR24 | 11.62504362 |
| DHCR7 | 10.22208726 |
| DHDDS | 7.41971624 |
| DHDH | 6.460045426 |
| DHFR | 6.153779213 |
| DHFRL1 | 8.710392788 |
| DHODH | 9.996877145 |
| DHPS | 9.112401573 |
| DHRS1 | 11.09376238 |
| DHRS12 | 6.885261963 |
| DHRS13 | 9.214992854 |
| DHRS2 | 6.429279475 |
| DHRS3 | 11.87810017 |
| DHRS4 | 10.12109034 |
| DHRS4L2 | 10.27689453 |
| DHRS7 | 13.03964491 |
| DHRS7B | 9.727271394 |
| DHRS9 | 7.147298771 |
| DHRSX | 7.295927857 |
| DHTKD1 | 9.584230689 |
| DHX15 | 10.56268992 |
| DHX16 | 9.770515403 |
| DHX29 | 8.477549994 |
| DHX30 | 8.914036635 |
| DHX32 | 10.28915463 |
| DHX33 | 8.991263721 |
| DHX34 | 6.871540375 |
| DHX35 | 6.656971695 |
| DHX36 | 8.96949171 |
| DHX38 | 7.22172864 |
| DHX40 | 5.802527945 |
| DHX57 | 5.661859046 |
| DHX8 | 6.634594765 |
| DHX9 | 6.845243078 |
| DIABLO | 8.707523634 |
| DIAPH1 | 11.32296842 |
| DIAPH2 | 8.946510832 |
| DIAPH3 | 6.066497337 |
| DICER1 | 8.335023027 |
| DIDO1 | 6.421542118 |
| DIMT1L | 11.57200108 |
| DIO1 | 11.2068316 |
| DIO3 | 6.163807952 |
| DIP13B | 8.884461806 |
| DIP2A | 6.16539572 |
| DIP2B | 8.16070088 |
| DIP2C | 8.213224755 |
| DIRAS2 | 5.437293402 |
| DIRAS3 | 6.696614651 |
| DIRC2 | 9.515946743 |
| DIS3L | 10.00744397 |
| DIS3L2 | 6.390085603 |
| DISP1 | 6.948282127 |
| DIXDC1 | 6.947010214 |
| DJ122O8.2 | 10.66112759 |
| dJ341D10.1 | 7.679265389 |
| DKC1 | 10.63429982 |
| DKFZP434B0335 | 7.290139592 |
| DKFZP434B061 | 5.772125987 |
| DKFZp434E1119 | 5.789040516 |
| DKFZp434I1020 | 12.92975674 |
| DKFZp434K191 | 7.576417634 |
| DKFZp434M131 | 5.613961061 |
| DKFZp434N035 | 5.905663073 |
| DKFZp547C195 | 6.253725813 |
| DKFZP564B147 | 8.601509904 |
| DKFZP564J102 | 8.919199043 |
| DKFZp564K142 | 11.63686766 |
| DKFZP564O0523 | 6.542481684 |
| DKFZP586H2123 | 8.025649381 |
| DKFZP586I1420 | 7.080054065 |
| DKFZP586P0123 | 5.777156417 |
| DKFZp667M2411 | 5.442281784 |
| DKFZp686I15217 | 5.785069489 |
| DKFZp686K16132 | 6.016596853 |
| DKFZp686O24166 | 6.137754302 |
| DKFZp761E198 | 5.881732495 |
| DKFZp761O2018 | 6.070167058 |
| DKFZp761P0423 | 8.356652988 |
| DKFZp762E1312 | 6.108292659 |
| DKFZp779B1540 | 5.683809439 |
| DKFZP781I1119 | 5.777214643 |
| DKK3 | 7.22643704 |
| DKKL1 | 6.643933538 |
| DLAT | 8.567645763 |
| DLC1 | 7.066207195 |
| DLD | 10.42559369 |
| DLEC1 | 5.655962001 |
| DLEU1 | 7.194200436 |
| DLG1 | 5.87445834 |
| DLG2 | 6.784023606 |
| DLG4 | 6.215006088 |
| DLG5 | 8.793314277 |
| DLG7 | 5.547489254 |
| DLGAP4 | 7.176924214 |
| DLK2 | 6.157824819 |
| DLL1 | 6.481848069 |
| DLL4 | 5.841269392 |
| DLST | 9.144140555 |
| DLX3 | 5.60435246 |
| DMAP1 | 9.371926046 |
| DMD | 5.945200744 |
| DMGDH | 8.583105704 |
| DMKN | 6.612071548 |
| DMN | 7.989390064 |
| DMPK | 5.625679263 |
| DMRT1 | 8.504010271 |
| DMRTA1 | 8.394193927 |
| DMRTC1 | 7.953908693 |
| DMTF1 | 7.423324142 |
| DMWD | 6.089313031 |
| DMXL1 | 6.321036525 |
| DMXL2 | 5.815350179 |
| DNA2L | 5.798131681 |
| DNAH17 | 5.983844237 |
| DNAH3 | 5.860286813 |
| DNAH5 | 5.830856203 |
| DNAH7 | 5.653989746 |
| DNAI1 | 5.56819645 |
| DNAI2 | 5.569641456 |
| DNAJA1 | 13.33585717 |
| DNAJA2 | 11.76655092 |
| DNAJA3 | 12.92401627 |
| DNAJA4 | 6.763969065 |
| DNAJB1 | 9.147290655 |
| DNAJB11 | 13.1185624 |
| DNAJB12 | 7.015815372 |
| DNAJB14 | 7.209123362 |
| DNAJB2 | 9.834365361 |
| DNAJB4 | 6.753242712 |
| DNAJB5 | 6.614010108 |
| DNAJB6 | 10.44156611 |
| DNAJB7 | 5.976767076 |
| DNAJB8 | 6.187252241 |
| DNAJB9 | 12.40051327 |
| DNAJC1 | 8.160788112 |
| DNAJC10 | 6.115893367 |
| DNAJC11 | 6.124585536 |
| DNAJC12 | 10.8915237 |
| DNAJC13 | 7.39398586 |
| DNAJC14 | 7.95379989 |
| DNAJC15 | 9.355553007 |
| DNAJC16 | 5.86230063 |
| DNAJC17 | 8.688312834 |
| DNAJC19 | 8.937332658 |
| DNAJC21 | 7.227574859 |
| DNAJC22 | 10.40029126 |
| DNAJC27 | 7.370436481 |
| DNAJC3 | 9.569032716 |
| DNAJC5 | 7.173656393 |
| DNAJC5B | 5.751073887 |
| DNAJC7 | 10.23332215 |
| DNAJC8 | 12.42688146 |
| DNAJC9 | 10.30484262 |
| DNAL1 | 7.105653862 |
| DNAL4 | 8.883499259 |
| DNALI1 | 7.719669347 |
| DNAPTP6 | 10.16807765 |
| DNASE1L1 | 6.360765499 |
| DNASE1L3 | 12.35786945 |
| DNASE2 | 11.04567556 |
| DNCL1 | 13.415478 |
| DNCL2A | 9.998302905 |
| DND1 | 5.651569118 |
| DNM1L | 8.231319251 |
| DNM2 | 7.631520658 |
| DNMT1 | 10.8223571 |
| DNMT3A | 5.729208833 |
| DNMT3B | 5.751508411 |
| DNMT3L | 6.653901502 |
| DNPEP | 8.72799234 |
| DNTTIP1 | 9.279402251 |
| DNTTIP2 | 5.771062283 |
| DOC1 | 7.397993915 |
| DOC2A | 7.89083187 |
| DOCK1 | 7.540412354 |
| DOCK10 | 8.189360187 |
| DOCK11 | 7.95168558 |
| DOCK2 | 9.067727753 |
| DOCK5 | 5.838775204 |
| DOCK6 | 7.292448112 |
| DOCK7 | 8.646728873 |
| DOCK8 | 7.405367157 |
| DOCK9 | 6.680099867 |
| DOHH | 6.72862353 |
| DOK1 | 5.780069722 |
| DOK2 | 7.278224259 |
| DOK4 | 7.643677074 |
| DOLK | 8.833256293 |
| DOLPP1 | 9.225624275 |
| DOM3Z | 7.365991272 |
| DONSON | 6.896032299 |
| DOPEY1 | 6.016097117 |
| DOPEY2 | 6.486839809 |
| DPAGT1 | 9.359225331 |
| DPCR1 | 6.05138749 |
| DPEP3 | 5.603506976 |
| DPF1 | 5.786493622 |
| DPF2 | 8.901966586 |
| DPH2 | 7.978546508 |
| DPH3 | 9.606972514 |
| DPH5 | 9.748047736 |
| DPM1 | 11.08136783 |
| DPM2 | 7.221124962 |
| DPM3 | 11.6806378 |
| DPP3 | 6.719031544 |
| DPP4 | 7.729660824 |
| DPP6 | 6.255382099 |
| DPP7 | 7.054603454 |
| DPP8 | 8.538611714 |
| DPP9 | 9.122958311 |
| DPT | 7.868199352 |
| DPY19L1 | 10.48979781 |
| DPY19L3 | 5.397011178 |
| DPY19L4 | 8.549995863 |
| DPY30 | 11.0181493 |
| DPYD | 10.02001902 |
| DPYS | 12.79928366 |
| DPYSL2 | 11.0062219 |
| DPYSL3 | 8.545686359 |
| DR1 | 8.152222346 |
| DRAP1 | 10.67330598 |
| DRB1 | 9.415057006 |
| DRD3 | 5.691822141 |
| DRD4 | 6.280784425 |
| DREV1 | 5.611674105 |
| DRG1 | 10.52170698 |
| DRG2 | 7.245693045 |
| DSC2 | 8.489450867 |
| DSCAM | 5.761983184 |
| DSCC1 | 6.782666107 |
| DSCR10 | 6.821544788 |
| DSCR1L1 | 6.734435209 |
| DSCR3 | 9.987570687 |
| DSCR5 | 8.624240242 |
| DSCR6 | 6.480690121 |
| DSCR9 | 5.959261538 |
| DSEL | 6.275189847 |
| DSG2 | 6.178193158 |
| DSN1 | 8.016382064 |
| DSP | 8.195589058 |
| DST | 5.79087861 |
| DSTN | 9.356591676 |
| DTL | 7.823863355 |
| DTNA | 6.765766317 |
| DTNBP1 | 6.564100597 |
| DTWD1 | 9.102909559 |
| DTWD2 | 7.152061664 |
| DTX1 | 8.022555632 |
| DTX2 | 9.19622968 |
| DTX3L | 7.491088123 |
| DTYMK | 9.89300797 |
| DULLARD | 7.401478411 |
| DUPD1 | 5.696336148 |
| DUS1L | 8.014737654 |
| DUS2L | 8.600994209 |
| DUS3L | 8.973108505 |
| DUS4L | 8.325135888 |
| DUSP1 | 12.98902476 |
| DUSP10 | 7.239446103 |
| DUSP11 | 10.11087045 |
| DUSP12 | 9.953394506 |
| DUSP13 | 5.946491016 |
| DUSP14 | 9.479140623 |
| DUSP16 | 8.419086936 |
| DUSP18 | 6.267924115 |
| DUSP19 | 5.696708367 |
| DUSP2 | 6.240040275 |
| DUSP22 | 9.897138811 |
| DUSP23 | 13.00156278 |
| DUSP26 | 5.89187847 |
| DUSP27 | 5.87073888 |
| DUSP28 | 8.104416256 |
| DUSP3 | 12.52390152 |
| DUSP4 | 5.956179175 |
| DUSP5 | 10.10561095 |
| DUSP6 | 6.966360248 |
| DUSP8 | 5.626816793 |
| DUSP9 | 6.579407193 |
| DUT | 7.874782807 |
| DVL2 | 6.091676313 |
| DVL3 | 6.910868781 |
| DXS9879E | 10.94612599 |
| DYDC2 | 5.81225257 |
| DYM | 10.00737359 |
| DYNC1H1 | 11.89862792 |
| DYNC1I2 | 10.07695457 |
| DYNC1LI1 | 6.538325269 |
| DYNC1LI2 | 10.82712755 |
| DYNC2H1 | 6.04602656 |
| DYNC2LI1 | 7.044489151 |
| DYNLL2 | 10.54067049 |
| DYNLRB1 | 11.45581147 |
| DYNLRB2 | 6.462704636 |
| DYNLT1 | 11.67497958 |
| DYNLT3 | 9.799886861 |
| DYRK1A | 10.23611548 |
| DYRK2 | 6.694787237 |
| DYRK3 | 8.63373299 |
| DYRK4 | 9.864497728 |
| DYSF | 9.064826701 |
| E2F1 | 6.141790815 |
| E2F2 | 6.799324097 |
| E2F3 | 9.624881154 |
| E2F4 | 9.855812367 |
| E2F5 | 7.930696845 |
| E2F6 | 8.954543747 |
| E4F1 | 9.389109538 |
| EAF1 | 8.904288275 |
| EAF2 | 7.586145799 |
| EARS2 | 8.511734636 |
| EBAG9 | 9.643693657 |
| EBF1 | 5.696014239 |
| EBI2 | 7.990545769 |
| EBI3 | 8.784941166 |
| EBNA1BP2 | 12.29330468 |
| EBP | 12.50657574 |
| EBPL | 11.90459242 |
| ECAT8 | 6.481500081 |
| ECD | 7.264580013 |
| ECE1 | 6.104146248 |
| ECGF1 | 7.566583971 |
| ECH1 | 13.03316075 |
| ECHDC1 | 6.532310889 |
| ECHDC2 | 13.92612993 |
| ECHDC3 | 11.34337333 |
| ECHS1 | 13.43354978 |
| ECM1 | 6.886822432 |
| ECM2 | 6.835826072 |
| ECOP | 8.050532972 |
| ECSIT | 8.566870117 |
| EDAR | 5.332349475 |
| EDARADD | 6.270743803 |
| EDC3 | 6.308944913 |
| EDC4 | 9.279229639 |
| EDEM1 | 11.04284896 |
| EDEM2 | 9.772750945 |
| EDEM3 | 5.758812972 |
| EDF1 | 10.59092679 |
| EDG1 | 8.660059248 |
| EDG4 | 6.382078969 |
| EDG6 | 5.778665182 |
| EDN1 | 7.29752887 |
| EDNRA | 6.759602361 |
| EDNRB | 6.963834021 |
| EED | 6.522725386 |
| EEF1A1 | 15.35439027 |
| EEF1A2 | 5.987306591 |
| EEF1B2 | 12.03813615 |
| EEF1D | 11.11423697 |
| EEF1E1 | 11.07589507 |
| EEF1G | 14.00932456 |
| EEF2 | 13.46089982 |
| EEF2K | 10.06397936 |
| EEFSEC | 6.133684765 |
| EEPD1 | 7.01376345 |
| EFCAB4A | 5.855447779 |
| EFCAB7 | 5.661723303 |
| EFEMP1 | 6.954653149 |
| EFEMP2 | 7.981755968 |
| EFHA1 | 10.99184481 |
| EFHA2 | 5.828632648 |
| EFHB | 6.06753385 |
| EFHC1 | 5.819558336 |
| EFHD1 | 10.10014387 |
| EFHD2 | 10.78724531 |
| EFNA1 | 12.24442094 |
| EFNA3 | 6.535733443 |
| EFNA4 | 7.006337312 |
| EFNB1 | 6.599439161 |
| EFNB2 | 9.58546385 |
| EFNB3 | 6.152555302 |
| EFTUD1 | 7.658051981 |
| EFTUD2 | 9.832620261 |
| EGFL11 | 8.113041785 |
| EGFL7 | 5.835267739 |
| EGFLAM | 8.071116964 |
| EGFR | 7.193758442 |
| EGLN1 | 7.977105686 |
| EGLN2 | 7.54124368 |
| EGLN3 | 6.242421627 |
| EGR1 | 11.63527618 |
| EGR2 | 8.211854292 |
| EGR3 | 5.717872056 |
| EHBP1 | 10.9093311 |
| EHD1 | 10.85266115 |
| EHD2 | 5.989864421 |
| EHD3 | 6.25867431 |
| EHD4 | 10.28851563 |
| EHHADH | 9.543190623 |
| EHMT1 | 5.657708208 |
| EHMT2 | 5.957242179 |
| EI24 | 11.71778371 |
| EID1 | 6.574174888 |
| EID2 | 8.712223857 |
| EIF1 | 12.57370786 |
| EIF1AX | 10.17164513 |
| EIF1AY | 11.69986361 |
| EIF1B | 11.33270741 |
| eIF2A | 10.90775353 |
| EIF2AK1 | 10.52345128 |
| EIF2AK2 | 10.79217897 |
| EIF2AK3 | 8.200099057 |
| EIF2AK4 | 8.832710313 |
| EIF2B1 | 9.416860061 |
| EIF2B2 | 9.769947224 |
| EIF2B3 | 10.47433933 |
| EIF2B4 | 10.99624342 |
| EIF2B5 | 10.1747677 |
| EIF2C1 | 7.49872454 |
| EIF2C2 | 9.020140956 |
| EIF2C3 | 6.663504256 |
| EIF2C4 | 6.128176461 |
| EIF2S1 | 9.358379106 |
| EIF2S2 | 7.917530505 |
| EIF2S3 | 10.43273768 |
| EIF3C | 7.192128944 |
| EIF3EIP | 12.43221403 |
| EIF3M | 12.45967208 |
| EIF3S1 | 10.36495152 |
| EIF3S10 | 11.54908293 |
| EIF3S12 | 12.45971261 |
| EIF3S2 | 12.66718893 |
| EIF3S3 | 11.15368345 |
| EIF3S4 | 11.51570259 |
| EIF3S5 | 11.98450539 |
| EIF3S6 | 14.01111696 |
| EIF3S7 | 11.44524986 |
| EIF3S9 | 10.47249726 |
| EIF4A1 | 14.41784102 |
| EIF4A2 | 12.76248969 |
| EIF4A3 | 11.58035891 |
| EIF4B | 11.29454851 |
| EIF4E2 | 11.1133483 |
| EIF4E3 | 7.244844095 |
| EIF4EBP1 | 7.587312296 |
| EIF4EBP2 | 10.82456636 |
| EIF4EBP3 | 9.166127398 |
| EIF4ENIF1 | 7.572090491 |
| EIF4G1 | 8.369931069 |
| EIF4G2 | 12.43818646 |
| EIF4G3 | 9.234365806 |
| EIF4H | 8.541093815 |
| EIF5 | 8.868856154 |
| EIF5A | 11.76880568 |
| EIF5A2 | 7.221923434 |
| EIF5B | 8.730971622 |
| EIF6 | 8.52775954 |
| ELA1 | 5.768304863 |
| ELA2 | 8.139105902 |
| ELA2A | 6.124120543 |
| ELA2B | 5.692584015 |
| ELA3A | 5.862517648 |
| ELA3B | 5.72038237 |
| ELAC1 | 6.419278017 |
| ELAC2 | 8.220286862 |
| ELAVL1 | 6.045802177 |
| ELF1 | 10.39344957 |
| ELF2 | 7.473777064 |
| ELF3 | 8.873247251 |
| ELF4 | 7.075745645 |
| ELF5 | 5.873678908 |
| ELK1 | 10.30484168 |
| ELK4 | 5.973995141 |
| ELL | 7.700408087 |
| ELL2 | 9.952177971 |
| Ells1 | 7.333616665 |
| ELMO1 | 6.544422236 |
| ELMO2 | 5.521211113 |
| ELMO3 | 6.447707301 |
| ELMOD2 | 8.713888991 |
| ELOF1 | 8.59203794 |
| ELOVL1 | 8.518515379 |
| ELOVL2 | 8.275172509 |
| ELOVL4 | 5.560786513 |
| ELOVL5 | 10.93616778 |
| ELOVL6 | 9.433488977 |
| ELP2 | 9.882322732 |
| ELP3 | 10.20059005 |
| ELP4 | 8.36684348 |
| ELSPBP1 | 8.708826859 |
| EMCN | 8.095468672 |
| EMD | 11.15294329 |
| EME2 | 6.463701669 |
| EMG1 | 10.59281714 |
| EMILIN1 | 6.50628186 |
| EMILIN2 | 8.397021268 |
| EML1 | 8.63192106 |
| EML2 | 9.458235826 |
| EML3 | 10.11340812 |
| EML4 | 11.20300811 |
| EMP1 | 9.469190377 |
| EMP3 | 9.525443036 |
| EMR1 | 6.554920293 |
| EMR2 | 8.570071344 |
| EMR3 | 5.7132739 |
| EMR4 | 5.780228815 |
| EMX2 | 5.636326678 |
| EN2 | 5.815935644 |
| ENAH | 5.477842911 |
| ENC1 | 8.184930953 |
| ENDOG | 9.144014936 |
| ENG | 8.611449249 |
| ENO1 | 13.72413137 |
| ENO2 | 5.777227545 |
| ENO3 | 10.82567908 |
| ENOPH1 | 10.00905741 |
| ENOSF1 | 10.66803951 |
| ENOX2 | 5.930485035 |
| ENPEP | 10.30352802 |
| ENPP1 | 9.385829556 |
| ENPP2 | 8.767751632 |
| ENPP3 | 6.585489479 |
| ENPP4 | 9.356278439 |
| ENPP7 | 6.104060688 |
| ENSA | 7.164103658 |
| ENTHD1 | 5.540647395 |
| ENTPD1 | 6.800367621 |
| ENTPD2 | 5.796191833 |
| ENTPD4 | 6.118031605 |
| ENTPD5 | 8.530445794 |
| ENTPD7 | 7.981650624 |
| ENTPD8 | 6.548604797 |
| ENY2 | 12.53967101 |
| EOMES | 7.33844921 |
| EP300 | 6.756198238 |
| EP400 | 7.864628544 |
| EPAS1 | 12.95981226 |
| EPB41 | 5.958122216 |
| EPB41L1 | 5.672383836 |
| EPB41L2 | 7.645206468 |
| EPB41L3 | 9.904073688 |
| EPB41L4B | 7.591052553 |
| EPB41L5 | 6.087642339 |
| EPB42 | 5.426156324 |
| EPB49 | 6.231272489 |
| EPC1 | 7.234584419 |
| EPC2 | 6.013908988 |
| EPDR1 | 10.28238939 |
| EPHA1 | 8.29706134 |
| EPHA2 | 9.982001344 |
| EPHA3 | 6.435722778 |
| EPHA8 | 5.790889445 |
| EPHB1 | 6.084077875 |
| EPHB4 | 7.943225454 |
| EPHB6 | 5.440359634 |
| EPHX1 | 10.84171888 |
| EPHX2 | 11.28289615 |
| EPM2A | 7.265173389 |
| EPM2AIP1 | 8.627755633 |
| EPN1 | 12.15289849 |
| EPN2 | 6.121331693 |
| EPO | 10.29256465 |
| EPOR | 5.8645593 |
| EPPB9 | 8.183868596 |
| EPRS | 11.29209749 |
| EPS15 | 8.119223938 |
| EPS8 | 8.9044618 |
| EPSTI1 | 9.549478749 |
| ERAF | 6.272099497 |
| ERAL1 | 9.878945891 |
| ERBB2 | 6.095504603 |
| ERBB2IP | 5.82627523 |
| ERBB3 | 8.022545125 |
| ERCC1 | 7.088481771 |
| ERCC2 | 7.588748636 |
| ERCC3 | 8.183184639 |
| ERCC5 | 9.582611982 |
| ERCC6 | 5.610105333 |
| ERCC6L | 5.578408879 |
| ERCC8 | 6.845463523 |
| ERF | 7.834449092 |
| ERG | 5.770903509 |
| ERGIC1 | 8.552254031 |
| ERGIC2 | 10.10067213 |
| ERGIC3 | 9.36558264 |
| ERH | 12.67298166 |
| ERICH1 | 8.316222058 |
| ERLIN1 | 9.958044808 |
| ERLIN2 | 7.267794942 |
| ERMAP | 7.361448171 |
| ERMN | 7.413198541 |
| ERMP1 | 8.480464814 |
| ERN1 | 7.533975734 |
| ERO1L | 9.148209544 |
| ERO1LB | 6.926077871 |
| ERP27 | 5.90497366 |
| ERRFI1 | 12.90573589 |
| ESCO1 | 5.990632699 |
| ESD | 12.6925429 |
| ESF1 | 8.700173487 |
| ESM1 | 5.764820739 |
| ESPN | 6.33828741 |
| ESPNP | 5.624957358 |
| ESR1 | 8.613754972 |
| ESR2 | 5.870421672 |
| ESRRA | 8.526476062 |
| ESRRB | 5.596506652 |
| ESRRG | 5.983235342 |
| ETF1 | 9.944031001 |
| ETFA | 13.7706179 |
| ETFB | 13.58259312 |
| ETFDH | 11.62369685 |
| ETHE1 | 6.4198506 |
| ETNK1 | 9.442085823 |
| ETNK2 | 7.873063716 |
| ETS1 | 10.53925897 |
| ETS2 | 10.1630575 |
| ETV2 | 5.695653951 |
| ETV3 | 6.009446557 |
| ETV4 | 5.719730363 |
| ETV5 | 7.675402367 |
| ETV6 | 9.419863921 |
| EVC2 | 10.76088901 |
| EVI1 | 7.793075697 |
| EVI2A | 6.539183113 |
| EVI2B | 7.979541526 |
| EVI5 | 9.4548469 |
| EVI5L | 8.546940422 |
| EVL | 12.77904197 |
| EVX1 | 5.987759003 |
| EWSR1 | 11.08348915 |
| EXDL2 | 7.88498682 |
| EXO1 | 6.225722631 |
| EXOC1 | 8.007304979 |
| EXOC2 | 7.17746491 |
| EXOC6 | 9.314435044 |
| EXOC7 | 8.170465786 |
| EXOC8 | 8.48000877 |
| EXOSC1 | 9.094039497 |
| EXOSC10 | 8.815707973 |
| EXOSC2 | 7.893243406 |
| EXOSC3 | 10.58743126 |
| EXOSC4 | 9.062393865 |
| EXOSC5 | 9.324507504 |
| EXOSC6 | 9.644079605 |
| EXOSC7 | 9.706047006 |
| EXOSC8 | 11.27770249 |
| EXOSC9 | 9.329846119 |
| EXPH5 | 6.424234071 |
| EXT1 | 11.66272953 |
| EXT2 | 9.588895145 |
| EXTL1 | 5.8505362 |
| EXTL2 | 7.179038331 |
| EXTL3 | 7.296534934 |
| EYA2 | 6.432516915 |
| EYA3 | 5.599267514 |
| EZH1 | 6.928833006 |
| EZH2 | 5.994324622 |
| F10 | 10.97978253 |
| F11 | 8.671013767 |
| F11R | 9.749894248 |
| F12 | 13.43375204 |
| F13A1 | 6.531625857 |
| F13B | 10.54955803 |
| F2 | 15.15824917 |
| F2R | 8.63936165 |
| F2RL1 | 8.772728728 |
| F2RL2 | 5.528497035 |
| F2RL3 | 5.509462149 |
| F3 | 5.750797246 |
| F5 | 11.73213299 |
| F7 | 10.48235439 |
| F8 | 6.342098681 |
| F8A1 | 8.546382871 |
| F8A3 | 5.627556779 |
| F9 | 14.10522038 |
| FAAH | 7.458290663 |
| FAAH2 | 8.486632326 |
| FABP1 | 13.83450113 |
| FABP2 | 5.812368402 |
| FABP4 | 7.704123762 |
| FABP5 | 5.998231324 |
| FABP7 | 6.221540383 |
| FADD | 9.918726188 |
| FADS1 | 10.17907386 |
| FADS2 | 6.820495429 |
| FADS3 | 9.34718923 |
| FAF1 | 8.139813263 |
| FAH | 11.91431515 |
| FAHD1 | 8.5680776 |
| FAHD2A | 7.092061449 |
| FAHD2B | 10.40692655 |
| FAIM | 7.270657041 |
| FAIM3 | 7.795707822 |
| FALZ | 5.876688271 |
| FAM100A | 7.308928488 |
| FAM100B | 9.577257052 |
| FAM101A | 6.046779648 |
| FAM101B | 6.162269577 |
| FAM102B | 7.157820083 |
| FAM103A1 | 8.927196357 |
| FAM104A | 9.460995207 |
| FAM104B | 7.429653365 |
| FAM105A | 6.922875334 |
| FAM105B | 9.16379939 |
| FAM107A | 7.524765632 |
| FAM107B | 11.99538085 |
| FAM108B1 | 7.323192656 |
| FAM109A | 7.197526726 |
| FAM109B | 7.291850596 |
| FAM10A4 | 10.3869049 |
| FAM10A7 | 6.40319146 |
| FAM111A | 7.181609558 |
| FAM111B | 6.052630798 |
| FAM113A | 6.50903439 |
| FAM113B | 8.280723361 |
| FAM114A1 | 7.718489184 |
| FAM116A | 8.962339053 |
| FAM119A | 6.826475699 |
| FAM119B | 7.769156333 |
| FAM120A | 11.31537704 |
| FAM120B | 10.43929849 |
| FAM122A | 7.586922344 |
| FAM122B | 5.876082855 |
| FAM123A | 5.885073169 |
| FAM124A | 6.260798581 |
| FAM124B | 6.200978673 |
| FAM125A | 9.877287335 |
| FAM125B | 6.546167457 |
| FAM126B | 7.486239967 |
| FAM128B | 5.579088377 |
| FAM129B | 10.48919862 |
| FAM130A1 | 7.89979305 |
| FAM131A | 6.459791565 |
| FAM134C | 8.567111537 |
| FAM135A | 5.759618339 |
| FAM136A | 10.46922566 |
| FAM13A1 | 8.801416359 |
| FAM14A | 7.389260219 |
| FAM14B | 7.458795985 |
| FAM151A | 6.093552855 |
| FAM152B | 11.53508745 |
| FAM156A | 6.346945804 |
| FAM158A | 8.453282799 |
| FAM162A | 10.80209994 |
| FAM164A | 6.472335987 |
| FAM171A1 | 10.10528916 |
| FAM173A | 10.32839788 |
| FAM174A | 9.548594083 |
| FAM176B | 6.196430672 |
| FAM18B | 10.50730812 |
| FAM18B2 | 7.591530302 |
| FAM20A | 8.61968124 |
| FAM20B | 10.57257883 |
| FAM20C | 6.934053684 |
| FAM21B | 5.558964132 |
| FAM21C | 5.492375883 |
| FAM22F | 5.697128612 |
| FAM23B | 5.798971061 |
| FAM26F | 10.44632639 |
| FAM29A | 5.71685564 |
| FAM32A | 10.73689985 |
| FAM33A | 7.600326694 |
| FAM35A | 7.68877141 |
| FAM36A | 9.983688903 |
| FAM38A | 9.569267147 |
| FAM38B | 7.059272661 |
| FAM3A | 11.18107457 |
| FAM3C | 9.805200048 |
| FAM3D | 6.110459584 |
| FAM40A | 8.608048528 |
| FAM41C | 5.706193588 |
| FAM43A | 8.514007781 |
| FAM44A | 5.651969632 |
| FAM44B | 10.829375 |
| FAM45A | 10.4452292 |
| FAM45B | 5.712296203 |
| FAM46A | 12.0496585 |
| FAM46C | 9.65596905 |
| FAM48A | 6.004844402 |
| FAM49A | 6.948521391 |
| FAM49B | 8.162566709 |
| FAM50A | 11.60780356 |
| FAM50B | 8.457293805 |
| FAM51A1 | 6.875753782 |
| FAM53A | 6.394145998 |
| FAM53C | 9.904033798 |
| FAM54B | 10.19296539 |
| FAM57A | 6.972618528 |
| FAM58A | 9.505708143 |
| FAM59A | 7.782523195 |
| FAM60A | 9.454269187 |
| FAM62A | 11.15380578 |
| FAM62B | 6.409131166 |
| FAM63A | 10.15952011 |
| FAM63B | 6.874655629 |
| FAM64A | 5.801649014 |
| FAM65A | 9.576623313 |
| FAM65B | 7.839898455 |
| FAM69B | 7.012932469 |
| FAM70B | 6.826632524 |
| FAM71E1 | 6.374884295 |
| FAM73A | 7.196448979 |
| FAM73B | 6.338584623 |
| FAM76A | 7.694183208 |
| FAM76B | 7.187381135 |
| FAM7A1 | 5.52625832 |
| FAM7A3 | 5.745748734 |
| FAM82A | 5.818256068 |
| FAM82A2 | 11.12202388 |
| FAM82B | 9.864928806 |
| FAM83F | 7.591894682 |
| FAM84B | 9.297191556 |
| FAM86A | 8.490195417 |
| FAM86B1 | 7.91951601 |
| FAM86C | 5.886980902 |
| FAM89A | 8.715242451 |
| FAM89B | 8.231243251 |
| FAM8A1 | 12.19257845 |
| FAM91A1 | 7.117265106 |
| FAM92A1 | 7.288424185 |
| FAM96A | 13.63463954 |
| FAM96B | 11.79217413 |
| FAM98A | 10.59833733 |
| FAM98C | 8.605344733 |
| FANCA | 5.80250569 |
| FANCB | 5.610419523 |
| FANCC | 8.415097483 |
| FANCD2 | 6.264814688 |
| FANCE | 6.770275551 |
| FANCG | 6.505633198 |
| FANCL | 7.256165186 |
| FANK1 | 5.591898058 |
| FAP | 5.68925725 |
| FAR1 | 6.055757561 |
| FARP1 | 7.266427863 |
| FARS2 | 9.653809334 |
| FARSLA | 8.335311009 |
| FARSLB | 6.753566296 |
| FAS | 6.255994862 |
| FASN | 11.00491528 |
| FASTK | 6.799461962 |
| FASTKD1 | 6.951642289 |
| FASTKD2 | 7.116146624 |
| FASTKD3 | 8.941044697 |
| FASTKD5 | 9.309167779 |
| FAT | 9.101325441 |
| FAT2 | 5.771907241 |
| FAT3 | 6.319313704 |
| FAT4 | 5.768723192 |
| FAU | 14.1968921 |
| FBL | 10.92323396 |
| FBLIM1 | 6.079171061 |
| FBLN1 | 7.563715368 |
| FBLN2 | 7.135178337 |
| FBLN5 | 6.087617996 |
| FBLN7 | 6.031261202 |
| FBN2 | 5.626629885 |
| FBP1 | 12.93916327 |
| FBXL10 | 7.760039984 |
| FBXL11 | 8.768285271 |
| FBXL12 | 7.557237014 |
| FBXL15 | 9.224067985 |
| FBXL17 | 6.259005918 |
| FBXL19 | 8.264752842 |
| FBXL20 | 7.448856461 |
| FBXL3 | 8.284089487 |
| FBXL5 | 6.754315094 |
| FBXL6 | 8.03078806 |
| FBXO11 | 7.026150533 |
| FBXO15 | 5.584407576 |
| FBXO16 | 5.94477736 |
| FBXO17 | 6.726192611 |
| FBXO18 | 10.2040944 |
| FBXO2 | 7.826402736 |
| FBXO21 | 10.8955495 |
| FBXO22 | 8.271824622 |
| FBXO24 | 5.967039099 |
| FBXO25 | 5.876554807 |
| FBXO27 | 8.244681964 |
| FBXO28 | 8.760493201 |
| FBXO3 | 7.86147181 |
| FBXO30 | 7.748469089 |
| FBXO31 | 9.38958072 |
| FBXO32 | 6.420949673 |
| FBXO33 | 9.78975588 |
| FBXO34 | 8.861902567 |
| FBXO36 | 7.500946217 |
| FBXO38 | 8.931478695 |
| FBXO4 | 8.636263285 |
| FBXO42 | 7.127770218 |
| FBXO43 | 6.645254517 |
| FBXO44 | 5.605028317 |
| FBXO46 | 7.593484499 |
| FBXO5 | 8.04421206 |
| FBXO6 | 9.089192026 |
| FBXO7 | 7.752309856 |
| FBXO8 | 10.81151567 |
| FBXO9 | 6.404209128 |
| FBXW11 | 9.888526067 |
| FBXW2 | 6.275414609 |
| FBXW4 | 8.914750549 |
| FBXW5 | 8.230047793 |
| FBXW7 | 7.497508301 |
| FBXW9 | 7.715594912 |
| FCAMR | 7.935573901 |
| FCER1A | 6.070341951 |
| FCER1G | 11.28195926 |
| FCF1 | 6.883433978 |
| FCGBP | 5.838287145 |
| FCGR1A | 5.76494441 |
| FCGR2A | 7.30136232 |
| FCGR2B | 8.548860772 |
| FCGR3A | 5.733071374 |
| FCGR3B | 8.133058935 |
| FCGRT | 11.72103725 |
| FCHO2 | 7.892960456 |
| FCHSD1 | 5.599543929 |
| FCHSD2 | 9.267588822 |
| FCN1 | 10.03755568 |
| FCN2 | 9.179220701 |
| FCN3 | 14.59271001 |
| FCRL3 | 5.998128297 |
| FCRL6 | 8.309055958 |
| FCRLB | 6.751691339 |
| FDFT1 | 10.60358652 |
| FDPS | 9.397817448 |
| FDX1 | 7.116344781 |
| FDXR | 9.508365744 |
| FECH | 9.424836087 |
| FEM1A | 10.1078119 |
| FEM1B | 6.915292119 |
| FEM1C | 9.813451275 |
| FEN1 | 7.354447296 |
| FER | 6.322709471 |
| FER1L3 | 5.522225389 |
| FERD3L | 6.349412618 |
| FERMT2 | 12.14181951 |
| FERMT3 | 7.058466379 |
| FES | 8.006874249 |
| FETUB | 8.23861807 |
| FEZ1 | 7.14225741 |
| FEZ2 | 11.14205702 |
| FEZF1 | 5.650414557 |
| FFAR2 | 5.850480243 |
| FFAR3 | 6.079690994 |
| FGA | 14.74234103 |
| FGB | 15.37840146 |
| FGD2 | 5.588554943 |
| FGD3 | 7.567481994 |
| FGD4 | 5.914161865 |
| FGD5 | 7.748567338 |
| FGD6 | 5.978686178 |
| FGF1 | 5.807302742 |
| FGF12 | 5.762657577 |
| FGF13 | 5.841561012 |
| FGF14 | 5.816815728 |
| FGF17 | 5.724815362 |
| FGF18 | 6.734070588 |
| FGF19 | 6.471739565 |
| FGF21 | 7.294340658 |
| FGF23 | 6.06916299 |
| FGF4 | 5.534317263 |
| FGF6 | 7.334596234 |
| FGF9 | 6.764555479 |
| FGFBP2 | 5.517385295 |
| FGFR1OP | 6.923060522 |
| FGFR1OP2 | 7.228498571 |
| FGFR3 | 10.91260471 |
| FGFR4 | 6.643786068 |
| FGFRL1 | 9.622164518 |
| FGG | 12.14201266 |
| FGL1 | 12.40890988 |
| FGL2 | 9.319029335 |
| FGR | 8.264573578 |
| FH | 13.0064478 |
| FHIT | 7.24119706 |
| FHL1 | 8.575023359 |
| FHL2 | 9.469601213 |
| FHL3 | 6.616617056 |
| FHL5 | 6.08567782 |
| FHOD1 | 6.680486829 |
| FHOD3 | 5.539153397 |
| FIBCD1 | 5.966705548 |
| FIBP | 10.84961843 |
| FICD | 10.00428504 |
| FIG4 | 7.59273657 |
| FIGNL1 | 5.928403384 |
| FILIP1 | 5.648404577 |
| FIP1L1 | 8.361382309 |
| FIS | 10.70954017 |
| FIS1 | 11.67691213 |
| FIT1 | 9.076388547 |
| FIZ1 | 6.688273662 |
| FJX1 | 8.353834086 |
| FKBP11 | 12.92763175 |
| FKBP14 | 8.724847727 |
| FKBP1A | 9.522828316 |
| FKBP2 | 8.221229708 |
| FKBP3 | 7.638488497 |
| FKBP4 | 10.23472468 |
| FKBP5 | 10.05194897 |
| FKBP7 | 5.524111826 |
| FKBP8 | 7.352302617 |
| FKBP9 | 6.142171135 |
| FKBPL | 6.280227693 |
| FKRP | 6.10887397 |
| FKSG14 | 5.845438203 |
| FKSG24 | 7.727989805 |
| FKSG30 | 10.53693787 |
| FKSG44 | 10.53190618 |
| FLAD1 | 7.367789107 |
| FLCN | 6.909470556 |
| FLI1 | 8.444884682 |
| FLII | 8.088951331 |
| FLJ00038 | 8.443930756 |
| FLJ10081 | 11.38560644 |
| FLJ10099 | 9.015637573 |
| FLJ10154 | 10.73580545 |
| FLJ10159 | 7.947160533 |
| FLJ10213 | 6.166889718 |
| FLJ10357 | 6.112226471 |
| FLJ10374 | 7.822111791 |
| FLJ10404 | 5.762982384 |
| FLJ10769 | 9.39031184 |
| FLJ10781 | 6.246700201 |
| FLJ10803 | 5.758289235 |
| FLJ10916 | 8.744994432 |
| FLJ10986 | 12.62308202 |
| FLJ11000 | 10.43609976 |
| FLJ11151 | 7.142943347 |
| FLJ11171 | 8.796410725 |
| FLJ11184 | 5.865243894 |
| FLJ11235 | 6.514764124 |
| FLJ11259 | 8.856558885 |
| FLJ11286 | 10.85347 |
| FLJ11506 | 9.292949798 |
| FLJ11795 | 6.594407482 |
| FLJ12078 | 6.143052159 |
| FLJ12355 | 6.682908061 |
| FLJ12649 | 6.373858607 |
| FLJ12681 | 6.373701205 |
| FLJ12788 | 8.428218829 |
| FLJ12886 | 6.145971564 |
| FLJ12949 | 5.738426176 |
| FLJ13195 | 6.99677913 |
| FLJ13576 | 6.194984688 |
| FLJ13611 | 8.130235122 |
| FLJ13614 | 5.894844921 |
| FLJ13946 | 6.251987531 |
| FLJ14054 | 5.988852391 |
| FLJ14154 | 8.613253296 |
| FLJ14213 | 6.580214218 |
| FLJ14803 | 9.37744318 |
| FLJ16323 | 5.454279405 |
| FLJ16331 | 6.180557159 |
| FLJ20035 | 7.07011142 |
| FLJ20125 | 7.531765776 |
| FLJ20152 | 7.576669332 |
| FLJ20160 | 6.841250825 |
| FLJ20209 | 6.294477087 |
| FLJ20245 | 6.861126037 |
| FLJ20254 | 9.014302489 |
| FLJ20273 | 9.23294079 |
| FLJ20280 | 6.368120733 |
| FLJ20323 | 8.619311689 |
| FLJ20364 | 6.175586299 |
| FLJ20366 | 10.45309341 |
| FLJ20397 | 7.223061567 |
| FLJ20444 | 5.799227782 |
| FLJ20489 | 6.161278958 |
| FLJ20581 | 7.163527009 |
| FLJ20628 | 8.44548916 |
| FLJ20674 | 7.712169354 |
| FLJ20699 | 11.37197348 |
| FLJ20712 | 6.145528738 |
| FLJ20718 | 9.41174715 |
| FLJ20850 | 5.451769561 |
| FLJ20920 | 7.478900832 |
| FLJ21062 | 9.006876284 |
| FLJ21103 | 6.550359169 |
| FLJ21106 | 6.855633241 |
| FLJ21125 | 6.241580902 |
| FLJ21127 | 8.475120682 |
| FLJ21438 | 8.317877512 |
| FLJ21687 | 5.837291779 |
| FLJ21742 | 5.782365603 |
| FLJ21749 | 9.984259581 |
| FLJ21816 | 7.152824518 |
| FLJ21839 | 5.860583617 |
| FLJ21865 | 5.769983268 |
| FLJ21986 | 11.33762718 |
| FLJ22222 | 6.955024371 |
| FLJ22374 | 6.372466863 |
| FLJ22531 | 7.035584032 |
| FLJ22662 | 9.620406758 |
| FLJ22795 | 7.20706041 |
| FLJ23152 | 5.973741343 |
| FLJ23322 | 6.43645169 |
| FLJ23436 | 7.541281705 |
| FLJ23577 | 6.216651016 |
| FLJ23584 | 6.527691633 |
| FLJ23754 | 5.776559444 |
| FLJ25037 | 5.479570225 |
| FLJ25222 | 8.286870168 |
| FLJ25530 | 9.379532035 |
| FLJ25758 | 6.087089213 |
| FLJ25778 | 5.906286598 |
| FLJ27255 | 5.95692035 |
| FLJ27354 | 5.701426451 |
| FLJ27465 | 5.970246484 |
| FLJ30046 | 8.952201351 |
| FLJ30092 | 5.816077591 |
| FLJ30430 | 5.815143677 |
| FLJ30679 | 5.877821145 |
| FLJ30719 | 5.803557982 |
| FLJ31818 | 5.392429851 |
| FLJ31945 | 5.995232319 |
| FLJ32011 | 5.971249347 |
| FLJ32549 | 7.785304993 |
| FLJ32784 | 5.685072255 |
| FLJ32810 | 6.017397192 |
| FLJ33996 | 5.931854007 |
| FLJ34690 | 5.928268762 |
| FLJ34969 | 8.348216992 |
| FLJ35220 | 6.001471573 |
| FLJ35258 | 5.826975502 |
| FLJ35424 | 5.803867049 |
| FLJ35725 | 6.355678367 |
| FLJ35740 | 5.835286354 |
| FLJ35767 | 8.315942561 |
| FLJ35773 | 6.005991191 |
| FLJ35801 | 7.57963028 |
| FLJ36070 | 5.679287483 |
| FLJ36144 | 5.846965104 |
| FLJ36445 | 6.877557084 |
| FLJ36492 | 5.619078212 |
| FLJ37078 | 5.805621864 |
| FLJ37307 | 5.732876926 |
| FLJ37464 | 7.375771605 |
| FLJ37543 | 6.057955517 |
| FLJ37970 | 5.93626053 |
| FLJ38377 | 5.913465905 |
| FLJ38451 | 6.459915114 |
| FLJ38482 | 8.642274211 |
| FLJ38717 | 6.964388243 |
| FLJ38969 | 5.712515298 |
| FLJ38973 | 7.112089015 |
| FLJ38991 | 5.970618452 |
| FLJ39080 | 5.624409121 |
| FLJ39501 | 8.165288467 |
| FLJ39743 | 7.437287939 |
| FLJ39779 | 6.124731481 |
| FLJ39827 | 7.37087015 |
| FLJ40142 | 6.168259421 |
| FLJ40243 | 6.313053863 |
| FLJ40288 | 6.060661592 |
| FLJ40722 | 8.7464658 |
| FLJ40852 | 5.639259275 |
| FLJ41046 | 5.729840043 |
| FLJ41131 | 6.179433366 |
| FLJ41200 | 5.591736605 |
| FLJ41603 | 5.689132581 |
| FLJ42133 | 6.219120016 |
| FLJ42875 | 5.98373275 |
| FLJ43093 | 7.498723884 |
| FLJ43276 | 5.541206014 |
| FLJ43374 | 5.859817522 |
| FLJ43692 | 6.195982401 |
| FLJ43855 | 5.975436879 |
| FLJ43870 | 7.360628247 |
| FLJ43980 | 5.739776901 |
| FLJ44216 | 7.153877284 |
| FLJ44477 | 5.834545447 |
| FLJ44606 | 5.572060358 |
| FLJ44790 | 5.973856329 |
| FLJ44817 | 5.804921879 |
| FLJ45139 | 8.464067299 |
| FLJ45187 | 6.694408091 |
| FLJ45202 | 6.003146379 |
| FLJ45224 | 5.706867986 |
| FLJ45248 | 7.529634925 |
| FLJ45337 | 5.70224106 |
| FLJ45537 | 5.55915218 |
| FLJ45717 | 5.708078076 |
| FLJ45832 | 5.679565952 |
| FLJ45909 | 8.296495534 |
| FLJ45910 | 5.977271396 |
| FLJ45964 | 5.548303186 |
| FLJ45983 | 6.32822479 |
| FLJ46072 | 8.183252295 |
| FLJ46300 | 5.667851281 |
| FLJ46347 | 6.277787161 |
| FLJ46836 | 6.16200948 |
| FLJ46906 | 7.603792717 |
| FLJ90086 | 6.455297825 |
| FLJ90757 | 5.881419097 |
| FLJ90805 | 5.745346425 |
| FLNA | 6.972373175 |
| FLNB | 8.792178757 |
| FLNC | 5.82636252 |
| FLOT1 | 11.20404018 |
| FLOT2 | 13.16014932 |
| FLRT2 | 6.388890706 |
| FLRT3 | 10.35592368 |
| FLT1 | 6.007022341 |
| FLT3LG | 5.919564259 |
| FLT4 | 5.964147604 |
| FLVCR1 | 8.593909966 |
| FLVCR2 | 8.481383105 |
| FLYWCH1 | 5.896976676 |
| FLYWCH2 | 7.169249913 |
| FMNL1 | 5.899361089 |
| FMNL2 | 6.302847608 |
| FMO2 | 6.329110815 |
| FMO3 | 12.42598567 |
| FMO4 | 7.828342102 |
| FMO5 | 11.06510186 |
| FMOD | 6.172816234 |
| FN1 | 6.258554546 |
| FN3KRP | 10.46146197 |
| FNBP1 | 9.722409679 |
| FNBP1L | 9.750996842 |
| FNBP4 | 7.507620318 |
| FNDC3A | 10.20688527 |
| FNDC3B | 11.23413408 |
| FNDC4 | 11.26175114 |
| FNDC5 | 8.13967239 |
| FNTA | 10.16691513 |
| FNTB | 8.487673083 |
| FOLH1 | 7.104249301 |
| FOLR2 | 8.814877465 |
| FOLR3 | 6.386925306 |
| FOS | 10.14669526 |
| FOSB | 6.944697218 |
| FOSL1 | 5.46774451 |
| FOSL2 | 8.143498949 |
| FOXA1 | 11.29316546 |
| FOXA2 | 11.40599464 |
| FOXA3 | 11.88031195 |
| FOXC1 | 6.343775788 |
| FOXD2 | 5.802803946 |
| FOXD4L4 | 10.00842818 |
| FOXF1 | 5.671913264 |
| FOXG1 | 7.605631222 |
| FOXH1 | 5.780012721 |
| FOXI1 | 5.919718433 |
| FOXJ2 | 8.96551616 |
| FOXJ3 | 8.635504145 |
| FOXL2 | 5.702060227 |
| FOXO1A | 9.921434277 |
| FOXO3A | 11.37030865 |
| FOXP1 | 7.118693994 |
| FOXP3 | 6.062313429 |
| FOXQ1 | 9.038777567 |
| FOXR1 | 5.722786926 |
| FOXRED1 | 10.21359092 |
| FPGS | 5.856804086 |
| FPR1 | 10.8037832 |
| FPR2 | 6.798270525 |
| FPR3 | 7.5652441 |
| FRAG1 | 9.863767163 |
| FRAP1 | 9.628703399 |
| FRAS1 | 5.589761974 |
| FRAT1 | 6.186430782 |
| FRAT2 | 9.018421444 |
| FRG1 | 11.25482431 |
| FRK | 6.772078488 |
| FRMD3 | 6.248517438 |
| FRMD4A | 7.455393031 |
| FRMD4B | 7.478355829 |
| FRMD6 | 9.283943646 |
| FRRS1 | 5.92809826 |
| FRS3 | 6.380282768 |
| FRY | 6.738412238 |
| FRYL | 7.563806248 |
| FRZB | 7.068451732 |
| FSCB | 5.835506 |
| FSCN1 | 11.49451172 |
| FSD1CL | 6.282098574 |
| FSHB | 5.86057504 |
| FST | 9.471613887 |
| FSTL1 | 10.38061033 |
| FSTL3 | 6.559450676 |
| FTCD | 10.51162184 |
| FTH1 | 11.11502784 |
| FTHL11 | 11.87980261 |
| FTHL12 | 12.64131959 |
| FTHL2 | 12.86087146 |
| FTHL8 | 12.48216432 |
| FTL | 15.56335736 |
| FTSJ1 | 9.075212133 |
| FTSJ2 | 7.945269337 |
| FTSJ3 | 7.024910258 |
| FUBP1 | 6.378464642 |
| FUBP3 | 10.53144119 |
| FUCA1 | 11.46542668 |
| FUCA2 | 9.928648656 |
| FUK | 9.018918582 |
| FUNDC1 | 8.622045068 |
| FUNDC2 | 6.722338707 |
| FURIN | 9.182090185 |
| FUSIP1 | 8.608926372 |
| FUT11 | 5.883314127 |
| FUT2 | 5.49672915 |
| FUT3 | 6.085182783 |
| FUT4 | 7.415255705 |
| FUT5 | 5.694124448 |
| FUT6 | 7.035042487 |
| FUT8 | 6.583959032 |
| FUZ | 5.581219366 |
| FVT1 | 8.825007686 |
| FXC1 | 8.642215959 |
| FXN | 5.847599043 |
| FXR1 | 9.769456251 |
| FXR2 | 6.991239349 |
| FXYD1 | 8.50356907 |
| FXYD2 | 6.095869572 |
| FXYD5 | 9.407772838 |
| FXYD6 | 7.864667865 |
| FYB | 8.336708306 |
| FYCO1 | 9.026699639 |
| FYN | 7.316810467 |
| FYTTD1 | 8.757836972 |
| FZD1 | 6.122659347 |
| FZD4 | 9.574047852 |
| FZD5 | 8.052091864 |
| FZD6 | 6.810755067 |
| FZD8 | 5.737564902 |
| FZD9 | 5.86976263 |
| FZR1 | 5.906474044 |
| G0S2 | 10.09807291 |
| G1P3 | 12.1435854 |
| G3BP1 | 13.21834611 |
| G3BP2 | 9.238509771 |
| G6PC | 10.52830773 |
| G6PC3 | 9.657984947 |
| GAA | 7.408034137 |
| GAB1 | 6.013989454 |
| GAB2 | 7.413478512 |
| GABARAP | 8.708681758 |
| GABARAPL1 | 12.4749446 |
| GABARAPL2 | 12.08776066 |
| GABBR1 | 5.812074651 |
| GABPA | 7.09156996 |
| GABPB2 | 7.915326819 |
| GABRB3 | 6.07048298 |
| GABRE | 6.844747976 |
| GABRG2 | 5.84296946 |
| GAD1 | 5.721994702 |
| GADD45A | 9.540157437 |
| GADD45B | 10.80777711 |
| GADD45G | 10.07057595 |
| GADD45GIP1 | 8.277108913 |
| GAK | 10.60726639 |
| GAL3ST1 | 5.836098625 |
| GAL3ST4 | 6.26598449 |
| GALC | 8.602999903 |
| GALE | 10.38192861 |
| GALK1 | 12.92801044 |
| GALK2 | 7.469607366 |
| GALM | 9.843498866 |
| GALNAC4S-6ST | 9.236387621 |
| GALNS | 6.075700346 |
| GALNT1 | 9.261368071 |
| GALNT10 | 5.693973717 |
| GALNT11 | 9.45418605 |
| GALNT14 | 5.646246436 |
| GALNT2 | 7.03332532 |
| GALNT3 | 6.156724245 |
| GALNT4 | 8.280806957 |
| GALNT6 | 5.867895181 |
| GALNTL4 | 8.415795817 |
| GALR2 | 5.912579612 |
| GALT | 9.611248107 |
| GAMT | 10.44667471 |
| GAN | 5.700057908 |
| GANAB | 10.40817164 |
| GAPDH | 13.09837593 |
| GAPVD1 | 9.513250247 |
| GARNL1 | 7.148945479 |
| GARNL3 | 5.677608411 |
| GARS | 12.33415197 |
| GART | 8.062106397 |
| GAS1 | 7.063674942 |
| GAS2 | 9.755233853 |
| GAS2L3 | 5.749921559 |
| GAS5 | 5.594954033 |
| GAS6 | 8.80655364 |
| GAS7 | 6.175561469 |
| GAS8 | 6.512764196 |
| GATA2 | 5.929079293 |
| GATA4 | 6.578303355 |
| GATA6 | 6.205311467 |
| GATAD1 | 7.401811149 |
| GATAD2A | 10.20646727 |
| GATAD2B | 5.974898499 |
| GATC | 7.859938672 |
| GATM | 13.03432655 |
| GATS | 5.958419526 |
| GBA | 8.394199801 |
| GBA2 | 7.681572587 |
| GBA3 | 10.59670706 |
| GBAP | 5.754689419 |
| GBAS | 8.894518393 |
| GBE1 | 10.57083011 |
| GBF1 | 7.110934147 |
| GBL | 7.755486879 |
| GBP1 | 9.806461663 |
| GBP2 | 11.04852647 |
| GBP3 | 6.551765764 |
| GBP4 | 9.055986536 |
| GBP5 | 5.891340478 |
| GBP7 | 9.586101325 |
| GC | 15.42374918 |
| GCA | 11.04034213 |
| GCAT | 9.698958052 |
| GCC1 | 9.467267094 |
| GCC2 | 6.048116422 |
| GCDH | 11.17296753 |
| GCET2 | 6.796337561 |
| GCGR | 11.92806567 |
| GCH1 | 8.798919179 |
| GCHFR | 12.80829056 |
| GCKR | 8.604501715 |
| GCLC | 10.62915517 |
| GCLM | 10.35956932 |
| GCN1L1 | 9.968113326 |
| GCN5L2 | 8.765791498 |
| GCNT1 | 6.427857504 |
| GCNT2 | 7.303222997 |
| GCNT3 | 6.452406915 |
| GCNT4 | 8.750075335 |
| GCS1 | 7.841119762 |
| GCUD2 | 5.611320447 |
| GDA | 6.214038683 |
| GDAP2 | 6.752608692 |
| GDE1 | 8.568030197 |
| GDF11 | 5.938814479 |
| GDF15 | 8.702250004 |
| GDF2 | 6.437317458 |
| GDF5 | 5.633616938 |
| GDI1 | 7.475933906 |
| GDI2 | 12.67248266 |
| GDPD1 | 5.819904444 |
| GDPD4 | 6.093750514 |
| GDPD5 | 6.539656292 |
| GEFT | 5.738773835 |
| GEM | 6.429124979 |
| GEMIN4 | 9.909979803 |
| GEMIN5 | 6.663002182 |
| GEMIN6 | 10.5719988 |
| GENX-3414 | 9.547075865 |
| GFM1 | 11.56240281 |
| GFM2 | 6.545159182 |
| GFOD1 | 9.061070095 |
| GFOD2 | 10.04111289 |
| GFPT1 | 10.93810763 |
| GFPT2 | 6.336550463 |
| GFRA1 | 7.304836204 |
| GFRA2 | 8.040818083 |
| GFRA4 | 5.673721941 |
| GGA2 | 8.29397528 |
| GGA3 | 6.756464225 |
| GGCT | 9.195467045 |
| GGCX | 10.33422587 |
| GGH | 13.11390782 |
| GGNBP2 | 10.10036022 |
| GGPS1 | 10.04024393 |
| GGT1 | 5.843763591 |
| GGT2 | 6.141196198 |
| GGTL3 | 5.525677884 |
| GGTLA1 | 6.371177588 |
| GHDC | 7.57925625 |
| GHITM | 13.58070479 |
| GHR | 12.74716243 |
| GHRHR | 6.017838553 |
| GHSR | 5.705346985 |
| GIF | 5.870663963 |
| GIMAP1 | 7.085565446 |
| GIMAP2 | 7.898254851 |
| GIMAP4 | 11.10818007 |
| GIMAP5 | 9.092718554 |
| GIMAP6 | 8.264173793 |
| GIMAP7 | 9.377382412 |
| GIMAP8 | 9.37544779 |
| GINS2 | 8.979631871 |
| GINS3 | 7.806367139 |
| GINS4 | 6.017884129 |
| GIPC1 | 6.860433756 |
| GIPC2 | 5.911887355 |
| GIPR | 6.076334153 |
| GIT1 | 5.714027578 |
| GIT2 | 5.835906526 |
| GIYD1 | 6.594011908 |
| GIYD2 | 7.374708769 |
| GJA1 | 8.132283712 |
| GJA4 | 9.117536522 |
| GJA5 | 6.097233174 |
| GJA7 | 5.775141859 |
| GJB1 | 10.37083731 |
| GJB2 | 11.77713652 |
| GJB3 | 6.625451497 |
| GJB4 | 5.800166862 |
| GJC1 | 6.012478529 |
| GJC2 | 6.191609424 |
| GK | 10.76129323 |
| GKAP1 | 6.425943979 |
| GLA | 10.4746084 |
| GLB1 | 8.29119138 |
| GLB1L | 7.18767001 |
| GLCCI1 | 5.905267385 |
| GLCE | 10.09917447 |
| GLDC | 11.6893298 |
| GLDN | 5.598650372 |
| GLE1 | 8.555255959 |
| GLG1 | 11.61977508 |
| GLIPR1 | 8.860739146 |
| GLIPR1L1 | 5.637421841 |
| GLIPR1L2 | 5.696086621 |
| GLIPR2 | 9.628404235 |
| GLIS1 | 5.630655082 |
| GLMN | 8.324406938 |
| GLO1 | 12.3551331 |
| GLOD4 | 10.75885108 |
| GLRA1 | 6.470069065 |
| GLRB | 5.96574064 |
| GLRX | 13.23727909 |
| GLRX2 | 8.169712741 |
| GLRX5 | 13.59004292 |
| GLS | 6.154599916 |
| GLS2 | 7.958532042 |
| GLT1D1 | 8.53477468 |
| GLT25D1 | 10.8569029 |
| GLT8D1 | 7.57257279 |
| GLT8D2 | 6.057229085 |
| GLTP | 11.41132109 |
| GLTPD1 | 5.921260979 |
| GLTPD2 | 7.935195419 |
| GLTSCR1 | 7.524336622 |
| GLTSCR2 | 12.87543694 |
| GLUD1 | 13.9137581 |
| GLUD2 | 6.562452581 |
| GLUL | 6.285838078 |
| GLYAT | 8.879495648 |
| GLYATL1 | 9.054653226 |
| GLYATL2 | 5.894573992 |
| GLYCTK | 10.07824073 |
| gm127 | 6.48063106 |
| GM2A | 10.55528208 |
| GMCL1 | 8.471344196 |
| GMDS | 10.9119885 |
| GMEB1 | 6.813031536 |
| GMEB2 | 7.342661337 |
| GMFB | 11.20354467 |
| GMFG | 10.68632216 |
| GMIP | 7.76137778 |
| GMNN | 9.08768158 |
| GMPPA | 7.751466725 |
| GMPPB | 7.653628556 |
| GMPR | 6.940947815 |
| GMPR2 | 7.638904844 |
| GMPS | 10.44718153 |
| GNA11 | 11.71286862 |
| GNA13 | 9.81953509 |
| GNA14 | 7.105177462 |
| GNA15 | 6.879606548 |
| GNAI1 | 6.490628482 |
| GNAI2 | 10.6787409 |
| GNAI3 | 9.301853833 |
| GNAO1 | 6.709478766 |
| GNAQ | 6.780654115 |
| GNAS | 13.25884348 |
| GNAT1 | 5.899353343 |
| GNAT2 | 5.810438746 |
| GNAZ | 5.914955013 |
| GNB1 | 12.48696741 |
| GNB1L | 6.330558885 |
| GNB2 | 8.287091114 |
| GNB2L1 | 15.06263325 |
| GNB3 | 5.571694206 |
| GNB4 | 7.034221323 |
| GNB5 | 5.924519204 |
| GNE | 13.08273323 |
| GNG10 | 11.50852603 |
| GNG11 | 11.50736351 |
| GNG12 | 6.061486933 |
| GNG13 | 6.53708938 |
| GNG2 | 6.901911562 |
| GNG5 | 9.873538331 |
| GNG7 | 5.882572302 |
| GNGT1 | 5.833767514 |
| GNL1 | 8.842400367 |
| GNL2 | 10.79904393 |
| GNL3 | 8.623320946 |
| GNL3L | 9.25934893 |
| GNLY | 6.15604292 |
| GNMT | 9.332978475 |
| GNPAT | 10.12669873 |
| GNPDA1 | 10.26861524 |
| GNPDA2 | 7.643110516 |
| GNPNAT1 | 10.19386965 |
| GNPTAB | 7.618938056 |
| GNPTG | 10.48954369 |
| GNRH1 | 5.701260373 |
| GNRHR2 | 6.118802978 |
| GNS | 12.70527728 |
| GOLGA1 | 7.132821167 |
| GOLGA2 | 8.451859971 |
| GOLGA2L1 | 6.032304201 |
| GOLGA3 | 10.07413296 |
| GOLGA4 | 8.381952697 |
| GOLGA5 | 10.87587779 |
| GOLGA7 | 7.987354408 |
| GOLGB1 | 9.000030147 |
| GOLM1 | 7.974648962 |
| GOLPH3 | 12.40080053 |
| GOLPH3L | 9.112804011 |
| GOLPH4 | 8.934458533 |
| GOLT1A | 6.490724389 |
| GOLT1B | 10.21610908 |
| GOPC | 7.728554671 |
| GORASP1 | 8.531479835 |
| GORASP2 | 10.7996115 |
| GOSR1 | 9.000394355 |
| GOSR2 | 7.992747999 |
| GOT1 | 13.62488916 |
| GOT2 | 12.23636311 |
| GP1BA | 8.700956539 |
| GP1BB | 7.43115927 |
| GP6 | 5.532149808 |
| GP9 | 5.739605122 |
| GPA33 | 5.675966333 |
| GPAA1 | 8.379575583 |
| GPAM | 11.22701237 |
| GPATCH1 | 6.536816334 |
| GPATCH2 | 6.991643787 |
| GPATCH3 | 7.629884682 |
| GPATCH4 | 8.884325424 |
| GPBAR1 | 6.203166177 |
| GPBP1 | 9.363540809 |
| GPBP1L1 | 8.58727823 |
| GPC1 | 6.225956709 |
| GPC2 | 5.512775967 |
| GPC3 | 5.412197973 |
| GPC4 | 5.570145924 |
| GPC5 | 5.610064291 |
| GPC6 | 9.644300903 |
| GPD1 | 9.38355156 |
| GPD1L | 8.772776454 |
| GPHA2 | 5.704209639 |
| GPHN | 7.605743651 |
| GPI | 11.12974285 |
| GPIHBP1 | 6.912152405 |
| GPKOW | 10.09901048 |
| GPLD1 | 7.971393198 |
| GPM6A | 7.541739421 |
| GPNMB | 8.645346958 |
| GPR108 | 8.705774436 |
| GPR109B | 7.538366098 |
| GPR110 | 5.808138283 |
| GPR111 | 5.43786171 |
| GPR112 | 5.716384024 |
| GPR113 | 6.052979103 |
| GPR116 | 10.35591757 |
| GPR12 | 5.616633279 |
| GPR124 | 6.095651688 |
| GPR125 | 5.906887446 |
| GPR126 | 9.270059445 |
| GPR128 | 8.24562531 |
| GPR137 | 10.23837543 |
| GPR137B | 9.790764884 |
| GPR143 | 6.167799764 |
| GPR146 | 6.47518219 |
| GPR149 | 5.699472339 |
| GPR15 | 6.899517148 |
| GPR155 | 5.876736304 |
| GPR156 | 6.013770285 |
| GPR160 | 6.148625995 |
| GPR161 | 5.664099525 |
| GPR162 | 7.931444795 |
| GPR171 | 6.582103304 |
| GPR172A | 8.654225683 |
| GPR175 | 7.724895839 |
| GPR176 | 5.478674457 |
| GPR177 | 7.870185017 |
| GPR182 | 7.399537189 |
| GPR24 | 6.313538607 |
| GPR26 | 5.430735729 |
| GPR3 | 6.295646984 |
| GPR30 | 7.834438174 |
| GPR34 | 7.587387573 |
| GPR35 | 5.57512353 |
| GPR37 | 8.286154283 |
| GPR4 | 6.205537752 |
| GPR45 | 5.99442268 |
| GPR56 | 7.22904402 |
| GPR64 | 5.606664245 |
| GPR65 | 7.1215874 |
| GPR84 | 5.891761562 |
| GPR88 | 7.680032878 |
| GPR89A | 7.830563344 |
| GPRASP1 | 6.105577716 |
| GPRASP2 | 8.770981471 |
| GPRC5B | 6.232480538 |
| GPRC5C | 7.126273146 |
| GPS1 | 8.85741292 |
| GPS2 | 7.995797635 |
| GPSM1 | 6.793641437 |
| GPSM2 | 6.026682952 |
| GPSM3 | 8.19000408 |
| GPSN2 | 11.42694387 |
| GPT | 11.15468256 |
| GPT2 | 11.69160497 |
| GPX1 | 13.43974866 |
| GPX2 | 14.28018625 |
| GPX3 | 12.98709309 |
| GPX4 | 12.77647977 |
| GPX7 | 8.836090926 |
| GRAMD1A | 8.970448107 |
| GRAMD1C | 7.22040425 |
| GRAMD3 | 8.638197423 |
| GRAMD4 | 9.677657004 |
| GRASP | 5.869839293 |
| GRB10 | 7.222149068 |
| GRB14 | 7.539440198 |
| GRB2 | 8.914107855 |
| GREM1 | 5.736762008 |
| GREM2 | 7.361707001 |
| GRHL1 | 6.442653308 |
| GRHPR | 11.72048697 |
| GRIA2 | 5.700289022 |
| GRIA3 | 7.723326739 |
| GRIK1 | 5.75193495 |
| GRIK5 | 6.281429024 |
| GRIN2A | 6.056144207 |
| GRINA | 9.974843475 |
| GRINL1A | 6.831845691 |
| GRIP2 | 5.694072107 |
| GRIPAP1 | 6.869835461 |
| GRK5 | 6.268743276 |
| GRK6 | 6.22436183 |
| GRLF1 | 5.414436275 |
| GRM2 | 5.862785932 |
| GRM8 | 8.455041091 |
| GRN | 9.566226893 |
| GRP | 5.709540702 |
| GRPEL1 | 11.51633037 |
| GRPEL2 | 9.517778264 |
| GRSF1 | 10.1355147 |
| GRTP1 | 6.624075202 |
| GRWD1 | 9.170502556 |
| GSDM1 | 5.748355815 |
| GSDMDC1 | 12.30627481 |
| GSDML | 6.056383299 |
| GSG1 | 6.037128534 |
| GSG2 | 5.782963678 |
| GSK3B | 9.568806652 |
| GSN | 7.928806713 |
| GSPT1 | 12.76300401 |
| GSPT2 | 9.683235332 |
| GSR | 9.404047485 |
| GSS | 8.721907185 |
| GSTA1 | 12.03789827 |
| GSTA2 | 11.81805201 |
| GSTA4 | 8.899131501 |
| GSTA5 | 9.542892855 |
| GSTCD | 5.864722407 |
| GSTK1 | 12.49506087 |
| GSTM1 | 7.095053542 |
| GSTM3 | 6.846882531 |
| GSTM4 | 7.128290457 |
| GSTO1 | 14.28633978 |
| GSTO2 | 8.043339386 |
| GSTP1 | 11.26065903 |
| GSTT1 | 11.51668954 |
| GSTZ1 | 6.6783902 |
| GTDC1 | 6.384392807 |
| GTF2A1 | 5.707541949 |
| GTF2A2 | 11.69979192 |
| GTF2B | 9.8259617 |
| GTF2E1 | 8.18086815 |
| GTF2E2 | 11.7670386 |
| GTF2F1 | 8.78721485 |
| GTF2F2 | 10.46410392 |
| GTF2H1 | 7.951707564 |
| GTF2H2 | 6.408068514 |
| GTF2H3 | 8.199266163 |
| GTF2H4 | 8.344130735 |
| GTF2H5 | 11.69328413 |
| GTF2I | 5.687653062 |
| GTF2IP1 | 8.64407905 |
| GTF2IRD1 | 5.89746112 |
| GTF2IRD2 | 9.853443992 |
| GTF2IRD2B | 8.362488654 |
| GTF2IRD2P | 7.052404258 |
| GTF3A | 13.32343889 |
| GTF3C1 | 6.942759325 |
| GTF3C2 | 8.887447641 |
| GTF3C3 | 8.079757747 |
| GTF3C5 | 7.989208611 |
| GTF3C6 | 10.1392822 |
| GTPBP2 | 5.955300883 |
| GTPBP3 | 7.683877634 |
| GTPBP4 | 11.04002818 |
| GTPBP5 | 5.962693438 |
| GTPBP6 | 11.53752537 |
| GTPBP8 | 8.281038884 |
| GTSE1 | 7.161606273 |
| GTSF1 | 6.072836834 |
| GUCA1A | 6.065172899 |
| GUCA2B | 6.372868175 |
| GUCY1A3 | 8.612474697 |
| GUCY1B3 | 5.582048541 |
| GUF1 | 8.043282724 |
| GUK1 | 12.23845081 |
| GULP1 | 5.776162125 |
| GUSB | 11.71684095 |
| GUSBL1 | 6.755042059 |
| GUSBL2 | 7.261101716 |
| GVIN1 | 7.049764925 |
| GYG2 | 9.172135422 |
| GYPB | 5.523185706 |
| GYPC | 8.405622745 |
| GYPE | 5.592300851 |
| GYS1 | 5.522689431 |
| GYS2 | 7.886673713 |
| GZF1 | 5.718483444 |
| GZMA | 9.18828748 |
| GZMB | 7.444788522 |
| GZMH | 6.628996888 |
| GZMK | 8.202530081 |
| H19 | 11.96383617 |
| H1F0 | 6.057970089 |
| H1FX | 7.938639588 |
| H2AFJ | 10.8628322 |
| H2AFV | 6.447355448 |
| H2AFX | 7.412832227 |
| H2AFY | 9.248015584 |
| H2AFY2 | 8.615069287 |
| H2AFZ | 13.09022231 |
| H2BFS | 9.797909823 |
| H3F3A | 14.53724528 |
| H3F3B | 10.10922797 |
| H6PD | 9.325177802 |
| HAAO | 6.753512327 |
| HABP2 | 11.60002353 |
| HABP4 | 6.81364115 |
| HACE1 | 8.298969209 |
| HADH2 | 12.21104709 |
| HADHA | 9.570126469 |
| HADHSC | 12.53722124 |
| HAGH | 12.58296623 |
| HAK | 6.94022585 |
| HAL | 8.037547732 |
| HAMP | 15.3177685 |
| HAO1 | 10.82979571 |
| HAO2 | 9.481994241 |
| HAP1 | 5.529906143 |
| HAPLN2 | 5.788861212 |
| HAPLN4 | 5.840527123 |
| HARS | 10.22252271 |
| HARS2 | 7.610209535 |
| HAS1 | 5.762740807 |
| HAS2 | 6.10665105 |
| HAS3 | 6.177286113 |
| HAT1 | 9.327351454 |
| HAVCR2 | 10.93091043 |
| HAX1 | 11.68100392 |
| HBA1 | 12.14908593 |
| HBB | 13.86173241 |
| HBD | 8.252277063 |
| HBG1 | 6.220348709 |
| HBG2 | 6.385735766 |
| HBP1 | 9.566184465 |
| HBXIP | 6.410249684 |
| HCCA2 | 7.34503695 |
| HCCS | 6.981277671 |
| HCFC1 | 9.93702464 |
| HCFC1R1 | 8.869500351 |
| HCFC2 | 8.121188313 |
| HCG18 | 5.893838433 |
| HCG4 | 5.68140859 |
| HCG4P6 | 5.738404863 |
| HCK | 8.263853936 |
| HCLS1 | 9.808208 |
| HCP1 | 7.27124144 |
| HCP5 | 10.57045389 |
| HCRTR2 | 6.307353572 |
| HCST | 10.29603807 |
| HCTSL-s | 5.69295347 |
| HD | 6.254523688 |
| HDAC1 | 11.89685032 |
| HDAC11 | 6.580442205 |
| HDAC2 | 10.41258306 |
| HDAC3 | 10.0452402 |
| HDAC4 | 7.308976892 |
| HDAC6 | 8.716026054 |
| HDAC7A | 6.069189308 |
| HDAC8 | 6.789937685 |
| HDAC9 | 5.938355868 |
| HDC | 7.106217603 |
| HDCMA18P | 9.197557562 |
| HDDC2 | 11.30421432 |
| HDDC3 | 10.39898829 |
| HDGF | 9.592388282 |
| HDGF2 | 6.510367846 |
| HDGFRP3 | 5.905404171 |
| HDHD1A | 8.508796115 |
| HDHD2 | 10.76996277 |
| HDHD3 | 9.040172295 |
| HDLBP | 5.837892741 |
| HEATR1 | 9.601691645 |
| HEATR2 | 10.38630568 |
| HEATR5B | 6.88379303 |
| HEBP1 | 11.63440151 |
| HEBP2 | 12.91657879 |
| HECA | 6.632410399 |
| HECTD1 | 10.21211152 |
| HECTD2 | 6.288652241 |
| HECTD3 | 6.252450075 |
| HECW2 | 8.387772179 |
| HEG1 | 8.058064634 |
| HEL308 | 7.103528101 |
| HELB | 6.038709947 |
| HELZ | 7.768139422 |
| HEM1 | 8.028357678 |
| HEMGN | 6.354364978 |
| HEPH | 6.075946555 |
| HERC1 | 7.742760627 |
| HERC2 | 7.675859698 |
| HERC2P4 | 5.911835679 |
| HERC3 | 6.216354673 |
| HERC4 | 8.259613559 |
| HERC5 | 9.516247149 |
| HERC6 | 7.503083752 |
| HERPUD1 | 12.01255563 |
| HERPUD2 | 8.402256596 |
| HES1 | 8.376898889 |
| HES4 | 8.931614641 |
| HES5 | 5.738862092 |
| HES6 | 8.363463672 |
| HEXB | 12.87108639 |
| HEXDC | 7.261578738 |
| HEXIM1 | 6.884980599 |
| HEXIM2 | 6.325419153 |
| HEY1 | 7.703296444 |
| HEY2 | 6.559123308 |
| HEYL | 7.709507658 |
| HFE | 5.638398937 |
| HFE2 | 8.866976354 |
| HGD | 10.26793786 |
| HGF | 6.268293771 |
| HGFAC | 10.10063544 |
| HGS | 10.35417324 |
| HHAT | 5.872920028 |
| HHEX | 9.908141092 |
| HHIP | 6.614229387 |
| HHLA1 | 5.755727297 |
| HHLA3 | 5.970872278 |
| HIAT1 | 8.773662987 |
| HIATL1 | 8.321550161 |
| HIATL2 | 6.47120893 |
| HIBADH | 12.58126805 |
| HIBCH | 11.43614746 |
| HIC2 | 7.234418514 |
| HIF1A | 8.103381911 |
| HIF1AN | 6.658826466 |
| HIF3A | 5.812351419 |
| HIG2 | 8.805471615 |
| HIGD1A | 14.16654433 |
| HIGD2A | 11.96015728 |
| HILS1 | 5.857008956 |
| HINT1 | 14.81463451 |
| HINT2 | 12.17350186 |
| HINT3 | 8.109154944 |
| HIP1 | 6.089695614 |
| HIP1R | 8.991065477 |
| HIP2 | 10.4608696 |
| HIPK1 | 5.909075482 |
| HIPK2 | 10.90553386 |
| HIRA | 7.368215099 |
| HIRIP3 | 8.975260863 |
| HIST1H1C | 12.11261328 |
| HIST1H2AC | 10.05001961 |
| HIST1H2AH | 5.416573145 |
| HIST1H2AJ | 5.922650489 |
| HIST1H2AK | 6.016224435 |
| HIST1H2AL | 7.727102144 |
| HIST1H2AM | 5.880525852 |
| HIST1H2BC | 7.912025727 |
| HIST1H2BD | 9.841865735 |
| HIST1H2BE | 7.707847594 |
| HIST1H2BG | 5.42606761 |
| HIST1H2BJ | 5.753738248 |
| HIST1H2BK | 13.02771869 |
| HIST1H2BO | 6.043981108 |
| HIST1H3D | 6.158083667 |
| HIST1H3F | 5.98936688 |
| HIST1H3G | 6.080359528 |
| HIST1H3I | 6.022616404 |
| HIST1H4C | 14.79097702 |
| HIST1H4H | 5.632703948 |
| HIST1H4K | 9.627058161 |
| HIST2H2AA3 | 10.79844025 |
| HIST2H2AB | 5.628049654 |
| HIST2H2AC | 10.74163927 |
| HIST2H2BE | 7.517821315 |
| HIST2H4A | 7.22446324 |
| HIST3H2A | 5.678040908 |
| HIT-40 | 5.827071191 |
| HIVEP1 | 7.786764202 |
| HIVEP2 | 6.766529522 |
| HK1 | 9.988184247 |
| HK2 | 6.051594757 |
| HK3 | 7.347022315 |
| HKDC1 | 5.969676832 |
| HKR1 | 6.726385833 |
| HLA-A | 12.420899 |
| HLA-B | 12.35464965 |
| HLA-C | 8.658693304 |
| HLA-DMA | 11.0875146 |
| HLA-DMB | 10.87987359 |
| HLA-DOA | 5.996418764 |
| HLA-DPA1 | 11.0537688 |
| HLA-DPB1 | 6.488862865 |
| HLA-DPB2 | 5.745998313 |
| HLA-DQA1 | 9.918943634 |
| HLA-DQB1 | 6.29152097 |
| HLA-DRA | 12.25562112 |
| HLA-DRB1 | 9.142189831 |
| HLA-DRB3 | 9.463126167 |
| HLA-DRB4 | 11.09482598 |
| HLA-DRB5 | 8.088138929 |
| HLA-E | 12.47599041 |
| HLA-F | 9.047785223 |
| HLA-G | 6.225649815 |
| HLA-H | 10.13789437 |
| HLCS | 7.288667628 |
| HLF | 9.341062499 |
| HLX | 6.304190382 |
| HLXB9 | 5.99186068 |
| HM13 | 6.878817919 |
| HMBOX1 | 7.502428877 |
| HMBS | 10.05207652 |
| HMFN0672 | 6.760559934 |
| HMG1L1 | 10.49680884 |
| HMG20A | 8.147245073 |
| HMG20B | 5.75371364 |
| HMGA1 | 6.028949327 |
| HMGB1 | 7.65092474 |
| HMGB2 | 6.888229951 |
| HMGB3 | 8.644505819 |
| HMGCL | 12.48037191 |
| HMGCR | 10.29016197 |
| HMGCS1 | 12.17969615 |
| HMGCS2 | 13.86332549 |
| HMGN1 | 13.02189966 |
| HMGN2 | 7.435248439 |
| HMGN3 | 6.482244436 |
| HMGN4 | 9.678532568 |
| HMHA1 | 6.839128624 |
| HMMR | 5.917179849 |
| HMOX1 | 11.24410296 |
| HMOX2 | 5.660362483 |
| HN1 | 8.722726893 |
| HN1L | 7.585639565 |
| HNF4A | 6.309143543 |
| HNF4G | 8.976691144 |
| HNMT | 8.618630383 |
| HNRNPA0 | 10.17661537 |
| HNRNPAB | 11.08183285 |
| HNRNPD | 11.86588271 |
| HNRNPH2 | 6.477090916 |
| HNRNPL | 9.557433157 |
| HNRNPU | 5.711172213 |
| HNRPA1 | 6.162088856 |
| HNRPA2B1 | 12.31542788 |
| HNRPA3 | 10.11926234 |
| HNRPC | 12.63231001 |
| HNRPDL | 8.558344663 |
| HNRPF | 8.315304125 |
| HNRPH1 | 10.54928332 |
| HNRPH3 | 8.627062987 |
| HNRPK | 7.835568029 |
| HNRPLL | 8.741121914 |
| HNRPM | 8.9775128 |
| HNRPR | 11.36882339 |
| HNRPUL1 | 9.840228877 |
| HNRPUL2 | 6.375806578 |
| HOMER2 | 10.4569035 |
| HOMER3 | 5.767797411 |
| HOMEZ | 7.41924208 |
| HOM-TES-103 | 6.239002703 |
| HOOK1 | 11.43962236 |
| HOOK2 | 7.297814563 |
| HOOK3 | 6.533850473 |
| HOPX | 6.426878343 |
| HORMAD2 | 5.365452766 |
| HOXA5 | 6.340665579 |
| HOXA6 | 6.276442912 |
| HOXB1 | 5.984276098 |
| HOXB2 | 6.458379353 |
| HOXB4 | 5.845691384 |
| HOXB5 | 7.1794095 |
| HOXB6 | 5.870879308 |
| HOXB9 | 6.533021795 |
| HOXC13 | 8.065194259 |
| HOXC5 | 5.409010929 |
| HOXC6 | 5.845706891 |
| HOXD3 | 6.73333426 |
| HP | 15.24586988 |
| HP1BP3 | 6.211919654 |
| HPCAL1 | 8.687107965 |
| HPD | 12.58573723 |
| HPN | 11.66392507 |
| HPR | 12.69370695 |
| HPRT1 | 9.887960731 |
| HPS1 | 6.431491071 |
| HPS3 | 5.800186712 |
| HPS5 | 9.976504831 |
| HPS6 | 9.805920512 |
| HPSE | 6.453364103 |
| HPX | 15.18857187 |
| HRAS | 8.049440105 |
| HRASLS | 5.519983665 |
| HRASLS2 | 6.027289119 |
| HRASLS3 | 13.14126313 |
| HRB | 7.591527862 |
| HRB2 | 7.315356113 |
| HRBL | 7.598205749 |
| HRES1 | 5.816247199 |
| HRG | 13.48019828 |
| HRH2 | 6.229087937 |
| HRIHFB2122 | 7.190661595 |
| HRSP12 | 13.43865084 |
| HS1BP3 | 8.373003088 |
| HS2ST1 | 7.852164735 |
| HS3ST3A1 | 6.700780356 |
| HS3ST5 | 5.587446254 |
| HS3ST6 | 5.662747669 |
| HSBP1 | 11.22359545 |
| HSCB | 10.17278261 |
| HSD11B1 | 13.04008757 |
| HSD11B2 | 6.079504523 |
| HSD17B11 | 11.90312312 |
| HSD17B12 | 11.82284462 |
| HSD17B13 | 9.158942076 |
| HSD17B14 | 6.974469448 |
| HSD17B2 | 12.67844456 |
| HSD17B3 | 5.679950567 |
| HSD17B4 | 12.75916058 |
| HSD17B6 | 13.69785658 |
| HSD17B7 | 11.36546668 |
| HSD17B8 | 11.41757169 |
| HSD3B7 | 9.488711775 |
| HSDL1 | 7.1351511 |
| HSDL2 | 11.11947974 |
| HSF2 | 8.779071471 |
| HSGT1 | 7.005825464 |
| HSP90AA1 | 9.391408493 |
| HSP90AB1 | 12.01170152 |
| HSP90B1 | 13.58356123 |
| HSPA12A | 6.019822424 |
| HSPA12B | 6.586802524 |
| HSPA13 | 6.043822141 |
| HSPA14 | 9.386985821 |
| HSPA1A | 11.07235863 |
| HSPA1B | 9.867614465 |
| HSPA1L | 5.759156561 |
| HSPA2 | 6.199824863 |
| HSPA4 | 7.543563688 |
| HSPA4L | 7.747234378 |
| HSPA5 | 11.95597946 |
| HSPA6 | 6.522797791 |
| HSPA8 | 13.36842105 |
| HSPA9B | 13.02268724 |
| HSPB1 | 12.87761669 |
| HSPB2 | 5.757365651 |
| HSPB6 | 6.269683352 |
| HSPB7 | 5.715797967 |
| HSPB8 | 6.631589397 |
| HSPB9 | 5.958048901 |
| HSPBAP1 | 7.21965777 |
| HSPBP1 | 5.830091417 |
| HSPC023 | 11.34035508 |
| HSPC105 | 6.324677938 |
| HSPC111 | 10.43900036 |
| HSPC148 | 11.60219186 |
| HSPC152 | 12.96552056 |
| HSPC159 | 7.946268566 |
| HSPC171 | 11.45083239 |
| HSPC268 | 9.964221882 |
| HSPCAL3 | 6.896524736 |
| HSPD1 | 9.798674285 |
| HSPE1 | 14.19832299 |
| HSPG2 | 5.669472586 |
| HSPH1 | 10.2689018 |
| HSU79303 | 10.23938401 |
| HSZFP36 | 8.010827421 |
| HTATIP2 | 9.429323762 |
| HTATSF1 | 8.362837644 |
| HTLF | 10.33574072 |
| HTR1D | 5.784083147 |
| HTR1E | 5.789384612 |
| HTR2B | 6.909941079 |
| HTR6 | 5.670390264 |
| HTRA1 | 12.06571955 |
| HTRA2 | 8.176190581 |
| HTRA4 | 6.187686626 |
| HUS1 | 6.181211367 |
| HUS1B | 5.866011774 |
| HUWE1 | 6.216572764 |
| HYAL1 | 9.512113694 |
| HYAL2 | 9.149884047 |
| HYAL3 | 5.966234744 |
| HYAL4 | 5.842978554 |
| HYI | 7.573791593 |
| HYLS1 | 7.820586898 |
| HYOU1 | 9.58294505 |
| HYPB | 9.256949428 |
| HYPK | 5.483052285 |
| IARS | 10.32793461 |
| IARS2 | 11.25445798 |
| IBSP | 5.507527498 |
| IBTK | 11.48461678 |
| ICA1 | 7.673710432 |
| ICAM1 | 7.992746626 |
| ICAM2 | 10.09141367 |
| ICAM3 | 11.82159561 |
| ICAM4 | 6.051620775 |
| ICF45 | 6.339562062 |
| ICK | 8.43398117 |
| ICMT | 9.036836434 |
| ICOSLG | 5.634859053 |
| ICT1 | 11.25849501 |
| ID1 | 9.212825622 |
| ID2 | 12.38793648 |
| ID2B | 7.099824333 |
| ID3 | 10.03613281 |
| IDE | 8.818645124 |
| IDH1 | 10.09487255 |
| IDH2 | 12.75095804 |
| IDH3A | 8.796016919 |
| IDH3B | 11.45727435 |
| IDH3G | 9.183113213 |
| IDI1 | 10.16516375 |
| IDS | 8.065344765 |
| IDUA | 5.72812124 |
| IER2 | 7.655561853 |
| IER3 | 11.82131211 |
| IER3IP1 | 9.625162956 |
| IER5 | 7.138903025 |
| IER5L | 7.426044043 |
| IFI16 | 8.648690434 |
| IFI27 | 9.785380926 |
| IFI30 | 12.41515472 |
| IFI35 | 10.49847879 |
| IFI44 | 9.561375295 |
| IFI44L | 7.32606607 |
| IFIH1 | 9.515014745 |
| IFIT1 | 8.763695149 |
| IFIT2 | 8.347040164 |
| IFIT3 | 6.83251889 |
| IFIT5 | 7.506940582 |
| IFITM1 | 11.6524517 |
| IFITM2 | 14.6605096 |
| IFITM3 | 14.6947357 |
| IFITM4P | 7.804407372 |
| IFNAR1 | 10.00636762 |
| IFNAR2 | 9.399229359 |
| IFNG | 5.841239551 |
| IFNGR1 | 12.50011829 |
| IFNGR2 | 11.832287 |
| IFP38 | 11.15304626 |
| IFRD1 | 8.443870178 |
| IFRD2 | 8.91008029 |
| IFT122 | 7.849052879 |
| IFT20 | 10.57881073 |
| IFT52 | 6.993290889 |
| IFT57 | 6.395986085 |
| IFT74 | 9.536723822 |
| IFT80 | 5.995185961 |
| IGBP1 | 8.801037532 |
| IGF1 | 7.596147052 |
| IGF1R | 6.349811601 |
| IGF2 | 9.37959359 |
| IGF2BP1 | 5.78914804 |
| IGF2BP2 | 7.268502529 |
| IGF2R | 11.65796194 |
| IGFALS | 9.823046729 |
| IGFBP1 | 11.99564188 |
| IGFBP2 | 11.44631176 |
| IGFBP3 | 12.2767214 |
| IGFBP4 | 12.2374721 |
| IGFBP5 | 9.555343217 |
| IGFBP6 | 8.09371462 |
| IGFBP7 | 13.50288529 |
| IGFL3 | 5.628134382 |
| IGHMBP2 | 6.702206983 |
| IGJ | 10.09270513 |
| IGLL1 | 5.825149676 |
| IGLL3 | 5.752427595 |
| IGSF11 | 5.674790093 |
| IGSF2 | 8.409392833 |
| IGSF21 | 5.529540667 |
| IGSF3 | 6.122851523 |
| IGSF6 | 11.70073682 |
| IGSF8 | 5.439663476 |
| IGSF9 | 9.489703873 |
| IHH | 5.463072633 |
| IHPK1 | 7.305684514 |
| IHPK2 | 6.631542254 |
| IHPK3 | 8.032126954 |
| IIP45 | 5.862671993 |
| IK | 9.714986177 |
| IKBKAP | 6.06858106 |
| IKBKB | 8.063518819 |
| IKBKE | 5.77260555 |
| IKBKG | 11.35382907 |
| IKIP | 7.977362326 |
| IKZF3 | 8.06068196 |
| IL10RA | 7.566708225 |
| IL10RB | 9.721757703 |
| IL11RA | 8.339162105 |
| IL12RB1 | 6.255043902 |
| IL13RA1 | 12.10641937 |
| IL13RA2 | 7.980616868 |
| IL15 | 7.116724538 |
| IL15RA | 6.434267831 |
| IL16 | 5.460578846 |
| IL17F | 5.804024179 |
| IL17R | 6.576210888 |
| IL17RB | 9.615279296 |
| IL17RC | 7.001542983 |
| IL17RE | 5.80652746 |
| IL18 | 13.1264859 |
| IL18R1 | 8.697651963 |
| IL18RAP | 8.147109709 |
| IL1B | 8.771854421 |
| IL1F5 | 6.10935911 |
| IL1R1 | 8.701618284 |
| IL1R2 | 9.156263656 |
| IL1RAP | 8.696471682 |
| IL1RAPL1 | 6.882295809 |
| IL1RN | 8.166051686 |
| IL20 | 6.098143406 |
| IL20RA | 6.191985486 |
| IL20RB | 5.931301907 |
| IL21R | 7.192737175 |
| IL22 | 5.507216868 |
| IL22RA1 | 5.603767498 |
| IL25 | 5.802442362 |
| IL26 | 6.832578536 |
| IL27 | 6.550969438 |
| IL27RA | 7.365288105 |
| IL28RA | 6.383213061 |
| IL2RA | 5.770581128 |
| IL2RB | 7.721576086 |
| IL31RA | 5.674560456 |
| IL32 | 10.98775159 |
| IL33 | 10.1322238 |
| IL3RA | 6.705354151 |
| IL4R | 12.229894 |
| IL6 | 7.18306547 |
| IL6R | 5.970543687 |
| IL6ST | 7.047745885 |
| IL7 | 7.836975546 |
| IL7R | 6.863248019 |
| IL8 | 9.404898838 |
| IL8RA | 5.860791707 |
| IL8RB | 7.26196302 |
| IL8RBP | 7.698660798 |
| ILF2 | 11.94176581 |
| ILF3 | 7.179748508 |
| ILK | 9.752170617 |
| ILKAP | 8.46845799 |
| ILVBL | 10.33265156 |
| IMMP1L | 8.716886398 |
| IMMP2L | 9.515938069 |
| IMMT | 11.05127157 |
| IMP3 | 12.52298405 |
| IMP4 | 11.32975533 |
| IMP5 | 5.700507855 |
| IMPA1 | 10.11293498 |
| IMPA2 | 11.35702827 |
| IMPACT | 5.723949119 |
| IMPAD1 | 9.353077562 |
| IMPDH1 | 6.786543573 |
| IMPDH2 | 11.35211307 |
| IMPG2 | 5.776234562 |
| INADL | 5.986192896 |
| INCA1 | 5.635657119 |
| INDO | 6.029649589 |
| INDOL1 | 6.585439875 |
| ING1 | 8.704014169 |
| ING2 | 8.003952408 |
| ING3 | 6.379992193 |
| ING4 | 6.607000764 |
| ING5 | 5.595741187 |
| INGX | 5.684996305 |
| INHBA | 6.04171772 |
| INHBB | 7.892849531 |
| INHBC | 7.481655322 |
| INHBE | 13.03617863 |
| INMT | 10.40894964 |
| INO80 | 6.368703842 |
| INO80E | 7.857419033 |
| INOC1 | 7.825299445 |
| INPP1 | 10.6608373 |
| INPP4A | 5.656397643 |
| INPP4B | 8.466739595 |
| INPP5A | 8.440628774 |
| INPP5B | 6.672430291 |
| INPP5D | 6.964161827 |
| INPP5E | 5.907794299 |
| INPP5F | 7.061641993 |
| INPPL1 | 8.943374698 |
| INS | 6.064867471 |
| INSIG1 | 11.18659495 |
| INSIG2 | 9.917157762 |
| INSL3 | 5.628448214 |
| INSL4 | 5.792243141 |
| INSM1 | 6.625578715 |
| INSR | 7.138630096 |
| INT1 | 7.443815483 |
| INTS12 | 10.84920377 |
| INTS2 | 7.610995087 |
| INTS3 | 9.028641501 |
| INTS4 | 8.235011952 |
| INTS5 | 8.206173582 |
| INTS7 | 8.190558981 |
| INTS8 | 8.651990198 |
| INTS9 | 8.668149309 |
| IPF1 | 6.109519443 |
| IPMK | 5.804946059 |
| IPO11 | 9.653152831 |
| IPO13 | 7.165426317 |
| IPO4 | 8.976325153 |
| IPO5 | 7.630224118 |
| IPO7 | 8.357602551 |
| IPO8 | 8.156467584 |
| IPO9 | 7.281305761 |
| IPP | 7.786900351 |
| IPPK | 5.753177282 |
| IQCB1 | 8.704357492 |
| IQCC | 5.958860054 |
| IQCG | 6.302291083 |
| IQCH | 6.37424421 |
| IQCK | 7.142367489 |
| IQGAP1 | 8.188466834 |
| IQGAP2 | 11.04829904 |
| IQGAP3 | 6.221103364 |
| IQSEC1 | 9.074978053 |
| IQSEC2 | 5.577284473 |
| IQSEC3 | 5.7319964 |
| IQWD1 | 9.806047914 |
| IRAK1 | 10.65663062 |
| IRAK1BP1 | 6.441261576 |
| IRAK2 | 9.915997267 |
| IRAK3 | 7.501632312 |
| IRAK4 | 7.083509019 |
| IREB2 | 6.206989141 |
| IRF1 | 10.83897164 |
| IRF2 | 5.974353891 |
| IRF2BP1 | 7.463965114 |
| IRF2BP2 | 8.407583394 |
| IRF3 | 7.985195649 |
| IRF4 | 6.113457374 |
| IRF6 | 6.967042194 |
| IRF7 | 10.46844122 |
| IRF8 | 10.01582706 |
| IRS1 | 8.128341731 |
| IRS2 | 8.720635505 |
| IRS4 | 5.575928093 |
| IRX1 | 9.242743296 |
| IRX2 | 5.893459537 |
| IRX3 | 8.992342774 |
| IRX6 | 5.489956548 |
| ISCA1 | 8.132614028 |
| ISCA2 | 11.29044388 |
| ISCU | 9.839414955 |
| ISG15 | 9.251069785 |
| ISG20 | 10.98907587 |
| ISG20L1 | 10.25613912 |
| ISG20L2 | 8.654822751 |
| ISGF3G | 10.52698434 |
| ISL2 | 5.694671897 |
| ISLR | 5.653695038 |
| ISOC1 | 11.77798489 |
| ISOC2 | 11.20983811 |
| ISX | 5.612620312 |
| ISY1 | 8.57459546 |
| ISYNA1 | 6.257353036 |
| ITCH | 10.0742461 |
| ITFG2 | 7.900566244 |
| ITFG3 | 8.378498949 |
| ITGA1 | 9.803644769 |
| ITGA2 | 5.758502776 |
| ITGA2B | 6.420700658 |
| ITGA5 | 8.731449904 |
| ITGA7 | 5.976122924 |
| ITGA9 | 8.555840415 |
| ITGAD | 6.404147307 |
| ITGAE | 9.83033146 |
| ITGAL | 6.372928268 |
| ITGAM | 6.956402544 |
| ITGAV | 9.432747832 |
| ITGAX | 6.313807411 |
| ITGB1 | 9.083738894 |
| ITGB1BP1 | 8.357979416 |
| ITGB1BP2 | 5.895168602 |
| ITGB2 | 11.86725549 |
| ITGB3 | 7.857614074 |
| ITGB3BP | 7.469551392 |
| ITGB4 | 5.94475945 |
| ITGB5 | 10.63536267 |
| ITGBL1 | 5.675915218 |
| ITIH1 | 13.54486395 |
| ITIH2 | 12.78901785 |
| ITIH3 | 11.17030762 |
| ITIH4 | 9.464519342 |
| ITK | 5.813803558 |
| ITLN1 | 6.194913541 |
| ITM2A | 7.33283985 |
| ITM2B | 13.68229102 |
| ITM2C | 9.110775627 |
| ITPA | 9.605866384 |
| ITPK1 | 8.527013625 |
| ITPKA | 6.982297541 |
| ITPKB | 6.416847126 |
| ITPKC | 6.649004155 |
| ITPR1 | 8.846920753 |
| ITPR2 | 8.526745427 |
| ITPR3 | 6.779497775 |
| ITPRIP | 8.676193345 |
| ITR | 9.737034659 |
| ITSN1 | 9.297337259 |
| IVD | 9.371401922 |
| IVL | 6.111835898 |
| IVNS1ABP | 8.967480465 |
| IWS1 | 9.424744396 |
| IYD | 7.150100217 |
| JAG1 | 7.136730108 |
| JAGN1 | 9.160038998 |
| JAK1 | 8.270416435 |
| JAK2 | 6.396354784 |
| JAKMIP2 | 6.215606351 |
| JAM2 | 7.508796973 |
| JAM3 | 8.169148154 |
| JARID1A | 8.766883294 |
| JARID1B | 8.225421832 |
| JARID1D | 6.904723442 |
| JARID2 | 6.917165709 |
| JAZF1 | 8.599579395 |
| JDP2 | 7.955859717 |
| JMJD1A | 6.459108101 |
| JMJD1B | 8.029156572 |
| JMJD1C | 9.427643792 |
| JMJD2B | 6.533535987 |
| JMJD4 | 7.337718881 |
| JMJD5 | 8.400118126 |
| JMY | 5.917244379 |
| JOSD1 | 9.935101034 |
| JOSD2 | 6.703593326 |
| JOSD3 | 7.853714353 |
| JPH1 | 5.773175027 |
| JPH2 | 7.117278421 |
| JRKL | 6.169122625 |
| JTB | 8.091928905 |
| JTV1 | 11.92368295 |
| JUB | 7.045353364 |
| JUN | 6.9614295 |
| JUND | 13.13596198 |
| JUP | 7.726443185 |
| KA21 | 9.009905031 |
| KALRN | 8.27884625 |
| KANK1 | 10.28186357 |
| KANK2 | 9.181309508 |
| KARS | 9.465215475 |
| KAT5 | 6.37628288 |
| KATNA1 | 6.910568061 |
| KATNAL1 | 6.363699332 |
| KATNAL2 | 5.526340026 |
| KATNB1 | 7.479014027 |
| KBTBD11 | 9.184472648 |
| KBTBD2 | 10.36980299 |
| KBTBD3 | 6.63790686 |
| KBTBD4 | 8.090423979 |
| KBTBD5 | 5.605523794 |
| KBTBD6 | 6.41450195 |
| KBTBD7 | 7.89866035 |
| KBTBD8 | 7.496262553 |
| KBTBD9 | 6.583300118 |
| KCMF1 | 6.011705276 |
| KCNA10 | 5.89496863 |
| KCNA6 | 6.096615732 |
| KCNAB1 | 6.262972708 |
| KCNB1 | 6.945389283 |
| KCNE1L | 7.555452954 |
| KCNG1 | 6.473334868 |
| KCNH6 | 6.643862102 |
| KCNH7 | 6.485037984 |
| KCNJ10 | 9.284970934 |
| KCNJ14 | 6.151806445 |
| KCNJ15 | 5.746298878 |
| KCNJ16 | 7.230683598 |
| KCNJ2 | 6.684884726 |
| KCNJ4 | 6.419596102 |
| KCNJ8 | 10.24521877 |
| KCNK1 | 9.366485715 |
| KCNK10 | 5.619582118 |
| KCNK13 | 6.374094697 |
| KCNK17 | 6.107888194 |
| KCNK3 | 8.041033639 |
| KCNK4 | 5.800764413 |
| KCNK5 | 6.949375941 |
| KCNK6 | 7.286891202 |
| KCNK9 | 6.097080301 |
| KCNMA1 | 5.761005439 |
| KCNMB1 | 5.83956132 |
| KCNMB3 | 5.786869877 |
| KCNMB4 | 5.925952724 |
| KCNN2 | 7.685345254 |
| KCNQ1 | 5.738942718 |
| KCNRG | 5.617372624 |
| KCNS3 | 6.867766328 |
| KCNV2 | 7.789221771 |
| KCR1 | 5.824598706 |
| KCTD10 | 8.216475228 |
| KCTD11 | 6.081189717 |
| KCTD12 | 8.444236224 |
| KCTD13 | 6.185237171 |
| KCTD14 | 9.022341395 |
| KCTD15 | 7.068887615 |
| KCTD17 | 8.581539474 |
| KCTD18 | 8.130495559 |
| KCTD2 | 7.098544085 |
| KCTD20 | 10.64822851 |
| KCTD21 | 7.489308964 |
| KCTD3 | 9.993856477 |
| KCTD5 | 10.73121623 |
| KCTD6 | 7.630817099 |
| KCTD8 | 5.712341173 |
| KCTD9 | 8.352206662 |
| KDELC1 | 7.302805562 |
| KDELC2 | 9.933299835 |
| KDELR1 | 6.901564539 |
| KDELR2 | 12.65763262 |
| KDELR3 | 8.451681872 |
| KDR | 5.815200649 |
| KEAP1 | 7.988528733 |
| KENAE | 7.497238194 |
| KHDRBS1 | 9.009839706 |
| KHDRBS3 | 9.880362667 |
| KHK | 9.494512414 |
| KHSRP | 9.636150522 |
| KIAA0020 | 9.450636809 |
| KIAA0040 | 6.068489634 |
| KIAA0090 | 8.546878487 |
| KIAA0100 | 10.30694765 |
| KIAA0101 | 6.93296794 |
| KIAA0133 | 6.374196938 |
| KIAA0141 | 8.304047938 |
| KIAA0143 | 11.506369 |
| KIAA0152 | 11.75973565 |
| KIAA0157 | 10.00562786 |
| KIAA0174 | 10.82624603 |
| KIAA0179 | 6.820825747 |
| KIAA0182 | 9.02117723 |
| KIAA0194 | 8.998739302 |
| KIAA0195 | 9.461846525 |
| KIAA0196 | 9.678865114 |
| KIAA0232 | 6.713359167 |
| KIAA0240 | 8.345979645 |
| KIAA0241 | 7.180099553 |
| KIAA0247 | 7.913488927 |
| KIAA0256 | 8.714682581 |
| KIAA0258 | 6.46390686 |
| KIAA0261 | 7.871859115 |
| KIAA0319L | 6.990114793 |
| KIAA0323 | 7.589667485 |
| KIAA0329 | 9.088963988 |
| KIAA0355 | 9.503639638 |
| KIAA0367 | 6.802447132 |
| KIAA0391 | 9.579191179 |
| KIAA0406 | 6.677911876 |
| KIAA0408 | 6.596391779 |
| KIAA0409 | 10.22869512 |
| KIAA0423 | 7.669141611 |
| KIAA0427 | 6.906203291 |
| KIAA0460 | 6.224037116 |
| KIAA0494 | 9.072048624 |
| KIAA0495 | 7.452616547 |
| KIAA0513 | 7.333431427 |
| KIAA0527 | 7.267330121 |
| KIAA0528 | 9.610224906 |
| KIAA0556 | 7.590812092 |
| KIAA0564 | 7.453521735 |
| KIAA0565 | 5.783427415 |
| KIAA0586 | 7.431467795 |
| KIAA0644 | 5.686676091 |
| KIAA0649 | 6.146701564 |
| KIAA0652 | 7.17270349 |
| KIAA0664 | 9.029551203 |
| KIAA0672 | 6.507866174 |
| KIAA0674 | 7.548206393 |
| KIAA0692 | 5.732315436 |
| KIAA0746 | 8.772240407 |
| KIAA0753 | 6.393058734 |
| KIAA0773 | 6.277295596 |
| KIAA0776 | 8.605790332 |
| KIAA0804 | 6.88795789 |
| KIAA0830 | 6.560498711 |
| KIAA0831 | 8.637288238 |
| KIAA0853 | 5.887568917 |
| KIAA0859 | 8.409447847 |
| KIAA0888 | 5.373905722 |
| KIAA0889 | 5.90462593 |
| KIAA0892 | 6.611896289 |
| KIAA0895 | 8.217320047 |
| KIAA0907 | 7.93007369 |
| KIAA0913 | 9.659972469 |
| KIAA0922 | 6.695771141 |
| KIAA0947 | 6.79429016 |
| KIAA0999 | 9.485361883 |
| KIAA1012 | 10.08815134 |
| KIAA1026 | 5.736722687 |
| KIAA1033 | 7.97992674 |
| KIAA1076 | 6.045947014 |
| KIAA1128 | 7.614702887 |
| KIAA1143 | 7.598176014 |
| KIAA1155 | 7.616758201 |
| KIAA1160 | 9.750478472 |
| KIAA1161 | 7.745404815 |
| KIAA1166 | 5.786530253 |
| KIAA1191 | 10.16901071 |
| KIAA1199 | 5.916398167 |
| KIAA1205 | 5.689809453 |
| KIAA1211 | 6.279802271 |
| KIAA1217 | 7.186403297 |
| KIAA1219 | 6.783173081 |
| KIAA1239 | 7.764729135 |
| KIAA1244 | 5.899582802 |
| KIAA1267 | 7.185449492 |
| KIAA1271 | 5.783299308 |
| KIAA1274 | 7.468464299 |
| KIAA1276 | 5.728333733 |
| KIAA1279 | 8.774641575 |
| KIAA1285 | 7.153571298 |
| KIAA1324L | 5.57982099 |
| KIAA1328 | 5.996475473 |
| KIAA1333 | 5.865837775 |
| KIAA1370 | 8.537358324 |
| KIAA1383 | 6.266622802 |
| KIAA1407 | 5.572316232 |
| KIAA1423 | 10.73297523 |
| KIAA1429 | 7.081703371 |
| KIAA1434 | 8.190554238 |
| KIAA1467 | 6.249392015 |
| KIAA1468 | 7.63725935 |
| KIAA1505 | 6.16148615 |
| KIAA1522 | 7.156749583 |
| KIAA1524 | 5.482255442 |
| KIAA1529 | 5.608258248 |
| KIAA1530 | 6.561489616 |
| KIAA1539 | 7.243893797 |
| KIAA1542 | 10.20869387 |
| KIAA1543 | 6.330035614 |
| KIAA1545 | 5.656202366 |
| KIAA1553 | 6.305168143 |
| KIAA1571 | 7.131806239 |
| KIAA1586 | 5.659530798 |
| KIAA1598 | 10.53781493 |
| KIAA1600 | 10.04257391 |
| KIAA1602 | 6.725677531 |
| KIAA1604 | 6.340215435 |
| KIAA1618 | 7.44085024 |
| KIAA1622 | 5.965592497 |
| KIAA1627 | 6.146272861 |
| KIAA1641 | 5.966588991 |
| KIAA1644 | 5.834197167 |
| KIAA1671 | 10.85552644 |
| KIAA1679 | 5.897964387 |
| KIAA1704 | 7.093931015 |
| KIAA1715 | 5.770097666 |
| KIAA1737 | 8.915355413 |
| KIAA1794 | 6.083840899 |
| KIAA1797 | 8.471994227 |
| KIAA1826 | 8.651141458 |
| KIAA1840 | 9.75241664 |
| KIAA1843 | 5.749863317 |
| KIAA1862 | 6.796139868 |
| KIAA1920 | 6.878492688 |
| KIAA1935 | 6.612069014 |
| KIAA1949 | 8.764232464 |
| KIAA1957 | 7.374690333 |
| KIAA1958 | 7.038202527 |
| KIAA1961 | 8.573871373 |
| KIAA1967 | 8.339029605 |
| KIAA1984 | 5.944811888 |
| KIAA2002 | 6.077063408 |
| KIAA2010 | 6.222471181 |
| KIAA2013 | 9.610744199 |
| KIAA2018 | 5.671863899 |
| KIAA2026 | 5.803643905 |
| KIDINS220 | 8.955138192 |
| KIF11 | 5.662484246 |
| KIF13A | 6.075678158 |
| KIF13B | 8.162727851 |
| KIF14 | 6.139661132 |
| KIF16B | 5.996953247 |
| KIF1B | 8.154258537 |
| KIF1C | 8.135621182 |
| KIF20A | 5.593661477 |
| KIF20B | 7.052757501 |
| KIF21A | 10.20171197 |
| KIF22 | 8.782715883 |
| KIF23 | 5.899458941 |
| KIF2A | 6.998014511 |
| KIF2C | 5.817811347 |
| KIF3B | 8.320434548 |
| KIF4A | 5.982036294 |
| KIF5B | 8.110517447 |
| KIF5C | 5.782043088 |
| KIFAP3 | 9.118563093 |
| KIFC1 | 5.925184076 |
| KIFC2 | 6.038144206 |
| KIFC3 | 6.027541581 |
| KIR2DL3 | 5.558216236 |
| KIR2DL5A | 5.886078415 |
| KIR2DS1 | 6.006505965 |
| KIR2DS3 | 5.912089814 |
| KIR2DS5 | 5.757229134 |
| KIR3DP1 | 5.623865034 |
| KIRREL2 | 5.829959656 |
| KIT | 5.397829941 |
| KITLG | 5.468779157 |
| KL | 5.628731282 |
| KLB | 8.400190223 |
| KLC2 | 6.066395675 |
| KLC3 | 5.88600868 |
| KLF1 | 6.165018846 |
| KLF10 | 6.94543263 |
| KLF11 | 9.473848796 |
| KLF12 | 7.664164981 |
| KLF13 | 9.412913337 |
| KLF15 | 8.861425341 |
| KLF16 | 5.817160707 |
| KLF2 | 11.64338509 |
| KLF3 | 5.934140704 |
| KLF4 | 7.338172014 |
| KLF6 | 9.445886942 |
| KLF7 | 5.987739984 |
| KLF9 | 12.20224706 |
| KLHDC1 | 5.8447393 |
| KLHDC10 | 6.010788822 |
| KLHDC2 | 11.8441508 |
| KLHDC3 | 11.75390299 |
| KLHDC4 | 8.684738575 |
| KLHDC5 | 9.176076729 |
| KLHDC8B | 5.662670859 |
| KLHDC9 | 6.797398395 |
| KLHL11 | 5.674514758 |
| KLHL12 | 7.735627852 |
| KLHL15 | 6.451458636 |
| KLHL18 | 6.043907713 |
| KLHL2 | 9.877601724 |
| KLHL20 | 7.599626172 |
| KLHL21 | 8.032973978 |
| KLHL22 | 9.267955885 |
| KLHL23 | 7.659261286 |
| KLHL24 | 6.717510877 |
| KLHL25 | 7.894138932 |
| KLHL3 | 6.49022447 |
| KLHL36 | 9.384250583 |
| KLHL5 | 11.9690891 |
| KLHL7 | 7.484734261 |
| KLHL8 | 8.64695778 |
| KLHL9 | 9.60062593 |
| KLK10 | 6.559098616 |
| KLK14 | 5.744193313 |
| KLK3 | 5.694501217 |
| KLK5 | 5.717396355 |
| KLK7 | 5.840278676 |
| KLK8 | 5.602798626 |
| KLKB1 | 11.56230029 |
| KLRB1 | 7.957484244 |
| KLRC1 | 5.69960976 |
| KLRD1 | 6.643571623 |
| KLRF1 | 5.83943205 |
| KLRG1 | 7.137341275 |
| KMO | 9.595951123 |
| KNDC1 | 5.807373469 |
| KNG1 | 14.82577306 |
| KNS2 | 7.525122259 |
| KNTC1 | 6.762315329 |
| KPNA1 | 9.504747862 |
| KPNA2 | 8.147690591 |
| KPNA3 | 10.04384844 |
| KPNA4 | 10.36535655 |
| KPNA5 | 5.484977532 |
| KPNA6 | 10.22996669 |
| KPNB1 | 11.77175039 |
| KPTN | 6.30041202 |
| KRAS | 8.560336171 |
| KRCC1 | 10.66448012 |
| KREMEN1 | 5.836736886 |
| KREMEN2 | 8.178389688 |
| KRIT1 | 7.588188272 |
| KRT10 | 11.25123483 |
| KRT18 | 8.940803094 |
| KRT19 | 5.925560465 |
| KRT3 | 6.308859047 |
| KRT31 | 7.044300949 |
| KRT6C | 5.75277956 |
| KRT7 | 6.042711469 |
| KRT75 | 6.455802804 |
| KRT79 | 5.726462025 |
| KRT8 | 11.38408583 |
| KRTAP10-2 | 5.8140895 |
| KRTAP10-6 | 5.622512411 |
| KRTAP13-4 | 5.776180487 |
| KRTAP19-7 | 5.820429433 |
| KRTAP5-1 | 7.636053753 |
| KRTCAP2 | 12.99092628 |
| KRTCAP3 | 7.717608546 |
| KRTHA6 | 5.945500554 |
| KRTHB6 | 8.646772722 |
| KSR2 | 5.841773633 |
| KTELC1 | 9.443142292 |
| KTI12 | 9.017531258 |
| KTN1 | 8.394886888 |
| KYNU | 11.91263844 |
| L2HGDH | 8.856040093 |
| L3MBTL2 | 8.367992369 |
| L3MBTL3 | 7.065106119 |
| LACE1 | 7.268411432 |
| LACRT | 5.947972308 |
| LACTB | 9.225583728 |
| LACTB2 | 12.33812454 |
| LAD1 | 8.449783181 |
| LAG3 | 5.776959994 |
| LAIR1 | 6.47832713 |
| LAIR2 | 6.476489952 |
| LAMA1 | 5.824291573 |
| LAMA2 | 6.143081837 |
| LAMA3 | 6.695301896 |
| LAMA4 | 6.652183636 |
| LAMA5 | 9.695052904 |
| LAMB1 | 7.679187917 |
| LAMB2 | 9.007946434 |
| LAMB3 | 5.996488368 |
| LAMB4 | 5.758305334 |
| LAMC1 | 9.694481228 |
| LAMC3 | 7.659119089 |
| LAMP1 | 14.04662992 |
| LAMP2 | 11.88202902 |
| LAMP3 | 6.136862213 |
| LANCL1 | 9.640947426 |
| LANCL2 | 8.055493435 |
| LAP3 | 12.42959109 |
| LAPTM4A | 10.99120703 |
| LAPTM4B | 11.19331918 |
| LAPTM5 | 9.739706726 |
| LARGE | 7.33245267 |
| LARP1 | 11.49427019 |
| LARP2 | 8.718535819 |
| LARP4 | 6.852333956 |
| LARP5 | 9.050826992 |
| LARP6 | 8.025310755 |
| LARS | 9.342473431 |
| LARS2 | 7.809580413 |
| LAS1L | 9.022883126 |
| LASP1 | 13.17866764 |
| LASS1 | 5.971841806 |
| LASS2 | 11.86762433 |
| LASS4 | 7.721027826 |
| LASS5 | 7.987430683 |
| LASS6 | 10.55310913 |
| LAT | 5.78583493 |
| LAT1-3TM | 7.778993031 |
| LAT2 | 6.691254968 |
| LATS2 | 5.913047462 |
| LAX1 | 5.66586289 |
| LBA1 | 5.943955144 |
| LBH | 6.093592472 |
| LBP | 14.35738873 |
| LBR | 7.67908867 |
| LBX2 | 5.657972358 |
| LCAT | 10.73333087 |
| LCE1C | 5.982525363 |
| LCE3A | 5.600482846 |
| LCE3B | 5.614973605 |
| LCK | 5.849972745 |
| LCMT1 | 8.06605306 |
| LCMT2 | 8.748053111 |
| LCN2 | 9.841787636 |
| LCORL | 5.761796396 |
| LCP1 | 11.97265993 |
| LCP2 | 9.411981849 |
| LDB1 | 5.965349635 |
| LDB2 | 11.07856773 |
| LDHA | 14.88306098 |
| LDHB | 9.206724417 |
| LDHC | 5.618201244 |
| LDHD | 7.612548906 |
| LDLR | 12.64321382 |
| LDLRAD3 | 7.209824609 |
| LDLRAP1 | 6.752980486 |
| LDOC1 | 5.753724045 |
| LDOC1L | 7.732056833 |
| LEAP-2 | 10.03524496 |
| LECT2 | 11.24316172 |
| LEMD2 | 8.475778116 |
| LEMD3 | 9.277536148 |
| LENG1 | 6.964131694 |
| LEO1 | 9.453298401 |
| LEPR | 7.239289327 |
| LEPRE1 | 8.387820355 |
| LEPREL1 | 9.232015315 |
| LEPREL2 | 7.875037217 |
| LEPROT | 11.93459164 |
| LEPROTL1 | 9.801503446 |
| LETM1 | 7.356774829 |
| LETMD1 | 7.911942018 |
| LFNG | 7.222650084 |
| LGALS1 | 12.06927931 |
| LGALS2 | 6.570115757 |
| LGALS3 | 8.559375774 |
| LGALS3BP | 8.525577064 |
| LGALS4 | 9.737405055 |
| LGALS8 | 5.590271962 |
| LGALS9 | 5.432228948 |
| LGI2 | 5.968118644 |
| LGMN | 11.90095006 |
| LGP2 | 7.143404904 |
| LGR4 | 11.02988677 |
| LGR6 | 6.131595434 |
| LGTN | 9.566488603 |
| LHB | 6.008094155 |
| LHCGR | 5.857412617 |
| LHFP | 8.031968687 |
| LHFPL1 | 6.409802567 |
| LHFPL2 | 7.701908199 |
| LHPP | 11.10503214 |
| LHX2 | 7.285005148 |
| LHX6 | 6.307902414 |
| LIAS | 8.133395771 |
| LIF | 5.738982048 |
| LIFR | 8.381041835 |
| LIG1 | 5.710397832 |
| LIG3 | 6.993020502 |
| LIG4 | 6.608875948 |
| LILRA2 | 8.693489534 |
| LILRA3 | 8.722710423 |
| LILRA4 | 6.523873328 |
| LILRA5 | 5.435694195 |
| LILRB1 | 6.986940396 |
| LILRB3 | 8.905270425 |
| LILRB4 | 5.881326024 |
| LILRB5 | 6.254237254 |
| LIMA1 | 8.931386776 |
| LIMCH1 | 8.656788893 |
| LIMD1 | 6.703457645 |
| LIME1 | 7.172266487 |
| LIMK1 | 8.138538058 |
| LIMK2 | 6.765284448 |
| LIMS1 | 9.395929487 |
| LIMS2 | 7.391815825 |
| LIN37 | 5.643618059 |
| LIN52 | 6.547358804 |
| LIN54 | 8.62409353 |
| LIN7A | 7.161116607 |
| LIN7B | 6.53324258 |
| LIN7C | 6.804439686 |
| LIN9 | 7.434053869 |
| LINGO4 | 5.796755419 |
| LINS1 | 6.045364687 |
| LIPA | 12.41069194 |
| LIPC | 10.91385759 |
| LIPG | 9.025101585 |
| LIPH | 5.837998338 |
| LIPT1 | 6.711692694 |
| LITAF | 12.55117354 |
| LIX1L | 8.997551728 |
| LKAP | 10.07405014 |
| LLGL1 | 7.125687904 |
| LLGL2 | 5.737805658 |
| LMAN1 | 9.237565051 |
| LMAN2 | 9.668053729 |
| LMAN2L | 8.755934626 |
| LMBR1 | 9.607606514 |
| LMBR1L | 7.805119318 |
| LMBRD1 | 11.78406222 |
| LMBRD2 | 7.045340708 |
| LMCD1 | 8.058653724 |
| LMF2 | 8.597244309 |
| LMNA | 10.54427856 |
| LMNB1 | 6.507671139 |
| LMNB2 | 8.07958865 |
| LMO2 | 9.340949657 |
| LMO4 | 10.3413047 |
| LMO7 | 6.467449188 |
| LMOD1 | 5.721924396 |
| LMOD3 | 10.23254531 |
| LMTK2 | 6.042376959 |
| LNPEP | 7.746155506 |
| LNX2 | 9.579850244 |
| LONP1 | 10.54726824 |
| LONP2 | 10.96124213 |
| LONRF1 | 7.667599352 |
| LONRF2 | 5.867379601 |
| LONRF3 | 8.150228375 |
| LOXHD1 | 6.186206465 |
| LOXL3 | 5.60430982 |
| LOXL4 | 6.231521904 |
| LPAL2 | 5.808345953 |
| LPAR1 | 7.268800229 |
| LPAR4 | 5.891671821 |
| LPAR5 | 6.703054745 |
| LPCAT1 | 7.48923485 |
| LPCAT3 | 11.88647561 |
| LPGAT1 | 9.916723894 |
| LPHN1 | 5.569724644 |
| LPHN2 | 10.48136143 |
| LPIN1 | 10.31174802 |
| LPIN2 | 9.931382291 |
| LPL | 5.540847734 |
| LPP | 11.80474453 |
| LPPR2 | 6.655831607 |
| LPXN | 8.893954322 |
| LRAP | 8.740582175 |
| LRAT | 6.03626427 |
| LRBA | 6.264464288 |
| LRCH3 | 6.050323585 |
| LRCH4 | 5.578440589 |
| LRDD | 7.259244467 |
| LRFN4 | 5.842846148 |
| LRG1 | 14.63229308 |
| LRIG1 | 9.654954661 |
| LRIG2 | 6.088411853 |
| LRIG3 | 7.503916335 |
| LRMP | 7.228335465 |
| LRP1 | 8.034546989 |
| LRP10 | 10.72690697 |
| LRP11 | 7.745620112 |
| LRP12 | 6.134790173 |
| LRP2 | 5.87289358 |
| LRP2BP | 5.951839354 |
| LRP5 | 8.405679078 |
| LRP5L | 6.406517311 |
| LRP6 | 6.097698573 |
| LRPAP1 | 10.54078689 |
| LRPPRC | 8.859699875 |
| LRRC1 | 6.010398771 |
| LRRC14 | 7.110963608 |
| LRRC15 | 6.396249338 |
| LRRC16 | 6.06753633 |
| LRRC17 | 6.711190931 |
| LRRC19 | 6.125313137 |
| LRRC2 | 7.443729478 |
| LRRC20 | 10.08715615 |
| LRRC23 | 6.85368413 |
| LRRC25 | 6.170097477 |
| LRRC28 | 9.835629742 |
| LRRC29 | 6.564183024 |
| LRRC3 | 7.144462562 |
| LRRC31 | 7.087866455 |
| LRRC32 | 7.835382329 |
| LRRC33 | 6.776555633 |
| LRRC37A | 5.561879761 |
| LRRC37B | 7.406663618 |
| LRRC40 | 7.215745861 |
| LRRC41 | 10.06660873 |
| LRRC42 | 10.25535854 |
| LRRC44 | 6.096881042 |
| LRRC45 | 9.035609558 |
| LRRC47 | 9.537396779 |
| LRRC4C | 5.940189437 |
| LRRC51 | 6.97097392 |
| LRRC57 | 7.23148294 |
| LRRC58 | 7.265785461 |
| LRRC59 | 9.370716318 |
| LRRC6 | 5.987061905 |
| LRRC61 | 6.63027237 |
| LRRC8A | 8.155375664 |
| LRRC8C | 7.399331023 |
| LRRC8D | 9.539581204 |
| LRRC8E | 6.232422357 |
| LRRFIP1 | 10.06707895 |
| LRRFIP2 | 9.196986532 |
| LRRK1 | 5.734462902 |
| LRRK2 | 5.765741819 |
| LRRN2 | 5.907105343 |
| LRRTM2 | 5.805459049 |
| LRSAM1 | 8.854687636 |
| LRTM1 | 6.163573684 |
| LRWD1 | 8.839551783 |
| LSAMP | 6.136742242 |
| LSG1 | 7.982274297 |
| LSM1 | 10.10063783 |
| LSM10 | 10.37041108 |
| LSM11 | 5.681978448 |
| LSM12 | 7.846420592 |
| LSM14A | 10.94933438 |
| LSM2 | 12.02102964 |
| LSM3 | 12.06528445 |
| LSM4 | 10.80033132 |
| LSM5 | 12.38148117 |
| LSM6 | 8.31278234 |
| LSM7 | 11.4435289 |
| LSM8 | 6.378797979 |
| LSMD1 | 13.04154013 |
| LSR | 8.279586623 |
| LSS | 8.286531338 |
| LST1 | 9.121802547 |
| LTA | 6.210344451 |
| LTA4H | 11.17593494 |
| LTB | 7.419664458 |
| LTB4R | 6.621034365 |
| LTBP2 | 6.07988453 |
| LTBP3 | 6.302763993 |
| LTBP4 | 6.168280584 |
| LTBR | 10.88247872 |
| LTC4S | 5.959552222 |
| LTF | 5.657482348 |
| LTV1 | 10.99768754 |
| LUC7L | 6.693796897 |
| LUC7L2 | 6.998063042 |
| LUM | 9.116154617 |
| LUZP1 | 6.261836579 |
| LXN | 8.337453234 |
| LY6E | 12.640762 |
| LY6G5C | 5.889955216 |
| LY86 | 8.386919953 |
| LY9 | 5.879375617 |
| LY96 | 11.01789324 |
| LYAR | 8.944828958 |
| LYCAT | 9.765711685 |
| LYL1 | 6.110506927 |
| LYN | 11.42780479 |
| LYPD1 | 5.876750323 |
| LYPD2 | 5.721293165 |
| LYPD3 | 5.709765718 |
| LYPD5 | 5.718121914 |
| LYPLA1 | 9.177487253 |
| LYPLA2 | 6.423504655 |
| LYPLA2P1 | 5.882466472 |
| LYPLAL1 | 10.67921884 |
| LYRM1 | 12.62739114 |
| LYRM4 | 8.126726181 |
| LYSMD1 | 5.702171171 |
| LYSMD2 | 11.32245329 |
| LYSMD3 | 8.07520818 |
| LYSMD4 | 6.012673389 |
| LYST | 6.566412821 |
| LYZ | 11.76949153 |
| LYZL1 | 5.577259428 |
| LZIC | 8.110892801 |
| LZTFL1 | 8.748680268 |
| LZTR1 | 8.916426909 |
| LZTR2 | 6.173362362 |
| LZTS1 | 5.581647903 |
| M160 | 6.211755843 |
| M6PR | 10.54322614 |
| M6PRBP1 | 12.00331107 |
| MAB21L1 | 5.952996349 |
| MAB21L2 | 6.51358506 |
| MACF1 | 8.105913886 |
| MACROD1 | 8.412872001 |
| MAD1L1 | 6.806571014 |
| MAD2L1 | 7.831013774 |
| MAD2L1BP | 8.81541109 |
| MAD2L2 | 10.72128028 |
| MADD | 7.130854785 |
| MAEA | 8.307117363 |
| MAEL | 5.9619937 |
| MAF | 9.767990073 |
| MAF1 | 6.128326038 |
| MAFB | 10.49908093 |
| MAFF | 7.064902192 |
| MAFG | 6.147619463 |
| MAFK | 5.666543176 |
| MAG | 6.919199128 |
| MAGEA3 | 5.785129082 |
| MAGEA5 | 5.730386938 |
| MAGEA6 | 5.870846115 |
| MAGEB1 | 5.860386351 |
| MAGED1 | 9.055065278 |
| MAGED2 | 7.128158873 |
| MAGEE1 | 5.759159856 |
| MAGEF1 | 7.560708045 |
| MAGEH1 | 9.023387477 |
| MAGEL2 | 5.939525821 |
| MAGI1 | 5.976137597 |
| Magmas | 10.48885863 |
| MAGOH | 11.76991084 |
| MAK10 | 7.434079084 |
| MAK16 | 9.238832489 |
| MAL | 5.769895145 |
| MAL2 | 10.26446036 |
| MALAT1 | 7.256388003 |
| MALL | 6.087396811 |
| MALT1 | 7.971528351 |
| MAMDC2 | 5.784996816 |
| MAMDC4 | 6.192763249 |
| MAML1 | 5.794559611 |
| MAML3 | 6.828025017 |
| MAN1A1 | 10.85894927 |
| MAN1A2 | 7.865952401 |
| MAN1B1 | 10.13887851 |
| MAN1C1 | 8.305589008 |
| MAN2A1 | 12.4421437 |
| MAN2A2 | 7.098568539 |
| MAN2B1 | 6.690193365 |
| MAN2B2 | 11.51966993 |
| MAN2C1 | 8.914720353 |
| MANBA | 8.11746838 |
| MANBAL | 10.13545271 |
| MANEA | 7.600007204 |
| MANEAL | 6.177167579 |
| MANSC1 | 7.274299187 |
| MAOA | 11.62215633 |
| MAOB | 10.14768758 |
| MAP1B | 6.907465943 |
| MAP1D | 6.411881824 |
| MAP1LC3A | 6.304828631 |
| MAP1LC3B | 10.18014724 |
| MAP1S | 6.966472025 |
| MAP2K1 | 10.96283771 |
| MAP2K1IP1 | 10.75454159 |
| MAP2K2 | 6.871023822 |
| MAP2K3 | 5.565498238 |
| MAP2K4 | 8.835874415 |
| MAP2K5 | 6.704574953 |
| MAP2K6 | 6.696614177 |
| MAP2K7 | 8.933811895 |
| MAP3K1 | 9.30215627 |
| MAP3K10 | 5.933246284 |
| MAP3K11 | 8.645606232 |
| MAP3K12 | 6.433130113 |
| MAP3K13 | 6.987438814 |
| MAP3K14 | 6.607547913 |
| MAP3K2 | 8.664874757 |
| MAP3K3 | 5.636960164 |
| MAP3K4 | 8.040278578 |
| MAP3K5 | 8.795649687 |
| MAP3K6 | 7.165863888 |
| MAP3K7 | 10.06650573 |
| MAP3K7IP1 | 6.642511346 |
| MAP3K7IP2 | 7.963721545 |
| MAP3K8 | 8.675370531 |
| MAP3K9 | 6.31069115 |
| MAP4 | 5.743696817 |
| MAP4K1 | 5.761783091 |
| MAP4K2 | 9.340432393 |
| MAP4K3 | 6.720113925 |
| MAP4K4 | 6.503153808 |
| MAP4K5 | 8.596496587 |
| MAP6 | 5.784868835 |
| MAP6D1 | 6.439747395 |
| MAP7 | 10.02266053 |
| MAPBPIP | 12.17249621 |
| MAPK1 | 7.656411821 |
| MAPK10 | 5.517745201 |
| MAPK11 | 5.743433296 |
| MAPK12 | 5.441502189 |
| MAPK13 | 8.167642928 |
| MAPK14 | 6.052553122 |
| MAPK3 | 11.21410927 |
| MAPK4 | 6.769569604 |
| MAPK6 | 11.40106944 |
| MAPK7 | 5.493210688 |
| MAPK8 | 6.65772121 |
| MAPK8IP1 | 5.588484552 |
| MAPK8IP3 | 5.615338343 |
| MAPK9 | 8.27621805 |
| MAPKAP1 | 7.932144093 |
| MAPKAPK2 | 9.106515879 |
| MAPKAPK3 | 10.88993217 |
| MAPKAPK5 | 8.122786846 |
| MAPRE1 | 10.59362216 |
| MAPRE2 | 10.04752993 |
| MAPRE3 | 7.376951917 |
| Mar-01 | 6.04105506 |
| Mar-02 | 8.482495119 |
| Mar-03 | 6.303507488 |
| Mar-05 | 6.684960812 |
| Mar-06 | 10.81036728 |
| Mar-07 | 10.92274717 |
| Mar-09 | 5.598779218 |
| MARCKS | 11.61008531 |
| MARCKSL1 | 9.579629588 |
| MARCO | 10.60864225 |
| MARK3 | 6.230528036 |
| MARLIN1 | 5.723006604 |
| MARS | 9.381287129 |
| MARS2 | 8.456868519 |
| MARVELD1 | 9.080178119 |
| MARVELD2 | 9.06690238 |
| MARVELD3 | 6.760903134 |
| MAS1L | 5.725164049 |
| MASK | 9.665590109 |
| MASP1 | 7.647667701 |
| MASP2 | 11.24650199 |
| MASS1 | 9.974929126 |
| MAST2 | 6.412341839 |
| MAST3 | 7.255864385 |
| MASTL | 5.677007244 |
| MAT1A | 11.78376179 |
| MAT2A | 12.29756849 |
| MAT2B | 8.575035306 |
| MATN2 | 8.071482143 |
| MATN3 | 5.773447965 |
| MATR3 | 9.030891482 |
| MAX | 6.555792909 |
| MAZ | 6.122391666 |
| MB | 5.776962822 |
| MBD2 | 6.996031672 |
| MBD3 | 7.730762044 |
| MBD4 | 11.72142373 |
| MBD6 | 8.354516694 |
| MBIP | 8.621073301 |
| MBL2 | 12.02119463 |
| MBNL1 | 10.38779564 |
| MBNL2 | 8.81888196 |
| MBNL3 | 8.176545241 |
| MBOAT7 | 7.856368381 |
| MBP | 8.76310085 |
| MBTPS1 | 9.046329984 |
| MBTPS2 | 6.574724846 |
| MC1R | 5.776070798 |
| MC2R | 5.481588802 |
| MCART1 | 12.17308385 |
| MCART6 | 5.873317249 |
| MCC | 6.401493874 |
| MCCC1 | 11.04285617 |
| MCCC2 | 6.507090826 |
| MCEE | 12.06203616 |
| MCEMP1 | 8.750829357 |
| MCFD2 | 11.01470998 |
| MCL1 | 9.429657091 |
| MCM10 | 5.93146227 |
| MCM2 | 7.105138561 |
| MCM3 | 8.177028682 |
| MCM3AP | 9.230534615 |
| MCM4 | 9.169313864 |
| MCM5 | 7.952509441 |
| MCM6 | 5.680768007 |
| MCM7 | 8.238449562 |
| MCM8 | 12.47410458 |
| MCOLN1 | 8.993765724 |
| MCOLN2 | 5.961716386 |
| MCPH1 | 6.145440198 |
| MCRS1 | 6.452353268 |
| MCTP1 | 5.871562647 |
| MCTP2 | 5.81522989 |
| MCTS1 | 10.97975228 |
| MDC1 | 8.476779199 |
| MDFI | 5.732840972 |
| MDFIC | 6.374580538 |
| MDGA1 | 5.624420427 |
| MDH1 | 13.47929208 |
| MDH2 | 13.41988004 |
| MDK | 7.21377619 |
| MDM1 | 6.616859954 |
| MDM2 | 6.383291618 |
| MDM4 | 5.560415476 |
| MDN1 | 5.534525518 |
| MDP-1 | 8.755918015 |
| MDS032 | 8.48105812 |
| MDS1 | 7.192539598 |
| ME1 | 8.933227462 |
| ME2 | 9.654204388 |
| ME3 | 5.75202095 |
| MEA1 | 10.01131627 |
| MECP2 | 6.13696532 |
| MECR | 5.487205066 |
| MED10 | 10.59323505 |
| MED11 | 7.688310748 |
| MED12 | 6.063331861 |
| MED13L | 6.544922351 |
| MED16 | 7.921770916 |
| MED18 | 6.459632687 |
| MED19 | 9.34844721 |
| MED20 | 10.32730139 |
| MED22 | 6.120421564 |
| MED23 | 7.282273326 |
| MED25 | 6.896058073 |
| MED26 | 7.714473808 |
| MED27 | 6.929711104 |
| MED28 | 10.15575413 |
| MED29 | 9.310860962 |
| MED30 | 10.49347908 |
| MED31 | 6.73782647 |
| MED4 | 9.776834281 |
| MED6 | 10.93679393 |
| MED8 | 8.291375927 |
| MED9 | 7.650942045 |
| MEF2A | 6.170112853 |
| MEF2B | 7.924128521 |
| MEF2C | 7.750975328 |
| MEF2D | 7.66693579 |
| MEFV | 5.665619285 |
| MEGF8 | 7.136809448 |
| MEIS1 | 6.042410892 |
| MEIS2 | 9.302365392 |
| MELK | 6.07805797 |
| MEMO1 | 10.08251104 |
| MEN1 | 5.683122401 |
| MEPCE | 10.11865683 |
| MERTK | 7.625894104 |
| MESDC1 | 7.081549076 |
| MESDC2 | 6.601262084 |
| MESP1 | 7.474720562 |
| MEST | 5.870105074 |
| MET | 9.397648865 |
| METAP1 | 10.05202794 |
| METAP2 | 12.72760276 |
| METRN | 6.31249049 |
| METRNL | 6.92315928 |
| METT10D | 6.35365828 |
| METT11D1 | 8.039519271 |
| METT5D1 | 8.233206215 |
| METTL1 | 7.60750316 |
| METTL10 | 5.769358692 |
| METTL11A | 5.863764724 |
| METTL2A | 7.701986644 |
| METTL2B | 6.058218915 |
| METTL3 | 8.261312158 |
| METTL4 | 6.145151567 |
| METTL5 | 11.36930634 |
| METTL6 | 6.612728324 |
| METTL7A | 11.09504639 |
| METTL7B | 12.19694893 |
| MFAP1 | 8.765497596 |
| MFAP3 | 7.718885949 |
| MFAP3L | 6.049454173 |
| MFAP4 | 8.540652328 |
| MFGE8 | 9.550282357 |
| MFHAS1 | 7.241386587 |
| MFI2 | 6.83613823 |
| MFN1 | 7.759274219 |
| MFN2 | 9.25274002 |
| MFNG | 9.536325206 |
| MFSD1 | 11.37835229 |
| MFSD11 | 9.71928679 |
| MFSD2 | 10.60382945 |
| MFSD3 | 10.94456463 |
| MFSD5 | 8.599394605 |
| MFSD8 | 8.668752355 |
| MFSD9 | 5.926584531 |
| MGA | 7.103257093 |
| MGAM | 5.644024622 |
| MGAT1 | 11.51677593 |
| MGAT2 | 9.955503878 |
| MGAT4A | 8.621539952 |
| MGAT4B | 7.685539716 |
| MGC11257 | 11.23842467 |
| MGC119295 | 5.499586101 |
| MGC12760 | 5.895268894 |
| MGC12965 | 5.853821933 |
| MGC12966 | 8.710883585 |
| MGC13057 | 9.111313822 |
| MGC13114 | 8.605101288 |
| MGC13168 | 6.707663647 |
| MGC13379 | 8.806102995 |
| MGC15523 | 7.946583883 |
| MGC15619 | 6.008965193 |
| MGC15634 | 5.878019292 |
| MGC15763 | 8.72151643 |
| MGC15875 | 9.663765067 |
| MGC15885 | 5.986419663 |
| MGC15912 | 6.118755728 |
| MGC16075 | 5.845833072 |
| MGC16121 | 5.816915985 |
| MGC16169 | 7.535230363 |
| MGC16385 | 10.2595254 |
| MGC16597 | 5.812958911 |
| MGC16824 | 5.75444546 |
| MGC18216 | 5.978778423 |
| MGC19604 | 8.304922386 |
| MGC20983 | 6.381167933 |
| MGC21881 | 7.731051739 |
| MGC23909 | 10.0368889 |
| MGC24039 | 8.560695099 |
| MGC2408 | 10.09691925 |
| MGC2654 | 8.559608565 |
| MGC26963 | 7.004626778 |
| MGC27348 | 5.804049543 |
| MGC2747 | 9.826676184 |
| MGC2752 | 6.33306699 |
| MGC3020 | 5.625207552 |
| MGC3123 | 7.200500613 |
| MGC3196 | 7.736055106 |
| MGC3207 | 8.580882025 |
| MGC33214 | 6.824263448 |
| MGC33407 | 5.73911836 |
| MGC33600 | 5.765695271 |
| MGC33657 | 6.044412257 |
| MGC33692 | 6.715588041 |
| MGC34800 | 5.70377526 |
| MGC35361 | 7.269460594 |
| MGC3731 | 10.05430092 |
| MGC39584 | 5.807744095 |
| MGC39900 | 5.851911294 |
| MGC40168 | 5.725941184 |
| MGC40170 | 6.732709888 |
| MGC40405 | 7.961761979 |
| MGC40489 | 6.215605264 |
| MGC40579 | 6.888952116 |
| MGC4172 | 8.634901386 |
| MGC41945 | 5.953524331 |
| MGC42367 | 5.789862914 |
| MGC42630 | 5.539231671 |
| MGC4399 | 5.53485188 |
| MGC45491 | 6.102951679 |
| MGC50722 | 6.034127439 |
| MGC5139 | 6.12203866 |
| MGC52000 | 8.320175488 |
| MGC5352 | 8.697953782 |
| MGC5457 | 5.790198169 |
| MGC57346 | 6.525796747 |
| MGC57359 | 10.24043259 |
| MGC61598 | 6.844586776 |
| MGC62100 | 5.605567745 |
| MGC70857 | 8.190123394 |
| MGC70863 | 5.951238693 |
| MGC70870 | 10.17894921 |
| MGC71993 | 11.59092928 |
| MGC72104 | 9.785234632 |
| MGC9712 | 6.120665454 |
| MGEA5 | 10.67855582 |
| MGLL | 8.68005798 |
| MGMT | 12.64152405 |
| MGP | 10.15049205 |
| MGRN1 | 7.288470791 |
| MGST1 | 11.76382644 |
| MGST2 | 13.67558313 |
| MGST3 | 13.29870608 |
| MIA | 5.879795443 |
| MIA2 | 6.019417627 |
| MIB1 | 6.078849478 |
| MIB2 | 6.394679479 |
| MICA | 8.613475183 |
| MICAL1 | 5.751403224 |
| MICALCL | 6.00018642 |
| MICALL1 | 6.899749312 |
| MICB | 6.511789898 |
| MID1 | 7.946618789 |
| MID1IP1 | 9.165616317 |
| MID2 | 6.399632414 |
| MIDN | 10.1330834 |
| MIER1 | 9.411244464 |
| MIER2 | 6.019934378 |
| MIF | 13.14146058 |
| MIF4GD | 9.010327436 |
| MINA | 7.240323186 |
| MINK1 | 5.517955216 |
| MINPP1 | 9.684896942 |
| MIPEP | 8.619177263 |
| MIS12 | 10.42225469 |
| MITD1 | 10.09721717 |
| MITF | 5.926018291 |
| MIZF | 7.981488851 |
| MKI67IP | 10.88496888 |
| MKKS | 10.28181841 |
| MKL1 | 6.076833204 |
| MKL2 | 7.038568969 |
| MKLN1 | 10.21513109 |
| MKNK1 | 8.380484777 |
| MKNK2 | 7.158863579 |
| MKRN1 | 10.77429534 |
| MKRN2 | 9.676091237 |
| MKS1 | 5.907567625 |
| MKX | 8.282529592 |
| MLC1 | 5.648079307 |
| MLC1SA | 10.16998785 |
| MLF1IP | 7.553448805 |
| MLF2 | 10.0075253 |
| MLH1 | 9.980201363 |
| MLKL | 7.22827839 |
| MLL3 | 5.964278447 |
| MLL4 | 6.059950488 |
| MLL5 | 6.197074567 |
| MLLT10 | 7.556765351 |
| MLLT11 | 7.080219367 |
| MLLT3 | 9.508096623 |
| MLLT4 | 6.08726539 |
| MLLT6 | 7.846848307 |
| MLLT7 | 8.637135674 |
| MLPH | 9.572135325 |
| MLR2 | 8.066766063 |
| MLX | 6.805806512 |
| MLYCD | 8.728653961 |
| MMAA | 9.718511933 |
| MMAB | 6.789522895 |
| MMACHC | 10.25050473 |
| MMD | 10.59072418 |
| MME | 6.401427761 |
| MMP1 | 5.985160792 |
| MMP12 | 5.793977303 |
| MMP15 | 6.965010217 |
| MMP16 | 5.808175947 |
| MMP21 | 5.873109508 |
| MMP23A | 5.559903675 |
| MMP23B | 5.736872996 |
| MMP25 | 7.089240321 |
| MMP7 | 7.658602455 |
| MMP9 | 7.752642564 |
| MMPL1 | 5.813252099 |
| MMRN1 | 8.459802756 |
| MMRN2 | 6.064883803 |
| MMS19L | 9.446380446 |
| MN1 | 5.864488425 |
| MNAT1 | 9.100657343 |
| MNDA | 9.310128625 |
| MNT | 7.850414735 |
| MOAP1 | 9.339743648 |
| MOBK1B | 5.52168879 |
| MOBKL1A | 6.46667838 |
| MOBKL2A | 6.719198752 |
| MOBKL2B | 5.653073909 |
| MOBKL2C | 9.534703973 |
| MOCOS | 11.49694265 |
| MOCS1 | 6.636822938 |
| MOCS2 | 8.242529189 |
| MOCS3 | 5.442136206 |
| MOG | 5.788865863 |
| MOGAT1 | 6.335982984 |
| MOGAT2 | 6.223619816 |
| MON1A | 6.008918623 |
| MON1B | 7.615828898 |
| MON2 | 8.777594668 |
| MORC2 | 9.265483195 |
| MORC3 | 6.017441101 |
| MORC4 | 10.97794389 |
| MORF4L1 | 9.077954942 |
| MORF4L2 | 11.93525414 |
| MORG1 | 7.042369334 |
| MORN2 | 8.446001608 |
| MORN5 | 6.045205904 |
| MOS | 8.890028704 |
| MOSC1 | 9.657454198 |
| MOSC2 | 11.85111098 |
| MOSPD1 | 6.974751771 |
| MOSPD2 | 8.0526896 |
| MOSPD3 | 7.665658529 |
| MOV10 | 7.329195071 |
| MOXD1 | 5.967614353 |
| MPDU1 | 11.98759656 |
| MPDZ | 8.133341048 |
| MPEG1 | 5.590150089 |
| MPFL | 5.833985037 |
| MPG | 6.028691586 |
| MPHOSPH10 | 10.09826542 |
| MPHOSPH6 | 9.53856713 |
| MPHOSPH8 | 8.377054566 |
| MPHOSPH9 | 7.134105139 |
| MPI | 6.833775186 |
| MPL | 6.171930457 |
| MPN2 | 6.117542915 |
| MPND | 11.23312944 |
| MPO | 6.87721572 |
| MPP1 | 9.595103957 |
| MPP5 | 8.822872878 |
| MPP6 | 8.346708429 |
| MPPE1 | 7.609893057 |
| MPPED1 | 5.914710341 |
| MPPED2 | 6.414357761 |
| MPRIP | 7.971466643 |
| MPST | 7.700873622 |
| MPV17 | 9.224635038 |
| MPV17L | 7.329615505 |
| MPZ | 5.848436411 |
| MPZL1 | 8.50418745 |
| MPZL2 | 8.481353839 |
| MPZL3 | 5.603282169 |
| MR1 | 9.338965142 |
| MRAP | 5.925700879 |
| MRAS | 5.452019331 |
| MRCL3 | 13.99668258 |
| MRE11A | 5.670499861 |
| MRFAP1 | 12.6229513 |
| MRFAP1L1 | 8.861601024 |
| MRGPRF | 6.130448035 |
| MRLC2 | 13.60122081 |
| MRM1 | 5.842252389 |
| MRO | 5.418056296 |
| MRP63 | 6.395648475 |
| MRPL1 | 12.27921742 |
| MRPL11 | 8.800742083 |
| MRPL12 | 10.03443706 |
| MRPL13 | 12.35513954 |
| MRPL14 | 10.63022964 |
| MRPL15 | 12.76133397 |
| MRPL16 | 10.89482721 |
| MRPL17 | 11.9794318 |
| MRPL18 | 11.66479494 |
| MRPL19 | 11.14671425 |
| MRPL2 | 10.69948671 |
| MRPL20 | 11.47212186 |
| MRPL21 | 9.083461997 |
| MRPL22 | 12.25602122 |
| MRPL23 | 11.97306382 |
| MRPL24 | 12.65883953 |
| MRPL27 | 11.50478818 |
| MRPL28 | 7.876666937 |
| MRPL3 | 12.45052566 |
| MRPL30 | 8.511227058 |
| MRPL32 | 12.70674698 |
| MRPL33 | 12.50027604 |
| MRPL34 | 11.15348517 |
| MRPL35 | 8.636258655 |
| MRPL36 | 12.34125494 |
| MRPL37 | 12.15687059 |
| MRPL38 | 10.12463823 |
| MRPL39 | 9.126250057 |
| MRPL4 | 6.71235566 |
| MRPL40 | 11.94402235 |
| MRPL41 | 11.84106204 |
| MRPL42 | 8.341806295 |
| MRPL43 | 9.468486656 |
| MRPL44 | 10.11012822 |
| MRPL45 | 10.3639973 |
| MRPL46 | 11.62522863 |
| MRPL47 | 8.060364406 |
| MRPL48 | 10.47707266 |
| MRPL49 | 10.85939402 |
| MRPL50 | 11.25643197 |
| MRPL51 | 12.82114025 |
| MRPL52 | 6.00934577 |
| MRPL53 | 11.11394674 |
| MRPL54 | 12.65206935 |
| MRPL55 | 7.593671144 |
| MRPL9 | 11.04693749 |
| MRPS10 | 11.586789 |
| MRPS11 | 11.74704505 |
| MRPS12 | 8.152477366 |
| MRPS14 | 7.777805567 |
| MRPS15 | 11.93411776 |
| MRPS16 | 10.6505913 |
| MRPS17 | 11.25820108 |
| MRPS18A | 9.972255629 |
| MRPS18B | 9.357585947 |
| MRPS18C | 11.9419694 |
| MRPS2 | 9.048073271 |
| MRPS21 | 10.3203064 |
| MRPS22 | 12.3348251 |
| MRPS23 | 9.504501058 |
| MRPS24 | 12.39625313 |
| MRPS25 | 7.099892308 |
| MRPS26 | 10.70529486 |
| MRPS27 | 10.25806962 |
| MRPS28 | 11.91543755 |
| MRPS30 | 10.92691977 |
| MRPS31 | 10.88433886 |
| MRPS33 | 10.39637408 |
| MRPS34 | 9.25193735 |
| MRPS35 | 11.16542655 |
| MRPS36 | 8.336277016 |
| MRPS5 | 10.56640493 |
| MRPS6 | 13.03396622 |
| MRPS7 | 11.35721791 |
| MRPS9 | 10.24323669 |
| MRRF | 7.888743755 |
| MRS2L | 6.807279535 |
| MRVI1 | 5.476969467 |
| MS4A2 | 5.837999711 |
| MS4A3 | 5.849956753 |
| MS4A6A | 11.77570132 |
| MS4A7 | 10.81416095 |
| MSC | 5.774734761 |
| MSH2 | 6.224050975 |
| MSH3 | 8.914289115 |
| MSH6 | 10.83570716 |
| MSI2 | 6.294703293 |
| MSL2L1 | 6.277652428 |
| MSL3L1 | 8.141108859 |
| MSN | 11.04435011 |
| MSR1 | 5.856866506 |
| MSRA | 10.24120309 |
| MSRB2 | 12.62961066 |
| MSRB3 | 6.674020111 |
| MST1 | 13.26679073 |
| MST150 | 5.994200617 |
| MSTO1 | 8.541084043 |
| MSX1 | 7.369113087 |
| MSX2 | 5.605523243 |
| MSX2P1 | 5.644756234 |
| MT | 7.361751305 |
| MT1A | 15.01566258 |
| MT1B | 7.372808286 |
| MT1E | 7.143605049 |
| MT1F | 14.44242925 |
| MT1G | 15.19460128 |
| MT1H | 9.965971212 |
| MT1JP | 6.814005371 |
| MT1M | 13.95775 |
| MT1X | 15.24779187 |
| MT2A | 15.2348409 |
| MTA1 | 7.825420717 |
| MTA2 | 9.034431025 |
| MTA3 | 8.378867489 |
| MTAC2D1 | 5.749567572 |
| MTAP | 8.65777778 |
| MTCH1 | 12.43564196 |
| MTCH2 | 9.016714528 |
| MTCP1 | 10.95476975 |
| MTDH | 11.71931539 |
| MTERFD1 | 9.927590505 |
| MTF1 | 7.843272885 |
| MTF2 | 8.460079141 |
| MTFMT | 8.999406349 |
| MTFR1 | 10.77034294 |
| MTG1 | 6.21124584 |
| MTHFD1 | 9.100004262 |
| MTHFD1L | 8.285419587 |
| MTHFD2 | 7.76884845 |
| MTHFD2L | 7.284091138 |
| MTHFR | 7.287159103 |
| MTHFS | 13.31028757 |
| MTIF2 | 10.60175305 |
| MTIF3 | 10.93185341 |
| MTM | 8.565472051 |
| MTM1 | 8.202707942 |
| MTMR1 | 5.537087407 |
| MTMR11 | 7.014750173 |
| MTMR12 | 7.397825781 |
| MTMR15 | 9.805082529 |
| MTMR2 | 6.773450593 |
| MTMR3 | 7.517628021 |
| MTMR4 | 9.426460763 |
| MTMR6 | 10.19907151 |
| MTMR9 | 8.824609777 |
| MTO1 | 6.268351663 |
| MTP18 | 9.331261951 |
| MTPN | 9.872837644 |
| MTR | 7.925655635 |
| MTRF1 | 9.373212643 |
| MTRF1L | 6.079121526 |
| MTRR | 10.96453767 |
| MTSS1 | 8.230038445 |
| MTTP | 12.103392 |
| MTUS1 | 5.626570942 |
| MTX1 | 9.329195756 |
| MTX2 | 9.561112249 |
| MTX3 | 7.900660347 |
| MUC1 | 6.455832102 |
| MUC13 | 6.019152639 |
| MUC15 | 5.791919616 |
| MUC4 | 6.309420558 |
| MUC5AC | 5.849811052 |
| MUCDHL | 7.497471384 |
| MUM1 | 8.859769096 |
| MUM1L1 | 5.780798547 |
| MUS81 | 7.650120983 |
| MUSTN1 | 6.063902131 |
| MUT | 11.67112622 |
| MUTED | 9.724702281 |
| MUTYH | 6.558495964 |
| MVD | 6.489318531 |
| MVK | 7.844260301 |
| MVP | 10.40699726 |
| MX1 | 11.28799868 |
| MXD1 | 8.055890085 |
| MXD3 | 5.659772032 |
| MXD4 | 10.10596369 |
| MXI1 | 6.478774412 |
| MXRA5 | 8.00240948 |
| MXRA7 | 6.443095916 |
| MXRA8 | 5.564153504 |
| MYADM | 9.656779212 |
| MYADML | 5.838068293 |
| MYB | 5.792422302 |
| MYBBP1A | 5.877406345 |
| MYBL2 | 5.729995909 |
| MYBPC1 | 5.751071189 |
| MYBPC3 | 5.879063032 |
| MYBPH | 6.09222071 |
| MYC | 10.26054202 |
| MYCBP | 5.752099185 |
| MYCBP2 | 8.984995217 |
| MYCL1 | 6.747425093 |
| MYCN | 6.170875813 |
| MYCT1 | 6.536315022 |
| MYD88 | 9.543156094 |
| MYEF2 | 6.772924724 |
| MYEOV | 6.215189298 |
| MYEOV2 | 6.133498402 |
| MYH10 | 10.40135318 |
| MYH11 | 8.347353429 |
| MYH14 | 6.219120396 |
| MYH9 | 11.97586281 |
| MYL4 | 5.694581815 |
| MYL5 | 8.631216783 |
| MYL6 | 14.6804342 |
| MYL7 | 5.690463771 |
| MYL9 | 6.493583799 |
| MYLIP | 9.022578 |
| MYLK | 8.717433372 |
| MYLK2 | 6.573345258 |
| MYNN | 8.980087715 |
| MYO10 | 9.644646852 |
| MYO16 | 5.903882491 |
| MYO18A | 7.819796207 |
| MYO19 | 6.390307909 |
| MYO1A | 5.819939608 |
| MYO1B | 9.809199674 |
| MYO1C | 7.515706176 |
| MYO1D | 7.204555728 |
| MYO1E | 6.64716844 |
| MYO1G | 6.166119118 |
| MYO5A | 7.311876379 |
| MYO5C | 8.576006002 |
| MYO6 | 6.999282838 |
| MYO7A | 7.040811563 |
| MYO9A | 7.346440107 |
| MYO9B | 7.43822669 |
| MYOM1 | 9.86657114 |
| MYOM2 | 8.08321053 |
| MYOT | 5.794328876 |
| MYOZ1 | 5.825787046 |
| MYRIP | 7.615221153 |
| MYST1 | 8.447907165 |
| MYST2 | 5.754539883 |
| MYST3 | 9.723744872 |
| MYT1 | 5.922883508 |
| MZF1 | 7.211853834 |
| N4BP2 | 8.025509527 |
| N6AMT2 | 9.292042719 |
| NAALAD2 | 6.107504694 |
| NAALADL2 | 5.714961985 |
| NAB1 | 8.920073067 |
| NAB2 | 6.704930179 |
| NACA | 9.514093074 |
| NACA2 | 5.873682923 |
| NACAP1 | 8.878097799 |
| NADK | 8.578435943 |
| NADSYN1 | 8.854839116 |
| NAE1 | 8.285334762 |
| NAF1 | 6.965617909 |
| NAG | 7.467732401 |
| NAG18 | 5.983620654 |
| NAG6 | 6.07434243 |
| NAGA | 7.538173351 |
| NAGK | 10.64224209 |
| NAGLU | 9.890142572 |
| NAGPA | 8.01342869 |
| NAGS | 7.916794459 |
| NALP1 | 5.805304627 |
| NANOG | 5.80429503 |
| NANP | 5.837955471 |
| NANS | 10.9035637 |
| NAP1L1 | 8.540053568 |
| NAP1L2 | 6.298465546 |
| NAP1L3 | 6.145538718 |
| NAP1L4 | 10.69537368 |
| NAP1L5 | 8.600450763 |
| NAP5 | 5.900842524 |
| NAPA | 7.176905446 |
| NAPB | 6.785318286 |
| NAPEPLD | 5.681799628 |
| NAPG | 9.132174653 |
| NAPRT1 | 11.32537866 |
| NAPSA | 6.266052673 |
| NAPSB | 6.444124029 |
| NARF | 9.574750045 |
| NARFL | 6.157294802 |
| NARG1 | 7.856344692 |
| NARG1L | 6.653312146 |
| NARG2 | 6.311626209 |
| NARS | 11.26516133 |
| NARS2 | 9.340675558 |
| NASP | 7.549955711 |
| NAT1 | 7.830268696 |
| NAT10 | 9.732682527 |
| NAT12 | 7.311803446 |
| NAT14 | 6.30712179 |
| NAT2 | 11.14666229 |
| NAT5 | 13.02649918 |
| NAT6 | 5.705684454 |
| NAT8 | 9.670046977 |
| NAT8L | 9.374180381 |
| NAT9 | 8.015220247 |
| NAV1 | 5.788429228 |
| NAV2 | 8.185395315 |
| NBEA | 5.471200501 |
| NBEAL1 | 5.5774019 |
| NBEAL2 | 5.695041753 |
| NBL1 | 7.068104431 |
| NBLA10383 | 7.193311601 |
| NBN | 9.724015571 |
| NBPF1 | 8.36175366 |
| NBPF10 | 6.633847484 |
| NBPF14 | 6.018284246 |
| NBPF9 | 6.104458755 |
| NBR1 | 7.119840841 |
| NCALD | 8.666847003 |
| NCAM1 | 6.375393453 |
| NCAPD2 | 6.540816684 |
| NCAPD3 | 6.660943496 |
| NCAPG | 6.347359568 |
| NCAPH2 | 6.497430827 |
| NCBP1 | 10.13243819 |
| NCBP2 | 9.809435297 |
| NCDN | 5.744426038 |
| NCF1 | 5.741227087 |
| NCF2 | 7.702184964 |
| NCF4 | 7.060302886 |
| NCK1 | 9.162268231 |
| NCK2 | 9.309729729 |
| NCKAP1 | 12.17064687 |
| NCKIPSD | 7.95319492 |
| NCL | 9.029334132 |
| NCLN | 10.38745173 |
| NCOA1 | 8.349863809 |
| NCOA2 | 5.434794657 |
| NCOA3 | 9.396864134 |
| NCOA4 | 14.02578916 |
| NCOA5 | 7.531962584 |
| NCOA6 | 8.670542649 |
| NCOA7 | 12.2812944 |
| NCOR1 | 8.089229866 |
| NCOR2 | 8.226476997 |
| NCR3 | 6.788281837 |
| NCSTN | 11.45006135 |
| NDC80 | 5.9331986 |
| NDE1 | 8.226808065 |
| NDEL1 | 10.6062488 |
| NDFIP1 | 9.692357846 |
| NDFIP2 | 11.67755066 |
| NDN | 8.015576512 |
| NDNL2 | 6.67732183 |
| NDRG1 | 9.927904266 |
| NDRG2 | 10.93678642 |
| NDRG3 | 8.140969595 |
| NDRG4 | 5.813551217 |
| NDST1 | 8.670940319 |
| NDST2 | 6.464843159 |
| NDUFA1 | 14.41563773 |
| NDUFA10 | 10.84585042 |
| NDUFA11 | 12.83066532 |
| NDUFA12 | 12.24558061 |
| NDUFA12L | 11.56103696 |
| NDUFA13 | 11.59664644 |
| NDUFA2 | 13.26658201 |
| NDUFA3 | 13.10146776 |
| NDUFA4 | 14.71720956 |
| NDUFA4L2 | 6.413927379 |
| NDUFA5 | 5.811459023 |
| NDUFA6 | 10.15457127 |
| NDUFA7 | 10.84426352 |
| NDUFA8 | 12.19918207 |
| NDUFA9 | 11.51249556 |
| NDUFAB1 | 13.69084111 |
| NDUFAF1 | 10.56674914 |
| NDUFB1 | 7.293908526 |
| NDUFB10 | 12.68775699 |
| NDUFB11 | 12.86490764 |
| NDUFB2 | 13.8771162 |
| NDUFB3 | 11.06667279 |
| NDUFB4 | 5.825958466 |
| NDUFB5 | 12.96970207 |
| NDUFB6 | 11.96238435 |
| NDUFB7 | 12.71748931 |
| NDUFB8 | 13.86257522 |
| NDUFB9 | 12.89649226 |
| NDUFC1 | 11.00957551 |
| NDUFC2 | 8.029272119 |
| NDUFS1 | 8.236867527 |
| NDUFS2 | 8.321717608 |
| NDUFS3 | 12.7217023 |
| NDUFS4 | 12.79260478 |
| NDUFS5 | 13.42584466 |
| NDUFS6 | 14.21144322 |
| NDUFS7 | 11.87432636 |
| NDUFS8 | 12.7762769 |
| NDUFV1 | 10.96958274 |
| NDUFV2 | 14.07838462 |
| NDUFV3 | 6.899762628 |
| NEB | 5.660439284 |
| NEBL | 6.002878082 |
| NECAB2 | 5.71134786 |
| NECAP1 | 9.776890862 |
| NECAP2 | 10.13630349 |
| NEDD1 | 5.561955316 |
| NEDD4 | 6.566927823 |
| NEDD4L | 6.852854411 |
| NEDD8 | 11.02564627 |
| NEDD9 | 6.309206812 |
| NEFH | 5.90470809 |
| NEFM | 6.494398607 |
| NEIL1 | 6.064998754 |
| NEIL2 | 9.13028986 |
| NEK1 | 6.611223719 |
| NEK3 | 7.449079904 |
| NEK6 | 10.52925508 |
| NEK7 | 5.553969013 |
| NEK8 | 6.995564293 |
| NELF | 8.172911421 |
| NENF | 6.915346314 |
| NEO1 | 9.224756393 |
| NES | 6.486293177 |
| NET1 | 9.686555778 |
| NETO2 | 6.117228584 |
| NEU1 | 9.437442022 |
| NEU4 | 7.966576121 |
| NEURL2 | 6.146733248 |
| NEUROD2 | 5.798213381 |
| NEUROD6 | 5.800280564 |
| NEXN | 6.670580134 |
| NF2 | 5.75234285 |
| NFAM1 | 6.356880384 |
| NFAT5 | 7.452904148 |
| NFATC1 | 6.560718142 |
| NFATC2IP | 6.590542105 |
| NFATC3 | 6.368605374 |
| NFE2 | 7.370641115 |
| NFE2L1 | 9.36766187 |
| NFE2L2 | 10.9479595 |
| NFE2L3 | 6.102014629 |
| NFIA | 9.413221349 |
| NFIB | 10.83757416 |
| NFIC | 7.844175759 |
| NFIL3 | 10.95505497 |
| NFIX | 8.77170947 |
| NFKB1 | 11.64139181 |
| NFKB2 | 6.39464442 |
| NFKBIA | 13.88655474 |
| NFKBIB | 7.069258076 |
| NFKBID | 6.124883348 |
| NFKBIE | 7.996798218 |
| NFKBIL1 | 7.025934906 |
| NFKBIZ | 10.63391555 |
| NFS1 | 5.977151475 |
| NFU1 | 10.78554988 |
| NFX1 | 8.57468031 |
| NFXL1 | 8.374707392 |
| NFYA | 5.641093538 |
| NFYB | 7.206572765 |
| NFYC | 8.513977281 |
| NGEF | 7.977403921 |
| NGFR | 5.639349307 |
| NGFRAP1 | 10.01189705 |
| NGLY1 | 10.23520569 |
| NGRN | 10.4660322 |
| NHEDC2 | 8.411362544 |
| NHEJ1 | 5.933001555 |
| NHLH1 | 6.085810899 |
| NHLRC2 | 8.095599281 |
| NHLRC3 | 7.922409436 |
| NHP2L1 | 10.36642889 |
| NIBP | 6.484821392 |
| NICN1 | 9.103609424 |
| NID1 | 6.098306157 |
| NID2 | 5.814245574 |
| NIF3L1 | 11.25423027 |
| NIN | 7.399425574 |
| NINJ1 | 11.61383467 |
| NINJ2 | 10.7712463 |
| NIP30 | 7.515341703 |
| NIP7 | 10.74319065 |
| NIPA1 | 7.964029789 |
| NIPA2 | 8.33938793 |
| NIPBL | 6.729040845 |
| NIPSNAP1 | 10.88888716 |
| NIPSNAP3A | 8.3182018 |
| NISCH | 7.1503327 |
| NIT1 | 6.893122685 |
| NIT2 | 11.75070532 |
| NKAP | 6.85232927 |
| NKAPL | 5.603277111 |
| NKD2 | 6.581409637 |
| NKG7 | 7.930761884 |
| NKIRAS1 | 9.8861743 |
| NKIRAS2 | 6.947897735 |
| NKRF | 8.015774763 |
| NKTR | 7.852669944 |
| NKX1-1 | 5.811251394 |
| NKX3-1 | 7.985961984 |
| NKX6-1 | 5.552857324 |
| NLE1 | 5.995571136 |
| NLF1 | 6.008795194 |
| NLF2 | 9.429121097 |
| NLGN2 | 6.332592086 |
| NLGN4X | 5.615541545 |
| NLGN4Y | 6.42104927 |
| NLK | 7.439747626 |
| NLN | 6.883417468 |
| NLP | 5.692196814 |
| NLRC4 | 6.16574088 |
| NLRP12 | 5.932925829 |
| NLRP3 | 6.509726142 |
| NLRX1 | 7.302156971 |
| NMB | 6.686622898 |
| NMD3 | 9.386831448 |
| NME1 | 10.06725617 |
| NME1-NME2 | 8.760099457 |
| NME2 | 9.684019185 |
| NME3 | 8.924871867 |
| NME4 | 12.14670253 |
| NME6 | 6.40934575 |
| NME7 | 9.288185243 |
| NMI | 11.00099515 |
| NMNAT1 | 5.735774543 |
| NMNAT2 | 5.688810882 |
| NMNAT3 | 5.862573899 |
| NMRAL1 | 11.58627625 |
| NMT1 | 8.964265559 |
| NMT2 | 10.44560759 |
| NNMT | 14.99849551 |
| NNT | 8.893511405 |
| NOB1 | 7.574464469 |
| NOC2L | 7.650148422 |
| NOC3L | 8.295043886 |
| NOC4L | 6.243215026 |
| NODAL | 5.785337127 |
| NOL1 | 10.45497991 |
| NOL11 | 10.48893497 |
| NOL14 | 9.238275311 |
| NOL3 | 8.226387908 |
| NOL4 | 5.590661202 |
| NOL5A | 11.08429266 |
| NOL6 | 10.35777872 |
| NOL7 | 12.39292896 |
| NOL8 | 8.866321598 |
| NOL9 | 5.925767401 |
| NOLA1 | 8.177633079 |
| NOLA2 | 10.94605178 |
| NOLA3 | 13.33440743 |
| NOLC1 | 7.470070964 |
| NOM1 | 5.492614649 |
| NOMO1 | 8.332777728 |
| NOMO2 | 7.262468827 |
| NOMO3 | 5.426419007 |
| NONO | 6.97163272 |
| NOP5/NOP58 | 10.96658412 |
| NOS1AP | 5.393349708 |
| NOS3 | 8.370491968 |
| NOSIP | 10.51981853 |
| NOSTRIN | 7.447380261 |
| NOTCH1 | 8.715562782 |
| NOTCH2 | 8.674878883 |
| NOTCH3 | 5.6228187 |
| NOTCH4 | 7.097591707 |
| NOX1 | 5.546084554 |
| NOX4 | 5.545300665 |
| NOXA1 | 5.949910064 |
| NOXO1 | 6.333337878 |
| NP | 11.50309522 |
| N-PAC | 9.268032312 |
| NPAL1 | 7.488921115 |
| NPAL2 | 5.623084274 |
| NPAL3 | 7.699935761 |
| NPAS2 | 7.211188419 |
| NPAS3 | 6.032091172 |
| NPAT | 7.508417554 |
| NPC1 | 8.552731029 |
| NPC1L1 | 8.831564016 |
| NPC2 | 13.76656965 |
| NPCDR1 | 5.760060258 |
| NPDC1 | 8.608160504 |
| NPEPL1 | 8.021174491 |
| NPEPPS | 5.75025197 |
| NPFF | 5.682363163 |
| NPFFR2 | 5.659104749 |
| NPHP3 | 6.212572985 |
| NPHS1 | 5.925819247 |
| NPHS2 | 5.684028385 |
| NPIP | 6.228215071 |
| NPL | 9.341504463 |
| NPLOC4 | 8.322239936 |
| NPM1 | 14.12281624 |
| NPM3 | 5.646943906 |
| NPR1 | 7.411440112 |
| NPR2 | 6.124805675 |
| NPR3 | 5.619056095 |
| NPSR1 | 6.955308861 |
| NPTX2 | 7.43002998 |
| NPY1R | 6.99523402 |
| NPY5R | 6.228252614 |
| NQO1 | 7.955445433 |
| NQO2 | 10.56690243 |
| NR0B2 | 10.67365855 |
| NR1D2 | 7.072174217 |
| NR1H2 | 8.907498647 |
| NR1H3 | 11.46668071 |
| NR1H4 | 10.68379632 |
| NR1I2 | 5.954965967 |
| NR1I3 | 9.208877868 |
| NR2C1 | 6.962467123 |
| NR2C2 | 6.694110643 |
| NR2C2AP | 7.883104175 |
| NR2E1 | 5.781223194 |
| NR2E3 | 6.589547397 |
| NR2F1 | 8.217674323 |
| NR2F2 | 7.222859362 |
| NR2F6 | 10.58412994 |
| NR3C1 | 7.202467754 |
| NR3C2 | 6.822088072 |
| NR4A2 | 7.793493565 |
| NR5A1 | 6.576630346 |
| NR5A2 | 7.753040064 |
| NRAP | 5.707348983 |
| NRAS | 8.758305832 |
| NRBF2 | 10.78101235 |
| NRBP1 | 7.584234946 |
| NRBP2 | 9.184436254 |
| NRD1 | 11.12833577 |
| NRG1 | 6.115280124 |
| NRG2 | 5.478399451 |
| NRG3 | 5.609332139 |
| NRG4 | 6.70954056 |
| NRGN | 5.848068124 |
| NRIP1 | 10.89402164 |
| NRIP2 | 5.659555659 |
| NRIP3 | 8.49637416 |
| NRK | 5.826052339 |
| NRM | 5.761490177 |
| NRN1 | 5.784249762 |
| NRP1 | 9.311660538 |
| NRSN2 | 5.795929715 |
| NRTN | 6.561851245 |
| NRXN1 | 5.880793374 |
| NRXN2 | 7.459657882 |
| NRXN3 | 6.557555788 |
| NSBP1 | 7.386877191 |
| NSD1 | 5.649020887 |
| NSDHL | 10.84577862 |
| NSF | 7.654531865 |
| NSFL1C | 9.119120966 |
| NSMAF | 8.72579692 |
| NSMCE1 | 10.09827488 |
| NSMCE2 | 9.436827714 |
| NSMCE4A | 11.08458663 |
| NSUN2 | 11.4927607 |
| NSUN3 | 6.841286299 |
| NSUN4 | 8.21336865 |
| NSUN5 | 7.426486442 |
| NSUN5C | 5.911523096 |
| NSUN6 | 9.999425788 |
| NT5C | 10.4788741 |
| NT5C2 | 10.7752204 |
| NT5C3 | 7.939906251 |
| NT5C3L | 9.683426478 |
| NT5DC1 | 9.837274623 |
| NT5DC2 | 5.755374375 |
| NT5DC3 | 8.409265049 |
| NT5E | 9.548508503 |
| NTAN1 | 9.807874728 |
| NTE | 8.589839634 |
| NTF3 | 7.211885021 |
| NTF5 | 5.740939332 |
| NTHL1 | 10.14210746 |
| NTN4 | 6.159468015 |
| NTRK1 | 5.545464126 |
| NTRK3 | 5.879803254 |
| NTS | 6.057943077 |
| NUAK1 | 9.605348374 |
| NUAK2 | 5.59040649 |
| NUB1 | 8.633921598 |
| NUBP1 | 8.90839293 |
| NUBP2 | 7.395846241 |
| NUBPL | 9.215294885 |
| NUCB1 | 14.53771095 |
| NUCB2 | 12.09510231 |
| NUCKS1 | 9.198973087 |
| NUDC | 11.24835983 |
| NUDCD1 | 6.530915032 |
| NUDCD2 | 9.517653936 |
| NUDCD3 | 8.124963019 |
| NUDT1 | 9.465293084 |
| NUDT12 | 6.20758357 |
| NUDT13 | 5.839679513 |
| NUDT14 | 9.333508758 |
| NUDT15 | 9.217848258 |
| NUDT16 | 7.475723096 |
| NUDT16L1 | 9.061037339 |
| NUDT18 | 8.348136803 |
| NUDT2 | 10.37114858 |
| NUDT21 | 8.71312099 |
| NUDT22 | 9.025659627 |
| NUDT3 | 10.00225244 |
| NUDT5 | 11.90410831 |
| NUDT6 | 7.931672152 |
| NUDT8 | 9.118711273 |
| NUDT9 | 10.5980655 |
| NUFIP1 | 5.551176177 |
| NUFIP2 | 8.536566996 |
| NUMA1 | 5.674357301 |
| NUMB | 9.895970053 |
| NUMBL | 5.765521038 |
| NUP107 | 9.963374385 |
| NUP133 | 8.446665267 |
| NUP153 | 8.459351816 |
| NUP155 | 8.552435805 |
| NUP160 | 9.300967795 |
| NUP188 | 7.067222947 |
| NUP205 | 9.660931919 |
| NUP210 | 6.922600242 |
| NUP214 | 8.704202565 |
| NUP35 | 8.906803602 |
| NUP37 | 10.29896496 |
| NUP43 | 6.799924155 |
| NUP50 | 7.158714494 |
| NUP54 | 8.947943276 |
| NUP62 | 11.18556751 |
| NUP62CL | 7.022901902 |
| NUP85 | 9.625065408 |
| NUP88 | 11.72717152 |
| NUP93 | 9.39191279 |
| NUP98 | 6.191024478 |
| NUPL2 | 9.05936336 |
| NUSAP1 | 8.284167962 |
| NUTF2 | 9.289838698 |
| NVL | 7.26742565 |
| NXF1 | 7.846665139 |
| NXF3 | 5.640182241 |
| NXN | 6.50820062 |
| NXPH1 | 5.654350231 |
| NXT1 | 10.48852866 |
| NXT2 | 8.97839049 |
| NYD-SP11 | 6.102118718 |
| NYD-SP18 | 6.070795691 |
| NYD-SP21 | 6.138062273 |
| NY-REN-7 | 7.475350836 |
| NY-SAR-48 | 6.599884127 |
| OACT1 | 7.479697693 |
| OAF | 13.14374606 |
| OAS1 | 7.55299895 |
| OAS2 | 7.227341937 |
| OAS3 | 6.844218886 |
| OASL | 6.190027061 |
| OAT | 9.691584504 |
| OATL1 | 5.870058215 |
| OAZ1 | 15.04322896 |
| OAZ2 | 10.2280934 |
| OAZ3 | 7.529539669 |
| OBFC1 | 9.155449374 |
| OBFC2A | 9.425254145 |
| OBFC2B | 8.754420646 |
| OBP2A | 5.74778207 |
| OCEL1 | 10.07818011 |
| OCIAD1 | 8.635025098 |
| OCIAD2 | 11.74715221 |
| OCLN | 5.850998366 |
| OCM | 6.091839635 |
| OCRL | 8.671321443 |
| ODC1 | 11.82935976 |
| ODF2 | 7.437141391 |
| ODF2L | 6.228104245 |
| ODF3L2 | 5.716072257 |
| ODZ1 | 8.136294819 |
| OFD1 | 6.224920886 |
| OGDH | 7.265939251 |
| OGDHL | 9.079856654 |
| OGFOD1 | 8.05160737 |
| OGFOD2 | 5.591181446 |
| OGFR | 8.812716707 |
| OGFRL1 | 6.147984718 |
| OGG1 | 5.874584777 |
| OGT | 9.549409824 |
| OIP5 | 5.948563889 |
| OIT3 | 9.138526649 |
| OKL38 | 10.85474508 |
| OLAH | 5.688580485 |
| OLFM1 | 7.618446389 |
| OLFML1 | 7.492871979 |
| OLFML2B | 5.729897983 |
| OLFML3 | 7.294765806 |
| OLR1 | 5.467253245 |
| OMA1 | 9.16144909 |
| OMP | 5.687709957 |
| ONECUT1 | 6.757108607 |
| ONECUT2 | 5.541635496 |
| OPA1 | 8.209780023 |
| OPA3 | 6.141977165 |
| OPLAH | 7.818133946 |
| OPN3 | 7.742885232 |
| OPRD1 | 5.787257655 |
| OPRS1 | 7.846152349 |
| OPTN | 10.53020425 |
| OR10G3 | 6.878244748 |
| OR10G7 | 5.971422872 |
| OR11G2 | 5.4293872 |
| OR12D2 | 5.861854865 |
| OR12D3 | 5.714834604 |
| OR13C3 | 5.773840334 |
| OR14I1 | 6.0238588 |
| OR14J1 | 5.571243952 |
| OR1B1 | 6.133974377 |
| OR1G1 | 5.752544734 |
| OR1L8 | 5.899427951 |
| OR1N1 | 5.496450646 |
| OR2A1 | 5.567614628 |
| OR2A14 | 5.857042351 |
| OR2A2 | 6.036290034 |
| OR2A20P | 5.564604406 |
| OR2A42 | 6.430571358 |
| OR2B2 | 7.137445305 |
| OR2C1 | 5.856096989 |
| OR2D2 | 5.657458342 |
| OR2D3 | 5.300562145 |
| OR2H2 | 5.85190678 |
| OR2J2 | 6.251805874 |
| OR2L1P | 5.571433918 |
| OR2L3 | 5.767062496 |
| OR2M2 | 5.741479774 |
| OR2T2 | 6.224045001 |
| OR2T33 | 6.011860846 |
| OR2T35 | 8.126256215 |
| OR2W1 | 5.561833847 |
| OR2W5 | 5.996396221 |
| OR2Y1 | 5.685394398 |
| OR3A2 | 5.583983762 |
| OR4C13 | 5.930122096 |
| OR4C15 | 6.351595837 |
| OR4D10 | 5.724381299 |
| OR4F5 | 5.7256448 |
| OR4S1 | 6.132352495 |
| OR51B6 | 5.850362761 |
| OR51D1 | 5.450827341 |
| OR51G1 | 5.678092596 |
| OR51V1 | 5.6031446 |
| OR52A4 | 5.819219469 |
| OR52A5 | 5.87046127 |
| OR52D1 | 5.367441401 |
| OR52E6 | 5.997720721 |
| OR52N2 | 6.185895389 |
| OR56B1 | 7.631382091 |
| OR5H6 | 5.647055171 |
| OR5M11 | 5.767286851 |
| OR5R1 | 5.833515436 |
| OR6C4 | 5.691168523 |
| OR6T1 | 6.124545254 |
| OR6V1 | 5.737253852 |
| OR7A17 | 5.485988969 |
| OR7C1 | 5.809948481 |
| OR7C2 | 6.069432177 |
| OR7D2 | 5.788360613 |
| OR8B8 | 5.886399782 |
| OR8G2 | 5.729566698 |
| OR9A4 | 7.215117877 |
| ORAI1 | 7.095336879 |
| ORAI3 | 6.693156279 |
| ORAOV1 | 7.988823669 |
| ORC1L | 6.30277039 |
| ORC2L | 8.786644963 |
| ORC3L | 8.49153751 |
| ORC4L | 6.110338763 |
| ORC5L | 8.424782703 |
| ORC6L | 6.206507435 |
| ORF1-FL49 | 9.789756327 |
| ORM1 | 15.81408262 |
| ORM2 | 15.71348457 |
| ORMDL1 | 10.05786656 |
| ORMDL2 | 7.171581059 |
| ORMDL3 | 10.2902768 |
| OS9 | 9.253605407 |
| OSBP | 12.19098738 |
| OSBP2 | 5.568438032 |
| OSBPL10 | 6.39453025 |
| OSBPL11 | 8.294744195 |
| OSBPL1A | 7.350374743 |
| OSBPL2 | 9.146497191 |
| OSBPL3 | 5.529146528 |
| OSBPL5 | 6.186791528 |
| OSBPL6 | 7.16466778 |
| OSBPL7 | 5.600354557 |
| OSBPL8 | 8.947395197 |
| OSBPL9 | 11.80095136 |
| OSGEP | 9.053307652 |
| OSGEPL1 | 6.627117595 |
| OSGIN2 | 8.927476067 |
| OSM | 5.986247925 |
| OSMR | 6.424802418 |
| OSRF | 7.579305545 |
| OSTalpha | 11.62207754 |
| OSTbeta | 6.350886896 |
| OSTF1 | 11.39271289 |
| OSTM1 | 8.2327568 |
| OTC | 11.84077271 |
| OTOF | 5.878356856 |
| OTOP3 | 6.102911786 |
| OTUB1 | 5.518614499 |
| OTUB2 | 6.198060984 |
| OTUD1 | 7.676263991 |
| OTUD4 | 9.560835685 |
| OTUD5 | 8.750011704 |
| OTUD6A | 5.532009351 |
| OTUD6B | 6.983597571 |
| OVCA2 | 6.959384146 |
| OVGP1 | 8.582734507 |
| OXA1L | 10.1297886 |
| OXCT1 | 6.272733198 |
| OXCT2 | 6.501122123 |
| OXER1 | 6.451921203 |
| OXR1 | 10.74830549 |
| OXSM | 9.81473764 |
| OXSR1 | 9.570472168 |
| OXT | 7.459187956 |
| OXTR | 5.708481498 |
| P15RS | 9.217115441 |
| P18SRP | 5.436235055 |
| P2RX1 | 5.786504049 |
| P2RX2 | 5.973226867 |
| P2RX3 | 5.967062648 |
| P2RX4 | 9.038819756 |
| P2RX7 | 6.510383177 |
| P2RY10 | 5.755448808 |
| P2RY11 | 6.721838586 |
| P2RY13 | 7.672271533 |
| P2RY2 | 5.643963911 |
| P2RY4 | 5.857054209 |
| P2RY5 | 7.858142336 |
| P2RY6 | 6.21066528 |
| P4HA1 | 8.718357493 |
| P4HA2 | 8.643169123 |
| P4HA3 | 5.841186729 |
| P4HB | 13.95015475 |
| P704P | 7.005666383 |
| P76 | 7.273741359 |
| P8 | 11.3717784 |
| PA2G4 | 9.908084685 |
| PAAF1 | 9.063604453 |
| PABPC1 | 12.15944978 |
| PABPC4 | 9.361569173 |
| PABPN1 | 9.504543922 |
| PACRG | 6.795785386 |
| PACS1 | 6.617114041 |
| PACS2 | 5.940709481 |
| PACSIN2 | 11.05581992 |
| PACSIN3 | 8.142169018 |
| PADI4 | 7.104910904 |
| PAF1 | 10.67759107 |
| PAFAH1B1 | 9.878824624 |
| PAFAH1B2 | 6.827309162 |
| PAFAH1B3 | 6.527438832 |
| PAFAH2 | 9.271012535 |
| PAG1 | 6.601069955 |
| PAGE3 | 5.71878094 |
| PAH | 12.56877629 |
| PAICS | 11.83485783 |
| PAIP1 | 7.301391738 |
| PAIP2 | 9.041990392 |
| PAK1 | 7.30609616 |
| PAK1IP1 | 10.88588303 |
| PAK2 | 8.276663203 |
| PAK4 | 7.114925869 |
| PAK7 | 6.077308382 |
| PALLD | 10.63066807 |
| PALM | 8.993299626 |
| PALM2-AKAP2 | 5.76697427 |
| PAM | 8.242232252 |
| PAMCI | 5.799696886 |
| PAN2 | 8.447155646 |
| PAN3 | 8.124029105 |
| PANK1 | 8.291201734 |
| PANK2 | 9.256286041 |
| PANK3 | 8.469985655 |
| PANK4 | 6.093688478 |
| PANX1 | 7.54759524 |
| PANX2 | 8.908311212 |
| PAOX | 7.350983386 |
| PAPD1 | 9.412997777 |
| PAPD4 | 9.836769961 |
| PAPD5 | 9.538247286 |
| PAPLN | 5.497339694 |
| PAPOLA | 11.32130155 |
| PAPPA | 6.535802721 |
| PAPPA2 | 5.751371656 |
| PAPSS1 | 9.583733437 |
| PAPSS2 | 11.57724105 |
| PAQR3 | 8.148591678 |
| PAQR4 | 7.61815012 |
| PAQR7 | 7.355141274 |
| PAQR8 | 5.877216088 |
| PAQR9 | 8.126401137 |
| PARC | 6.056758582 |
| PARD3 | 5.876684533 |
| PARD6A | 7.541448468 |
| PARD6G | 6.867616595 |
| PARG | 5.598334187 |
| PARK7 | 13.6299965 |
| PARN | 8.839815946 |
| PARP1 | 12.02140037 |
| PARP10 | 6.611107686 |
| PARP12 | 8.94309879 |
| PARP14 | 8.99952852 |
| PARP16 | 7.188131979 |
| PARP2 | 7.148454078 |
| PARP3 | 6.593224557 |
| PARP4 | 10.20043521 |
| PARP6 | 7.213968304 |
| PARP8 | 6.11335612 |
| PARP9 | 10.99679324 |
| PARS2 | 5.874120402 |
| PARVA | 7.952335443 |
| PARVB | 6.176044848 |
| PARVG | 6.359723427 |
| PASK | 6.555011489 |
| PATL1 | 9.956038155 |
| PAWR | 8.668766591 |
| PAX1 | 5.958931989 |
| PAX7 | 5.687195767 |
| PAX9 | 5.769381938 |
| PAXIP1 | 7.899532508 |
| PB1 | 6.227745237 |
| PBEF1 | 11.10240813 |
| PBK | 5.963012191 |
| PBLD | 10.05386371 |
| PBX1 | 6.106259633 |
| PBX2 | 8.448224838 |
| PBX3 | 10.4649558 |
| PC | 6.155334462 |
| PCAF | 10.82798887 |
| PCBD1 | 11.8949789 |
| PCBD2 | 6.609558348 |
| PCBP1 | 13.04899509 |
| PCBP2 | 12.45864524 |
| PCBP4 | 6.209371175 |
| PCCA | 11.29923462 |
| PCCB | 12.80337994 |
| PCDH10 | 7.373725245 |
| PCDH11Y | 5.660769115 |
| PCDH12 | 6.104057822 |
| PCDH17 | 7.946430336 |
| PCDH18 | 5.737018335 |
| PCDH19 | 5.664273519 |
| PCDH20 | 7.155980139 |
| PCDH24 | 8.321351894 |
| PCDH7 | 5.715725692 |
| PCDH9 | 6.104303488 |
| PCDHA10 | 5.749049372 |
| PCDHA9 | 5.613474674 |
| PCDHAC2 | 7.193512538 |
| PCDHB19P | 6.237875149 |
| PCDHGB5 | 6.563313119 |
| PCDHGB6 | 7.558458902 |
| PCF11 | 7.872679076 |
| PCGF1 | 7.065277093 |
| PCGF2 | 8.521592922 |
| PCGF5 | 7.412318096 |
| PCGF6 | 7.926847445 |
| PCID2 | 11.08717126 |
| PCIF1 | 6.251723709 |
| PCK1 | 10.34938686 |
| PCK2 | 9.596329952 |
| PCM1 | 10.5898406 |
| PCMT1 | 12.53863408 |
| PCMTD1 | 9.851752944 |
| PCMTD2 | 8.635387484 |
| PCNA | 8.620609691 |
| PCNP | 11.59295024 |
| PCNT2 | 8.444376846 |
| PCNX | 9.298754382 |
| PCNXL2 | 6.698147987 |
| PCNXL3 | 7.209528489 |
| PCOLCE | 8.71842522 |
| PCOLCE2 | 9.947097523 |
| PCOLN3 | 8.599518679 |
| PCSK5 | 7.216886036 |
| PCSK6 | 6.900527369 |
| PCSK7 | 8.764875648 |
| PCSK9 | 7.849641325 |
| PCTK1 | 5.62064584 |
| PCTK2 | 5.783607365 |
| PCTK3 | 5.348548429 |
| PCTP | 9.809047564 |
| PCYOX1 | 10.10660849 |
| PCYOX1L | 5.598172523 |
| PCYT1A | 6.199281319 |
| PCYT2 | 8.890242718 |
| PDCD10 | 8.386451114 |
| PDCD11 | 8.594288416 |
| PDCD1LG2 | 5.619372239 |
| PDCD2 | 9.548335635 |
| PDCD2L | 8.968066372 |
| PDCD4 | 7.42847096 |
| PDCD5 | 10.71021193 |
| PDCD6 | 12.06380804 |
| PDCD6IP | 8.371665389 |
| PDCD7 | 8.467382962 |
| PDCL | 6.208370346 |
| PDCL3 | 9.870686128 |
| PDDC1 | 7.249637767 |
| PDE12 | 8.653211068 |
| PDE1A | 6.576281434 |
| PDE2A | 6.452444411 |
| PDE3B | 7.9286864 |
| PDE4B | 5.378540671 |
| PDE4D | 5.33943723 |
| PDE4DIP | 7.214301746 |
| PDE5A | 6.556760234 |
| PDE6A | 5.743342743 |
| PDE6B | 6.295284657 |
| PDE6C | 8.741800736 |
| PDE6D | 8.738172509 |
| PDE6G | 6.47167387 |
| PDE7A | 5.733687726 |
| PDE7B | 7.674619974 |
| PDE8A | 7.703020931 |
| PDE8B | 5.794410971 |
| PDE9A | 6.567220378 |
| PDF | 6.511545259 |
| PDGFC | 8.799263805 |
| PDGFD | 6.308171588 |
| PDGFRA | 7.541797426 |
| PDGFRB | 8.802138808 |
| PDGFRL | 7.110811391 |
| PDHA1 | 11.57870858 |
| PDHB | 12.94538116 |
| PDHX | 10.2116515 |
| PDIA3 | 5.992318651 |
| PDIA3P | 6.659831165 |
| PDIA4 | 13.26997092 |
| PDIA5 | 10.20849173 |
| PDIA6 | 11.23568789 |
| PDIK1L | 8.208562423 |
| PDK2 | 6.817862514 |
| PDK3 | 8.493685144 |
| PDK4 | 9.545972029 |
| PDLIM1 | 10.64540762 |
| PDLIM3 | 7.84375898 |
| PDLIM5 | 5.986100103 |
| PDLIM7 | 6.26689107 |
| PDP2 | 5.783805604 |
| PDPK1 | 7.673021714 |
| PDPN | 6.184040389 |
| PDPR | 7.018646596 |
| PDRG1 | 8.651178389 |
| PDS5A | 7.684690269 |
| PDS5B | 7.289378941 |
| PDSS1 | 8.677405197 |
| PDSS2 | 10.65339111 |
| PDXDC1 | 6.515525617 |
| PDXK | 10.78670242 |
| PDXP | 11.16061911 |
| PDZD8 | 8.601508898 |
| PDZK1 | 7.651271096 |
| PDZK3 | 5.516929153 |
| PEA15 | 11.04345892 |
| PEBP1 | 13.84734969 |
| PEBP4 | 5.721259541 |
| PECAM1 | 8.491810225 |
| PECI | 12.81802148 |
| PECR | 11.68442621 |
| PEF1 | 9.794070823 |
| PEG3 | 7.85530084 |
| PELI1 | 8.924334565 |
| PELI2 | 6.447531958 |
| PELO | 11.49381255 |
| PELP1 | 8.158921851 |
| PEMT | 10.36045387 |
| PEPD | 11.97945105 |
| PER1 | 6.225353383 |
| PER2 | 10.39967177 |
| PER3 | 5.503028572 |
| PERLD1 | 9.178858884 |
| PERP | 11.57787509 |
| PES1 | 9.176603505 |
| PET112L | 10.28789717 |
| PEX1 | 7.810979316 |
| PEX10 | 9.960097134 |
| PEX11A | 10.03157377 |
| PEX11B | 9.992217289 |
| PEX11G | 9.817716887 |
| PEX13 | 9.851726329 |
| PEX14 | 7.522378647 |
| PEX16 | 8.708076623 |
| PEX19 | 9.979065264 |
| PEX26 | 8.61173238 |
| PEX3 | 9.805839818 |
| PEX5 | 11.65109237 |
| PEX6 | 6.769826822 |
| PEX7 | 9.002652161 |
| PFAAP5 | 7.998467304 |
| PFAS | 7.513852297 |
| PFDN1 | 9.179583612 |
| PFDN2 | 8.852045309 |
| PFDN5 | 13.74177554 |
| PFDN6 | 9.963346381 |
| PFKFB1 | 5.639343291 |
| PFKFB2 | 6.162192001 |
| PFKFB3 | 7.438453463 |
| PFKFB4 | 6.996847937 |
| PFKL | 8.32836662 |
| PFKM | 6.37506351 |
| PFKP | 7.081096185 |
| PFN1 | 12.48852024 |
| PFN2 | 7.516533921 |
| PFN4 | 6.214588463 |
| PFTK1 | 8.496846123 |
| PGA5 | 6.9647033 |
| PGAM1 | 8.816910718 |
| PGAM4 | 11.04596565 |
| PGBD1 | 6.347671843 |
| PGBD2 | 6.523236364 |
| PGBD3 | 6.145173064 |
| PGBD4 | 5.365275875 |
| PGBD5 | 5.783791367 |
| PGCP | 8.704888032 |
| PGD | 9.906392899 |
| PGGT1B | 6.669775216 |
| PGK1 | 12.73445406 |
| PGK2 | 5.893593439 |
| PGLS | 10.06242203 |
| PGLYRP1 | 6.093136345 |
| PGM1 | 13.56556949 |
| PGM2 | 7.948644022 |
| PGM2L1 | 5.622077929 |
| PGM3 | 10.40960927 |
| PGM5 | 8.431328887 |
| PGPEP1 | 7.252539534 |
| PGRMC1 | 13.84268768 |
| PGRMC2 | 12.6681753 |
| PH-4 | 6.223172603 |
| PHACS | 8.847235635 |
| PHACTR2 | 9.932629548 |
| PHACTR3 | 6.173097125 |
| PHACTR4 | 10.37142659 |
| PHB | 11.76058494 |
| PHB2 | 10.90840822 |
| PHC1 | 6.072012771 |
| PHC2 | 6.496914269 |
| PHCA | 8.804663243 |
| PHF1 | 6.502504195 |
| PHF10 | 11.33311966 |
| PHF11 | 10.66201995 |
| PHF12 | 6.449238936 |
| PHF13 | 10.3209784 |
| PHF14 | 6.77307921 |
| PHF15 | 6.509784227 |
| PHF16 | 9.361690911 |
| PHF17 | 7.570284735 |
| PHF19 | 6.755822002 |
| PHF2 | 7.188480255 |
| PHF20 | 6.769511563 |
| PHF20L1 | 8.790054116 |
| PHF21A | 9.319861385 |
| PHF23 | 9.144899797 |
| PHF3 | 8.045911828 |
| PHF5A | 8.565292084 |
| PHGDH | 12.19555273 |
| PHIP | 8.852112808 |
| PHKA2 | 10.36584655 |
| PHKB | 7.704272977 |
| PHKG2 | 6.615739638 |
| PHLDA1 | 11.19806353 |
| PHLDA2 | 7.049940803 |
| PHLDB1 | 7.223278283 |
| PHLDB2 | 7.850062312 |
| PHLPP | 6.389401845 |
| PHLPPL | 6.486977884 |
| PHOSPHO2 | 7.629267256 |
| PHOX2A | 5.969889271 |
| PHPT1 | 11.48138001 |
| PHTF1 | 8.222277723 |
| PHTF2 | 8.753204748 |
| PHYH | 12.69941791 |
| PHYH2 | 12.52589537 |
| PHYHD1 | 9.609650545 |
| PHYHIP | 5.739489435 |
| PHYHIPL | 6.497211525 |
| PI3 | 6.837448022 |
| PI4K2A | 6.335119959 |
| PI4K2B | 10.36522597 |
| PI4KB | 8.529486899 |
| PIAS1 | 7.966053513 |
| PIAS2 | 7.303593864 |
| PIAS3 | 8.457119378 |
| PIAS4 | 10.35970381 |
| PIBF1 | 6.969391011 |
| PICALM | 7.126789625 |
| PID1 | 10.09716325 |
| PIGA | 7.585888654 |
| PIGB | 8.732798674 |
| PIGC | 9.025924666 |
| PIGF | 8.243278889 |
| PIGG | 7.036988295 |
| PIGH | 8.707080002 |
| PIGK | 10.07802186 |
| PIGL | 6.648277557 |
| PIGM | 10.48765785 |
| PIGN | 8.401730873 |
| PIGO | 7.807329801 |
| PIGQ | 9.156988846 |
| PIGR | 8.705657453 |
| PIGS | 7.008490524 |
| PIGT | 9.84962815 |
| PIGU | 9.996731154 |
| PIGV | 10.05767692 |
| PIGW | 8.244260129 |
| PIGX | 8.063874045 |
| PIGY | 12.36935203 |
| PIH1D1 | 8.528036767 |
| PIK3AP1 | 11.08505559 |
| PIK3C2A | 5.794807814 |
| PIK3C2B | 6.329449454 |
| PIK3C2G | 7.311274221 |
| PIK3C3 | 7.852682299 |
| PIK3CA | 7.185879259 |
| PIK3CB | 6.996222678 |
| PIK3CD | 6.81869482 |
| PIK3CG | 6.150090105 |
| PIK3IP1 | 6.85553916 |
| PIK3R1 | 8.452415314 |
| PIK3R2 | 10.97079576 |
| PIK3R3 | 5.817123217 |
| PIK3R4 | 9.231506049 |
| PIK4CA | 10.64638354 |
| PILRA | 6.219929956 |
| PILRB | 5.625064449 |
| PIM1 | 11.1512345 |
| PIM2 | 7.585778356 |
| PIM3 | 7.418126608 |
| PIN1 | 10.6050817 |
| PIN4 | 6.387607189 |
| PINK1 | 10.23450865 |
| PINX1 | 7.032240099 |
| PIP3-E | 8.851036656 |
| PIP4K2C | 7.917046062 |
| PIP5K1A | 6.447344149 |
| PIP5K1C | 5.662160744 |
| PIP5K2A | 7.430850504 |
| PIP5K2B | 7.919994082 |
| PIPOX | 12.10922626 |
| PIR | 10.75914787 |
| PISD | 8.924995297 |
| PITPNA | 6.764043377 |
| PITPNB | 12.12288211 |
| PITPNC1 | 7.122829206 |
| PITPNM1 | 7.627374741 |
| PITRM1 | 8.36021144 |
| PITX1 | 5.687369752 |
| PIWIL1 | 5.683183961 |
| PIWIL2 | 5.618828736 |
| PJA1 | 5.408935642 |
| PJA2 | 12.69121999 |
| PKD1 | 5.805504778 |
| PKD2 | 6.500593836 |
| PKD2L1 | 5.56690084 |
| PKIB | 6.886301735 |
| PKIG | 6.890977149 |
| PKLR | 6.837435439 |
| PKM2 | 5.396874119 |
| PKN1 | 6.764351707 |
| PKN2 | 6.897717024 |
| PKNOX1 | 7.919996252 |
| PKNOX2 | 6.005411051 |
| PKP1 | 5.684633952 |
| PKP2 | 8.655211359 |
| PKP3 | 5.723475617 |
| PKP4 | 9.311448193 |
| PLA1A | 9.254026165 |
| PLA2G12A | 6.984862147 |
| PLA2G12B | 12.21850156 |
| PLA2G15 | 7.311917972 |
| PLA2G1B | 6.581246961 |
| PLA2G2A | 11.60974955 |
| PLA2G2F | 5.518843475 |
| PLA2G4A | 6.705451292 |
| PLA2G4B | 7.527042473 |
| PLA2G4C | 8.4942702 |
| PLA2G6 | 5.835440337 |
| PLA2G7 | 8.359547732 |
| PLAA | 7.243266073 |
| PLAC1 | 5.776407583 |
| PLAC8 | 8.571293982 |
| PLAC9 | 8.25981133 |
| PLAG1 | 5.825756521 |
| PLAGL2 | 7.328277291 |
| PLAT | 6.046800403 |
| PLAU | 6.631300333 |
| PLAUR | 6.639627391 |
| PLB1 | 5.688914121 |
| PLCB1 | 7.212101947 |
| PLCB2 | 6.180328557 |
| PLCB3 | 5.954688383 |
| PLCD1 | 6.748608114 |
| PLCE1 | 5.873741438 |
| PLCG1 | 6.912354952 |
| PLCG2 | 9.018873636 |
| PLCL2 | 8.71041291 |
| PLCL4 | 5.867832393 |
| PLCXD1 | 7.83914288 |
| PLCXD3 | 6.317663948 |
| PLD1 | 8.56801496 |
| PLD2 | 5.75549701 |
| PLD3 | 7.396743696 |
| PLD4 | 5.877221264 |
| PLDN | 9.214194975 |
| PLEC1 | 5.811576288 |
| PLEK | 10.42392724 |
| PLEK2 | 8.827816311 |
| PLEKHA1 | 11.4405608 |
| PLEKHA2 | 7.360640948 |
| PLEKHA3 | 7.988154945 |
| PLEKHA4 | 8.510330994 |
| PLEKHA5 | 9.886463483 |
| PLEKHA6 | 6.759161464 |
| PLEKHA7 | 7.262067175 |
| PLEKHA8 | 5.804155717 |
| PLEKHA9 | 7.097989127 |
| PLEKHB1 | 5.757158088 |
| PLEKHB2 | 7.283637498 |
| PLEKHF1 | 9.386677426 |
| PLEKHF2 | 8.250278616 |
| PLEKHG1 | 6.441825553 |
| PLEKHG2 | 5.744440589 |
| PLEKHG3 | 8.716878961 |
| PLEKHG7 | 5.783319759 |
| PLEKHH1 | 5.710779771 |
| PLEKHH3 | 7.275225755 |
| PLEKHJ1 | 6.277823246 |
| PLEKHM1 | 6.815933983 |
| PLEKHM2 | 9.486724352 |
| PLEKHN1 | 5.684546829 |
| PLEKHO1 | 8.171431624 |
| PLEKHO2 | 8.139562507 |
| PLG | 13.21280608 |
| PLGLA1 | 8.310892515 |
| PLGLB2 | 8.736406295 |
| PLIN | 6.429027721 |
| PLK2 | 6.155691494 |
| PLK4 | 6.383439188 |
| PLLP | 6.525520905 |
| PLOD1 | 11.71355354 |
| PLOD2 | 10.01632016 |
| PLOD3 | 7.211409553 |
| PLP1 | 7.92019696 |
| PLP2 | 5.545473082 |
| PLRG1 | 10.99345308 |
| PLS1 | 7.628886726 |
| PLS3 | 11.67872415 |
| PLSCR1 | 10.14690744 |
| PLSCR4 | 11.27930291 |
| PLTP | 8.026887947 |
| PLVAP | 9.837350985 |
| PLXDC1 | 5.711276657 |
| PLXDC2 | 6.535796972 |
| PLXNA1 | 6.055213528 |
| PLXNB1 | 8.903079611 |
| PLXNB2 | 9.824507483 |
| PLXNC1 | 6.05262713 |
| PLXND1 | 6.742223277 |
| PM20D2 | 6.97469002 |
| PMAIP1 | 5.579510655 |
| PMEPA1 | 6.040132206 |
| PMF1 | 8.155182147 |
| PML | 5.759716163 |
| PMM1 | 11.07010521 |
| PMM2 | 10.1492808 |
| PMP22 | 7.126942384 |
| PMPCA | 10.70449056 |
| PMPCB | 12.22587355 |
| PMS1 | 6.110094087 |
| PMS2 | 6.691323622 |
| PMS2CL | 6.150123811 |
| PMS2L1 | 5.728507755 |
| PMS2L11 | 5.735721823 |
| PMS2L3 | 6.83595038 |
| PMS2L5 | 8.61047896 |
| PMVK | 10.06051559 |
| PNKD | 8.346816765 |
| PNKP | 9.817141161 |
| PNLIP | 5.976975517 |
| PNLIPRP2 | 6.540463796 |
| PNMA1 | 6.782672896 |
| PNMA2 | 5.48145308 |
| PNMA3 | 8.859481223 |
| PNMA6A | 6.54948142 |
| PNN | 8.667095951 |
| PNO1 | 9.59944364 |
| PNPLA2 | 6.247244184 |
| PNPLA4 | 6.147016045 |
| PNPLA5 | 5.973010282 |
| PNPLA7 | 9.246428219 |
| PNPLA8 | 9.627561285 |
| PNPO | 12.73263976 |
| PNPT1 | 7.601677425 |
| PNRC1 | 8.401036349 |
| PNRC2 | 6.700950152 |
| PODN | 7.81963629 |
| PODNL1 | 5.864001285 |
| PODXL | 5.927002824 |
| POF1B | 5.979558476 |
| POFUT1 | 7.663210817 |
| POFUT2 | 7.939343917 |
| POGK | 9.810717968 |
| POGZ | 6.147438728 |
| POLA | 7.993050625 |
| POLA2 | 8.511609507 |
| POLB | 10.42846213 |
| POLD1 | 6.611713999 |
| POLD2 | 13.02048431 |
| POLD3 | 6.984050133 |
| POLD4 | 9.302177843 |
| POLDIP2 | 9.522125498 |
| POLDIP3 | 7.385389879 |
| POLE | 9.317951704 |
| POLE2 | 8.488918014 |
| POLE3 | 12.19863331 |
| POLE4 | 12.4863368 |
| POLG | 10.1022862 |
| POLG2 | 6.510345358 |
| POLH | 5.692448477 |
| POLR1B | 6.520791516 |
| POLR1C | 10.29964787 |
| POLR1D | 10.05585293 |
| POLR1E | 8.483982947 |
| POLR2A | 11.38696522 |
| POLR2B | 11.0333523 |
| POLR2C | 9.853232558 |
| POLR2D | 8.837605117 |
| POLR2E | 10.25029876 |
| POLR2F | 12.92753535 |
| POLR2G | 11.06796556 |
| POLR2H | 12.13575873 |
| POLR2I | 11.42090408 |
| POLR2J | 7.862272997 |
| POLR2J2 | 5.801785711 |
| POLR2J3 | 10.93789078 |
| POLR2K | 7.413053376 |
| POLR2L | 8.235287692 |
| POLR3A | 7.032624896 |
| POLR3B | 10.32741339 |
| POLR3C | 10.00171108 |
| POLR3D | 5.898902053 |
| POLR3E | 8.277456005 |
| POLR3F | 8.020157245 |
| POLR3GL | 11.43276918 |
| POLR3H | 8.708895512 |
| POLR3K | 8.954991252 |
| POLRMT | 8.016023138 |
| POLS | 8.695411396 |
| POM121 | 10.94878971 |
| POMC | 6.01228946 |
| POMGNT1 | 10.53274044 |
| POMP | 13.37801703 |
| POMT1 | 7.655853149 |
| POMT2 | 6.369584909 |
| POMZP3 | 5.722368622 |
| PON1 | 10.40245723 |
| PON2 | 12.75310114 |
| PON3 | 14.09972203 |
| POP1 | 6.262556596 |
| POP2 | 5.860539665 |
| POP4 | 11.31798661 |
| POP5 | 7.5073211 |
| POP7 | 9.312295874 |
| POR | 10.55267176 |
| PORCN | 6.775795787 |
| POSTN | 5.676425103 |
| POT1 | 6.217220682 |
| POU1F1 | 5.548135153 |
| POU2F1 | 5.759514225 |
| POU2F2 | 7.478983195 |
| POU2F3 | 5.569108545 |
| POU3F4 | 5.681599526 |
| POU6F1 | 6.27131348 |
| PPA1 | 13.88985052 |
| PPA2 | 12.14144808 |
| PPAN | 7.970543933 |
| PPAP2A | 9.222907361 |
| PPAP2B | 9.054746155 |
| PPAP2C | 6.297850111 |
| PPAPDC1A | 5.822134359 |
| PPAPDC1B | 10.00787601 |
| PPAPDC2 | 9.319458075 |
| PPAPDC3 | 5.67275781 |
| PPARA | 5.837168615 |
| PPARBP | 9.529619654 |
| PPARD | 6.046869761 |
| PPARG | 10.99008968 |
| PPARGC1A | 9.485949395 |
| PPAT | 8.973145069 |
| PPBP | 8.760647802 |
| PPCS | 11.98734327 |
| PPEF2 | 5.810889912 |
| PPFIA1 | 7.235680926 |
| PPFIA3 | 6.590404334 |
| PPFIA4 | 5.603543789 |
| PPFIBP1 | 6.993056971 |
| PPFIBP2 | 10.39188744 |
| PPGB | 9.301412431 |
| PPHLN1 | 10.48249414 |
| PPIA | 7.72837052 |
| PPIAL4 | 10.80034261 |
| PPIB | 13.93362445 |
| PPIC | 10.92503726 |
| PPID | 9.293751186 |
| PPIE | 8.375879447 |
| PPIF | 6.39899803 |
| PPIG | 8.856709662 |
| PPIH | 9.618539276 |
| PPIL1 | 7.917911128 |
| PPIL2 | 5.758542076 |
| PPIL3 | 11.70055928 |
| PPIL4 | 5.564474554 |
| PPIL5 | 7.970242318 |
| PPL | 6.748250479 |
| PPM1A | 5.840016955 |
| PPM1B | 5.891889023 |
| PPM1D | 8.125566819 |
| PPM1E | 6.819950415 |
| PPM1F | 9.42361799 |
| PPM1G | 7.036708929 |
| PPM1H | 7.405896323 |
| PPM1K | 8.04564184 |
| PPM1M | 8.397107641 |
| PPM2C | 7.461564579 |
| PPME1 | 7.820326652 |
| PPOX | 7.862226594 |
| PPP1CA | 9.889192792 |
| PPP1CB | 10.03679512 |
| PPP1CC | 12.20250009 |
| PPP1R10 | 7.531126178 |
| PPP1R11 | 11.87964487 |
| PPP1R12A | 7.467806508 |
| PPP1R12C | 7.176201708 |
| PPP1R13B | 8.046064181 |
| PPP1R13L | 6.395726725 |
| PPP1R14A | 8.726518659 |
| PPP1R14B | 12.51825862 |
| PPP1R15A | 11.13173339 |
| PPP1R15B | 6.576526865 |
| PPP1R16A | 10.88332587 |
| PPP1R16B | 6.601833487 |
| PPP1R1B | 6.504672223 |
| PPP1R2 | 9.789649662 |
| PPP1R3B | 6.314090822 |
| PPP1R3C | 11.40259704 |
| PPP1R3D | 5.985547795 |
| PPP1R3E | 6.169966251 |
| PPP1R3F | 6.3741296 |
| PPP1R7 | 10.04173546 |
| PPP1R8 | 6.842001658 |
| PPP2CA | 12.57015266 |
| PPP2CB | 9.081741737 |
| PPP2R1A | 11.28000091 |
| PPP2R1B | 9.784194661 |
| PPP2R2A | 10.08662912 |
| PPP2R2B | 5.934386712 |
| PPP2R2D | 7.191037479 |
| PPP2R3A | 6.505195737 |
| PPP2R3B | 5.628738841 |
| PPP2R3C | 9.600521345 |
| PPP2R4 | 11.14778527 |
| PPP2R5A | 10.02330255 |
| PPP2R5B | 5.856232701 |
| PPP2R5C | 7.227165993 |
| PPP2R5D | 6.236698969 |
| PPP2R5E | 10.80216844 |
| PPP3CA | 7.151794916 |
| PPP3CB | 9.449085514 |
| PPP3CC | 8.382984089 |
| PPP3R1 | 10.04953 |
| PPP4C | 11.47401559 |
| PPP4R1 | 10.07842841 |
| PPP4R2 | 8.213011093 |
| PPP4R4 | 6.088579265 |
| PPP5C | 6.423695691 |
| PPP6C | 11.66213195 |
| PPRC1 | 9.360027998 |
| PPT1 | 11.76974173 |
| PPT2 | 6.757916012 |
| PPTC7 | 7.850719876 |
| PPWD1 | 5.915775225 |
| PQBP1 | 8.101280981 |
| PQLC1 | 12.79785652 |
| PQLC3 | 9.909209637 |
| PRAF2 | 7.963393648 |
| PRAME | 5.912935119 |
| PRAMEF10 | 5.506938417 |
| PRAMEF7 | 6.66260978 |
| PRAP1 | 9.915928958 |
| PRC1 | 7.084785036 |
| PRCC | 6.833117336 |
| PRCP | 13.46531159 |
| PRDM1 | 7.084336934 |
| PRDM10 | 6.313977588 |
| PRDM15 | 5.741847802 |
| PRDM2 | 5.65279422 |
| PRDM4 | 9.406935301 |
| PRDM9 | 6.062816001 |
| PRDX1 | 14.87023956 |
| PRDX2 | 12.1681891 |
| PRDX3 | 13.29482199 |
| PRDX4 | 14.38891898 |
| PRDX5 | 12.83857714 |
| PRDX6 | 12.68275482 |
| PREB | 10.56212415 |
| PREI3 | 9.108519037 |
| PRELID1 | 10.23932718 |
| PREP | 8.929249215 |
| PREPL | 8.608842651 |
| PREX1 | 5.792632691 |
| PRF1 | 6.716220221 |
| PRG2 | 7.994285896 |
| PRG-3 | 7.305623293 |
| PRG4 | 13.38677782 |
| PRIC285 | 9.487565485 |
| PRICKLE1 | 6.358214149 |
| PRICKLE2 | 7.804039062 |
| PRIM1 | 8.785708153 |
| PRIM2 | 7.297237515 |
| PRIM2A | 6.132624945 |
| PRKAA1 | 9.635773847 |
| PRKAB1 | 7.463282602 |
| PRKAB2 | 8.048056477 |
| PRKACB | 6.975066008 |
| PRKACG | 5.542369971 |
| PRKAG1 | 6.645780235 |
| PRKAG2 | 9.077638944 |
| PRKAR1A | 12.07385796 |
| PRKAR1B | 6.703310177 |
| PRKAR2A | 7.280677764 |
| PRKCA | 9.20653973 |
| PRKCABP | 5.65830339 |
| PRKCB1 | 7.001343049 |
| PRKCD | 8.527107489 |
| PRKCDBP | 8.818716829 |
| PRKCE | 8.983852136 |
| PRKCH | 8.479431018 |
| PRKCI | 7.267292727 |
| PRKCQ | 5.885709451 |
| PRKCSH | 10.35088828 |
| PRKCZ | 6.452522367 |
| PRKD1 | 5.641787281 |
| PRKD2 | 8.955813658 |
| PRKD3 | 6.438854077 |
| PRKDC | 8.615715339 |
| PRKG1 | 6.414495114 |
| PRKRA | 10.03892019 |
| PRKRIP1 | 7.138762706 |
| PRKRIR | 10.72036705 |
| PRKX | 5.607388351 |
| PRLR | 6.923966942 |
| PRM3 | 5.957734929 |
| PRMT1 | 8.63176482 |
| PRMT2 | 7.642924462 |
| PRMT3 | 7.580943992 |
| PRMT5 | 9.604948521 |
| PRMT6 | 9.381900643 |
| PRMT7 | 9.248751674 |
| PRNP | 12.73974522 |
| PRNPIP | 7.0245617 |
| PRO0132 | 5.880194771 |
| PRO1768 | 6.22609588 |
| PRO1853 | 8.698045266 |
| PROC | 11.11073074 |
| PROCA1 | 6.101077656 |
| PROCR | 7.345250136 |
| PRODH | 5.593131738 |
| PRODH2 | 11.92992613 |
| PROK2 | 8.280837529 |
| PROKR2 | 5.833530839 |
| PROM1 | 6.654676621 |
| PROM2 | 5.879316368 |
| PROS1 | 13.73117 |
| ProSAPiP1 | 5.701933989 |
| PROSC | 11.83812583 |
| PROX1 | 10.8027159 |
| PROZ | 9.01745162 |
| PRP2 | 6.527900303 |
| PRPF18 | 9.162783263 |
| PRPF19 | 11.16195223 |
| PRPF3 | 8.640928048 |
| PRPF31 | 10.24874359 |
| PRPF38A | 7.821892075 |
| PRPF38B | 5.992689829 |
| PRPF39 | 5.701092435 |
| PRPF4 | 9.665279597 |
| PRPF40B | 5.587655849 |
| PRPF4B | 5.860385544 |
| PRPF8 | 10.80438939 |
| PRPS1 | 10.59857354 |
| PRPS1L1 | 6.506602436 |
| PRPS2 | 8.268014875 |
| PRPSAP1 | 11.67202391 |
| PRPSAP2 | 9.290767774 |
| PRR13 | 5.551916327 |
| PRR14 | 10.35306704 |
| PRR16 | 5.52324118 |
| PRR18 | 5.787014757 |
| PRR19 | 5.78608774 |
| PRR3 | 6.829356286 |
| PRR4 | 5.913016336 |
| PRR5 | 9.098220722 |
| PRR6 | 10.80036711 |
| PRR7 | 5.824040873 |
| PRR8 | 5.814727972 |
| PRRC1 | 9.989306167 |
| PRRG1 | 8.515689243 |
| PRRG2 | 6.087693598 |
| PRRG4 | 8.699925831 |
| PRRT2 | 6.103834495 |
| PRRT3 | 6.142939004 |
| PRRX1 | 5.546688635 |
| PRRX2 | 5.974572278 |
| PRSS1 | 6.064558907 |
| PRSS2 | 6.237807289 |
| PRSS23 | 9.285763065 |
| PRSS3 | 7.020510512 |
| PRSS36 | 8.755725135 |
| PRSS8 | 9.951276699 |
| PRTFDC1 | 6.989025218 |
| PRTN3 | 6.055579335 |
| PRUNE | 7.50829855 |
| PRX | 5.563631123 |
| PSAP | 13.65980928 |
| PSARL | 10.4375445 |
| PSAT1 | 11.65703324 |
| PSCA | 7.199582874 |
| PSCD1 | 7.5313696 |
| PSCD2 | 5.998547968 |
| PSCD4 | 8.048854609 |
| PSCDBP | 7.955739621 |
| PSD2 | 5.979861038 |
| PSD3 | 6.353404182 |
| PSD4 | 6.279839917 |
| PSEN1 | 6.107250387 |
| PSEN2 | 8.634581991 |
| PSENEN | 8.530370427 |
| PSF1 | 6.184352147 |
| PSG11 | 5.997774651 |
| PSG9 | 5.86915286 |
| PSIP1 | 8.240471458 |
| PSKH1 | 5.716326824 |
| PSMA1 | 9.324935094 |
| PSMA2 | 10.1259966 |
| PSMA3 | 12.72119386 |
| PSMA4 | 11.61709649 |
| PSMA5 | 13.03120866 |
| PSMA6 | 11.79236431 |
| PSMA7 | 11.80744263 |
| PSMAL | 6.388486148 |
| PSMB1 | 14.42989883 |
| PSMB10 | 12.91260843 |
| PSMB2 | 12.05835283 |
| PSMB3 | 13.28731455 |
| PSMB4 | 13.16024988 |
| PSMB5 | 12.47898293 |
| PSMB6 | 12.59957718 |
| PSMB7 | 13.27649511 |
| PSMB8 | 10.53944764 |
| PSMB9 | 7.052220087 |
| PSMC1 | 13.59753457 |
| PSMC2 | 12.94881625 |
| PSMC3 | 12.40503577 |
| PSMC3IP | 5.828021946 |
| PSMC4 | 9.815876211 |
| PSMC5 | 12.4370772 |
| PSMC6 | 11.7963074 |
| PSMD1 | 10.90772603 |
| PSMD10 | 11.31624631 |
| PSMD11 | 7.565746245 |
| PSMD12 | 13.57646576 |
| PSMD13 | 8.651688275 |
| PSMD14 | 12.08562542 |
| PSMD2 | 10.70114037 |
| PSMD3 | 7.834917397 |
| PSMD4 | 11.82989626 |
| PSMD5 | 9.006035043 |
| PSMD6 | 10.54744508 |
| PSMD7 | 11.42296639 |
| PSMD8 | 10.82681735 |
| PSMD9 | 5.862926113 |
| PSME1 | 13.52816637 |
| PSME2 | 13.43068195 |
| PSME3 | 8.747294577 |
| PSME4 | 11.09084459 |
| PSMF1 | 12.18891202 |
| PSMG1 | 11.31557801 |
| PSMG2 | 11.78225511 |
| PSMG3 | 7.664595545 |
| PSPC1 | 8.013706722 |
| PSPH | 7.122807332 |
| PSRC1 | 5.645373913 |
| PSTK | 7.622246098 |
| PSTPIP1 | 5.653694857 |
| PSTPIP2 | 9.136622426 |
| PTAFR | 5.900498405 |
| PTBP1 | 12.44533123 |
| PTBP2 | 7.364591794 |
| PTCD1 | 7.267019703 |
| PTCD2 | 6.178991679 |
| PTDSS1 | 10.43563579 |
| PTDSS2 | 7.780134736 |
| PTEN | 9.731475532 |
| PTER | 7.284034567 |
| PTGDS | 5.96091897 |
| PTGER1 | 5.97896523 |
| PTGER2 | 5.876162748 |
| PTGER3 | 5.627967701 |
| PTGER4 | 8.59987861 |
| PTGES2 | 7.100973497 |
| PTGES3 | 11.83287258 |
| PTGFR | 6.731192105 |
| PTGFRN | 5.981599784 |
| PTGIR | 5.857125167 |
| PTGIS | 7.003716704 |
| PTGR1 | 13.30960749 |
| PTGR2 | 5.598735754 |
| PTGS1 | 7.640718609 |
| PTGS2 | 6.406560694 |
| PTHR1 | 9.440310464 |
| PTK2 | 8.472551533 |
| PTK2B | 6.01045408 |
| PTK9 | 6.465867454 |
| PTMA | 12.04079012 |
| PTMS | 9.448872341 |
| PTN | 6.415227019 |
| PTOV1 | 10.82538858 |
| PTP4A1 | 9.961194235 |
| PTP4A2 | 7.469417165 |
| PTP4A3 | 5.90923258 |
| PTPLA | 7.763814594 |
| PTPLAD1 | 9.996440178 |
| PTPLAD2 | 9.778067855 |
| PTPLB | 10.88724367 |
| PTPN1 | 10.07283041 |
| PTPN11 | 11.1817444 |
| PTPN12 | 8.97192834 |
| PTPN14 | 5.497418181 |
| PTPN18 | 5.931461504 |
| PTPN2 | 7.972649757 |
| PTPN22 | 5.610543413 |
| PTPN23 | 9.066880479 |
| PTPN3 | 9.714567562 |
| PTPN4 | 8.270068982 |
| PTPN5 | 5.959990298 |
| PTPN6 | 6.833326768 |
| PTPN7 | 5.834821928 |
| PTPN9 | 6.644251045 |
| PTPNS1 | 11.94663763 |
| PTPRA | 6.753831354 |
| PTPRB | 7.624761441 |
| PTPRC | 6.366281935 |
| PTPRCAP | 6.399195033 |
| PTPRD | 5.76428669 |
| PTPRE | 7.362993103 |
| PTPRF | 7.495840449 |
| PTPRG | 8.036122487 |
| PTPRH | 5.94692862 |
| PTPRJ | 6.261034181 |
| PTPRK | 9.744598482 |
| PTPRM | 8.19933391 |
| PTPRO | 6.458849865 |
| PTPRQ | 5.796758608 |
| PTPRR | 5.800433522 |
| PTPRS | 5.678908265 |
| PTPRT | 5.712482172 |
| PTPRU | 6.025462291 |
| PTRF | 10.65045731 |
| PTRH1 | 9.368381193 |
| PTRH2 | 11.94320393 |
| PTS | 11.03550892 |
| PTTG1 | 6.588732562 |
| PTTG1IP | 10.69169347 |
| PTX3 | 6.409812432 |
| PUF60 | 10.97563829 |
| PUM1 | 10.21835393 |
| PUM2 | 9.441404685 |
| PURA | 8.399849853 |
| PURB | 10.17744353 |
| PURG | 8.361275376 |
| PUS1 | 8.349604533 |
| PUS10 | 6.641564495 |
| PUS3 | 10.13627936 |
| PUS7 | 9.831138215 |
| PUS7L | 7.16666981 |
| PUSL1 | 7.074701464 |
| PVALB | 7.27497192 |
| PVR | 8.821756887 |
| PVRL1 | 5.807243973 |
| PVRL2 | 9.293641003 |
| PVRL3 | 10.09486902 |
| PWP1 | 10.32307612 |
| PWP2 | 6.322336277 |
| PWWP2 | 6.836551613 |
| PXK | 5.743896895 |
| PXMP2 | 12.19623767 |
| PXMP3 | 10.72261531 |
| PXMP4 | 6.681234602 |
| PXN | 6.266485711 |
| PYCARD | 9.688971328 |
| PYCR1 | 6.22215679 |
| PYCR2 | 9.610883653 |
| PYCRL | 5.853730535 |
| PYGB | 7.515852723 |
| PYGL | 11.48241854 |
| PYGM | 6.17763773 |
| PYGO2 | 6.273820521 |
| PYROXD1 | 6.289328234 |
| PZP | 9.047393245 |
| QARS | 12.60878674 |
| QDPR | 12.58722197 |
| QKI | 6.732997529 |
| QP-C | 14.72268971 |
| QPCT | 7.626203137 |
| QPCTL | 5.728520679 |
| QPRT | 9.492170278 |
| QRICH1 | 6.263035749 |
| QRSL1 | 6.498778696 |
| QSCN6L1 | 7.186972311 |
| QSOX1 | 5.47192405 |
| QTRT1 | 8.162842601 |
| QTRTD1 | 5.671006887 |
| R3HCC1 | 10.46581024 |
| R3HDM1 | 7.095372583 |
| R3HDM2 | 7.17993083 |
| R3HDML | 5.576517852 |
| RAB10 | 11.81850472 |
| RAB11A | 11.08805244 |
| RAB11B | 5.791662254 |
| RAB11FIP1 | 6.209379669 |
| RAB11FIP2 | 7.722924126 |
| RAB11FIP3 | 9.635452507 |
| RAB11FIP5 | 8.446163604 |
| RAB13 | 10.7947456 |
| RAB14 | 6.019618075 |
| RAB15 | 8.159344623 |
| RAB17 | 8.267254422 |
| RAB18 | 8.447060656 |
| RAB1A | 11.54801641 |
| RAB1B | 6.327339566 |
| RAB20 | 11.65551623 |
| RAB21 | 9.751129176 |
| RAB22A | 9.641551158 |
| RAB23 | 7.158141035 |
| RAB24 | 7.878135258 |
| RAB25 | 5.442333868 |
| RAB26 | 6.251077029 |
| RAB27A | 6.989711406 |
| RAB27B | 5.8089938 |
| RAB28 | 8.130733637 |
| RAB2A | 10.61867629 |
| RAB2B | 10.59839726 |
| RAB30 | 6.252182805 |
| RAB31 | 10.21473937 |
| RAB32 | 10.55728966 |
| RAB33B | 9.79522435 |
| RAB34 | 6.681153106 |
| RAB35 | 9.394108689 |
| RAB37 | 5.529729089 |
| RAB38 | 6.313974275 |
| RAB3B | 6.237785615 |
| RAB3C | 6.03023526 |
| RAB3GAP1 | 10.59109373 |
| RAB3GAP2 | 10.49846184 |
| RAB3IL1 | 9.827274836 |
| RAB3IP | 9.158962088 |
| RAB40B | 8.762260589 |
| RAB41 | 5.96308725 |
| RAB43 | 6.558913529 |
| RAB4A | 8.267228014 |
| RAB4B | 7.353285592 |
| RAB5A | 11.57801116 |
| RAB5B | 11.12980546 |
| RAB5C | 10.0039807 |
| RAB6A | 9.016644904 |
| RAB6IP1 | 9.508019982 |
| RAB7A | 12.02055493 |
| RAB7L1 | 10.72824054 |
| RAB8A | 11.13472965 |
| RAB8B | 8.710155271 |
| RAB9A | 10.87116954 |
| RABAC1 | 12.78874913 |
| RABEP1 | 9.193772497 |
| RABEP2 | 5.811779586 |
| RABEPK | 12.45498947 |
| RABGAP1 | 9.724261832 |
| RABGAP1L | 6.068751917 |
| RABGEF1 | 8.808356547 |
| RABGGTA | 7.13135419 |
| RABGGTB | 11.57423306 |
| RABIF | 6.446520838 |
| RABL2A | 6.013872999 |
| RABL2B | 6.28621959 |
| RABL3 | 9.329336595 |
| RABL4 | 8.578663531 |
| RABL5 | 6.060158425 |
| RAC1 | 7.04347288 |
| RAC2 | 10.25438335 |
| RAC3 | 6.608351525 |
| RACGAP1 | 5.86020624 |
| RAD1 | 6.299194635 |
| RAD17 | 7.890441362 |
| RAD21 | 8.682860544 |
| RAD23A | 10.44982683 |
| RAD23B | 9.942132057 |
| RAD50 | 7.96037049 |
| RAD51AP1 | 7.411810578 |
| RAD51C | 8.629530752 |
| RAD51L1 | 5.649504904 |
| RAD51L3 | 6.451727778 |
| RAD52 | 5.722570826 |
| RAD52B | 6.10266783 |
| RAD54B | 6.024019552 |
| RAD54L2 | 8.832433081 |
| RAD9A | 6.105511988 |
| RAE1 | 8.312468446 |
| RAF1 | 9.769247679 |
| RAG1 | 5.59146995 |
| RAG1AP1 | 10.13536248 |
| RAGE | 7.640971677 |
| RAI1 | 5.996138404 |
| RAI14 | 10.33529796 |
| RAI16 | 6.615400441 |
| RAI2 | 6.003367882 |
| RALA | 10.41964169 |
| RALB | 10.51966802 |
| RALBP1 | 8.814402453 |
| RALGDS | 10.42834212 |
| RALGPS1 | 5.921585181 |
| RALGPS2 | 6.308161215 |
| RALY | 9.197649834 |
| RAMP1 | 8.57498375 |
| RAMP2 | 5.931228677 |
| RAMP3 | 8.717787022 |
| RAN | 9.661217558 |
| RANBP1 | 10.71760215 |
| RANBP10 | 7.248906026 |
| RANBP2 | 5.801347713 |
| RANBP3 | 5.990725572 |
| RANBP3L | 5.742353548 |
| RANBP6 | 8.487878091 |
| RANBP9 | 8.77622236 |
| RANGAP1 | 10.90013696 |
| RANGRF | 8.495467613 |
| RAP1A | 6.139483083 |
| RAP1B | 9.067004189 |
| RAP1GAP | 12.29358052 |
| RAP1GDS1 | 9.265780284 |
| RAP2A | 9.943691748 |
| RAP2B | 5.946170482 |
| RAP2C | 9.995832124 |
| RAPGEF1 | 7.67981574 |
| RAPGEF3 | 6.627595384 |
| RAPGEF4 | 7.05681729 |
| RAPGEF6 | 7.955681083 |
| RAPGEFL1 | 5.833479994 |
| RAPH1 | 7.195067606 |
| RAPSN | 5.919229959 |
| RARA | 8.218051867 |
| RARB | 6.668104306 |
| RARRES1 | 8.963219612 |
| RARRES2 | 13.19091968 |
| RARRES3 | 12.59107658 |
| RARS | 11.88989269 |
| RARS2 | 10.12407855 |
| RASA1 | 9.217020475 |
| RASA2 | 5.285186695 |
| RASA3 | 5.850157767 |
| RASAL2 | 5.712344433 |
| RASD1 | 12.71220442 |
| RASEF | 5.92121691 |
| RASGEF1B | 6.097185912 |
| RASGRF1 | 5.871526397 |
| RASGRP1 | 5.439039697 |
| RASGRP2 | 5.959412202 |
| RASGRP3 | 8.37525371 |
| RASIP1 | 9.957984175 |
| RASL10B | 5.72256393 |
| RASL11A | 6.216016944 |
| RASL11B | 5.809044845 |
| RASSF1 | 6.362851754 |
| RASSF2 | 7.197683289 |
| RASSF4 | 7.108875735 |
| RASSF7 | 10.78866831 |
| RASSF8 | 5.992351114 |
| RAVER1 | 7.760165631 |
| RAVER2 | 6.282170721 |
| RAXL1 | 8.325295405 |
| RB1 | 6.316577281 |
| RB1CC1 | 9.940812144 |
| RBAF600 | 9.760705243 |
| RBAK | 6.267535851 |
| RBBP4 | 5.886865169 |
| RBBP5 | 8.308086057 |
| RBBP6 | 5.77984632 |
| RBBP7 | 10.26510695 |
| RBBP8 | 5.556081877 |
| RBBP9 | 8.678302618 |
| RBED1 | 6.147614716 |
| RBKS | 11.41250113 |
| RBL2 | 9.266466987 |
| RBM10 | 7.975196333 |
| RBM12 | 8.029597515 |
| RBM12B | 7.255272808 |
| RBM14 | 9.432367592 |
| RBM15 | 9.59040828 |
| RBM15B | 7.561776755 |
| RBM16 | 7.548421406 |
| RBM17 | 9.241974473 |
| RBM18 | 8.894946347 |
| RBM19 | 5.530716848 |
| RBM22 | 11.47426799 |
| RBM23 | 10.64861614 |
| RBM25 | 9.343327552 |
| RBM26 | 7.624154712 |
| RBM28 | 7.390134487 |
| RBM3 | 7.512815287 |
| RBM33 | 7.1573883 |
| RBM34 | 9.808201762 |
| RBM35A | 5.566410907 |
| RBM38 | 5.726019743 |
| RBM4 | 7.451540686 |
| RBM41 | 7.151009842 |
| RBM42 | 8.928427683 |
| RBM4B | 8.024846935 |
| RBM5 | 9.893381491 |
| RBM6 | 7.115281275 |
| RBM7 | 9.07540279 |
| RBM9 | 7.982842986 |
| RBMS1 | 5.741094214 |
| RBMS2 | 6.1635273 |
| RBMS3 | 5.469876614 |
| RBMX | 10.94922885 |
| RBMX2 | 6.134130007 |
| RBP1 | 11.46286625 |
| RBP4 | 13.56767205 |
| RBP5 | 8.385907055 |
| RBP7 | 7.728232124 |
| RBPJ | 9.93803432 |
| RBPMS | 5.774448974 |
| RBPMS2 | 10.33423211 |
| RBX1 | 12.64542069 |
| RC3H2 | 8.51965105 |
| RCAN1 | 10.4594495 |
| RCAN3 | 6.238897364 |
| RCBTB1 | 5.935859015 |
| RCC1 | 8.932012723 |
| RCC2 | 10.03119075 |
| RCCD1 | 6.580132394 |
| RCE1 | 7.201003857 |
| RCHY1 | 6.447914807 |
| RCL1 | 10.74130376 |
| RCN1 | 12.48194081 |
| RCN2 | 10.07603224 |
| RCN3 | 7.128620276 |
| RCOR2 | 6.094334844 |
| RCOR3 | 8.180324853 |
| RCP9 | 7.281731748 |
| RCSD1 | 6.522921179 |
| RD3 | 5.988536956 |
| RDBP | 7.445516993 |
| RDH10 | 10.05764994 |
| RDH11 | 12.5022378 |
| RDH12 | 7.091066862 |
| RDH13 | 6.719693964 |
| RDH14 | 9.279179384 |
| RDH16 | 13.07197033 |
| RDH5 | 10.854494 |
| RDH8 | 6.500484492 |
| RDHE2 | 5.643872343 |
| RDX | 10.90511031 |
| REC8L1 | 6.624579954 |
| RECK | 6.008404702 |
| RECQL | 7.205502586 |
| REEP1 | 7.05044507 |
| REEP3 | 6.28823356 |
| REEP4 | 5.805432653 |
| REEP5 | 12.25129156 |
| REEP6 | 11.65947559 |
| REG1A | 6.815909421 |
| REG1B | 6.291095582 |
| REG3A | 5.935024369 |
| REG3G | 5.432009797 |
| REG4 | 5.799950965 |
| REGL | 5.926463337 |
| RELA | 6.21915859 |
| RELB | 6.617779653 |
| RELN | 10.82013254 |
| REN | 6.014866531 |
| RENBP | 8.540019176 |
| RENT1 | 7.856161244 |
| REP15 | 6.409512394 |
| REPIN1 | 9.427919557 |
| REPS1 | 6.404373088 |
| REPS2 | 7.938605893 |
| RER1 | 10.36728456 |
| RERE | 8.340463665 |
| RERG | 5.423398697 |
| RERGL | 7.129162642 |
| REST | 5.694503934 |
| RET | 6.319985196 |
| RETN | 6.516755059 |
| RETNLB | 5.751251627 |
| RETSAT | 9.51654211 |
| REV1L | 8.83944031 |
| REV3L | 6.876060659 |
| REXO1 | 6.280150615 |
| REXO2 | 12.30790044 |
| REXO4 | 9.903023046 |
| RFC1 | 7.656111311 |
| RFC2 | 6.921694186 |
| RFC3 | 6.230724295 |
| RFC4 | 9.390498384 |
| RFC5 | 9.243524806 |
| RFESD | 6.743022508 |
| RFFL | 7.776508452 |
| RFK | 6.288052385 |
| RFNG | 7.144801743 |
| RFP | 8.031018461 |
| RFPL1 | 6.200771473 |
| RFPL2 | 5.735513154 |
| RFPL3 | 5.726663279 |
| RFT1 | 6.343144431 |
| RFTN1 | 10.97286304 |
| RFTN2 | 6.531703347 |
| RFWD2 | 11.14106821 |
| RFWD3 | 8.220531954 |
| RFX1 | 7.460730101 |
| RFX2 | 5.987892693 |
| RFX3 | 9.319685029 |
| RFX4 | 6.069951669 |
| RFX5 | 8.923616732 |
| RFXANK | 7.765103083 |
| RFXAP | 6.407137634 |
| Rg9mtd1 | 7.384763134 |
| RG9MTD2 | 7.39501036 |
| RG9MTD3 | 5.751330865 |
| RGAG1 | 5.526544108 |
| RGAG4 | 6.290243267 |
| RGC32 | 8.334528262 |
| RGL1 | 10.82263507 |
| RGL2 | 8.895744111 |
| RGL4 | 6.207898431 |
| RGMA | 5.42966236 |
| RGMB | 6.406559091 |
| RGN | 9.9626652 |
| RGS1 | 8.801503133 |
| RGS10 | 7.17124612 |
| RGS11 | 5.836925988 |
| RGS12 | 8.252503724 |
| RGS14 | 6.878904359 |
| RGS16 | 8.707641608 |
| RGS18 | 7.488655456 |
| RGS19 | 8.11501466 |
| RGS2 | 9.273854008 |
| RGS3 | 5.898101023 |
| RGS4 | 7.201653541 |
| RGS5 | 7.08854653 |
| RGS7BP | 5.65708909 |
| RHAG | 5.6060845 |
| RHBDD1 | 8.413891956 |
| RHBDD2 | 10.75571546 |
| RHBDD3 | 7.410725834 |
| RHBDF1 | 7.98572438 |
| RHBDF2 | 7.976532079 |
| RHCE | 5.47311701 |
| RHCG | 5.682738259 |
| RHEB | 12.17656101 |
| RHOA | 12.763761 |
| RHOB | 12.12286091 |
| RHOBTB1 | 5.769875322 |
| RHOBTB2 | 5.887095098 |
| RHOBTB3 | 10.31578899 |
| RHOC | 11.60339323 |
| RHOG | 10.21428633 |
| RHOJ | 7.061571626 |
| RHOQ | 11.63796206 |
| RHOT1 | 8.507545008 |
| RHOT2 | 7.540730681 |
| RHOU | 11.70718171 |
| RHPN2 | 11.35718285 |
| RIC8A | 10.08566991 |
| RIC8B | 8.808071899 |
| RICS | 6.475075881 |
| RICTOR | 9.42901935 |
| RIF1 | 7.032705947 |
| RILP | 5.842092916 |
| RILPL1 | 5.515120319 |
| RILPL2 | 8.761670892 |
| RIMS3 | 6.405492436 |
| RIMS4 | 6.531556267 |
| RIN2 | 9.323794452 |
| RIN3 | 5.560871435 |
| RING1 | 12.07136129 |
| RINT1 | 7.88948064 |
| RIOK1 | 7.675238145 |
| RIOK2 | 9.336572041 |
| RIOK3 | 11.02117264 |
| RIPK1 | 9.938112612 |
| RIPK2 | 8.941644011 |
| RIPK4 | 8.461758265 |
| RIPK5 | 7.090819392 |
| RIS1 | 7.764722783 |
| RIT1 | 7.668108479 |
| RKHD1 | 6.129181666 |
| RKHD2 | 8.604741208 |
| RLF | 7.9356507 |
| RMI1 | 7.169038115 |
| RMND1 | 8.163944806 |
| RMND5B | 7.98186154 |
| RN7SK | 6.169601198 |
| RNASE1 | 10.91415705 |
| RNASE10 | 6.443099808 |
| RNASE2 | 7.925673687 |
| RNASE3 | 6.151387733 |
| RNASE4 | 14.41095201 |
| RNASE6 | 6.654502224 |
| RNASEH1 | 9.929140957 |
| RNASEH2A | 7.048262132 |
| RNASEH2B | 9.020610228 |
| RNASEL | 6.645505386 |
| RNASEN | 8.09987652 |
| RNASET2 | 12.07602976 |
| RND1 | 9.030634863 |
| RND2 | 6.786569344 |
| RND3 | 11.4443064 |
| RNF10 | 8.507609198 |
| RNF103 | 10.04191659 |
| RNF11 | 9.01038856 |
| RNF111 | 5.696022689 |
| RNF113A | 9.784401093 |
| RNF113B | 5.750357747 |
| RNF114 | 11.41239664 |
| RNF121 | 7.779138636 |
| RNF122 | 8.324462147 |
| RNF123 | 7.951431946 |
| RNF125 | 6.636311917 |
| RNF126 | 8.496373605 |
| RNF128 | 7.329841207 |
| RNF13 | 8.005135287 |
| RNF130 | 11.000847 |
| RNF135 | 6.467598762 |
| RNF138 | 8.462747143 |
| RNF139 | 5.70337836 |
| RNF14 | 10.67044795 |
| RNF141 | 8.48892862 |
| RNF144 | 7.240941533 |
| RNF144B | 8.765589763 |
| RNF145 | 8.631307563 |
| RNF146 | 6.220157025 |
| RNF148 | 6.393467747 |
| RNF149 | 11.53119086 |
| RNF150 | 5.573844861 |
| RNF151 | 5.824871788 |
| RNF152 | 5.729323562 |
| RNF165 | 6.105177827 |
| RNF166 | 5.42112081 |
| RNF167 | 9.537363691 |
| RNF169 | 6.516190614 |
| RNF170 | 9.320960785 |
| RNF175 | 5.90881753 |
| RNF180 | 7.219197203 |
| RNF181 | 12.58583886 |
| RNF183 | 5.943329411 |
| RNF185 | 7.419242003 |
| RNF186 | 5.813149309 |
| RNF187 | 6.084488152 |
| RNF19A | 9.444651363 |
| RNF19B | 5.824115224 |
| RNF20 | 9.473755303 |
| RNF213 | 5.38042234 |
| RNF215 | 6.250069853 |
| RNF217 | 6.466161408 |
| RNF220 | 6.379780389 |
| RNF24 | 5.732890242 |
| RNF25 | 5.955218964 |
| RNF26 | 7.45335391 |
| RNF31 | 7.554757785 |
| RNF34 | 10.02167171 |
| RNF36 | 5.831073963 |
| RNF38 | 8.553848745 |
| RNF4 | 6.842052967 |
| RNF40 | 7.387374106 |
| RNF41 | 7.918904275 |
| RNF44 | 6.701345829 |
| RNF5P1 | 9.754002414 |
| RNF6 | 7.114944004 |
| RNF7 | 8.957447873 |
| RNF8 | 6.188374833 |
| RNGTT | 9.912267236 |
| RNH1 | 8.958611093 |
| RNMT | 8.281642721 |
| RNMTL1 | 10.62543021 |
| RNPEP | 9.881554215 |
| RNPEPL1 | 6.338330789 |
| RNPS1 | 9.298368028 |
| RNU12 | 5.743324851 |
| RNU39 | 7.018693175 |
| RNU40 | 6.361756 |
| RNUT1 | 8.815485548 |
| RNUXA | 9.980806582 |
| ROBO1 | 5.736520443 |
| ROBO3 | 6.731372276 |
| ROBO4 | 6.619070617 |
| ROCK1 | 6.899291451 |
| ROCK2 | 8.550859271 |
| ROD1 | 10.69473418 |
| ROGDI | 7.172987314 |
| ROM1 | 8.35417776 |
| ROPN1B | 5.763360491 |
| ROPN1L | 6.082144744 |
| RORA | 6.957937474 |
| RORC | 7.455216312 |
| RP11-49G10.8 | 7.38948832 |
| RP11-529I10.4 | 9.139047807 |
| RP2 | 7.968955814 |
| RP9 | 9.62108801 |
| RPA1 | 8.88272875 |
| RPA2 | 9.256269285 |
| RPA3 | 12.34540857 |
| RPAIN | 11.06196488 |
| RPAP1 | 6.372841086 |
| RPAP2 | 7.889380608 |
| RPAP3 | 9.243332075 |
| RPE | 9.305402207 |
| RPESP | 5.736386233 |
| RPGR | 6.704050993 |
| RPH3AL | 7.098718853 |
| RPIA | 8.668297843 |
| RPL10A | 13.13047719 |
| RPL11 | 15.22217956 |
| RPL12 | 14.55969146 |
| RPL13 | 9.347637405 |
| RPL13A | 12.34046971 |
| RPL14 | 11.8002894 |
| RPL14L | 12.64175345 |
| RPL15 | 8.637855502 |
| RPL17 | 9.660575335 |
| RPL18 | 14.22386787 |
| RPL18A | 14.17578115 |
| RPL19 | 14.51769365 |
| RPL22 | 11.44894611 |
| RPL23 | 13.11553378 |
| RPL23A | 9.62529593 |
| RPL23AP13 | 5.695183626 |
| RPL23AP7 | 7.686458439 |
| RPL24 | 14.36827528 |
| RPL26 | 13.7778452 |
| RPL26L1 | 10.1972323 |
| RPL27 | 14.4073688 |
| RPL27A | 10.44076387 |
| RPL28 | 7.245944715 |
| RPL29 | 9.160956283 |
| RPL3 | 14.73109279 |
| RPL30 | 14.71413672 |
| RPL31 | 14.84312888 |
| RPL32 | 6.752324489 |
| RPL34 | 9.997982944 |
| RPL35 | 14.40466496 |
| RPL35A | 14.38071833 |
| RPL36 | 10.54309161 |
| RPL36A | 6.419877135 |
| RPL36AL | 14.41208681 |
| RPL37 | 6.225345326 |
| RPL37A | 9.282819589 |
| RPL38 | 14.96260681 |
| RPL39 | 15.13274114 |
| RPL39L | 8.352311741 |
| RPL4 | 12.81293543 |
| RPL41 | 8.411530356 |
| RPL5 | 14.69864414 |
| RPL6 | 12.45120738 |
| RPL7 | 11.60361133 |
| RPL7A | 12.55440075 |
| RPL7L1 | 10.8820409 |
| RPL8 | 11.65578073 |
| RPL9 | 12.82441484 |
| RPLP0 | 12.80376314 |
| RPLP1 | 12.16072619 |
| RPLP2 | 14.26699155 |
| RPN1 | 13.53614872 |
| RPN2 | 11.9580773 |
| RPP14 | 7.854225012 |
| RPP21 | 11.01212124 |
| RPP25 | 7.175871972 |
| RPP38 | 6.395836646 |
| RPP40 | 11.0808768 |
| RPPH1 | 7.455725038 |
| RPRC1 | 8.007852369 |
| RPRML | 5.759224988 |
| RPS10 | 15.11826594 |
| RPS11 | 14.71302442 |
| RPS12 | 14.87352424 |
| RPS13 | 14.49083758 |
| RPS14 | 14.585151 |
| RPS15 | 8.168298738 |
| RPS15A | 15.02337673 |
| RPS16 | 15.18001267 |
| RPS17 | 13.73164173 |
| RPS18 | 14.45822575 |
| RPS19 | 14.98668522 |
| RPS19BP1 | 10.3057623 |
| RPS2 | 14.74228723 |
| RPS20 | 14.76138503 |
| RPS21 | 11.54447236 |
| RPS23 | 10.5845426 |
| RPS24 | 14.8334871 |
| RPS25 | 15.25855155 |
| RPS26 | 7.02303262 |
| RPS27A | 14.51815283 |
| RPS27L | 13.23435178 |
| RPS28 | 14.35224694 |
| RPS29 | 13.36698522 |
| RPS3 | 14.21577248 |
| RPS3A | 14.28830991 |
| RPS4X | 13.24513138 |
| RPS4Y1 | 12.52290829 |
| RPS5 | 14.13717777 |
| RPS6 | 15.3274647 |
| RPS6KA1 | 7.240183136 |
| RPS6KA2 | 6.880280489 |
| RPS6KA3 | 7.267060344 |
| RPS6KA4 | 8.235309309 |
| RPS6KA5 | 6.745087214 |
| RPS6KB1 | 10.42927359 |
| RPS6KB2 | 7.176806077 |
| RPS6KC1 | 6.629437615 |
| RPS6KL1 | 5.715834907 |
| RPS7 | 10.7141178 |
| RPS8 | 14.6964207 |
| RPS9 | 14.13596948 |
| RPSA | 7.043457887 |
| RPUSD1 | 7.163310095 |
| RPUSD2 | 9.962846846 |
| RPUSD3 | 10.05303623 |
| RPUSD4 | 11.14611754 |
| RQCD1 | 6.438673071 |
| RRAGA | 11.83423723 |
| RRAGB | 7.55636947 |
| RRAGC | 9.403573731 |
| RRAGD | 10.06412434 |
| RRAS | 9.863796874 |
| RRAS2 | 9.73620829 |
| RRBP1 | 9.287628015 |
| RRM1 | 11.33633874 |
| RRM2 | 5.746957629 |
| RRM2B | 8.026645355 |
| RRN3 | 9.524225677 |
| RRP12 | 7.721659717 |
| RRP7A | 9.308591394 |
| RRP9 | 7.111331587 |
| RRS1 | 9.427490408 |
| RSAD1 | 8.638489368 |
| RSAD2 | 5.625045618 |
| RSBN1 | 8.766706764 |
| RSBN1L | 8.10556507 |
| RSC1A1 | 7.07507789 |
| RSF1 | 6.883696439 |
| RSL1D1 | 10.43551145 |
| RSPH3 | 7.444236186 |
| RSPO3 | 7.739144155 |
| RSPRY1 | 11.05900883 |
| RSRC1 | 8.505101918 |
| RSRC2 | 9.849155393 |
| RSU1 | 8.947894177 |
| RTCD1 | 9.314589117 |
| RTEL1 | 5.734663859 |
| RTF1 | 6.403230498 |
| RTKN | 7.905042311 |
| RTL1 | 6.169468587 |
| RTN1 | 5.758056812 |
| RTN2 | 5.883264113 |
| RTN3 | 10.58190279 |
| RTN4 | 12.4276051 |
| RTN4IP1 | 9.512902007 |
| RTN4R | 6.617808038 |
| RTN4RL1 | 6.315323267 |
| RTN4RL2 | 5.845164648 |
| RTP1 | 5.796642671 |
| RTP3 | 11.65816879 |
| RTP4 | 6.856264965 |
| RTTN | 5.704555903 |
| RUFY1 | 6.993509554 |
| RUFY2 | 5.840529636 |
| RUFY3 | 7.277915741 |
| RUFY4 | 6.333071053 |
| RUNDC1 | 8.130428002 |
| RUNDC2B | 7.429216391 |
| RUNDC3A | 5.296701821 |
| RUNDC3B | 9.374399215 |
| RUNX1 | 5.946426307 |
| RUNX3 | 7.01719639 |
| RUSC1 | 8.751339608 |
| RUSC2 | 6.689381757 |
| RUTBC1 | 8.998800611 |
| RUVBL1 | 7.495851054 |
| RUVBL2 | 9.941121616 |
| RW1 | 9.615814177 |
| RWDD1 | 6.44683953 |
| RWDD2A | 9.300809623 |
| RWDD2B | 7.375387403 |
| RWDD3 | 7.457577576 |
| RWDD4A | 9.622016807 |
| RXRA | 12.30592795 |
| RXRB | 9.668325445 |
| RXRG | 6.279758253 |
| RYBP | 10.00619447 |
| RYK | 9.698328072 |
| S100A10 | 12.31410352 |
| S100A11 | 9.408008179 |
| S100A12 | 9.004906723 |
| S100A13 | 7.137780644 |
| S100A14 | 6.809175006 |
| S100A16 | 10.20677708 |
| S100A4 | 11.61347957 |
| S100A6 | 11.25931976 |
| S100A7L1 | 5.769888097 |
| S100A8 | 13.95941226 |
| S100A9 | 10.51489533 |
| S100P | 10.63928458 |
| S100PBP | 5.813934817 |
| S1PR3 | 7.564106217 |
| SAA1 | 14.89066284 |
| SAA2 | 14.34135227 |
| SAA4 | 15.00104709 |
| SAAL1 | 9.283398682 |
| SAC | 8.370752278 |
| SAC3D1 | 9.001079331 |
| SACM1L | 9.914551253 |
| SAE1 | 11.9666931 |
| SAFB | 9.198386806 |
| SAFB2 | 8.658595493 |
| SALL1 | 5.891687739 |
| SAMD11 | 6.915658784 |
| SAMD14 | 6.171565381 |
| SAMD4A | 8.664988078 |
| SAMD4B | 7.013219261 |
| SAMD5 | 7.3824046 |
| SAMD6 | 7.078360017 |
| SAMD9 | 5.753113451 |
| SAMD9L | 6.815652904 |
| SAMHD1 | 5.65712593 |
| SAMM50 | 10.28413568 |
| SAMSN1 | 9.069921088 |
| SAP130 | 8.350288895 |
| SAP18 | 8.189134434 |
| SAP30 | 9.313305463 |
| SAP30BP | 7.326276623 |
| SAP30L | 10.08532777 |
| SAPS1 | 7.245010894 |
| SAPS2 | 8.025499737 |
| SAPS3 | 9.204892682 |
| SAR1A | 10.36007165 |
| SAR1B | 12.71661148 |
| SARDH | 7.362813736 |
| SARM1 | 5.710598248 |
| SARS | 12.92700747 |
| SARS2 | 9.918149188 |
| SART2 | 9.917889041 |
| SASH1 | 8.670240418 |
| SASH3 | 6.532496122 |
| SAT1 | 13.64739881 |
| SAT2 | 11.75084091 |
| SATB2 | 7.116864402 |
| SATL1 | 5.916354174 |
| SAV1 | 8.605126247 |
| SBDS | 10.97223543 |
| SBDSP | 9.836213475 |
| SBEM | 6.32092593 |
| SBF1 | 8.763203747 |
| SBF2 | 5.858549492 |
| SBK1 | 5.970921449 |
| SBNO1 | 5.934550107 |
| SBNO2 | 6.616520284 |
| SC4MOL | 11.32244884 |
| SC5DL | 9.421474868 |
| SC65 | 7.233480562 |
| SCAF1 | 7.355233633 |
| SCAMP1 | 8.672443615 |
| SCAMP2 | 7.242555027 |
| SCAMP3 | 10.08595235 |
| SCAMP4 | 7.358019391 |
| SCAND1 | 9.168191924 |
| SCAND3 | 5.80400339 |
| SCAP | 11.96365755 |
| SCAP1 | 10.11029748 |
| SCAPER | 6.947386667 |
| SCARA3 | 5.792496897 |
| SCARA5 | 5.886969721 |
| SCARB1 | 13.79585404 |
| SCARB2 | 13.14539875 |
| SCARF2 | 5.431764627 |
| SCARNA9 | 8.914387012 |
| SCCPDH | 10.59281013 |
| SCD | 13.58938727 |
| SCFD1 | 11.19230102 |
| SCFD2 | 8.555025329 |
| SCG5 | 7.454567211 |
| SCGB1C1 | 5.906772891 |
| SCGB3A1 | 6.893399263 |
| SCGB3A2 | 5.605648849 |
| SCGN | 6.492062236 |
| SCHIP1 | 7.029738789 |
| SCLT1 | 5.861425412 |
| SCLY | 7.19422278 |
| SCMH1 | 7.072393576 |
| SCML1 | 8.960343843 |
| SCML2 | 6.271831866 |
| SCN1B | 5.713211474 |
| SCN2A2 | 5.69978152 |
| SCN5A | 5.572959229 |
| SCN7A | 5.906586283 |
| SCN8A | 5.900504407 |
| SCN9A | 6.710923729 |
| SCNM1 | 7.855805315 |
| SCNN1A | 8.662300956 |
| SCO1 | 10.48180519 |
| SCO2 | 8.358827647 |
| SCOC | 10.51877695 |
| SCP2 | 7.776432799 |
| SCPEP1 | 10.10422488 |
| SCRG1 | 6.027587246 |
| SCRIB | 9.065301204 |
| SCRN1 | 5.66304669 |
| SCRN2 | 6.867686048 |
| SCRN3 | 5.851335921 |
| SCRT1 | 6.338043217 |
| SCRT2 | 5.861506162 |
| SCT | 5.874289247 |
| SCTR | 6.034305759 |
| SCUBE2 | 5.91334676 |
| SCYL1 | 7.738408368 |
| SCYL1BP1 | 8.639227868 |
| SCYL2 | 10.46397789 |
| SCYL3 | 7.195957425 |
| SDAD1 | 10.9716697 |
| SDC1 | 13.03299547 |
| SDC2 | 9.365695399 |
| SDC3 | 5.693375987 |
| SDC4 | 12.31463604 |
| SDCBP | 10.8572338 |
| SDCBP2 | 7.282794965 |
| SDCCAG1 | 7.79319004 |
| SDCCAG10 | 9.516883706 |
| SDCCAG3 | 10.3041562 |
| SDCCAG33 | 7.80764051 |
| SDCCAG3L | 6.054156651 |
| SDCCAG8 | 6.708931707 |
| SDF2 | 11.21242575 |
| SDF2L1 | 13.79674027 |
| SDF4 | 9.872934279 |
| SDFR1 | 8.271267938 |
| SDHA | 10.16414578 |
| SDHAL2 | 5.800868231 |
| SDHB | 13.58158683 |
| SDHC | 12.23556252 |
| SDHD | 12.5009619 |
| SDK1 | 5.667586565 |
| SDK2 | 5.597200348 |
| SDPR | 9.575983378 |
| SDS | 12.53927546 |
| SDSL | 11.25278301 |
| SEC10L1 | 7.333377561 |
| SEC11A | 12.11033217 |
| SEC11B | 9.425280063 |
| SEC11C | 13.57151424 |
| SEC13 | 10.18090065 |
| SEC14L1 | 10.48309151 |
| SEC14L2 | 8.201915025 |
| SEC14L4 | 10.1749395 |
| SEC22C | 8.251699969 |
| SEC22L1 | 9.496194603 |
| SEC22L2 | 10.09691734 |
| SEC23A | 7.657275018 |
| SEC23B | 10.83515275 |
| SEC23IP | 9.059328785 |
| SEC24B | 6.775048712 |
| SEC24C | 8.351983634 |
| SEC24D | 10.24849118 |
| SEC31A | 7.965875351 |
| SEC61A1 | 10.28544382 |
| SEC61A2 | 7.169935587 |
| SEC61B | 12.3209787 |
| SEC61G | 13.82873679 |
| SEC62 | 9.374785651 |
| SEC63 | 9.594445214 |
| SEC6L1 | 6.662372861 |
| SEC8L1 | 9.772407639 |
| SECISBP2 | 9.080761696 |
| SEH1L | 7.169405003 |
| SEL1L | 8.512066962 |
| SELE | 8.143162742 |
| SELENBP1 | 9.409954794 |
| SELI | 9.51325751 |
| SELK | 5.702698426 |
| SELL | 6.614975638 |
| SELM | 8.956557102 |
| SELO | 8.580404022 |
| SELP | 5.602151146 |
| SELPLG | 8.814282798 |
| SELS | 10.36998721 |
| SELT | 10.12554712 |
| SEMA3C | 5.658299148 |
| SEMA3D | 5.909802761 |
| SEMA3F | 5.641953301 |
| SEMA4A | 6.281462065 |
| SEMA4B | 10.04875179 |
| SEMA4C | 5.790236164 |
| SEMA4D | 7.12577817 |
| SEMA4F | 5.914923779 |
| SEMA4G | 5.827254394 |
| SEMA5A | 6.195235013 |
| SEMA5B | 5.808550868 |
| SEMA6A | 8.964425225 |
| SEMA6B | 6.778104312 |
| SEMA6D | 6.365824818 |
| SENP2 | 7.812553231 |
| SENP3 | 5.911006161 |
| SENP5 | 6.600742442 |
| SENP6 | 8.493945828 |
| SENP7 | 6.090816691 |
| SENP8 | 5.709908847 |
| Sep-15 | 12.91359784 |
| SEPHS1 | 7.806463825 |
| SEPHS2 | 12.48883528 |
| SEPN1 | 8.262459611 |
| SEPP1 | 14.07128732 |
| SEPSECS | 7.459350424 |
| Sep-10 | 8.008282491 |
| Sep-11 | 9.723863038 |
| Sep-12 | 6.4665075 |
| Sep-02 | 8.826138688 |
| Sep-03 | 5.860647394 |
| Sep-04 | 7.846159143 |
| Sep-06 | 5.719273245 |
| Sep-07 | 8.054317424 |
| Sep-08 | 6.060484869 |
| Sep-09 | 13.11885157 |
| SEPW1 | 11.98388173 |
| SEPX1 | 13.24583911 |
| SERAC1 | 6.185723133 |
| SERBP1 | 10.42607676 |
| SERF1A | 10.83715695 |
| SERF1B | 9.855211252 |
| SERF2 | 13.63561361 |
| SERGEF | 8.796345306 |
| SERHL2 | 5.563150981 |
| SERINC1 | 10.51867981 |
| SERINC2 | 9.300180818 |
| SERINC3 | 9.35558819 |
| SERINC4 | 6.186992792 |
| SERP1 | 10.99613923 |
| SERP2 | 8.552436489 |
| SERPINA1 | 10.0596986 |
| SERPINA10 | 14.05066324 |
| SERPINA11 | 12.75264393 |
| SERPINA12 | 6.13838687 |
| SERPINA3 | 15.68303749 |
| SERPINA4 | 6.902185907 |
| SERPINA5 | 12.77476152 |
| SERPINA6 | 13.36640762 |
| SERPINA7 | 13.37725935 |
| SERPINB1 | 11.96614731 |
| SERPINB5 | 5.59449405 |
| SERPINB6 | 11.83952069 |
| SERPINB8 | 8.996862987 |
| SERPINB9 | 5.672607843 |
| SERPINC1 | 15.09031179 |
| SERPIND1 | 11.63544503 |
| SERPINE1 | 9.66514563 |
| SERPINE2 | 9.591753202 |
| SERPINF1 | 12.99914478 |
| SERPINF2 | 13.22697594 |
| SERPING1 | 9.88925854 |
| SERPINH1 | 8.581547563 |
| SERPINI1 | 6.306416587 |
| SERTAD1 | 10.73322056 |
| SERTAD2 | 8.094917254 |
| SERTAD3 | 6.994994284 |
| SERTAD4 | 5.702530167 |
| SESN1 | 8.05192187 |
| SESN2 | 6.52901735 |
| SET | 11.85176095 |
| SETBP1 | 7.272863684 |
| SETD1A | 7.947096479 |
| SETD3 | 9.06881328 |
| SETD4 | 6.971053259 |
| SETD6 | 7.885104463 |
| SETD8 | 7.149386032 |
| SETDB1 | 5.804469005 |
| SETDB2 | 8.915561827 |
| SETMAR | 8.803487892 |
| SETX | 7.728536103 |
| SEZ6L2 | 5.980158442 |
| SF1 | 5.753542287 |
| SF3A1 | 7.695064959 |
| SF3A2 | 8.631575638 |
| SF3A3 | 10.15240962 |
| SF3B1 | 8.931440937 |
| SF3B14 | 12.18950653 |
| SF3B2 | 12.50442045 |
| SF3B3 | 10.04568447 |
| SF3B4 | 10.01670484 |
| SF3B5 | 11.82671228 |
| SF4 | 7.695529034 |
| SFMBT1 | 7.104384534 |
| SFMBT2 | 5.579684784 |
| SFPQ | 9.138876733 |
| SFRP1 | 7.202738327 |
| SFRP4 | 7.62865604 |
| SFRP5 | 5.497938711 |
| SFRS1 | 12.144962 |
| SFRS10 | 11.12861065 |
| SFRS11 | 8.173802642 |
| SFRS12 | 7.874972507 |
| SFRS14 | 7.037734221 |
| SFRS15 | 8.319323477 |
| SFRS16 | 5.888522273 |
| SFRS17A | 7.382240329 |
| SFRS2 | 11.95646545 |
| SFRS3 | 9.67145559 |
| SFRS5 | 11.41134265 |
| SFRS6 | 12.19134285 |
| SFRS7 | 9.57741733 |
| SFRS8 | 7.569761727 |
| SFRS9 | 12.57995248 |
| SFT2D1 | 11.04735828 |
| SFT2D2 | 6.066044148 |
| SFT2D3 | 6.683994206 |
| SFTPA2 | 8.926526048 |
| SFTPD | 5.798416782 |
| SFXN1 | 11.13866701 |
| SFXN2 | 7.818547892 |
| SFXN3 | 8.427482343 |
| SFXN4 | 11.00706294 |
| SFXN5 | 7.665911776 |
| SGCA | 5.669278416 |
| SGCB | 6.469994894 |
| SGCD | 5.509644005 |
| SGCE | 10.17624918 |
| SGK | 12.94748005 |
| SGK2 | 8.559872854 |
| SGK3 | 9.121999304 |
| SGMS1 | 6.311555295 |
| SGOL2 | 5.561054766 |
| SGPL1 | 7.884459164 |
| SGPP1 | 12.49473061 |
| SGPP2 | 5.720146425 |
| SGSH | 7.350475538 |
| SGSM3 | 6.70014998 |
| SGTA | 7.159564102 |
| SH2B1 | 6.692442499 |
| SH2B3 | 9.470822532 |
| SH2D1A | 7.088060353 |
| SH2D2A | 5.946072424 |
| SH2D3C | 6.978825354 |
| SH2D4A | 8.4196426 |
| SH2D5 | 6.060066355 |
| SH3BGR | 6.501362173 |
| SH3BGRL | 10.6610401 |
| SH3BGRL2 | 10.77068545 |
| SH3BGRL3 | 9.389463957 |
| SH3BP1 | 5.956650291 |
| SH3BP4 | 10.16416913 |
| SH3BP5L | 7.225884822 |
| SH3D19 | 9.611109599 |
| SH3GL1 | 8.903905819 |
| SH3GLB1 | 8.770358565 |
| SH3GLB2 | 6.416780243 |
| SH3KBP1 | 9.666363362 |
| SH3MD2 | 7.431653478 |
| SH3MD4 | 5.772241459 |
| SH3PXD2A | 8.623846451 |
| SH3PXD2B | 5.575563037 |
| SH3RF2 | 6.275742249 |
| SH3TC1 | 8.061444798 |
| SH3YL1 | 8.395890711 |
| SHANK2 | 6.867362106 |
| SHANK3 | 7.74695943 |
| SHARPIN | 8.303484077 |
| SHB | 7.974362737 |
| SHBG | 9.189375281 |
| SHC1 | 7.600326039 |
| SHC2 | 6.783070737 |
| SHD | 6.964163476 |
| SHE | 6.376940871 |
| SHFM1 | 11.11282381 |
| SHISA4 | 8.113433377 |
| SHISA5 | 6.615850044 |
| SHKBP1 | 5.45432499 |
| SHMT1 | 11.37532325 |
| SHMT2 | 13.13094455 |
| SHOX2 | 6.052768304 |
| SHQ1 | 7.949517764 |
| SHRM | 7.719259192 |
| SHROOM1 | 7.706919935 |
| SHROOM2 | 6.895077457 |
| SIAE | 10.21020777 |
| SIAH1 | 8.584250356 |
| SIAH2 | 8.162722249 |
| SIDT2 | 11.16304127 |
| SIGIRR | 8.4955386 |
| SIGLEC10 | 7.359844004 |
| SIGLEC11 | 6.434168489 |
| SIGLEC12 | 5.705146638 |
| SIGLEC7 | 6.092742708 |
| SIGLEC9 | 6.421809929 |
| SIKE | 5.757274744 |
| SIL1 | 12.63228607 |
| SILV | 6.450190905 |
| SIN3A | 9.001928133 |
| SIP1 | 8.581107062 |
| SIPA1 | 9.519778766 |
| SIPA1L1 | 9.737517927 |
| SIPA1L2 | 8.847676822 |
| SIPA1L3 | 7.406713827 |
| SIRPB1 | 5.628629859 |
| SIRPB2 | 5.905192223 |
| SIRPD | 6.096824618 |
| SIRT1 | 8.98022161 |
| SIRT2 | 7.156269465 |
| SIRT4 | 6.866890405 |
| SIRT5 | 7.432392229 |
| SIRT7 | 8.101724011 |
| SIT1 | 5.608651428 |
| SIVA | 11.12558513 |
| SIX3 | 6.299874195 |
| SIX5 | 8.787898215 |
| SKAP2 | 8.575021127 |
| SKI | 6.555649023 |
| SKIP | 6.477111407 |
| SKIV2L | 9.355034681 |
| SKIV2L2 | 9.903779072 |
| SKP1A | 11.60792247 |
| SKP2 | 7.237479942 |
| SLA | 9.593008243 |
| SLAMF1 | 5.545033115 |
| SLAMF7 | 6.663246611 |
| SLAMF8 | 5.848903198 |
| SLAMF9 | 7.260838299 |
| SLBP | 8.752273053 |
| SLC10A1 | 10.86039815 |
| SLC10A3 | 5.827178736 |
| SLC10A7 | 8.827104963 |
| SLC11A1 | 6.302028242 |
| SLC11A2 | 11.46926626 |
| SLC12A2 | 6.980921271 |
| SLC12A6 | 5.780038262 |
| SLC12A7 | 7.168267674 |
| SLC12A8 | 7.426073367 |
| SLC12A9 | 9.590473805 |
| SLC13A3 | 5.81435762 |
| SLC13A5 | 13.60862631 |
| SLC14A2 | 6.11450681 |
| SLC15A1 | 7.253280802 |
| SLC15A2 | 5.918290255 |
| SLC15A3 | 8.829623949 |
| SLC15A4 | 8.221454085 |
| SLC16A10 | 10.14641751 |
| SLC16A11 | 5.975686425 |
| SLC16A13 | 5.889786488 |
| SLC16A14 | 6.129408175 |
| SLC16A2 | 8.550027452 |
| SLC16A3 | 8.402540216 |
| SLC16A4 | 5.643606595 |
| SLC16A5 | 7.095978298 |
| SLC16A6 | 5.685077629 |
| SLC16A7 | 6.186977249 |
| SLC16A9 | 6.575537034 |
| SLC17A1 | 8.101980265 |
| SLC17A2 | 13.40991777 |
| SLC17A3 | 9.835303744 |
| SLC17A4 | 7.224522539 |
| SLC17A5 | 12.09431783 |
| SLC17A8 | 5.64032765 |
| SLC18A1 | 5.791237088 |
| SLC19A1 | 7.398306625 |
| SLC19A2 | 8.605215194 |
| SLC19A3 | 9.843854465 |
| SLC1A1 | 9.143241705 |
| SLC1A2 | 7.551011397 |
| SLC1A3 | 7.043845354 |
| SLC1A4 | 8.11474837 |
| SLC1A5 | 6.127427794 |
| SLC1A7 | 5.881672518 |
| SLC20A1 | 9.910551002 |
| SLC20A2 | 7.32764234 |
| SLC22A1 | 10.75035581 |
| SLC22A11 | 5.862481131 |
| SLC22A15 | 6.040963691 |
| SLC22A16 | 5.766780336 |
| SLC22A17 | 7.594820127 |
| SLC22A18 | 10.45926198 |
| SLC22A18AS | 9.420178788 |
| SLC22A3 | 5.779283443 |
| SLC22A4 | 5.955641585 |
| SLC22A5 | 7.294338643 |
| SLC22A7 | 10.52715485 |
| SLC22A9 | 7.927968055 |
| SLC23A1 | 8.526511622 |
| SLC23A2 | 8.691881047 |
| SLC24A1 | 6.479936168 |
| SLC24A3 | 5.899571525 |
| SLC24A6 | 9.701939857 |
| SLC25A1 | 8.485149824 |
| SLC25A10 | 10.25715053 |
| SLC25A11 | 8.871241134 |
| SLC25A12 | 6.275576998 |
| SLC25A13 | 11.00135816 |
| SLC25A14 | 8.199940521 |
| SLC25A15 | 11.3317749 |
| SLC25A16 | 9.128028863 |
| SLC25A17 | 7.69523991 |
| SLC25A18 | 10.29949456 |
| SLC25A19 | 8.902834472 |
| SLC25A20 | 8.911056218 |
| SLC25A22 | 8.041451978 |
| SLC25A23 | 10.77133282 |
| SLC25A24 | 5.659523281 |
| SLC25A25 | 8.585553132 |
| SLC25A26 | 7.336795638 |
| SLC25A28 | 11.83951714 |
| SLC25A29 | 6.027631687 |
| SLC25A3 | 8.475995009 |
| SLC25A30 | 6.634368283 |
| SLC25A31 | 6.068808282 |
| SLC25A32 | 5.881622833 |
| SLC25A34 | 6.042188817 |
| SLC25A37 | 8.153496195 |
| SLC25A38 | 7.961374823 |
| SLC25A39 | 11.53569232 |
| SLC25A4 | 10.80379933 |
| SLC25A40 | 5.594744596 |
| SLC25A42 | 6.389470277 |
| SLC25A43 | 5.768192257 |
| SLC25A44 | 10.7092369 |
| SLC25A46 | 10.48163261 |
| SLC25A5 | 14.49622762 |
| SLC25A6 | 11.44505757 |
| SLC26A1 | 5.558439791 |
| SLC26A10 | 5.676901571 |
| SLC26A11 | 7.466100021 |
| SLC26A2 | 8.586435546 |
| SLC26A3 | 5.83236169 |
| SLC26A6 | 7.772097737 |
| SLC26A9 | 5.639773894 |
| SLC27A1 | 6.801431228 |
| SLC27A2 | 12.80609425 |
| SLC27A3 | 11.36318722 |
| SLC27A4 | 6.700969865 |
| SLC27A5 | 11.66555189 |
| SLC27A6 | 8.847121561 |
| SLC28A1 | 6.835019202 |
| SLC29A1 | 7.525435793 |
| SLC29A2 | 5.775696831 |
| SLC29A3 | 7.724915093 |
| SLC29A4 | 8.538430655 |
| SLC2A10 | 10.24917372 |
| SLC2A11 | 6.020022309 |
| SLC2A12 | 5.686131622 |
| SLC2A13 | 7.120261793 |
| SLC2A14 | 6.018299776 |
| SLC2A2 | 13.18659937 |
| SLC2A3 | 10.56402639 |
| SLC2A4 | 5.765334058 |
| SLC2A4RG | 8.647998083 |
| SLC2A6 | 8.717668628 |
| SLC2A8 | 10.92048689 |
| SLC2A9 | 7.228348197 |
| SLC30A1 | 9.91945887 |
| SLC30A10 | 7.176544053 |
| SLC30A3 | 6.027432654 |
| SLC30A5 | 8.266964899 |
| SLC30A6 | 5.515479067 |
| SLC30A7 | 11.38240782 |
| SLC30A9 | 10.56071696 |
| SLC31A1 | 12.13587816 |
| SLC31A2 | 10.27972664 |
| SLC33A1 | 9.763139878 |
| SLC34A1 | 5.657901992 |
| SLC35A1 | 10.79453682 |
| SLC35A2 | 8.156753725 |
| SLC35A3 | 10.70593313 |
| SLC35A4 | 6.73536843 |
| SLC35A5 | 11.50857539 |
| SLC35B1 | 12.96310689 |
| SLC35B2 | 9.625794577 |
| SLC35B3 | 10.06885812 |
| SLC35B4 | 6.114210436 |
| SLC35C1 | 11.09359581 |
| SLC35C2 | 8.600080165 |
| SLC35D1 | 10.03013939 |
| SLC35D2 | 10.44416866 |
| SLC35E1 | 9.773291536 |
| SLC35E3 | 9.858647943 |
| SLC35F3 | 6.022039635 |
| SLC35F4 | 5.752786293 |
| SLC36A1 | 8.89436134 |
| SLC36A4 | 9.096586437 |
| SLC37A1 | 7.4466354 |
| SLC37A2 | 7.078805962 |
| SLC37A3 | 7.108323162 |
| SLC37A4 | 13.4553965 |
| SLC38A1 | 9.674001347 |
| SLC38A11 | 6.108244909 |
| SLC38A2 | 12.80666123 |
| SLC38A3 | 8.885986422 |
| SLC38A4 | 6.148740617 |
| SLC38A5 | 5.989637426 |
| SLC38A6 | 8.482052609 |
| SLC38A7 | 6.795567501 |
| SLC38A9 | 8.533234802 |
| SLC39A1 | 11.26303719 |
| SLC39A10 | 6.265349303 |
| SLC39A11 | 9.442517498 |
| SLC39A13 | 5.791216879 |
| SLC39A14 | 13.00106616 |
| SLC39A3 | 7.971458178 |
| SLC39A4 | 6.46765728 |
| SLC39A5 | 11.06705416 |
| SLC39A6 | 6.367311678 |
| SLC39A7 | 9.213652714 |
| SLC39A8 | 10.12611687 |
| SLC39A9 | 7.619703852 |
| SLC3A1 | 7.743184124 |
| SLC3A2 | 8.763925426 |
| SLC40A1 | 8.272919314 |
| SLC41A1 | 6.397125767 |
| SLC41A2 | 10.27277012 |
| SLC41A3 | 9.312351944 |
| SLC43A1 | 9.968866766 |
| SLC43A2 | 6.430700466 |
| SLC43A3 | 8.121471809 |
| SLC44A1 | 11.7892612 |
| SLC44A2 | 9.860886374 |
| SLC44A3 | 8.159543869 |
| SLC44A4 | 5.513404397 |
| SLC45A2 | 5.591711911 |
| SLC45A3 | 8.355096081 |
| SLC46A2 | 6.544233085 |
| SLC47A1 | 11.09932272 |
| SLC4A10 | 5.794852268 |
| SLC4A1AP | 7.548374477 |
| SLC4A2 | 8.968065912 |
| SLC4A4 | 8.414181493 |
| SLC5A1 | 6.249713394 |
| SLC5A11 | 6.312232073 |
| SLC5A3 | 5.553274922 |
| SLC5A6 | 11.03179371 |
| SLC5A8 | 6.882655916 |
| SLC6A1 | 9.407485533 |
| SLC6A12 | 9.083573599 |
| SLC6A16 | 7.654136431 |
| SLC6A17 | 5.698806477 |
| SLC6A19 | 5.864053611 |
| SLC6A20 | 5.825563235 |
| SLC6A9 | 7.168715877 |
| SLC7A1 | 8.394418606 |
| SLC7A2 | 12.74832073 |
| SLC7A5 | 8.623063336 |
| SLC7A6 | 5.555008703 |
| SLC7A6OS | 7.037307476 |
| SLC7A7 | 9.757468265 |
| SLC7A8 | 7.14966417 |
| SLC7A9 | 10.92367295 |
| SLC9A1 | 8.303421006 |
| SLC9A2 | 5.566947477 |
| SLC9A3R1 | 12.36045297 |
| SLC9A3R2 | 6.409021013 |
| SLC9A5 | 5.767931106 |
| SLC9A6 | 8.058395327 |
| SLC9A7 | 6.115681651 |
| SLC9A8 | 7.045192559 |
| SLC9A9 | 8.056102792 |
| SLCO1A2 | 5.678507375 |
| SLCO1B1 | 10.67160479 |
| SLCO1B3 | 11.40604692 |
| SLCO2A1 | 7.750850482 |
| SLCO2B1 | 10.43971289 |
| SLCO3A1 | 6.678084958 |
| SLCO4A1 | 8.314439669 |
| SLCO4C1 | 7.576590916 |
| SLFN11 | 8.639255297 |
| SLFN12 | 6.158771414 |
| SLFN13 | 5.599139476 |
| SLFN5 | 5.77828574 |
| SLIT2 | 6.40607289 |
| SLITL2 | 10.72603861 |
| SLITRK2 | 5.911835243 |
| SLITRK3 | 8.181960112 |
| SLITRK4 | 5.529782499 |
| SLK | 8.419972554 |
| SLMAP | 9.824429171 |
| SLPI | 11.17821084 |
| SLTM | 7.936088869 |
| SLU7 | 7.667773466 |
| SMA4 | 7.557023151 |
| SMAD1 | 5.812761923 |
| SMAD2 | 6.713121245 |
| SMAD3 | 7.552101733 |
| SMAD4 | 10.18661464 |
| SMAD5 | 11.25670844 |
| SMAD6 | 8.751620274 |
| SMAD7 | 7.012461987 |
| SMAP1 | 8.982427189 |
| SMAP2 | 8.064998008 |
| SMARCA1 | 8.311478662 |
| SMARCA2 | 10.96951698 |
| SMARCA3 | 7.681683582 |
| SMARCA4 | 10.13668039 |
| SMARCA5 | 7.811520646 |
| SMARCAD1 | 8.183588687 |
| SMARCAL1 | 7.921756236 |
| SMARCB1 | 8.669842913 |
| SMARCC1 | 9.793203635 |
| SMARCC2 | 6.447821501 |
| SMARCD1 | 6.817418189 |
| SMARCD2 | 8.742169907 |
| SMARCD3 | 7.298437008 |
| SMARCE1 | 9.740777898 |
| SMC2L1 | 6.465627651 |
| SMC3 | 8.720960853 |
| SMC4L1 | 9.272302922 |
| SMC6L1 | 7.24767773 |
| SMCP | 6.637810046 |
| SMCR7L | 9.915371318 |
| SMEK2 | 10.38910243 |
| SMG1 | 7.873548386 |
| SMG5 | 8.437047057 |
| SMG6 | 5.778513119 |
| SMG7 | 7.891123083 |
| SMN1 | 6.975570751 |
| SMNDC1 | 9.407649669 |
| SMO | 6.891049833 |
| SMOC1 | 6.032483283 |
| SMOC2 | 5.774141846 |
| SMOX | 7.512853234 |
| SMPD1 | 8.324720936 |
| SMPD2 | 6.89629655 |
| SMPD3 | 5.793743903 |
| SMPDL3A | 11.80089195 |
| SMPDL3B | 5.661598959 |
| SMS | 10.40598765 |
| SMTN | 5.764305347 |
| SMU1 | 8.458125079 |
| SMUG1 | 10.69788338 |
| SMURF1 | 5.939974325 |
| SMURF2 | 5.817213583 |
| SMYD2 | 8.417510544 |
| SMYD3 | 8.724681212 |
| SNAI1 | 6.498296528 |
| SNAI2 | 6.310299279 |
| SNAI3 | 6.04010132 |
| SNAP23 | 8.132794971 |
| SNAP25 | 6.225139141 |
| SNAP29 | 8.588888515 |
| SNAPC2 | 7.732758525 |
| SNAPC3 | 8.997832437 |
| SNAPC4 | 8.433591677 |
| SNAPC5 | 8.23759425 |
| SNAPIN | 9.098116673 |
| SNCA | 7.094424231 |
| SNCAIP | 5.883178185 |
| SNCB | 8.041725352 |
| SND1 | 12.56145255 |
| SNF1LK | 9.260193555 |
| SNF1LK2 | 5.740919146 |
| SNF8 | 10.90105609 |
| SNIP | 6.101835023 |
| SNIP1 | 9.017876429 |
| SNN | 7.400816001 |
| SNORA10 | 6.418427537 |
| SNORA33 | 7.27025194 |
| SNORA62 | 7.919248219 |
| SNORA64 | 6.268772449 |
| SNORA65 | 6.430587207 |
| SNORA70 | 6.430117317 |
| SNORD100 | 5.85434033 |
| SNORD14A | 5.623936551 |
| SNORD15B | 5.592134978 |
| SNORD16 | 7.242950467 |
| SNORD21 | 6.131874063 |
| SNORD25 | 5.569176578 |
| SNORD31 | 6.171624115 |
| SNORD32A | 7.295455931 |
| SNORD33 | 6.411241381 |
| SNORD34 | 5.509045747 |
| SNORD35A | 6.140125239 |
| SNORD35B | 6.80300986 |
| SNORD38A | 5.564173677 |
| SNORD43 | 6.836278651 |
| SNORD4A | 5.616754613 |
| SNORD68 | 7.755339285 |
| SNORD73A | 6.017063116 |
| SNPH | 6.442530745 |
| SNRK | 9.358197194 |
| SNRP70 | 11.00379707 |
| SNRPA | 8.561645146 |
| SNRPA1 | 11.28181694 |
| SNRPB | 11.81085064 |
| SNRPB2 | 11.19450672 |
| SNRPC | 9.987139516 |
| SNRPD1 | 8.048645768 |
| SNRPD2 | 8.986775172 |
| SNRPD3 | 8.613968038 |
| SNRPE | 5.912425944 |
| SNRPF | 12.60359977 |
| SNRPG | 13.98799403 |
| SNRPN | 9.392342431 |
| SNTA1 | 7.372750454 |
| SNTB1 | 11.00255806 |
| SNTB2 | 6.325847142 |
| SNTG1 | 5.69928322 |
| SNURF | 10.2779099 |
| SNW1 | 9.863851066 |
| SNX1 | 6.25581535 |
| SNX10 | 12.17349112 |
| SNX11 | 9.46880919 |
| SNX12 | 6.088848431 |
| SNX13 | 8.435227083 |
| SNX14 | 8.283591213 |
| SNX15 | 9.359042748 |
| SNX16 | 7.565987051 |
| SNX17 | 10.86047996 |
| SNX19 | 10.51402067 |
| SNX2 | 11.13610501 |
| SNX21 | 6.295487395 |
| SNX22 | 5.668550591 |
| SNX24 | 6.577422335 |
| SNX25 | 7.121944446 |
| SNX26 | 7.26579519 |
| SNX27 | 10.102316 |
| SNX3 | 13.46656199 |
| SNX30 | 7.605829562 |
| SNX32 | 5.621496034 |
| SNX33 | 7.419388688 |
| SNX4 | 10.83417296 |
| SNX5 | 9.257035762 |
| SNX6 | 7.792792939 |
| SNX7 | 7.712599791 |
| SNX8 | 8.346619458 |
| SNX9 | 5.70716029 |
| SOAT1 | 7.314399854 |
| SOAT2 | 6.106629466 |
| SOCS1 | 8.502786995 |
| SOCS2 | 9.074224301 |
| SOCS3 | 7.965272929 |
| SOCS4 | 7.217629411 |
| SOCS5 | 6.182090669 |
| SOCS6 | 6.777707243 |
| SOCS7 | 5.910127589 |
| SOD1 | 15.28830943 |
| SOD2 | 10.58085681 |
| SOD3 | 5.328485562 |
| SON | 6.037198432 |
| SORBS1 | 7.284105523 |
| SORBS2 | 8.15975835 |
| SORBS3 | 7.051613764 |
| SORCS1 | 6.128568015 |
| SORCS2 | 6.94299896 |
| SORD | 10.69817394 |
| SORL1 | 8.627597792 |
| SORT1 | 6.493221765 |
| SOS1 | 5.55347626 |
| SOS2 | 5.987062389 |
| SOX13 | 8.343831541 |
| SOX17 | 5.970520242 |
| SOX18 | 10.40700864 |
| SOX2 | 6.49603532 |
| SOX30 | 6.841275189 |
| SOX4 | 6.828014158 |
| SOX6 | 5.572711331 |
| SOX7 | 6.611017459 |
| SOX9 | 6.375971774 |
| SP1 | 8.030085824 |
| SP110 | 7.610749516 |
| SP140 | 5.85590416 |
| SP2 | 7.19364408 |
| SP3 | 7.237027675 |
| SP4 | 6.429775903 |
| SPA17 | 8.518557941 |
| SPACA1 | 5.739888538 |
| SPAG1 | 5.492313889 |
| SPAG16 | 8.761068096 |
| SPAG4 | 6.299804328 |
| SPAG5 | 8.434752978 |
| SPAG7 | 9.536594743 |
| SPAG8 | 5.689333093 |
| SPAG9 | 7.226938455 |
| SPARC | 10.2078671 |
| SPARCL1 | 8.284408663 |
| SPAST | 8.354265612 |
| SPATA1 | 10.06238105 |
| SPATA13 | 7.139505796 |
| SPATA18 | 5.6847403 |
| SPATA2 | 6.012783205 |
| SPATA20 | 9.275170092 |
| SPATA2L | 8.764350989 |
| SPATA5L1 | 8.637465291 |
| SPATA7 | 7.969305478 |
| SPATC1 | 5.807356511 |
| SPATS2 | 6.211837365 |
| SPC25 | 5.757679052 |
| SPCS1 | 13.11694311 |
| SPCS2 | 13.55221358 |
| SPCS3 | 8.293850621 |
| SPDEF | 6.091270874 |
| SPECC1L | 10.57469429 |
| SPEN | 9.03262373 |
| SPESP1 | 6.216221509 |
| SPFH2 | 10.25327876 |
| SPG20 | 7.165187606 |
| SPG21 | 7.880546167 |
| SPG3A | 5.487378899 |
| SPG7 | 8.357099795 |
| SPHAR | 7.064595496 |
| SPHK2 | 9.600350721 |
| SPI1 | 8.593444937 |
| SPIC | 6.567032871 |
| SPIN | 7.308721525 |
| SPIN3 | 7.176112838 |
| SPINK1 | 14.89588012 |
| SPINLW1 | 5.816654799 |
| SPINT2 | 8.0485721 |
| SPIRE1 | 8.947950829 |
| SPIRE2 | 6.137964856 |
| SPN | 7.342547431 |
| SPNS1 | 9.816091067 |
| SPOCK2 | 7.81089563 |
| SPON1 | 7.203662518 |
| SPON2 | 6.549520959 |
| SPOPL | 7.608908351 |
| SPP1 | 10.68317825 |
| SPP2 | 11.95900042 |
| SPPL2A | 12.36686442 |
| SPPL3 | 5.838288184 |
| SPR | 10.78942473 |
| SPRED1 | 10.52648539 |
| SPRED2 | 8.274490262 |
| SPRR1A | 8.43409963 |
| SPRR2D | 5.885719541 |
| SPRR2F | 5.953336929 |
| SPRY1 | 7.867448158 |
| SPRY2 | 9.461717311 |
| SPRY4 | 5.650907891 |
| SPRYD3 | 9.896813122 |
| SPRYD4 | 10.8200663 |
| SPSB1 | 9.135070665 |
| SPSB2 | 7.155115949 |
| SPSB3 | 10.24566544 |
| SPTA1 | 5.539623009 |
| SPTAN1 | 9.314406085 |
| SPTBN1 | 7.769762718 |
| SPTBN2 | 6.207968098 |
| SPTBN5 | 5.659110279 |
| SPTLC1 | 8.419766215 |
| SPTLC2 | 6.29955808 |
| SPTY2D1 | 8.243820489 |
| SQLE | 7.437586299 |
| SQRDL | 11.11370992 |
| SQSTM1 | 11.45275659 |
| SRBD1 | 9.643902604 |
| SRC | 7.076274119 |
| SRCAP | 6.176520649 |
| SRCRB4D | 7.489713479 |
| SRD5A1 | 7.818891432 |
| SRD5A2 | 10.07428239 |
| SRD5A2L | 6.064119292 |
| SREBF1 | 10.7319044 |
| SREBF2 | 8.754605611 |
| SRF | 10.87948098 |
| SRFBP1 | 7.269247277 |
| SRGAP1 | 6.058956714 |
| SRGAP2 | 5.647908856 |
| SRGAP3 | 5.62534411 |
| SRGN | 13.1364374 |
| SRI | 7.750646291 |
| SRM | 8.783715415 |
| SRP14 | 14.25201725 |
| SRP19 | 11.3030899 |
| SRP46 | 10.19017914 |
| SRP54 | 9.380179942 |
| SRP68 | 10.51203448 |
| SRP72 | 10.88043305 |
| SRP9 | 9.124117474 |
| SRPK1 | 9.597804421 |
| SRPK2 | 6.254752724 |
| SRPR | 10.49443337 |
| SRPRB | 13.22573269 |
| SRPX | 9.514689272 |
| SRPX2 | 6.738899054 |
| SRR | 6.108215495 |
| SRRM1 | 11.09494038 |
| SRRM1L | 7.410219808 |
| SRRM2 | 9.719212005 |
| SRrp35 | 6.132832131 |
| SRXN1 | 9.807958039 |
| SS18 | 9.443174591 |
| SS18L1 | 7.780162555 |
| SS18L2 | 12.54616077 |
| SSB | 11.068035 |
| SSBP1 | 12.75744123 |
| SSBP2 | 9.09258239 |
| SSBP3 | 6.645615942 |
| SSBP4 | 5.530743027 |
| SSFA2 | 7.537151135 |
| SSH1 | 5.92950724 |
| SSH2 | 7.743629939 |
| SSH3 | 8.171741073 |
| SSNA1 | 9.127291845 |
| SSPN | 5.715652292 |
| SSR1 | 11.61303471 |
| SSR2 | 9.276923893 |
| SSR3 | 8.125180281 |
| SSR4 | 13.96552827 |
| SSRP1 | 10.58656007 |
| SSSCA1 | 8.85142266 |
| SSTR1 | 6.021783558 |
| SSTR2 | 8.753073474 |
| SSU72 | 12.07182447 |
| SSX2IP | 6.493679046 |
| SSX4 | 5.775322423 |
| SSX8 | 6.024933343 |
| ST13 | 11.43598846 |
| ST14 | 5.619397993 |
| ST3GAL1 | 7.88207971 |
| ST3GAL2 | 8.200349931 |
| ST3GAL3 | 6.893765625 |
| ST3GAL4 | 9.81970328 |
| ST3GAL5 | 9.118848865 |
| ST3GAL6 | 10.89359491 |
| ST5 | 6.463696244 |
| ST6GAL1 | 13.48250355 |
| ST6GALNAC2 | 5.789543589 |
| ST6GALNAC4 | 7.489489892 |
| ST6GALNAC6 | 9.334289306 |
| ST7 | 9.357720411 |
| ST7L | 6.633162933 |
| ST8SIA4 | 5.716967223 |
| STAB1 | 9.811814797 |
| STAB2 | 8.49091024 |
| STAC | 6.052881199 |
| STAC3 | 5.972469753 |
| STAG1 | 7.858915545 |
| STAG2 | 9.580918153 |
| STAG3 | 8.178770394 |
| STAG3L1 | 6.083045379 |
| STAM | 8.343414934 |
| STAM2 | 7.648023642 |
| STAMBP | 10.69025816 |
| STAMBPL1 | 8.234904132 |
| STAP2 | 7.596036048 |
| STARD10 | 11.55679949 |
| STARD13 | 6.34195469 |
| STARD3 | 8.043013448 |
| STARD3NL | 9.380736406 |
| STARD5 | 9.319357692 |
| STARD7 | 9.371718393 |
| STARD8 | 8.852736547 |
| STAT1 | 11.21042131 |
| STAT2 | 11.22782459 |
| STAT3 | 9.865080327 |
| STAT4 | 7.524693287 |
| STAT5A | 8.34444154 |
| STAT5B | 9.001588025 |
| STAT6 | 8.845019561 |
| STAU | 8.090133648 |
| STAU2 | 8.145704613 |
| STC1 | 6.290848004 |
| STEAP1 | 9.961559793 |
| STEAP2 | 6.961099902 |
| STEAP3 | 8.734165353 |
| STEAP4 | 6.792016937 |
| STGC3 | 5.808457661 |
| STIL | 7.124403898 |
| STIM1 | 9.145034013 |
| STIM2 | 6.662054906 |
| STIP1 | 9.777747022 |
| STK10 | 6.231982279 |
| STK11 | 7.476657471 |
| STK11IP | 6.228657542 |
| STK16 | 7.143338636 |
| STK17B | 6.69609744 |
| STK19 | 9.082263776 |
| STK24 | 8.342905709 |
| STK25 | 9.439759357 |
| STK3 | 10.15525461 |
| STK32B | 7.463261962 |
| STK35 | 9.183412261 |
| STK36 | 8.017001478 |
| STK38 | 11.18911635 |
| STK38L | 5.604775795 |
| STK39 | 6.283361293 |
| STK4 | 10.41482295 |
| STK40 | 6.173487591 |
| STMN1 | 6.25094929 |
| STMN3 | 8.166260262 |
| STOM | 11.16295228 |
| STOML1 | 8.081944084 |
| STOML2 | 12.59749493 |
| STOX2 | 8.048611139 |
| STRA13 | 12.09457478 |
| STRADA | 9.122782667 |
| STRAP | 11.8494929 |
| STRBP | 7.505363106 |
| STRN | 8.63421201 |
| STRN3 | 10.23581284 |
| STRN4 | 6.266318807 |
| STS | 5.669735094 |
| STS-1 | 7.90308256 |
| STT3A | 11.17371409 |
| STT3B | 9.318524467 |
| STUB1 | 9.766708032 |
| STX10 | 8.834616673 |
| STX11 | 8.45223339 |
| STX12 | 8.932191141 |
| STX16 | 10.07810874 |
| STX17 | 7.099046333 |
| STX18 | 6.195394548 |
| STX1A | 5.70945191 |
| STX2 | 8.757446064 |
| STX3 | 8.172290618 |
| STX5A | 8.74213275 |
| STX6 | 6.960627202 |
| STX7 | 7.891324852 |
| STX8 | 10.14929083 |
| STXBP2 | 9.063438885 |
| STXBP3 | 9.205307215 |
| STXBP4 | 5.635661797 |
| STXBP5 | 7.161479422 |
| STXBP6 | 7.983948439 |
| STYX | 5.746311171 |
| STYXL1 | 8.126550659 |
| SUB1 | 10.06720587 |
| SUCLA2 | 8.569262177 |
| SUCLG1 | 12.37503973 |
| SUCLG2 | 12.65804739 |
| SUCNR1 | 6.620930424 |
| SUDS3 | 7.477007103 |
| SUFU | 6.095704625 |
| SUGT1 | 11.85454845 |
| SULF1 | 5.966562631 |
| SULF2 | 7.386212567 |
| SULT1A1 | 6.331731773 |
| SULT1A2 | 7.881391135 |
| SULT1A3 | 7.996279084 |
| SULT1A4 | 7.67238974 |
| SULT1B1 | 7.100017512 |
| SULT1C2 | 5.736163602 |
| SULT1C4 | 6.092814372 |
| SULT1E1 | 6.320479389 |
| SULT2A1 | 11.94160285 |
| SULT2B1 | 5.556788512 |
| SULT4A1 | 5.481633465 |
| SULT6B1 | 5.782932277 |
| SUMF1 | 10.03043121 |
| SUMF2 | 8.940294325 |
| SUMO1P3 | 10.82422637 |
| SUMO2 | 12.24438553 |
| SUMO3 | 12.2162865 |
| SUMO4 | 5.789592797 |
| SUOX | 11.13280218 |
| SUPT16H | 9.824515236 |
| SUPT3H | 6.294776686 |
| SUPT4H1 | 8.916665448 |
| SUPT5H | 8.815739143 |
| SUPT6H | 8.598050385 |
| SUPV3L1 | 8.545029363 |
| SURB7 | 6.419627959 |
| SURF1 | 10.61860141 |
| SURF2 | 7.929812806 |
| SURF4 | 12.2954277 |
| SURF5 | 9.378795962 |
| SURF6 | 6.089820416 |
| SUSD1 | 8.077352604 |
| SUSD2 | 5.861049696 |
| SUSD3 | 8.182478283 |
| SUSD4 | 7.404054845 |
| SUV39H1 | 8.575435837 |
| SUV39H2 | 6.617036167 |
| SUV420H1 | 7.586229974 |
| SUV420H2 | 6.591211738 |
| SUZ12 | 11.85410211 |
| SV2B | 5.979812716 |
| SVIL | 7.873737615 |
| SWAP70 | 8.852248844 |
| SYAP1 | 9.340872582 |
| SYDE1 | 5.778409339 |
| SYDE2 | 6.039819868 |
| SYF2 | 10.12197426 |
| SYK | 6.69126951 |
| SYMPK | 6.950469504 |
| SYN1 | 5.800249396 |
| SYN2 | 5.73926043 |
| SYNC1 | 5.919346276 |
| SYNCRIP | 10.83577616 |
| SYNE2 | 6.587431035 |
| SYNGR1 | 5.872046322 |
| SYNJ1 | 7.863243796 |
| SYNJ2 | 7.383511079 |
| SYNJ2BP | 10.91026863 |
| SYNPO | 5.736952378 |
| SYNPO2 | 6.589271984 |
| SYPL1 | 9.992929147 |
| SYPL2 | 5.82048155 |
| SYS1 | 9.116136917 |
| SYT11 | 6.175435062 |
| SYT12 | 6.301091695 |
| SYT13 | 5.672402027 |
| SYT15 | 6.497893118 |
| SYT16 | 5.759405617 |
| SYT17 | 8.182133965 |
| SYT3 | 6.828017534 |
| SYT7 | 6.000587898 |
| SYT8 | 6.470390751 |
| SYT9 | 5.743153659 |
| SYTL2 | 6.742280259 |
| SYTL4 | 8.965466689 |
| SYVN1 | 12.08057825 |
| TAAR2 | 8.708214107 |
| TAAR5 | 5.369402053 |
| TAAR6 | 5.496996404 |
| TAB3 | 5.513524555 |
| TACC1 | 11.46789331 |
| TACC2 | 9.346825187 |
| TACC3 | 6.463288742 |
| TACR1 | 5.826981577 |
| TACSTD1 | 7.184123708 |
| TADA1L | 6.840048835 |
| TADA2L | 7.632544003 |
| TADA3L | 8.237203431 |
| TAF10 | 10.61291646 |
| TAF12 | 8.316078639 |
| TAF13 | 7.056434775 |
| TAF15 | 11.48545402 |
| TAF1A | 6.189337271 |
| TAF1B | 8.139821468 |
| TAF1C | 8.50870725 |
| TAF1L | 5.479866619 |
| TAF2 | 8.3219345 |
| TAF4 | 8.211166691 |
| TAF5 | 7.074388031 |
| TAF5L | 8.775102948 |
| TAF6 | 5.769351498 |
| TAF6L | 9.07616798 |
| TAF7 | 8.996556375 |
| TAF9 | 8.426609749 |
| TAF9L | 7.560953915 |
| TAGLN | 12.24156829 |
| TAGLN2 | 10.50773013 |
| TAL1 | 5.726600032 |
| TALDO1 | 12.41125246 |
| TANC1 | 8.2940339 |
| TANK | 10.57754767 |
| TAOK1 | 7.624743735 |
| TAOK2 | 6.766448843 |
| TAP1 | 11.09877748 |
| TAP2 | 6.739012157 |
| TAPBP | 8.43379865 |
| TAPBPL | 9.146321517 |
| TAPT1 | 8.158258975 |
| TARBP1 | 6.361315149 |
| TARBP2 | 8.792476992 |
| TARDBP | 6.493677014 |
| TARP | 6.340315734 |
| TARS | 11.73022499 |
| TARS2 | 7.403274526 |
| TARSL2 | 6.175134205 |
| TAS2R10 | 6.00139593 |
| TAS2R3 | 5.951997562 |
| TAS2R4 | 5.852583707 |
| TAS2R5 | 5.571177425 |
| TASP1 | 6.828548275 |
| TAT | 12.65784336 |
| TATDN1 | 9.541447674 |
| TATDN2 | 7.833301547 |
| TATDN3 | 8.030281551 |
| TAX1BP1 | 12.08101888 |
| TAX1BP3 | 9.836366624 |
| TAZ | 5.838249856 |
| TBC1D10A | 6.556377058 |
| TBC1D10B | 6.038338826 |
| TBC1D10C | 6.283353164 |
| TBC1D13 | 7.440796588 |
| TBC1D14 | 8.227743448 |
| TBC1D15 | 10.94505944 |
| TBC1D16 | 9.561802727 |
| TBC1D17 | 5.615459894 |
| TBC1D19 | 5.916862247 |
| TBC1D2 | 7.760738798 |
| TBC1D20 | 5.68547235 |
| TBC1D22A | 10.1119377 |
| TBC1D22B | 7.726892145 |
| TBC1D23 | 7.886483166 |
| TBC1D2B | 7.126040041 |
| TBC1D4 | 7.598545795 |
| TBC1D5 | 5.676530513 |
| TBC1D7 | 9.34284803 |
| TBC1D8 | 6.961917367 |
| TBC1D8B | 6.553976548 |
| TBC1D9 | 8.54169878 |
| TBC1D9B | 8.323197805 |
| TBCA | 13.915488 |
| TBCB | 10.53498343 |
| TBCC | 8.006239193 |
| TBCCD1 | 7.372935862 |
| TBCD | 7.145155347 |
| TBCE | 10.50073846 |
| TBCEL | 5.907218057 |
| TBK1 | 10.05168717 |
| TBL1X | 9.983328679 |
| TBL1XR1 | 7.164159272 |
| TBL2 | 8.966647198 |
| TBL3 | 7.100646614 |
| TBP | 7.87908591 |
| TBPL1 | 10.19739801 |
| TBRG4 | 7.643584954 |
| TBX1 | 7.124195829 |
| TBX10 | 7.82086149 |
| TBX15 | 8.48135763 |
| TBX2 | 6.981357239 |
| TBX21 | 6.314147713 |
| TBX22 | 6.176750592 |
| TBX3 | 7.050898347 |
| TBX5 | 5.705257735 |
| TBX6 | 5.782577639 |
| TBXA2R | 5.926933022 |
| TBXAS1 | 9.343454096 |
| TCEA1 | 6.120056043 |
| TCEA2 | 7.242967415 |
| TCEA3 | 12.9754974 |
| TCEAL1 | 9.093113214 |
| TCEAL3 | 8.730845183 |
| TCEAL4 | 9.176570651 |
| TCEAL5 | 6.052062944 |
| TCEAL7 | 5.713330574 |
| TCEAL8 | 9.673523263 |
| TCEB1 | 10.87870967 |
| TCEB2 | 13.11060127 |
| TCERG1 | 7.98360079 |
| TCF1 | 6.397250843 |
| TCF12 | 8.547377349 |
| TCF2 | 6.857567588 |
| TCF20 | 6.104886263 |
| TCF21 | 6.071546476 |
| TCF25 | 11.75572967 |
| TCF3 | 5.648148687 |
| TCF4 | 7.968205001 |
| TCF7L2 | 6.812011448 |
| TCFL5 | 8.416436637 |
| TCHP | 7.521641031 |
| TCIRG1 | 7.166631507 |
| TCN1 | 6.613164662 |
| TCN2 | 7.607664522 |
| TCP1 | 9.697704938 |
| TCP10L | 8.343348559 |
| TCP11L1 | 7.21406752 |
| TCP11L2 | 5.717006351 |
| TCTA | 8.608835415 |
| TCTEX1D1 | 6.140106125 |
| TCTEX1D2 | 7.156937394 |
| TDG | 10.0445412 |
| TDO2 | 13.51414418 |
| TDP1 | 6.488939266 |
| TDRD1 | 9.051816095 |
| TDRD10 | 5.527975632 |
| TDRD3 | 6.744910037 |
| TDRD6 | 5.977669927 |
| TDRD7 | 8.427830529 |
| TDRKH | 5.557568786 |
| TEAD2 | 10.18414046 |
| TEAD3 | 6.679738849 |
| TEAD4 | 8.673622175 |
| TEC | 5.617406647 |
| TEF | 8.443986351 |
| TEGT | 14.16351231 |
| TEK | 9.060555346 |
| TEKT1 | 5.886302101 |
| TELO2 | 6.307963402 |
| TENC1 | 5.869548337 |
| TERC | 6.757259933 |
| TERF1 | 7.692603305 |
| TERF2 | 8.037870244 |
| TERF2IP | 9.56570841 |
| TES | 7.403502906 |
| TESC | 7.710818469 |
| TESK1 | 6.465949514 |
| TESK2 | 7.560146426 |
| TESSP2 | 5.84921318 |
| TESSP5 | 6.574198037 |
| TETRAN | 10.07846026 |
| TEX10 | 7.895874127 |
| TEX2 | 10.76964478 |
| TEX261 | 10.61670712 |
| TEX264 | 9.171870768 |
| TF | 15.15420032 |
| TFAM | 10.49125072 |
| TFAP4 | 5.494935403 |
| TFB1M | 8.342040678 |
| TFB2M | 10.88176858 |
| TFCP2 | 7.849300434 |
| TFDP1 | 10.00461013 |
| TFDP2 | 6.14194512 |
| TFE3 | 6.569891501 |
| TFEC | 5.803330048 |
| TFF2 | 6.371737138 |
| TFF3 | 8.599082901 |
| TFG | 9.657965044 |
| TFIP11 | 8.189852885 |
| TFPI | 11.7728065 |
| TFPI2 | 8.250892151 |
| TFPT | 7.827516759 |
| TFR2 | 9.306228521 |
| TFRC | 11.88921662 |
| TGDS | 10.29869984 |
| TGFA | 6.657843098 |
| TGFB3 | 9.33707238 |
| TGFBI | 12.17749985 |
| TGFBR1 | 6.432542319 |
| TGFBR2 | 11.10658973 |
| TGFBR3 | 11.83241934 |
| TGFBRAP1 | 6.83995261 |
| TGIF1 | 7.421527019 |
| TGIF2 | 7.795470079 |
| TGM2 | 9.877005334 |
| TGM3 | 5.683352994 |
| TGM6 | 7.319673614 |
| TGOLN2 | 11.75575828 |
| TH | 5.868778977 |
| TH1L | 10.3251517 |
| THADA | 7.644876948 |
| THAP1 | 8.087243894 |
| THAP10 | 7.850504909 |
| THAP11 | 10.18887802 |
| THAP4 | 5.641281121 |
| THAP6 | 6.882599915 |
| THAP7 | 6.430284861 |
| THBD | 6.393867832 |
| THBS1 | 10.05983174 |
| THBS2 | 8.948919777 |
| THBS3 | 6.286072718 |
| THEM2 | 12.63749973 |
| THEM4 | 6.073018856 |
| THEX1 | 6.063233924 |
| THH | 6.190242402 |
| THNSL1 | 8.90436865 |
| THOC1 | 9.058327934 |
| THOC2 | 9.179750901 |
| THOC3 | 9.976965704 |
| THOC4 | 9.253607427 |
| THOC5 | 7.406852218 |
| THOC6 | 8.036319534 |
| THOC7 | 12.3161205 |
| THOP1 | 10.89315897 |
| THPO | 7.889141407 |
| THRA | 5.730813004 |
| THRAP1 | 5.885955889 |
| THRAP3 | 6.184833204 |
| THRAP4 | 7.271584664 |
| THRAP5 | 8.421297301 |
| THRB | 6.199529233 |
| THRSP | 11.3379501 |
| THSD3 | 6.235853886 |
| THUMPD1 | 6.403031377 |
| THUMPD2 | 7.367503997 |
| THUMPD3 | 7.115442544 |
| THY1 | 6.466118487 |
| THYN1 | 8.915571548 |
| TIA1 | 6.921430918 |
| TIAF1 | 5.706955392 |
| TIAL1 | 9.191636902 |
| TIAM2 | 5.730615839 |
| TICAM1 | 8.014371512 |
| TICAM2 | 8.254200562 |
| TIE1 | 7.296782116 |
| TIFA | 7.49247049 |
| TIGA1 | 10.70568559 |
| TIGD2 | 9.025896487 |
| TIGD3 | 5.798449288 |
| TIGD4 | 5.755773428 |
| TIGD5 | 8.223469328 |
| TIGD6 | 5.810176477 |
| TIGD7 | 5.897340098 |
| TIMD4 | 10.15924316 |
| TIMELESS | 9.470592436 |
| TIMM10 | 10.94557031 |
| TIMM13 | 6.411593624 |
| TIMM17A | 7.054691463 |
| TIMM17B | 9.123395184 |
| TIMM22 | 9.644151146 |
| TIMM23 | 11.76326035 |
| TIMM44 | 8.098391156 |
| TIMM8A | 8.515435714 |
| TIMM8B | 9.547921528 |
| TIMM9 | 9.767124937 |
| TIMP1 | 14.16569203 |
| TIMP2 | 8.01597522 |
| TIMP3 | 6.275419059 |
| TIMP4 | 6.011286264 |
| TINAG | 6.353327605 |
| TINAGL1 | 6.176512509 |
| TINF2 | 9.948381951 |
| TINP1 | 11.91906104 |
| TIPARP | 10.18235785 |
| TIPIN | 7.649483678 |
| TIPRL | 9.25642453 |
| TIRAP | 6.41580418 |
| TJAP1 | 10.02037268 |
| TJP1 | 9.221107209 |
| TJP2 | 7.760381365 |
| TJP3 | 6.921020346 |
| TK1 | 7.834296457 |
| TK2 | 7.812836939 |
| TKT | 11.93773371 |
| TLCD1 | 6.885874354 |
| TLE1 | 9.482925845 |
| TLE2 | 6.216341284 |
| TLE3 | 5.578441599 |
| TLE6 | 6.920430911 |
| TLK1 | 9.277583934 |
| TLK2 | 7.345761144 |
| TLN1 | 9.454764783 |
| TLN2 | 9.749549061 |
| TLR1 | 7.699494633 |
| TLR3 | 6.38530652 |
| TLR4 | 7.407473417 |
| TLR5 | 7.376886376 |
| TLR7 | 5.431796998 |
| TLR8 | 6.547832815 |
| TLX1 | 5.572876208 |
| TM2D1 | 9.333805955 |
| TM2D2 | 7.39405005 |
| TM2D3 | 9.327642866 |
| TM4SF1 | 12.71026721 |
| TM4SF18 | 9.523371791 |
| TM4SF4 | 14.17088815 |
| TM4SF5 | 10.37812262 |
| TM6SF2 | 7.408849045 |
| TM7SF2 | 9.366624489 |
| TM7SF3 | 9.095897514 |
| TM9SF1 | 9.464277707 |
| TM9SF2 | 12.37301059 |
| TM9SF3 | 10.6049174 |
| TM9SF4 | 9.30876277 |
| TMBIM1 | 9.78499432 |
| TMBIM4 | 13.47087495 |
| TMC3 | 5.71144589 |
| TMC4 | 5.5999968 |
| TMC5 | 6.086368085 |
| TMC6 | 7.860427626 |
| TMCC1 | 6.973259325 |
| TMCO1 | 13.09932263 |
| TMCO3 | 11.15193238 |
| TMCO4 | 5.819366231 |
| TMED1 | 11.14129861 |
| TMED10 | 9.101968721 |
| TMED2 | 12.97378933 |
| TMED3 | 11.55722413 |
| TMED4 | 9.194074177 |
| TMED5 | 12.66806062 |
| TMED6 | 10.21834158 |
| TMED7 | 11.80985948 |
| TMED8 | 6.137797704 |
| TMED9 | 13.0380319 |
| TMEM1 | 6.222127768 |
| TMEM100 | 7.983430395 |
| TMEM101 | 9.799020156 |
| TMEM103 | 7.615485012 |
| TMEM105 | 5.930648663 |
| TMEM106B | 5.736322469 |
| TMEM106C | 11.2935848 |
| TMEM109 | 10.60164433 |
| TMEM11 | 9.02869191 |
| TMEM110 | 7.482065589 |
| TMEM111 | 12.13700223 |
| TMEM113 | 9.372909232 |
| TMEM115 | 9.67606636 |
| TMEM116 | 8.673020926 |
| TMEM117 | 6.914965927 |
| TMEM120A | 10.45571014 |
| TMEM121 | 6.734998836 |
| TMEM123 | 12.80305517 |
| TMEM126A | 12.27119417 |
| TMEM126B | 11.2099225 |
| TMEM127 | 7.754885016 |
| TMEM128 | 6.859802832 |
| TMEM129 | 6.750718263 |
| TMEM133 | 6.435473369 |
| TMEM135 | 9.724683423 |
| TMEM136 | 5.819557123 |
| TMEM138 | 9.26975308 |
| TMEM139 | 5.724085216 |
| TMEM141 | 11.36777782 |
| TMEM143 | 6.379532519 |
| TMEM144 | 7.735405444 |
| TMEM147 | 11.34769388 |
| TMEM149 | 11.20254093 |
| TMEM14A | 11.05075306 |
| TMEM14B | 12.58544206 |
| TMEM14C | 12.45379703 |
| TMEM150 | 6.456277331 |
| TMEM151A | 5.937346321 |
| TMEM154 | 6.840822354 |
| TMEM159 | 5.643339224 |
| TMEM160 | 10.43445073 |
| TMEM161A | 5.79502929 |
| TMEM165 | 8.49219385 |
| TMEM166 | 12.58308844 |
| TMEM167B | 9.011333124 |
| TMEM16A | 8.119629146 |
| TMEM16B | 5.896142626 |
| TMEM16G | 6.665322017 |
| TMEM16K | 6.671165743 |
| TMEM17 | 6.618587624 |
| TMEM170A | 8.453259662 |
| TMEM173 | 7.287973888 |
| TMEM175 | 10.15815704 |
| TMEM176A | 12.7757794 |
| TMEM177 | 9.807813002 |
| TMEM18 | 5.89600676 |
| TMEM180 | 6.762216931 |
| TMEM182 | 6.273779743 |
| TMEM184B | 6.873241432 |
| TMEM185A | 7.963839783 |
| TMEM185B | 8.938257409 |
| TMEM186 | 9.631633168 |
| TMEM189 | 6.033880813 |
| TMEM189-UBE2V1 | 6.029304947 |
| TMEM19 | 6.102977549 |
| TMEM194 | 6.53703542 |
| TMEM195 | 9.063611666 |
| TMEM199 | 8.502076669 |
| TMEM2 | 10.58151552 |
| TMEM20 | 8.20848081 |
| TMEM200A | 6.315659715 |
| TMEM201 | 6.173919391 |
| TMEM203 | 6.64957253 |
| TMEM205 | 13.51817897 |
| TMEM207 | 8.322870064 |
| TMEM22 | 6.587952477 |
| TMEM26 | 7.586053549 |
| TMEM27 | 10.15616346 |
| TMEM30A | 10.19012772 |
| TMEM30B | 8.6136601 |
| TMEM32 | 9.753747719 |
| TMEM33 | 6.147424491 |
| TMEM34 | 5.624403224 |
| TMEM37 | 9.832054023 |
| TMEM38A | 6.540468772 |
| TMEM38B | 9.96423701 |
| TMEM39A | 9.010570371 |
| TMEM39B | 8.765338217 |
| TMEM4 | 11.96525722 |
| TMEM41A | 8.786030468 |
| TMEM41B | 11.51660423 |
| TMEM42 | 9.012257387 |
| TMEM43 | 9.540041896 |
| TMEM44 | 7.301073738 |
| TMEM45A | 11.41086676 |
| TMEM45B | 9.028286229 |
| TMEM47 | 10.70972532 |
| TMEM48 | 7.882056568 |
| TMEM49 | 8.596280092 |
| TMEM5 | 11.20454426 |
| TMEM50A | 7.584530958 |
| TMEM50B | 5.675253114 |
| TMEM51 | 8.387797019 |
| TMEM52 | 5.706891149 |
| TMEM53 | 8.429259299 |
| TMEM54 | 7.591082345 |
| TMEM55A | 6.820305713 |
| TMEM55B | 7.455226514 |
| TMEM56 | 9.024093974 |
| TMEM57 | 8.857819784 |
| TMEM59 | 13.94217778 |
| TMEM60 | 10.92627555 |
| TMEM62 | 9.745612032 |
| TMEM63A | 6.980633052 |
| TMEM63B | 7.525949073 |
| TMEM65 | 7.601918443 |
| TMEM66 | 14.16441706 |
| TMEM68 | 5.95398717 |
| TMEM69 | 8.017225029 |
| TMEM70 | 8.766894393 |
| TMEM71 | 6.651380687 |
| TMEM74 | 5.824582449 |
| TMEM76 | 6.211792734 |
| TMEM77 | 8.539095577 |
| TMEM79 | 5.925771537 |
| TMEM8 | 8.524215301 |
| TMEM80 | 6.857557439 |
| TMEM81 | 6.464625894 |
| TMEM82 | 6.826200141 |
| TMEM83 | 5.826428449 |
| TMEM85 | 12.40146112 |
| TMEM86A | 6.264910204 |
| TMEM86B | 7.322384852 |
| TMEM87A | 11.44097546 |
| TMEM87B | 6.097010974 |
| TMEM88 | 6.305684693 |
| TMEM9 | 9.820005271 |
| TMEM92 | 5.744085537 |
| TMEM93 | 10.19299155 |
| TMEM95 | 5.669513224 |
| TMEM97 | 12.23150989 |
| TMEM98 | 7.688908845 |
| TMEM99 | 10.21351292 |
| TMEM9B | 11.12518179 |
| TMF1 | 7.113298026 |
| TMIGD1 | 5.770768948 |
| TMIGD2 | 6.840589733 |
| TMLHE | 7.13098184 |
| TMOD1 | 9.629561389 |
| TMOD3 | 6.358925438 |
| TMOD4 | 5.566407481 |
| TMPO | 6.290672894 |
| TMPRSS13 | 5.826509736 |
| TMPRSS2 | 9.206429788 |
| TMPRSS6 | 9.650521598 |
| TMPRSS7 | 6.124599181 |
| TMPRSS9 | 6.373901475 |
| TMSB10 | 13.98546904 |
| TMSB4Y | 5.754884912 |
| TMSL3 | 15.34785664 |
| TMTC3 | 5.718689835 |
| TMTC4 | 8.576039332 |
| TMUB1 | 9.476928262 |
| TNC | 6.64467283 |
| TncRNA | 6.485417481 |
| TNF | 6.520440641 |
| TNFAIP1 | 10.42168778 |
| TNFAIP2 | 7.137426508 |
| TNFAIP3 | 8.620613146 |
| TNFAIP6 | 6.404894243 |
| TNFAIP8L1 | 9.601114957 |
| TNFAIP8L2 | 6.02718776 |
| TNFAIP8L3 | 6.946362883 |
| TNFRSF10A | 7.693923039 |
| TNFRSF10B | 7.810332622 |
| TNFRSF10C | 6.088498468 |
| TNFRSF10D | 7.099507862 |
| TNFRSF11B | 9.080933326 |
| TNFRSF12A | 10.12817795 |
| TNFRSF14 | 11.52398929 |
| TNFRSF17 | 5.656316133 |
| TNFRSF19 | 5.833934658 |
| TNFRSF1A | 11.40813812 |
| TNFRSF1B | 10.69760703 |
| TNFRSF21 | 9.19906549 |
| TNFRSF25 | 7.16511948 |
| TNFRSF6B | 8.510892023 |
| TNFRSF7 | 6.63166242 |
| TNFRSF9 | 5.883023879 |
| TNFSF10 | 10.63776072 |
| TNFSF11 | 5.546596989 |
| TNFSF12 | 8.301634536 |
| TNFSF12-TNFSF13 | 5.83023601 |
| TNFSF13 | 5.826694078 |
| TNFSF13B | 8.788646671 |
| TNFSF14 | 7.586776121 |
| TNFSF15 | 6.044694034 |
| TNFSF7 | 5.842888783 |
| TNFSF9 | 6.472724255 |
| TNIK | 5.668220966 |
| TNIP1 | 10.07269355 |
| TNIP2 | 9.96202012 |
| TNIP3 | 5.772627504 |
| TNK2 | 6.872861854 |
| TNKS1BP1 | 6.795162537 |
| TNKS2 | 6.380198979 |
| TNNI3 | 5.62860144 |
| TNP1 | 5.763996259 |
| TNPO1 | 8.218751129 |
| TNPO2 | 9.908470716 |
| TNPO3 | 9.142991926 |
| TNRC15 | 8.007626956 |
| TNRC5 | 6.982666508 |
| TNRC6A | 6.50251576 |
| TNRC6B | 7.381493935 |
| TNRC9 | 7.003299561 |
| TNS1 | 7.234767105 |
| TNS3 | 11.77678334 |
| TNS4 | 8.100170715 |
| TOB1 | 10.34842611 |
| TOB2 | 6.784583193 |
| TOE1 | 6.734436659 |
| TOLLIP | 8.708029635 |
| TOM1 | 5.721381027 |
| TOM1L1 | 8.841220028 |
| TOM1L2 | 6.548383088 |
| TOMM20 | 12.47239858 |
| TOMM22 | 11.39043074 |
| TOMM34 | 10.33316113 |
| TOMM40 | 11.09649551 |
| TOMM7 | 14.21234108 |
| TOMM70A | 11.43846531 |
| TOP1 | 6.141382956 |
| TOP1MT | 5.964088742 |
| TOP2A | 6.887792934 |
| TOP2B | 10.66528928 |
| TOP3B | 7.14643829 |
| TOPBP1 | 9.345445451 |
| TOPORS | 7.684956589 |
| TOR1A | 10.27589068 |
| TOR1AIP1 | 9.91989317 |
| TOR1AIP2 | 6.889346055 |
| TOR1B | 9.289211434 |
| TOR2A | 7.192530103 |
| TOR3A | 9.398576888 |
| TP53 | 5.766536693 |
| TP53AP1 | 8.855203767 |
| TP53BP1 | 6.306783847 |
| TP53BP2 | 8.299113471 |
| TP53I11 | 5.64602961 |
| TP53I13 | 9.755243198 |
| TP53I3 | 8.891920401 |
| TP53INP1 | 11.01280385 |
| TP53INP2 | 8.092429369 |
| TP53RK | 8.47475421 |
| TPCN1 | 5.742929232 |
| TPCN2 | 8.631922645 |
| TPD52 | 6.353356848 |
| TPD52L1 | 8.50686777 |
| TPD52L2 | 11.77176832 |
| TPD52L3 | 8.251501426 |
| TPI1 | 12.41282181 |
| TPK1 | 8.564678805 |
| TPM1 | 11.19849824 |
| TPM2 | 9.194684761 |
| TPM3 | 9.459018143 |
| TPM4 | 7.070306263 |
| TPMT | 8.845314309 |
| TPP1 | 10.5696271 |
| TPP2 | 7.100525823 |
| TPPP2 | 5.728884669 |
| TPR | 8.533122204 |
| TPRG1L | 12.39524601 |
| TPRKB | 9.626113694 |
| TPSAB1 | 6.147330221 |
| TPSG1 | 5.499515176 |
| TPST1 | 11.12058487 |
| TPST2 | 8.532506072 |
| TPT1 | 15.67786929 |
| TPX2 | 6.571562385 |
| TRA2A | 7.970927795 |
| TRABD | 11.37862985 |
| TRADD | 6.427038472 |
| TRAF1 | 5.808501422 |
| TRAF2 | 5.945250662 |
| TRAF3 | 6.346311623 |
| TRAF3IP2 | 8.965784368 |
| TRAF4 | 6.295973615 |
| TRAF5 | 5.733915985 |
| TRAF6 | 6.668879642 |
| TRAFD1 | 8.00264842 |
| TRAIP | 5.801010465 |
| TRAK1 | 7.693548416 |
| TRAK2 | 9.142850445 |
| TRAM1 | 14.27755535 |
| TRAM2 | 9.384986268 |
| TRAP1 | 9.851861515 |
| TRAPPC1 | 9.745345798 |
| TRAPPC2 | 8.413756733 |
| TRAPPC2L | 12.06447325 |
| TRAPPC3 | 11.41300673 |
| TRAPPC4 | 10.48663866 |
| TRAPPC6A | 9.818930532 |
| TRAPPC6B | 8.946067731 |
| TRAT1 | 5.543883057 |
| TRDN | 6.109958587 |
| TREM1 | 6.08726042 |
| TREML1 | 5.90527362 |
| TREML2 | 5.958949622 |
| TREX1 | 5.615524011 |
| TRHDE | 5.539377849 |
| TRIAD3 | 8.209905166 |
| TRIAP1 | 10.39329184 |
| TRIB1 | 11.90238022 |
| TRIB2 | 7.08310473 |
| TRIB3 | 11.89916975 |
| TRIM10 | 6.070648335 |
| TRIM11 | 6.811573598 |
| TRIM13 | 7.674957713 |
| TRIM15 | 9.292539252 |
| TRIM16 | 5.720513418 |
| TRIM17 | 6.286756258 |
| TRIM2 | 8.927345886 |
| TRIM21 | 8.06444842 |
| TRIM22 | 7.190057432 |
| TRIM23 | 7.604242099 |
| TRIM24 | 10.37942722 |
| TRIM25 | 6.275010678 |
| TRIM26 | 8.77529137 |
| TRIM28 | 7.32597989 |
| TRIM3 | 5.691254771 |
| TRIM32 | 7.230601123 |
| TRIM33 | 9.346988327 |
| TRIM35 | 6.202117371 |
| TRIM36 | 5.831227283 |
| TRIM37 | 7.684843446 |
| TRIM38 | 7.726547713 |
| TRIM39 | 8.454607965 |
| TRIM4 | 6.819101999 |
| TRIM41 | 7.408824377 |
| TRIM43 | 8.161515367 |
| TRIM44 | 10.24967822 |
| TRIM47 | 6.626465497 |
| TRIM5 | 7.741102633 |
| TRIM52 | 5.847445287 |
| TRIM54 | 5.972329114 |
| TRIM55 | 9.334573783 |
| TRIM56 | 7.444449823 |
| TRIM58 | 5.979256709 |
| TRIM6 | 6.324762986 |
| TRIM61 | 6.51670382 |
| TRIM67 | 6.256576743 |
| TRIM68 | 9.065663188 |
| TRIM73 | 7.508315773 |
| TRIM8 | 11.23498004 |
| TRIM9 | 6.330413339 |
| TRIO | 5.97595406 |
| TRIP10 | 5.709760594 |
| TRIP11 | 9.311006101 |
| TRIP12 | 9.545230887 |
| TRIP13 | 6.376280217 |
| TRIP4 | 8.444136289 |
| TRIP6 | 9.822695649 |
| TRIT1 | 8.582308891 |
| TRK1 | 9.895830582 |
| TRMT1 | 7.931496457 |
| TRMT12 | 9.100063432 |
| TRMT2A | 6.678470247 |
| TRMT6 | 6.686820734 |
| TRMU | 6.540514564 |
| TRNT1 | 7.223103497 |
| TROVE2 | 6.944513824 |
| TRPA1 | 5.743922723 |
| TRPC4 | 6.046686202 |
| TRPC4AP | 9.567546089 |
| TRPC5 | 6.321804948 |
| TRPM4 | 10.62390739 |
| TRPM6 | 6.033848373 |
| TRPM7 | 5.964000717 |
| TRPM8 | 10.43812283 |
| TRPS1 | 6.16334079 |
| TRPT1 | 9.233193238 |
| TRPV1 | 5.897051045 |
| TRPV2 | 7.050567816 |
| TRPV3 | 5.706284669 |
| TRPV4 | 8.183621878 |
| TRPV5 | 5.788810732 |
| TRQ1 | 7.585525848 |
| TRRAP | 9.29087183 |
| TRSPAP1 | 6.784029212 |
| TRUB1 | 5.527496929 |
| TRUB2 | 12.04905711 |
| TRY6 | 6.351632834 |
| TSC1 | 6.396916221 |
| TSC2 | 6.142078212 |
| TSC22D1 | 11.45735974 |
| TSC22D2 | 8.038992734 |
| TSC22D3 | 10.65427548 |
| TSC22D4 | 10.41199434 |
| TSEN2 | 8.33832591 |
| TSEN34 | 10.09444988 |
| TSEN54 | 6.636915594 |
| TSFM | 11.46134252 |
| TSG101 | 11.37586734 |
| TSGA14 | 5.721056309 |
| TSKU | 7.133394331 |
| TSLP | 6.010203129 |
| TSN | 6.985382984 |
| TSNAX | 9.842876495 |
| TSP50 | 5.694770539 |
| TSPAN1 | 7.07013667 |
| TSPAN10 | 7.122148633 |
| TSPAN12 | 9.67219847 |
| TSPAN13 | 11.40066165 |
| TSPAN14 | 8.139163893 |
| TSPAN17 | 11.02096004 |
| TSPAN18 | 6.656708302 |
| TSPAN3 | 9.977221693 |
| TSPAN31 | 10.49405194 |
| TSPAN32 | 5.664695046 |
| TSPAN33 | 10.30085654 |
| TSPAN4 | 8.078619785 |
| TSPAN5 | 6.039497004 |
| TSPAN6 | 11.50245359 |
| TSPAN7 | 8.922281432 |
| TSPAN8 | 10.95573692 |
| TSPAN9 | 12.48268901 |
| TSPO | 10.78955073 |
| TSPY2 | 5.783413072 |
| TSPYL1 | 10.81528976 |
| TSPYL2 | 6.146256322 |
| TSPYL5 | 7.069984006 |
| TSPYL6 | 7.899846339 |
| TSR1 | 8.101718705 |
| TSR2 | 10.76671553 |
| TSRC1 | 6.945710023 |
| TSSC1 | 8.979312882 |
| TSSC4 | 9.841298262 |
| TSSK1B | 5.810517226 |
| TSSK6 | 5.760221662 |
| TST | 14.42860883 |
| TSTA3 | 9.220830271 |
| TTBK2 | 6.105784854 |
| TTC1 | 8.915703399 |
| TTC10 | 7.239388221 |
| TTC13 | 7.699596518 |
| TTC14 | 7.400711188 |
| TTC15 | 9.269232145 |
| TTC16 | 6.513007053 |
| TTC17 | 8.402196304 |
| TTC19 | 10.47223687 |
| TTC23 | 8.473990193 |
| TTC25 | 5.804119386 |
| TTC26 | 6.556406158 |
| TTC27 | 9.626882941 |
| TTC3 | 11.15092683 |
| TTC32 | 8.730473074 |
| TTC35 | 7.874819144 |
| TTC37 | 9.995012929 |
| TTC39B | 7.91289567 |
| TTC4 | 10.72478909 |
| TTC5 | 8.836184259 |
| TTC7A | 6.777385871 |
| TTC7B | 5.666432045 |
| TTC8 | 7.692194625 |
| TTC9B | 5.829049529 |
| TTC9C | 6.286506387 |
| TTF1 | 7.304091539 |
| TTF2 | 7.343999281 |
| TTK | 5.765205722 |
| TTL | 9.244330425 |
| TTLL1 | 5.995072452 |
| TTLL12 | 8.840773553 |
| TTLL13 | 6.118527061 |
| TTLL3 | 7.065595753 |
| TTLL4 | 5.53686475 |
| TTLL5 | 6.991429059 |
| TTLL7 | 5.894959771 |
| TTN | 5.858846951 |
| TTR | 15.11395628 |
| TTRAP | 6.694812738 |
| TTTY1 | 6.032551736 |
| TTTY14 | 7.20076175 |
| TTTY15 | 6.606469867 |
| TTTY6 | 5.996284268 |
| TTYH1 | 5.802995529 |
| TTYH3 | 7.095859215 |
| TUB | 5.540872292 |
| TUBA1A | 11.05874571 |
| TUBA1B | 14.58653392 |
| TUBA1C | 13.74867287 |
| TUBA3D | 8.537333821 |
| TUBA4 | 6.045678511 |
| TUBA4A | 9.567482531 |
| TUBB | 11.23017959 |
| TUBB1 | 5.701637031 |
| TUBB2B | 6.007171674 |
| TUBB2C | 11.73810009 |
| TUBB4Q | 8.339371897 |
| TUBB6 | 8.099148045 |
| TUBB8 | 7.908570733 |
| TUBD1 | 7.566280582 |
| TUBE1 | 8.869294837 |
| TUBG1 | 9.038674878 |
| TUBG2 | 7.330479179 |
| TUBGCP2 | 9.014121948 |
| TUBGCP3 | 7.777814602 |
| TUBGCP4 | 7.539002243 |
| TUBGCP5 | 7.966429462 |
| TUBGCP6 | 6.031616569 |
| TUFM | 11.03811776 |
| TUFT1 | 8.738389575 |
| TUG1 | 10.18007088 |
| TULP1 | 5.867530582 |
| TULP2 | 8.461877892 |
| TULP3 | 6.592164882 |
| TULP4 | 7.658321612 |
| TUSC1 | 7.500520242 |
| TUSC2 | 7.202965305 |
| TUSC3 | 7.358986252 |
| TUSC4 | 11.35672699 |
| TUT1 | 7.488175946 |
| TWF2 | 8.79899816 |
| TWIST2 | 5.886271572 |
| TWISTNB | 7.546017619 |
| TWSG1 | 6.638713742 |
| TXLNA | 12.04597513 |
| TXLNB | 5.766737511 |
| TXN | 14.17878648 |
| TXN2 | 8.386863376 |
| TXNDC | 11.56420507 |
| TXNDC11 | 8.861928461 |
| TXNDC12 | 11.20103041 |
| TXNDC13 | 8.858911933 |
| TXNDC14 | 11.49560588 |
| TXNDC16 | 5.862294239 |
| TXNDC3 | 5.667632291 |
| TXNDC4 | 6.568097421 |
| TXNDC5 | 10.00130044 |
| TXNDC9 | 10.39417938 |
| TXNIP | 12.64608811 |
| TXNL1 | 10.12308763 |
| TXNL4A | 5.880801796 |
| TXNL4B | 6.774785958 |
| TXNL5 | 13.85342858 |
| TXNRD1 | 9.461630863 |
| TXNRD2 | 10.67070746 |
| TYK2 | 10.26923108 |
| TYMS | 8.654399786 |
| TYRO3 | 5.621539102 |
| TYROBP | 11.96981349 |
| TYSND1 | 9.335487092 |
| TYW1 | 8.108237577 |
| TYW3 | 8.488542326 |
| U1SNRNPBP | 6.936138961 |
| U2AF1 | 10.33174948 |
| U2AF1L2 | 6.043976482 |
| U2AF1L3 | 6.638598249 |
| U2AF2 | 10.58434265 |
| UACA | 6.498509975 |
| UAP1 | 11.38467874 |
| UBA2 | 5.780519513 |
| UBA52 | 14.78436276 |
| UBA7 | 8.563263466 |
| UBAC1 | 10.15382602 |
| UBAC2 | 10.72604185 |
| UBAP1 | 9.898175873 |
| UBAP2 | 9.176395716 |
| UBAP2L | 8.436418235 |
| UBB | 14.62944296 |
| UBC | 14.13774018 |
| UBD | 9.96211886 |
| UBE1 | 9.634288413 |
| UBE1C | 9.928736082 |
| UBE1DC1 | 8.395998538 |
| UBE1L2 | 9.57747272 |
| UBE2B | 5.852638157 |
| UBE2C | 6.522331811 |
| UBE2CBP | 5.909774613 |
| UBE2D2 | 6.523804309 |
| UBE2D3 | 9.207264926 |
| UBE2D4 | 10.44649022 |
| UBE2E1 | 12.42669669 |
| UBE2E2 | 10.25272091 |
| UBE2E3 | 7.131757012 |
| UBE2F | 10.59618114 |
| UBE2G1 | 9.040503804 |
| UBE2G2 | 10.35889928 |
| UBE2H | 7.925433122 |
| UBE2I | 11.01252273 |
| UBE2J1 | 11.14966275 |
| UBE2J2 | 7.741058012 |
| UBE2L3 | 7.303739246 |
| UBE2L6 | 11.21235334 |
| UBE2M | 11.62696377 |
| UBE2N | 10.0633363 |
| UBE2O | 6.170031803 |
| UBE2Q1 | 8.541617458 |
| UBE2Q2 | 9.237820501 |
| UBE2R2 | 6.315474112 |
| UBE2S | 8.921164476 |
| UBE2T | 8.091597103 |
| UBE2U | 5.822145017 |
| UBE2V1 | 6.511019034 |
| UBE2V2 | 11.01765342 |
| UBE2W | 7.653872224 |
| UBE2Z | 9.833020913 |
| UBE3A | 10.6741483 |
| UBE3B | 7.755471682 |
| UBE3C | 9.287776422 |
| UBE4A | 9.405667187 |
| UBE4B | 9.148090305 |
| UBIAD1 | 10.23091146 |
| UBL3 | 10.1643211 |
| UBL4A | 7.039704602 |
| UBL5 | 12.62657993 |
| UBL7 | 6.625737617 |
| UBLCP1 | 9.555162424 |
| UBN1 | 9.827863724 |
| UBOX5 | 5.925435439 |
| UBP1 | 10.32483756 |
| UBPH | 8.314872662 |
| UBQLN1 | 10.0229822 |
| UBQLN2 | 10.61797113 |
| UBQLN3 | 6.085194891 |
| UBQLN4 | 10.11500576 |
| UBR1 | 6.574476793 |
| UBR2 | 9.068902867 |
| UBR5 | 7.754820762 |
| UBTD1 | 7.519833626 |
| UBTF | 6.679841414 |
| UBXD2 | 12.55579718 |
| UBXD3 | 6.266363055 |
| UBXD5 | 5.621255852 |
| UBXD6 | 9.228195123 |
| UBXD8 | 11.76483267 |
| UBXN1 | 10.32613818 |
| UBXN2A | 9.289176413 |
| UBXN6 | 9.763597784 |
| UCHL1 | 5.76353881 |
| UCHL3 | 11.44113966 |
| UCHL5 | 10.02078795 |
| UCHL5IP | 6.329445942 |
| UCK1 | 8.455027606 |
| UCK2 | 9.256216196 |
| UCKL1 | 8.43467125 |
| UCN | 6.346953064 |
| UCP1 | 5.822920808 |
| UCP2 | 7.220148018 |
| UCRC | 7.626390199 |
| UEV3 | 7.3165932 |
| UFC1 | 10.41454249 |
| UFD1L | 6.570882693 |
| UFM1 | 10.71127799 |
| UFSP2 | 10.46882947 |
| UGCG | 10.09795069 |
| UGCGL1 | 8.235842661 |
| UGCGL2 | 8.242727068 |
| UGDH | 12.3738099 |
| UGP2 | 10.65838829 |
| UGT1A1 | 9.609930374 |
| UGT1A10 | 5.689893494 |
| UGT1A3 | 5.369781983 |
| UGT1A4 | 8.697207927 |
| UGT1A6 | 7.491732271 |
| UGT1A7 | 5.994664663 |
| UGT1A8 | 5.669887935 |
| UGT1A9 | 5.80687871 |
| UGT2A3 | 9.336644147 |
| UGT2B10 | 13.01902352 |
| UGT2B11 | 13.89756746 |
| UGT2B15 | 8.904748412 |
| UGT2B17 | 12.81929222 |
| UGT2B28 | 9.241182174 |
| UGT2B4 | 13.45705658 |
| UGT2B7 | 11.93483899 |
| UGT3A1 | 9.838752454 |
| UHMK1 | 6.793474637 |
| UHRF1 | 6.792204344 |
| UHRF1BP1L | 6.793093019 |
| UHRF2 | 6.942668013 |
| UIMC1 | 9.714968113 |
| UIP1 | 9.377268117 |
| ULBP3 | 5.749098254 |
| ULK1 | 10.50663114 |
| ULK2 | 6.099138606 |
| ULK3 | 5.632504226 |
| UMOD | 5.713104946 |
| UMPS | 5.624697086 |
| UNC119 | 8.872276878 |
| UNC13B | 7.723288542 |
| UNC45A | 6.900714742 |
| UNC50 | 11.75993997 |
| UNC5CL | 8.341485191 |
| UNC84A | 8.8819444 |
| UNC84B | 9.398318977 |
| UNC93A | 7.22459708 |
| UNC93B1 | 5.44394433 |
| UNG | 8.242790808 |
| UNKL | 6.316383915 |
| UNQ1940 | 7.524751192 |
| UNQ2541 | 5.522021771 |
| UNQ3033 | 5.643294791 |
| UNQ830 | 7.926387315 |
| UNQ9391 | 6.70552148 |
| UNQ9433 | 5.553150762 |
| UPB1 | 12.11091643 |
| UPF2 | 8.585578638 |
| UPF3A | 8.132616363 |
| UPF3B | 8.222276201 |
| UPK1A | 5.592156062 |
| UPK1B | 6.342001391 |
| UPK3B | 6.249103979 |
| UPP1 | 10.52818808 |
| UPP2 | 6.831967931 |
| UPRT | 7.657069336 |
| UQCC | 5.976333902 |
| UQCR | 6.119178814 |
| UQCRB | 11.04616066 |
| UQCRC1 | 11.70153679 |
| UQCRC2 | 11.21777852 |
| UQCRFS1 | 13.0771028 |
| UQCRH | 10.7236107 |
| URG4 | 8.800380678 |
| URM1 | 10.9186475 |
| UROD | 12.81660447 |
| UROS | 11.55276772 |
| USF1 | 8.131521222 |
| USF2 | 10.21647431 |
| USH1G | 5.85812555 |
| USH2A | 6.427038542 |
| USHBP1 | 6.137898533 |
| USMG5 | 9.03802243 |
| USP1 | 8.244298709 |
| USP10 | 8.545716996 |
| USP11 | 6.960711464 |
| USP12 | 6.379255808 |
| USP13 | 8.079006513 |
| USP14 | 9.198060725 |
| USP15 | 7.762062236 |
| USP16 | 10.00499475 |
| USP18 | 6.478680351 |
| USP2 | 5.934476057 |
| USP20 | 5.945757988 |
| USP21 | 5.623152624 |
| USP22 | 6.208485243 |
| USP24 | 8.75924449 |
| USP25 | 5.906132412 |
| USP29 | 5.838085553 |
| USP3 | 9.670734544 |
| USP30 | 7.196370619 |
| USP32 | 5.624689841 |
| USP33 | 7.609686065 |
| USP34 | 6.89528195 |
| USP36 | 6.095412307 |
| USP37 | 7.142542183 |
| USP38 | 10.79881327 |
| USP39 | 8.789181247 |
| USP4 | 7.11892026 |
| USP41 | 6.291754271 |
| USP42 | 5.479935139 |
| USP46 | 5.838051513 |
| USP47 | 7.256002898 |
| USP48 | 6.711008677 |
| USP49 | 10.84059727 |
| USP5 | 10.5579691 |
| USP51 | 7.679141081 |
| USP53 | 5.614050159 |
| USP6 | 5.83795252 |
| USP7 | 8.696466668 |
| USP8 | 9.080544617 |
| USP9X | 9.987020628 |
| USP9Y | 5.828359536 |
| USPL1 | 8.112073257 |
| UST | 5.638712818 |
| UTP11L | 7.97510077 |
| UTP14A | 7.52908756 |
| UTP14C | 9.591151126 |
| UTP15 | 6.810358913 |
| UTP23 | 8.590113494 |
| UTP3 | 9.759443529 |
| UTP6 | 10.37315558 |
| UTRN | 5.929177782 |
| UTS2 | 7.55365715 |
| UTX | 7.713418465 |
| UTY | 6.348544861 |
| UVRAG | 8.052616535 |
| UXS1 | 7.889633009 |
| UXT | 9.880509724 |
| VAC14 | 6.989697933 |
| VAMP1 | 6.375931107 |
| VAMP2 | 5.80364856 |
| VAMP3 | 10.31942598 |
| VAMP4 | 8.346707562 |
| VAMP5 | 12.0021425 |
| VAMP7 | 10.85816412 |
| VAMP8 | 13.28416093 |
| VANGL1 | 5.885238211 |
| VAPA | 7.205066573 |
| VAPB | 6.226519161 |
| VARS | 10.05649771 |
| VARS2 | 9.844382686 |
| VASH1 | 6.735453808 |
| VASP | 6.921629715 |
| VAT1 | 9.116746114 |
| VAT1L | 5.700925387 |
| VAV1 | 6.718649191 |
| VAV2 | 9.313685527 |
| VAV3 | 6.206143682 |
| VAX1 | 5.914384822 |
| VAX2 | 6.077494984 |
| VBP1 | 10.74651353 |
| VCAM1 | 7.858591649 |
| VCAN | 8.666494694 |
| VCL | 10.96457044 |
| VCP | 9.490482412 |
| VCPIP1 | 9.061179299 |
| VCX2 | 5.976204276 |
| VDAC1 | 12.02937551 |
| VDAC2 | 13.09891341 |
| VDAC3 | 12.51030072 |
| VDP | 11.88109455 |
| VEGFA | 6.476674059 |
| VEGFB | 9.512201859 |
| VEGFC | 6.131182356 |
| VENTX | 6.119714539 |
| VEZF1 | 10.39310499 |
| VEZT | 10.00430094 |
| VGLL4 | 9.34049425 |
| VHL | 8.331397274 |
| VIL1 | 7.956597381 |
| VIL2 | 11.15694309 |
| VIM | 11.56545495 |
| VIPR1 | 6.452690869 |
| VKORC1 | 12.38858461 |
| VKORC1L1 | 9.979737536 |
| VMAC | 6.398489218 |
| VMD2 | 6.345644685 |
| VMD2L3 | 7.459100445 |
| VMO1 | 8.493359992 |
| VN1R4 | 5.997512236 |
| VN1R5 | 6.361364162 |
| VNN1 | 12.93496745 |
| VNN2 | 8.542188048 |
| VNN3 | 7.675412308 |
| VPRBP | 6.386241319 |
| VPREB3 | 6.196801421 |
| VPS11 | 7.169484189 |
| VPS13A | 6.484297137 |
| VPS13B | 5.848857571 |
| VPS13D | 6.336077879 |
| VPS16 | 7.826986785 |
| VPS18 | 7.681307588 |
| VPS24 | 8.02021251 |
| VPS25 | 9.784389915 |
| VPS26 | 9.045821247 |
| VPS26B | 8.919921827 |
| VPS28 | 10.62493654 |
| VPS29 | 13.00537579 |
| VPS33A | 7.77976385 |
| VPS33B | 7.499827635 |
| VPS35 | 10.86914339 |
| VPS36 | 9.282435391 |
| VPS37A | 8.469167967 |
| VPS37B | 7.258467072 |
| VPS37C | 10.49612344 |
| VPS37D | 7.649429474 |
| VPS39 | 7.255604554 |
| VPS41 | 7.56685444 |
| VPS45A | 7.870580441 |
| VPS4A | 7.488436948 |
| VPS4B | 10.1303514 |
| VPS52 | 7.933735718 |
| VPS54 | 7.594959344 |
| VPS72 | 8.043640046 |
| VRK1 | 7.936660659 |
| VRK2 | 7.179401705 |
| VRK3 | 8.376959017 |
| VSIG2 | 6.565370083 |
| VSIG4 | 8.619992601 |
| VSIG6 | 5.841582944 |
| VSNL1 | 7.517133229 |
| VSTM3 | 7.865182123 |
| VTA1 | 10.47073529 |
| VTCN1 | 5.679492564 |
| VTI1A | 7.365425188 |
| VTI1B | 8.62542848 |
| VTN | 14.35625951 |
| VWA1 | 9.756249035 |
| VWA5A | 6.389477726 |
| VWCE | 7.192819641 |
| VWF | 9.02268183 |
| WAC | 8.029584984 |
| WARS | 9.193986248 |
| WAS | 9.123484709 |
| WASF2 | 7.341299759 |
| WASF3 | 7.882620133 |
| WASL | 11.26761365 |
| WASPIP | 7.82054495 |
| WBP1 | 10.43605193 |
| WBP2 | 12.8983234 |
| WBP4 | 9.290488223 |
| WBP5 | 9.677373074 |
| WBSCR14 | 8.562289877 |
| WBSCR16 | 6.027455417 |
| WBSCR18 | 6.334774533 |
| WBSCR20B | 5.601564072 |
| WBSCR22 | 10.44162291 |
| WDFY1 | 10.70419323 |
| WDFY2 | 7.298935883 |
| WDFY3 | 6.163539691 |
| WDHD1 | 5.693240822 |
| WDR1 | 9.464696228 |
| WDR12 | 9.781759325 |
| WDR13 | 9.883443611 |
| WDR18 | 12.02215741 |
| WDR19 | 8.63985086 |
| WDR20 | 8.722195378 |
| WDR21A | 7.372132275 |
| WDR21B | 6.353025601 |
| WDR22 | 8.256101347 |
| WDR23 | 8.705730229 |
| WDR24 | 5.754435376 |
| WDR25 | 8.458207447 |
| WDR26 | 7.582986471 |
| WDR32 | 9.945688157 |
| WDR33 | 9.673135518 |
| WDR34 | 7.696692902 |
| WDR35 | 5.745737822 |
| WDR36 | 9.094166182 |
| WDR37 | 7.45838985 |
| WDR4 | 8.091854601 |
| WDR40A | 10.08536643 |
| WDR41 | 8.697527898 |
| WDR42A | 10.67775873 |
| WDR43 | 7.772485389 |
| WDR44 | 7.155675267 |
| WDR45 | 7.177145651 |
| WDR45L | 9.346204012 |
| WDR46 | 8.354978194 |
| WDR47 | 5.480727701 |
| WDR48 | 8.44922691 |
| WDR5 | 6.932901522 |
| WDR50 | 8.464163697 |
| WDR51B | 8.495447439 |
| WDR53 | 7.581877759 |
| WDR54 | 8.252323645 |
| WDR55 | 8.96575423 |
| WDR57 | 9.446390589 |
| WDR59 | 9.656349584 |
| WDR5B | 5.81664714 |
| WDR6 | 9.902203749 |
| WDR60 | 7.326166031 |
| WDR61 | 11.89083073 |
| WDR63 | 5.838825671 |
| WDR64 | 5.508186698 |
| WDR65 | 6.988849118 |
| WDR66 | 6.14407503 |
| WDR67 | 7.329454887 |
| WDR68 | 8.684932943 |
| WDR7 | 7.493480036 |
| WDR70 | 8.42586778 |
| WDR72 | 12.66446544 |
| WDR73 | 6.906876816 |
| WDR74 | 7.791148533 |
| WDR75 | 10.25794405 |
| WDR79 | 7.469723788 |
| WDR8 | 8.138563598 |
| WDR81 | 7.381011239 |
| WDR86 | 6.026799825 |
| WDR89 | 6.036541835 |
| WDR91 | 6.169975565 |
| WDSOF1 | 8.493679278 |
| WDSUB1 | 8.03611914 |
| WEE1 | 7.809730883 |
| WFDC1 | 6.623552855 |
| WFDC5 | 5.649108339 |
| WFS1 | 9.870482488 |
| WHDC1 | 7.190806009 |
| WHSC1 | 5.773200919 |
| WHSC1L1 | 5.740837386 |
| WHSC2 | 6.353115897 |
| WIBG | 9.27734922 |
| WIF1 | 5.684715793 |
| WIPF2 | 6.027571692 |
| WIPI1 | 9.226057678 |
| WISP1 | 6.171348657 |
| WISP3 | 5.788964527 |
| WNK1 | 6.404363192 |
| WNK3 | 6.679313784 |
| WNT2 | 6.48417792 |
| WNT3 | 6.398633892 |
| WNT4 | 6.100429817 |
| WNT5A | 6.11085695 |
| WNT5B | 7.04418591 |
| WNT7A | 6.000124993 |
| WNT8A | 5.757889327 |
| WNT9A | 5.693853697 |
| WRB | 9.653844949 |
| WRN | 8.175874734 |
| WRNIP1 | 8.450207565 |
| WSB1 | 7.150373413 |
| WSB2 | 10.58109848 |
| WSCD1 | 6.127547117 |
| WT1 | 6.091808115 |
| WTAP | 8.014018711 |
| WTIP | 5.904131231 |
| WWC1 | 9.891385496 |
| WWC2 | 6.094505916 |
| WWC3 | 6.378795936 |
| WWOX | 6.230976967 |
| WWP1 | 12.50401343 |
| WWP2 | 6.548685103 |
| WWTR1 | 5.782753869 |
| XAB1 | 10.63452085 |
| XAB2 | 9.076059526 |
| XAF1 | 9.977032888 |
| XBP1 | 13.51153218 |
| XCR1 | 6.619762951 |
| XDH | 9.186264032 |
| XIAP | 7.964777224 |
| XIST | 7.322072961 |
| XK | 7.868250486 |
| XKR6 | 7.77478638 |
| XKR8 | 5.738628875 |
| XLKD1 | 10.11806942 |
| XPA | 8.310243065 |
| XPC | 9.121624441 |
| XPNPEP1 | 11.20089725 |
| XPNPEP2 | 8.444340785 |
| XPNPEP3 | 5.561338545 |
| XPO1 | 9.695695221 |
| XPO4 | 8.81233586 |
| XPO5 | 7.873305895 |
| XPO6 | 8.825210093 |
| XPO7 | 5.826220766 |
| XPOT | 10.08449213 |
| XPR1 | 8.806432133 |
| XRCC1 | 7.105742817 |
| XRCC2 | 5.793983616 |
| XRCC5 | 12.01042445 |
| XRCC6 | 9.803205945 |
| XRCC6BP1 | 8.974967233 |
| XRN1 | 6.254842117 |
| XRN2 | 8.540847669 |
| XTP3TPA | 10.12965484 |
| XYLB | 7.921661232 |
| XYLT2 | 9.361612673 |
| YAF2 | 6.646612754 |
| YAP1 | 11.36861202 |
| YARS | 12.39136376 |
| YARS2 | 9.808153696 |
| YBX1 | 13.05511976 |
| YBX2 | 5.418539722 |
| YDJC | 5.730204448 |
| YEATS2 | 7.409367087 |
| YEATS4 | 8.210965129 |
| YES1 | 11.55516324 |
| YIF1A | 12.75240396 |
| YIF1B | 8.520280053 |
| YIPF1 | 10.72683857 |
| YIPF2 | 7.966212072 |
| YIPF3 | 10.7953915 |
| YIPF4 | 10.35361242 |
| YIPF5 | 8.541702971 |
| YIPF6 | 11.74219024 |
| YKT6 | 6.034441263 |
| YME1L1 | 9.525336095 |
| YOD1 | 8.565509737 |
| YPEL1 | 5.800972919 |
| YPEL2 | 9.982208302 |
| YPEL3 | 7.43308264 |
| YPEL5 | 10.60597447 |
| YRDC | 10.45549852 |
| YTHDC1 | 7.427048776 |
| YTHDC2 | 6.970538635 |
| YTHDF1 | 10.12124039 |
| YTHDF2 | 10.7017398 |
| YTHDF3 | 9.253920598 |
| YWHAB | 10.56485475 |
| YWHAE | 7.948932402 |
| YWHAG | 8.828336395 |
| YWHAH | 13.31607498 |
| YWHAQ | 13.57803613 |
| YWHAZ | 8.861259835 |
| YY1 | 12.04659977 |
| YY1AP1 | 8.216601724 |
| ZA20D1 | 8.226149698 |
| ZACN | 6.36344984 |
| ZADH2 | 8.994547 |
| ZAK | 8.638807154 |
| ZAP70 | 5.946070499 |
| ZAR1 | 5.685493038 |
| ZBED1 | 11.11676308 |
| ZBED3 | 6.814141786 |
| ZBED4 | 7.497263944 |
| ZBED5 | 8.230463427 |
| ZBP1 | 5.997876308 |
| ZBTB10 | 5.720511204 |
| ZBTB11 | 6.532241471 |
| ZBTB16 | 9.267465919 |
| ZBTB17 | 7.787003893 |
| ZBTB2 | 6.041968125 |
| ZBTB20 | 8.826840512 |
| ZBTB22 | 8.209343173 |
| ZBTB24 | 7.645337721 |
| ZBTB25 | 6.666332557 |
| ZBTB26 | 5.479594217 |
| ZBTB3 | 7.608220537 |
| ZBTB33 | 10.35886253 |
| ZBTB34 | 6.509263254 |
| ZBTB39 | 6.402887788 |
| ZBTB4 | 7.152171974 |
| ZBTB40 | 7.055212901 |
| ZBTB41 | 6.560818976 |
| ZBTB43 | 8.634875448 |
| ZBTB45 | 6.823983505 |
| ZBTB46 | 5.969393641 |
| ZBTB47 | 5.795333493 |
| ZBTB48 | 7.98230198 |
| ZBTB5 | 6.84507324 |
| ZBTB7A | 8.701542974 |
| ZBTB7B | 5.889199997 |
| ZBTB9 | 8.049015247 |
| ZC3H10 | 6.601876275 |
| ZC3H12A | 7.992756362 |
| ZC3H14 | 9.521292115 |
| ZC3H18 | 7.234730382 |
| ZC3H3 | 5.942604348 |
| ZC3H5 | 8.356463844 |
| ZC3H6 | 5.619679605 |
| ZC3H7A | 9.450186313 |
| ZC3H8 | 7.955925847 |
| ZC3HAV1 | 7.427607752 |
| ZC3HC1 | 10.20339053 |
| ZCCHC10 | 5.786455791 |
| ZCCHC11 | 7.262449113 |
| ZCCHC12 | 5.637448664 |
| ZCCHC14 | 9.359572558 |
| ZCCHC16 | 5.603979744 |
| ZCCHC17 | 9.31229841 |
| ZCCHC2 | 6.283349033 |
| ZCCHC3 | 6.88988284 |
| ZCCHC6 | 9.908090196 |
| ZCCHC7 | 9.289020893 |
| ZCCHC8 | 6.764129107 |
| ZCCHC9 | 11.01989096 |
| ZCRB1 | 8.216103226 |
| ZCSL3 | 7.919672985 |
| ZCWPW1 | 8.228017708 |
| ZDHHC1 | 5.721627207 |
| ZDHHC11 | 6.100169661 |
| ZDHHC12 | 8.176302576 |
| ZDHHC13 | 5.979277585 |
| ZDHHC14 | 10.82753072 |
| ZDHHC16 | 11.61251214 |
| ZDHHC17 | 7.618553818 |
| ZDHHC18 | 6.438037045 |
| ZDHHC19 | 6.219420776 |
| ZDHHC2 | 6.683167412 |
| ZDHHC20 | 5.674072445 |
| ZDHHC21 | 5.295186644 |
| ZDHHC22 | 5.907119489 |
| ZDHHC23 | 7.409747242 |
| ZDHHC24 | 6.915844282 |
| ZDHHC3 | 9.397869883 |
| ZDHHC4 | 5.846260013 |
| ZDHHC5 | 9.608323186 |
| ZDHHC6 | 8.979742868 |
| ZDHHC7 | 8.991899682 |
| ZDHHC8 | 10.26719713 |
| ZDHHC9 | 7.218141904 |
| ZEB2 | 7.899963418 |
| ZER1 | 6.019263261 |
| ZF | 7.658476199 |
| ZFAND1 | 10.10408768 |
| ZFAND2A | 11.86872337 |
| ZFAND2B | 9.469613473 |
| ZFAND3 | 7.690364182 |
| ZFAND5 | 12.77709776 |
| ZFAND6 | 10.55706661 |
| ZFAT | 6.759378132 |
| ZFHX4 | 5.945482886 |
| ZFP1 | 6.306007099 |
| ZFP106 | 8.556954476 |
| ZFP14 | 5.960783615 |
| ZFP161 | 8.907485211 |
| ZFP3 | 5.472326986 |
| ZFP30 | 6.034302959 |
| ZFP36 | 12.00554095 |
| ZFP36L1 | 10.71860575 |
| ZFP37 | 6.33086215 |
| ZFP64 | 7.037922263 |
| ZFP90 | 8.262048252 |
| ZFP91 | 9.36684586 |
| ZFP95 | 5.843380913 |
| ZFPL1 | 7.493274222 |
| ZFPM1 | 7.653520504 |
| ZFPM2 | 5.415802428 |
| ZFR | 10.07807417 |
| ZFX | 5.882301301 |
| ZFY | 6.326795276 |
| ZFYVE1 | 8.490515556 |
| ZFYVE16 | 6.77789045 |
| ZFYVE19 | 7.994760769 |
| ZFYVE20 | 10.82862511 |
| ZFYVE21 | 10.15265526 |
| ZFYVE26 | 7.713418151 |
| ZFYVE28 | 5.602943409 |
| ZFYVE9 | 5.88899787 |
| ZG16 | 8.179683321 |
| ZGPAT | 10.20509175 |
| ZHX1 | 11.73466383 |
| ZHX2 | 7.601008444 |
| ZHX3 | 8.593760498 |
| ZIM3 | 6.044645225 |
| ZKSCAN1 | 8.07257293 |
| ZKSCAN2 | 5.859315455 |
| ZKSCAN3 | 6.705081863 |
| ZKSCAN4 | 6.376368569 |
| ZMAT2 | 10.16218027 |
| ZMAT3 | 9.164483045 |
| ZMAT4 | 8.152462971 |
| ZMAT5 | 8.746540651 |
| ZMIZ1 | 10.32707253 |
| ZMIZ2 | 6.24188169 |
| ZMPSTE24 | 12.11403376 |
| ZMYM1 | 7.939563156 |
| ZMYM2 | 8.207090016 |
| ZMYM3 | 6.273667719 |
| ZMYM4 | 8.104125292 |
| ZMYM5 | 6.531187344 |
| ZMYM6 | 10.98945407 |
| ZMYND10 | 6.189840536 |
| ZMYND11 | 7.803751463 |
| ZMYND12 | 7.947845712 |
| ZMYND15 | 7.550562718 |
| ZMYND19 | 8.208215348 |
| ZMYND8 | 6.454569481 |
| ZNF10 | 5.97562181 |
| ZNF101 | 5.596116842 |
| ZNF12 | 7.375644898 |
| ZNF124 | 5.656834396 |
| ZNF132 | 5.760130702 |
| ZNF133 | 7.343423113 |
| ZNF134 | 6.222083539 |
| ZNF135 | 5.723461664 |
| ZNF136 | 6.242804442 |
| ZNF138 | 5.900324346 |
| ZNF14 | 11.66160267 |
| ZNF140 | 7.903311826 |
| ZNF142 | 8.138640072 |
| ZNF143 | 7.812736495 |
| ZNF146 | 6.915868803 |
| ZNF148 | 9.610773642 |
| ZNF154 | 5.944060823 |
| ZNF157 | 5.880293027 |
| ZNF16 | 5.855167908 |
| ZNF160 | 7.379402711 |
| ZNF165 | 6.577964421 |
| ZNF167 | 5.795189 |
| ZNF17 | 7.726215972 |
| ZNF174 | 6.812937698 |
| ZNF175 | 6.420061265 |
| ZNF18 | 7.570684529 |
| ZNF180 | 9.640631586 |
| ZNF181 | 6.995156385 |
| ZNF182 | 6.422991896 |
| ZNF184 | 5.784003482 |
| ZNF185 | 7.384354407 |
| ZNF187 | 7.592546959 |
| ZNF189 | 8.747322382 |
| ZNF192 | 5.660710086 |
| ZNF193 | 6.204109718 |
| ZNF195 | 7.53855619 |
| ZNF2 | 6.24195422 |
| ZNF20 | 7.436050376 |
| ZNF200 | 6.530408341 |
| ZNF202 | 5.878121055 |
| ZNF205 | 6.151400435 |
| ZNF207 | 11.0885991 |
| ZNF211 | 6.20592158 |
| ZNF212 | 7.345789663 |
| ZNF213 | 5.644681894 |
| ZNF214 | 5.589687397 |
| ZNF217 | 8.813225964 |
| ZNF218 | 8.249821288 |
| ZNF219 | 7.406665212 |
| ZNF22 | 10.69631142 |
| ZNF222 | 5.786809041 |
| ZNF223 | 9.575054039 |
| ZNF224 | 5.779032721 |
| ZNF226 | 8.571228768 |
| ZNF227 | 6.921188729 |
| ZNF228 | 6.878603152 |
| ZNF229 | 5.415405119 |
| ZNF23 | 7.116600888 |
| ZNF232 | 6.474116824 |
| ZNF234 | 6.557663097 |
| ZNF235 | 5.846780202 |
| ZNF238 | 6.336536174 |
| ZNF239 | 5.904969686 |
| ZNF24 | 7.24254557 |
| ZNF248 | 5.954651656 |
| ZNF25 | 8.166670851 |
| ZNF250 | 6.657507422 |
| ZNF251 | 5.810523591 |
| ZNF256 | 6.990562801 |
| ZNF259 | 8.836406728 |
| ZNF26 | 6.169058536 |
| ZNF260 | 7.160579711 |
| ZNF263 | 9.487161153 |
| ZNF264 | 7.293642296 |
| ZNF266 | 7.653288945 |
| ZNF268 | 6.140561396 |
| ZNF271 | 8.722307237 |
| ZNF274 | 7.803469839 |
| ZNF276 | 6.675160379 |
| ZNF277 | 10.04550654 |
| ZNF280D | 9.483161387 |
| ZNF281 | 10.13703642 |
| ZNF282 | 6.283948622 |
| ZNF283 | 6.525054577 |
| ZNF286A | 5.710383835 |
| ZNF294 | 8.468862706 |
| ZNF295 | 9.449709833 |
| ZNF296 | 5.778052697 |
| ZNF3 | 6.326980051 |
| ZNF30 | 6.523246505 |
| ZNF302 | 5.757727824 |
| ZNF304 | 6.113325652 |
| ZNF311 | 6.004631806 |
| ZNF312 | 6.005889959 |
| ZNF317 | 8.544405585 |
| ZNF318 | 8.52563843 |
| ZNF319 | 8.185917055 |
| ZNF32 | 8.01670206 |
| ZNF322B | 5.692070844 |
| ZNF323 | 8.604748869 |
| ZNF324 | 7.066056116 |
| ZNF324B | 6.224432645 |
| ZNF326 | 5.986245292 |
| ZNF329 | 8.512098712 |
| ZNF330 | 10.51120549 |
| ZNF331 | 7.560306528 |
| ZNF333 | 5.564149062 |
| ZNF334 | 5.861463549 |
| ZNF335 | 6.727537897 |
| ZNF337 | 6.485724579 |
| ZNF33A | 5.836521239 |
| ZNF33B | 8.367698924 |
| ZNF34 | 6.684001689 |
| ZNF341 | 6.113158913 |
| ZNF343 | 6.866222676 |
| ZNF35 | 7.223903814 |
| ZNF350 | 6.743147083 |
| ZNF354A | 6.226732041 |
| ZNF358 | 9.911336704 |
| ZNF362 | 7.639399696 |
| ZNF364 | 10.55448394 |
| ZNF366 | 6.131189477 |
| ZNF367 | 6.900323527 |
| ZNF37A | 6.215455315 |
| ZNF383 | 6.141341575 |
| ZNF384 | 6.032573676 |
| ZNF385C | 5.52038232 |
| ZNF394 | 6.495608964 |
| ZNF395 | 9.842765242 |
| ZNF397 | 5.638955866 |
| ZNF398 | 6.363202704 |
| ZNF408 | 6.552335627 |
| ZNF41 | 5.84883438 |
| ZNF410 | 9.061367104 |
| ZNF414 | 5.450342855 |
| ZNF415 | 5.719540852 |
| ZNF416 | 6.341431801 |
| ZNF417 | 6.632553779 |
| ZNF419 | 7.92989608 |
| ZNF426 | 6.580305176 |
| ZNF428 | 12.23298045 |
| ZNF429 | 5.589933485 |
| ZNF43 | 5.610359423 |
| ZNF432 | 6.493247005 |
| ZNF433 | 6.933290525 |
| ZNF434 | 8.17275866 |
| ZNF436 | 7.620746136 |
| ZNF438 | 8.49161663 |
| ZNF442 | 6.515318868 |
| ZNF444 | 7.737365971 |
| ZNF446 | 7.020444112 |
| ZNF45 | 6.938431774 |
| ZNF451 | 7.668396732 |
| ZNF452 | 6.144722278 |
| ZNF462 | 6.638538536 |
| ZNF467 | 8.501465587 |
| ZNF468 | 6.442266011 |
| ZNF470 | 5.815049605 |
| ZNF471 | 6.008991229 |
| ZNF473 | 5.599715264 |
| ZNF474 | 5.774120612 |
| ZNF480 | 7.855031467 |
| ZNF482 | 5.492732501 |
| ZNF483 | 5.75467349 |
| ZNF484 | 7.405829926 |
| ZNF485 | 6.262808686 |
| ZNF486 | 13.05611497 |
| ZNF491 | 5.695442961 |
| ZNF497 | 5.70109827 |
| ZNF498 | 6.531876115 |
| ZNF500 | 6.630029277 |
| ZNF503 | 7.729485687 |
| ZNF507 | 5.75563254 |
| ZNF509 | 7.010878103 |
| ZNF510 | 5.981738455 |
| ZNF511 | 11.42125009 |
| ZNF512 | 8.939644603 |
| ZNF512B | 6.897740094 |
| ZNF513 | 7.176203342 |
| ZNF514 | 5.768391089 |
| ZNF516 | 5.696806913 |
| ZNF518 | 7.520995573 |
| ZNF518B | 7.514383977 |
| ZNF521 | 6.274692182 |
| ZNF524 | 8.080795407 |
| ZNF526 | 7.183632465 |
| ZNF529 | 7.869700537 |
| ZNF532 | 6.162254073 |
| ZNF533 | 7.95185925 |
| ZNF537 | 6.746061687 |
| ZNF539 | 6.18485852 |
| ZNF541 | 8.769501935 |
| ZNF544 | 8.361841669 |
| ZNF545 | 5.679073184 |
| ZNF548 | 7.102259624 |
| ZNF551 | 5.720513605 |
| ZNF555 | 5.818468518 |
| ZNF556 | 5.728268021 |
| ZNF557 | 8.762238889 |
| ZNF558 | 7.246476163 |
| ZNF559 | 7.560136051 |
| ZNF561 | 5.836715257 |
| ZNF562 | 8.88621805 |
| ZNF564 | 7.12080786 |
| ZNF565 | 6.994786025 |
| ZNF569 | 8.285983558 |
| ZNF570 | 5.965627353 |
| ZNF573 | 7.523957624 |
| ZNF576 | 7.846432092 |
| ZNF579 | 5.67183756 |
| ZNF580 | 5.818474219 |
| ZNF581 | 8.245946224 |
| ZNF583 | 8.481459382 |
| ZNF584 | 5.379731803 |
| ZNF585A | 7.452894536 |
| ZNF585B | 5.709383702 |
| ZNF586 | 8.458640917 |
| ZNF592 | 5.865033067 |
| ZNF593 | 11.54021982 |
| ZNF595 | 6.139490837 |
| ZNF597 | 5.898978626 |
| ZNF600 | 6.439901664 |
| ZNF606 | 6.261208419 |
| ZNF608 | 5.708396693 |
| ZNF609 | 5.770911675 |
| ZNF613 | 8.274680701 |
| ZNF614 | 7.044593249 |
| ZNF615 | 7.310546364 |
| ZNF616 | 6.190394942 |
| ZNF618 | 6.129057647 |
| ZNF619 | 6.192683828 |
| ZNF621 | 8.200039238 |
| ZNF622 | 10.48826704 |
| ZNF624 | 6.408638171 |
| ZNF625 | 5.946914856 |
| ZNF626 | 7.154757739 |
| ZNF627 | 7.126398472 |
| ZNF629 | 6.637265682 |
| ZNF630 | 5.694775467 |
| ZNF638 | 8.33648966 |
| ZNF641 | 7.208301885 |
| ZNF642 | 5.679728503 |
| ZNF644 | 7.523632643 |
| ZNF646 | 5.871465992 |
| ZNF649 | 6.271786387 |
| ZNF650 | 6.713053575 |
| ZNF652 | 6.091476389 |
| ZNF653 | 8.574782793 |
| ZNF654 | 6.853120307 |
| ZNF655 | 7.999482609 |
| ZNF658 | 6.887914504 |
| ZNF664 | 10.49937252 |
| ZNF668 | 7.816475276 |
| ZNF669 | 5.868127502 |
| ZNF671 | 6.341619717 |
| ZNF672 | 9.322834076 |
| ZNF673 | 6.117839676 |
| ZNF675 | 5.732748448 |
| ZNF678 | 5.568740289 |
| ZNF679 | 5.418886214 |
| ZNF680 | 8.426099108 |
| ZNF683 | 6.370418705 |
| ZNF684 | 8.7268084 |
| ZNF688 | 7.275904675 |
| ZNF689 | 9.873176587 |
| ZNF69 | 7.557587958 |
| ZNF691 | 6.109204893 |
| ZNF696 | 7.422316641 |
| ZNF699 | 5.855286197 |
| ZNF7 | 8.019048754 |
| ZNF700 | 6.465294594 |
| ZNF701 | 5.653557046 |
| ZNF705A | 5.986175629 |
| ZNF706 | 8.332906262 |
| ZNF707 | 5.495293906 |
| ZNF710 | 6.065947893 |
| ZNF713 | 6.539631565 |
| ZNF717 | 6.115800669 |
| ZNF721 | 8.803933851 |
| ZNF74 | 5.69458607 |
| ZNF740 | 5.671382959 |
| ZNF746 | 8.875607146 |
| ZNF747 | 5.828192302 |
| ZNF75 | 6.166747819 |
| ZNF75A | 5.993445395 |
| ZNF76 | 6.168901773 |
| ZNF766 | 7.834554904 |
| ZNF77 | 6.725673959 |
| ZNF770 | 9.355720925 |
| ZNF772 | 5.635102018 |
| ZNF773 | 7.076655189 |
| ZNF780A | 5.731274712 |
| ZNF784 | 7.327244217 |
| ZNF786 | 9.187033638 |
| ZNF787 | 10.78475353 |
| ZNF789 | 6.347277154 |
| ZNF79 | 6.521156805 |
| ZNF791 | 7.988135828 |
| ZNF793 | 6.512202192 |
| ZNF8 | 6.064384036 |
| ZNF800 | 9.412714145 |
| ZNF816A | 6.769364768 |
| ZNF821 | 5.950225152 |
| ZNF828 | 9.741739117 |
| ZNF83 | 7.302997549 |
| ZNF84 | 7.56618906 |
| ZNF85 | 5.727185466 |
| ZNF91 | 8.725452891 |
| ZNF93 | 7.028770099 |
| ZNFN1A1 | 5.826616911 |
| ZNFN1A5 | 7.31705491 |
| ZNFX1 | 8.235718059 |
| ZNHIT1 | 12.32424736 |
| ZNHIT2 | 7.755205521 |
| ZNHIT3 | 10.90481554 |
| ZNHIT4 | 7.200587843 |
| ZNRD1 | 9.363581104 |
| ZNRF1 | 5.700128496 |
| ZNRF2 | 7.22982094 |
| ZNRF3 | 5.897056843 |
| ZP3 | 6.715616606 |
| ZP4 | 5.621722653 |
| ZRANB1 | 7.895049297 |
| ZRANB2 | 9.495649557 |
| ZRANB3 | 5.733362713 |
| ZSCAN16 | 7.947524065 |
| ZSCAN18 | 8.368805585 |
| ZSCAN2 | 6.256799648 |
| ZSCAN21 | 9.330878407 |
| ZSCAN5A | 6.96082411 |
| ZSWIM1 | 8.950321791 |
| ZSWIM4 | 6.26520483 |
| ZSWIM5 | 5.556428382 |
| ZSWIM6 | 8.931527995 |
| ZUFSP | 7.981816702 |
| ZW10 | 6.597169408 |
| ZWILCH | 5.404013999 |
| ZWINT | 6.445249885 |
| ZXDA | 5.628172748 |
| ZXDB | 9.244859006 |
| ZXDC | 8.081680855 |
| ZYG11B | 11.30900434 |
| ZYX | 10.45002947 |
| ZZEF1 | 8.680974356 |
| ZZZ3 | 9.493198731 |
