# Supplementary material for: Accumulation of Deleterious Passenger Mutations Is Associated with the Progression of Hepatocellular Carcinoma
Source: PLoS One. 2016 Sep 15;11(9):e0162586. doi: 10.1371/journal.pone.0162586 (PMC5025244; doi:10.1371/journal.pone.0162586)
Supplement: S3 Table — (DOCX) [file pone.0162586.s011.docx]

| **Table S3. Frequency of mutations in putative driver genes in tumour and non-tumour samples** | | | | | | | | | | | | | | |  |  |  |  |  |  |  |  |
| --- | --- | --- | --- | --- | --- | --- | --- | --- | --- | --- | --- | --- | --- | --- | --- | --- | --- | --- | --- | --- | --- | --- |
|  |  |  |  | |  |  |  |  |  |  |  |  |  |  |  |  |  |  |  |  |  |  |
|  |  | **TERT** | **TP53** | | **CTNNB1** | **AXIN1** | **CDKN2A** | **ARID1A** | **ARID2** | **PIK3CA** | **HNF1A** | **IL6ST** | **PTEN** | **BRAF** | **NFE2L2** | **MLL3** | **SMARCA4** | **ATM** | **KRAS** | **RB1** | **JAK1** | **BRCA2** |
| **Damaging** | **WES1*** | 0/0 | 0/0 | | 0/0 | 0/0 | 0/0 | 0/0 | **1/0** | 0/0 | 0/0 | 0/0 | 0/0 | **0/1** | 0/0 | 0/0 | 0/0 | **1/1** | 0/0 | **0/1** | 0/0 | **0/1** |
|  | **WES2**** | **0/2** | **0/2** | | **0/11** | **1/5** | 0/0 | **0/4** | **0/3** | 0/0 | **0/2** | 0/0 | 0/0 | 0/0 | **2/3** | 0/0 | **5/6** | **1/2** | 0/0 | **1/2** | 0/0 | **2/1** |
|  | **WES3**** | 0/0 | **2/6** | | **2/3** | **0/1** | 0/0 | **1/1** | 0/0 | 0/0 | 0/0 | **1/1** | 0/0 | 0/0 | 0/0 | 0/0 | **0/2** | **1/1** | 0/0 | **1/1** | 0/0 | **2/2** |
|  | **WES4**** | **1/1** | **0/7** | | **0/4** | **0/1** | 0/0 | 0/0 | 0/0 | 0/0 | 0/0 | 0/0 | 0/0 | **0/1** | 0/0 | 0/0 | **1/0** | 0/0 | 0/0 | **0/1** | **0/1** | 0/0 |
|  |  |  |  | |  |  |  |  |  |  |  |  |  |  |  |  |  |  |  |  |  |  |
| **Benign** | **WES1*** | 0/0 | 0/0 | | 0/0 | **0/2** | 0/0 | 0/0 | 0/0 | 0/0 | 0/0 | 0/0 | 0/0 | 0/0 | 0/0 | 0/0 | 0/0 | 0/0 | 0/0 | 0/0 | 0/0 | 0/0 |
|  | **WES2**** | **0/1** | **2/1** | | 0/0 | 0/0 | 0/0 | 0/0 | 0/0 | 0/0 | 0/0 | 0/0 | 0/0 | 0/0 | 0/0 | 0/0 | 0/0 | 0/0 | 0/0 | 0/0 | 0/0 | 0/0 |
|  | **WES3**** | 0/0 | 0/0 | | **2/2** | 0/0 | 0/0 | **1/1** | 0/0 | 0/0 | 0/0 | **1/0** | 0/0 | 0/0 | 0/0 | 0/0 | 0/0 | 0/0 | 0/0 | **1/1** | **1/0** | **2/2** |
|  | **WES4**** | 0/0 | 0/0 | | **0/1** | **1/1** | 0/0 | **1/2** | 0/0 | 0/0 | 0/0 | 0/0 | 0/0 | 0/0 | 0/0 | 0/0 | 0/0 | 0/0 | 0/0 | 0/0 | 0/0 | 0/0 |
| * Non-cirrhosis/Cirrhosis  ** Non-tumour/Tumour | | |  | |  |  |  |  |  |  |  |  |  |  |  |  |  |  |  |  |  |  |
| Oncogene | |  |  | |  |  |  |  |  |  |  |  |  |  |  |  |  |  |  |  |  |  |
| Tumor Suppressor gene | | | |  | | |  |  |  |  |  |  |  |  |  |  |  |  |  |  |  |  |
|  |  |  | | | | |  |  |  |  |  |  |  |  |  |  |  |  |  |  |  |  |
