# Supplementary material for: Accumulation of Deleterious Passenger Mutations Is Associated with the Progression of Hepatocellular Carcinoma
Source: PLoS One. 2016 Sep 15;11(9):e0162586. doi: 10.1371/journal.pone.0162586 (PMC5025244; doi:10.1371/journal.pone.0162586)
Supplement: S4 Table — (DOCX) [file pone.0162586.s012.docx]

**Table S4. Significantly enriched (p≤10^-3^) canonical pathways in DPM-affected genes.**

| **Dataset** | **Patient group** | **DPMs analysed** | **Canonical pathway** | **Pathway overlap** | **P value** | **Total enriched genes**  **(% of total DPMs)** |
| --- | --- | --- | --- | --- | --- | --- |
| **WES 1** | **NC** | 1238 | None | N/A | N/A | 0 (0%) |
|  | **C** | 818 | Nur77 Signalling in T Lymphocytes | 5/29 | 8.78 x 10^-3^ | 5 (0.61%) |
| **WES 2** | **NT** | 2362 | Notch Signalling  AMPK Signalling  Cell Cycle: G2/M DNA Damage Checkpoint Regulation | 8/19  21/90  11/38 | 1.76 x 10^-3^  5.82 x 10^-3^  8.13 x 10^-3^ | 40 (1.7%) |
|  | **T** | 3162 | AMPK Signalling  ATM Signalling  Notch Signalling  Role of CHK Proteins in Cell Cycle Checkpoint Control  Role of BRCA1 in DNA Damage Response | 32/90  18/41  10/19  13/29  16/41 | 3.48 x 10^-5^  8.28 x 10^-5^  5.63 x 10^-4^  6.31 x 10^-4^  1.00 x 10^-3^ | 89 (2.8%) |
| **WES 3** | **NT** | 2495 | Phospholipases | 9/29 | 8.11 x 10^-3^ | 9 (0.36%) |
|  | **T** | 3361 | Gustation Pathway  PPAR/RXR Activation  Cardiac -adrenergic Signalling  Stearate Biosynthesis I (Animals) | 18/46  33/120  20/64  10/25 | 7.53 x 10^-4^  8.36 x 10^-3^  8.58 x 10^-3^  9.37 x 10^-3^ | 81 (2.4%) |
| **WES 4** | **NT** | 2741 | Notch Signalling  AMPK Signalling  Cell Cycle: G2/M DNA Damage Checkpoint Regulation | 8/19  21/90  11/38 | 1.76 x 10^-3^  5.82 x 10^-3^  8.13 x 10^-3^ | 12 (0.44%) |
|  | **T** | 3479 | Factors Promoting Cardiogenesis in Vertebrates | 17/49 | 7.98 x 10^-3^ | 17 (0.49%) |
